# Optical coherence tomography angiography

**DOI:** 10.1016/j.preteyeres.2017.11.003

**Published:** 2017-12-08

**Authors:** Richard F. Spaide, James G. Fujimoto, Nadia K. Waheed, Srinivas R. Sadda, Giovanni Staurenghi

**Affiliations:** a Vitreous, Retina, Macula Consultants of New York, New York, NY, United States; b Department of Electrical Engineering & Computer Science and Research Laboratory of Electronics, Massachusetts Institute of Technology, Cambridge MA, United States; c The Department of Ophthalmology, Tufts University School of Medicine, Boston MA, United States; d Doheny Eye Institute, University of California – Los Angeles, Los Angeles, CA, United States; e Eye Clinic, Department of Biomedical and Clinical Sciences “Luigi Sacco”, Luigi Sacco Hospital, University of Milan, Milan, Italy

**Keywords:** Multimodal imaging, Optical coherence tomography, Optical coherence tomography angiography

## Abstract

Optical coherence tomography (OCT) was one of the biggest advances in ophthalmic imaging. Building on that platform, OCT angiography (OCTA) provides depth resolved images of blood flow in the retina and choroid with levels of detail far exceeding that obtained with older forms of imaging. This new modality is challenging because of the need for new equipment and processing techniques, current limitations of imaging capability, and rapid advancements in both imaging and in our understanding of the imaging and applicable pathophysiology of the retina and choroid. These factors lead to a steep learning curve, even for those with a working understanding dye-based ocular angiography. All for a method of imaging that is a little more than 10 years old. This review begins with a historical account of the development of OCTA, and the methods used in OCTA, including signal processing, image generation, and display techniques. This forms the basis to understand what OCTA images show as well as how image artifacts arise. The anatomy and imaging of specific vascular layers of the eye are reviewed. The integration of OCTA in multimodal imaging in the evaluation of retinal vascular occlusive diseases, diabetic retinopathy, uveitis, inherited diseases, age-related macular degeneration, and disorders of the optic nerve is presented. OCTA is an exciting, disruptive technology. Its use is rapidly expanding in clinical practice as well as for research into the pathophysiology of diseases of the posterior pole.

## 1. Introduction

Although still in development, optical coherence tomography (OCT) angiography (OCTA) can produce images of blood flow that have unprecedented resolution of all the vascular layers of the retina in a rapid, non-invasive fashion. Fluorescein angiography (FA), an alternate method of imaging flow, has been used in clinical practice for over 50 years. Unfortunately, FA cannot image several important layers of blood vessels in the eye; essentially only the superficial vascular plexus can be seen. Still, FA was used to create the field of medical retina. OCT angiography (OCTA) offers the possibility of also imaging the radial peripapillary capillary network and the intermediate and deep capillary plexuses. This capability opens a wealth of possibilities for disease description and quantification, research into pathogenesis of disease, and development and evaluation of new treatments. OCTA comes at the heels of development of higher speed structural OCT imaging, which itself is under rapid development. Curiously much of the recent drive to develop instrumentation platforms is to improve OCTA. Concurrently the scanning strategies and software being developed for OCTA are improving quickly. While the rate of change is breathtaking and exciting, the challenge becomes trying to understand what OCTA is and what it can accomplish. Understanding OCTA requires a comprehensive knowledge of many facets of imaging, starting with how the imaging works, what the potential deficiencies as well as strengths are. This paper is an in-depth review of OCTA and serves as a guide to understand OCTA in a practical way.

Many OCT instrument manufacturers are developing OCT angiography instruments. Custom made research prototype devices exist as well. The authors have experience with most of these instruments. The hardware and software of these instruments are changing rapidly. As such, specific recommendations are constrained to the time period when the particular software or hardware version of the instrument was in use, which is likely to be for only a short time. In light of this, the assessment below will try to be as generic as possible, but the reader should be aware that features and performance of specific OCTA instruments may change.

OCT generates images by interferometrically measuring the amplitude and delay of reflected or backscattered light. A beam of light is scanned on the retina or anterior eye and depth ranging is performed by interfering the reflected or backscattered from ocular structures with light that has travelled a known reference path (Huang et al., 1991). This method is a modification of classic Michelson interferometry. The interferograms that are generated vary in intensity with the amount of light reflected from a structure in the eye and the frequency of the interference fringes give information about the delay or optical path length compared with the reference path. Stated another way, OCT measures the depth of a given structure within the tissue as well as how much it reflects or scatters light. This measurement is known as an axial scan or A-scan, in analogy with ultrasound and because early OCT instruments used a scanning reference delay. A B-scan or cross sectional image is generated by sequentially acquiring many A-scans as the light beam is scanned in the transverse direction. Volumetric information is generated by sequentially acquiring multiple B-scans which are displaced perpendicular to the B-scan image, covering a region of the retina or anterior eye using raster scan. The retina is a stationary object for the most part, so if successive B-scans at the same position are acquired, they will be largely similar, except for the motion of blood within the tissue. At the sites of blood flow, the reflectivity or scattering changes from one scan to the next. By comparing repeated OCT B-scans, it is possible to image blood flow by looking for differences among the scans on a pixel-by-pixel basis. How the scans are acquired, how differences are determined, and what constitutes a difference are the core issues in OCTA. The technology and methods of OCTA have been investigated over the last ten years, have been applied in multiple research and clinical disciplines, and translated into a variety of instruments for ophthalmology.

## 2. Review of OCTA development

### 2.1. Doppler OCT and the origins of OCTA

The development of OCTA has a long history which predates commercial development in ophthalmology by more than ten years. The earliest studies to detect and measure blood flow used classic Doppler techniques, which compared the phase of successive OCT A-scans (Chen et al., 1997; Izatt et al., 1997). Doppler has limited ability to visualize retinal vasculature because blood flow is predominantly in the en face direction, perpendicular to the OCT beam. Classic Doppler OCT can quantitatively measure blood velocity along the direction of the OCT beam, however, measurements of blood flow require knowledge of the Doppler angle, the angle between the blood vessel and the light beam. The frequency shift of the interferometric fringe is measured over time. The sensitivity to motion depends on how long the Doppler signal is sampled at any given spot. Longer sampling times yields better sensitivity at the cost of decreased scan speed and decreased spatial resolution. More than 15 years ago, it was recognized that blood vessels could be visualized using Doppler OCT with time domain OCT by extracting and comparing the A-scan phase changes which are related to the Doppler frequency shift (Zhao et al., 2000). This method was known as Optical Doppler Tomography and was demonstrated for dermatological imaging of blood vessels in the skin. High dynamic range Doppler measurements were achieved using time domain OCT with a high speed scanning at 8000 A-scans per second (Yang et al., 2003a) to image cardiac dynamics in the Xenopus laevis (Yang et al., 2003d) and blood flow in the rat and human gastrointestinal tracts using endoscopic access (Yang et al., 2003c). With the development of spectral domain OCT (SD-OCT), the phase of the A-scans became directly accessible and imaging speeds increased. Pulsatile blood flow was measured in the retina using SD-OCT at a 29,000 A-scan rate (White et al., 2003). As early as 2005, researchers demonstrated that blood flow could be visualized using swept source OCT (SS-OCT) by measuring the variance of the Doppler signal and intensity variation without phase, to visualize blood flow in phantoms and the chick chorioallantoic membrane (Zhang and Chen, 2005).

### 2.2. Ophthalmic OCTA

One of the first demonstrations visualizing the vasculature in the human eye was performed in 2006, using a method known as Optical Coherence Angiography. After compensating for bulk eye motion, Doppler phase as well as variance or power of the phase between successive A-scans was measured at 18,700 A-scans per second (Makita et al., 2006). This study demonstrated several important advances which are used in current ophthalmic OCTA; volumetric data was acquired; retinal layers were segmented and the vascular structure of the retina and choriocapillaris were displayed in en face images. However, phase based detection is very sensitive to bulk eye motion as well as motion of other structures within the eye not directly related to blood flow. An early method, known as Optical Microangiography (also commonly called OMAG) addressed the problem of bulk eye motion by modifying OCT hardware to introduce a modulation or externally imposed phase variation which improved the differentiation between stationary tissue and blood flow (Wang et al., 2007a). These methods were demonstrated in brain science for visualizing cerebral vasculature through the intact skull in the mouse (Wang and Hurst, 2007). Imaging of human retinal and choroidal vasculature using OMAG was demonstrated in 2008 (An and Wang, 2008).

With the increased speed that was enabled by SS-OCT, speckle variance detection methods were demonstrated by comparing repeated B-scans rather than A-scans. In 2008, using a swept light source operating at 43,000 to 67,000 A-scans per second, imaging of microvasculature in a rat dorsal skin flap model was demonstrated by calculating speckle variance, a measure of amplitude variation, between 3 repeated B-scans (Mariampillai et al., 2008). Other investigators using SD-OCT at 25,000 A-scans per second, demonstrated imaging of retinal microvasculature using phase variance between multiple B-scans which are slightly displaced during raster scanning (Fingler et al., 2009). This study described the need to motion correct successive B-scans as well to threshold the phase variance data in order to remove unwanted signals from bulk eye motion. These concepts are used in current ophthalmic OCTA methods.

With advances in SD-OCT imaging speeds, phase variance OCT imaging of retinal microvasculature was demonstrated at 125,000 Ascans per second in 2011 (Kim et al., 2011). These increases in speed were important in order to enable imaging larger regions, while reducing the effects of bulk eye motion which produce unwanted OCTA signals. This study demonstrated imaging over 3 mm × 3 mm regions as well as stitching multiple regions in order to increase the field of view. Current commercial instruments operate at comparable speeds, but image larger regions.

### 2.3. Commercialization

Multiple algorithms and scan protocols were investigated based on logarithmic intensity and speckle contrast for imaging retinal microvasculature with SS-OCT at 1050 nm wavelength and 50,000 A-scans per second (Motaghiannezam and Fraser, 2012). In 2012, split spectrum amplitude decorrelation (SSADA) was demonstrated to improve signal to noise and reduce sensitivity to bulk eye motion (Jia et al., 2012b). This algorithm uses signal processing methods to divide the OCT spectrum into multiple narrow band spectra, which reduces the axial image resolution in order to reduce sensitivity to eye motion and match the transverse OCT image resolution. Speckle decorrelations are calculated on a B-scan to B-scan basis between the split spectral data and then combined to generate a single data set with increased signal to noise. There has been rapid commercial development of OCTA in recent years. Optovue introduced the first commercial OCTA product, the AngioVue which was based on SD-OCT and released outside the US in 2014. The first commercial SS-OCT instruments were introduced by Topcon as the Atlantis and Triton product line, which image at 1050 nm wavelength and 100,000 A-scans per second. Zeiss introduced the AngioVue OCTA using the SD-OCT platform and more recently, the AngioPlex OCTA using SS-OCT. The development of vertical cavity surface emitting lasers (VCSEL) enabled imaging speeds of 400,000 A-scans per second suggesting that higher imaging speeds will be commercially available in the future (Choi et al., 2013a; Grulkowski et al., 2012; Moult et al., 2014). The commercialization of OCTA is an important milestone because it made the technology widely available to the clinical research community and dramatically accelerated progress.

## 3. How OCT angiography works

### 3.1. Brief overview

**Fig. 1.**
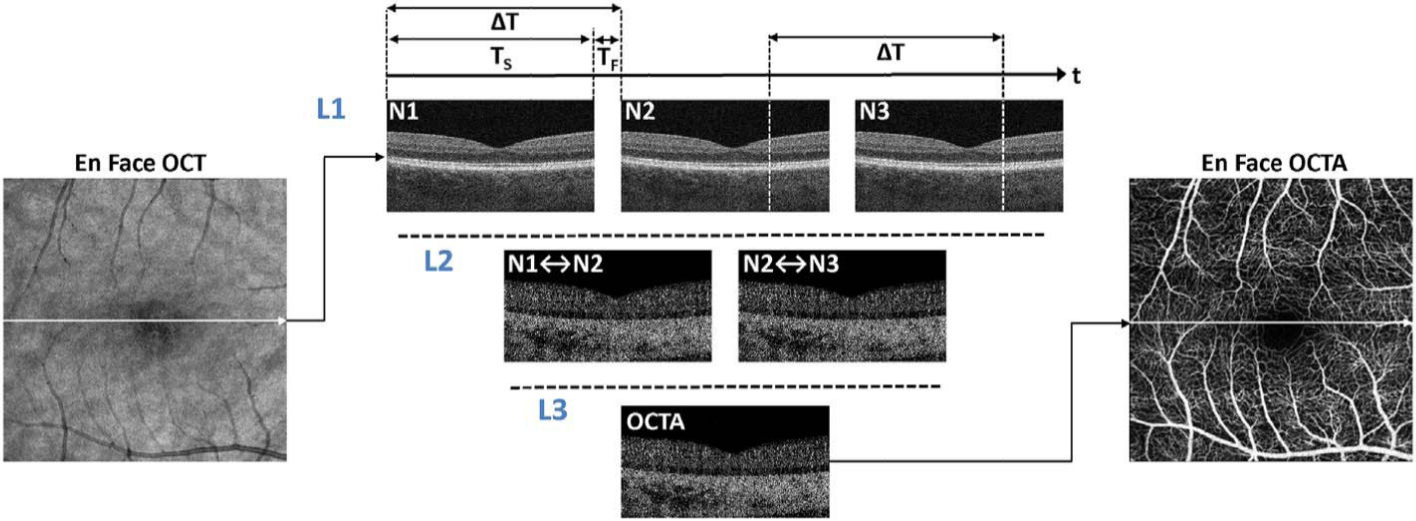
Simplified schematic of how optical coherence tomography angiography (OCTA) works. OCTA visualizes vasculature by detecting motion contrast from moving blood cells. Repeated B-scans (N1, N2, N3) are acquired from the same retinal location (Line L1) and differences or decorrelations between successive B-scans are calculated (Line L2). The decorrelation data is combined into an OCTA cross sectional image (Line 3) and the procedure is repeated at successive positions to generate a volumetric data set. The acquisition time (T_S_) for each B-scan is determined by the A-scan rate times the number of A-scans per B-scan. The OCT beam is rapidly scanned back to the initial positon during the fly back time (T_F_) and the B-scan is repeated after a time delay (ΔT), the interscan time determined by the sum of the acquisition and fly back times. Each A-scan which composes a B-scan is also repeated at the interscan time. The interscan time is an important parameter which determines OCTA sensitivity and saturation behavior.

OCTA is a functional extension of structural OCT which performs repeated B-scans to detect motion contrast and visualize vasculature. Stationary objects won’t produce much of a change from one image to the next, while moving objects will. By looking for change over time, an image can be created that highlights movement. Since the only expected motion in the retina is blood flow in vessels, the net expected result is to visualize blood flow. As with any simplification to actual practice, implementation decisions have to be made which can lead to differences in function and performance. These implementation decisions include how flow is detected, what defines a difference between B-scans, how bulk eye motion not caused by blood flow is managed, and how results are displayed. Each of these in turn prompts a series of hardware and software decisions, each having some type of tradeoff in terms of performance, potential problems, or cost. Fig. 1 shows a simplified schematic of how OCTA works compared with structural OCT imaging. Conventional structural OCT imaging acquires a 3D volume or image cube by performing successive B-scans at different retinal locations in a “raster” scan pattern. The raster scan covers an area of the fundus with a high density of A-scans. However, in order to detect motion and generate vascular contrast, it is necessary to repeatedly image the same retinal area multiple times. Multiple repeated B-scans are performed in the same location (row L1, N1 to N3) and the structural images are compared on a pixel by pixel basis in order to detect signal changes which occur because of flowing erythrocytes. The changes between repeated B-scans are then displayed as a motion contrast image (rows L2 and L3). The repeated B-scan can be compared in pairwise fashion or in various combinations using algorithms to obtain a motion contrast image (row L3). In order to generate volumetric OCTA data over an area of the retina, repeated B-scans are performed at successively displaced locations in the retina, following a raster scan pattern. The OCTA volume enables 3D visualization of the microvasculature and is typically displayed by segmenting the different retinal layers and projecting an en face view in analogy with fluorescein or indocyanine green angiography (ICGA).

### 3.2. Advantages and limitations of OCTA

OCTA has the advantage that it can visualize microvasculature with depth resolution, similar to structural OCT. In contrast to FA or ICGA, OCTA images are not obscured by hyper-fluorescence from dye leakage and therefore OCTA can generate high contrast, well-defined images of the microvasculature. This high quality image data is amenable to software based image processing which can provide quantitative markers for vascular pathology. Volumetric data can be segmented and OCTA from different retinal layers can be projected to enable separate visualization of retinal capillary plexuses and the choriocapillaris, as well as visualizing vascular pathologies including neovascularization and alterations in retinal capillary as well as choriocapillaris structure. In addition, OCTA images can be viewed in cross-section to confirm the depth location of vascular pathology. Structural OCT data is acquired simultaneously with OCTA data and therefore it is possible to display en face and cross-sectional structural OCT images which are intrinsically co-registered with OCTA data. Since OCTA does not require administration of exogenous contrast, it can be performed at any patient visit, when FA or ICGA would not be indicated. OCTA can also be performed repeatedly during a single imaging session to obtain comprehensive, wide field information on microvasculature or to assess microvascular response to functional stimulation. Finally, OCTA can be performed much more rapidly than FA or ICGA, streamlining clinical workflow.

At the same time, OCTA has important limitations. Since it visualizes microvasculature using motion contrast, imaging protocols require re-scanning the same retinal position multiple times. Therefore OCTA requires higher imaging speeds (A-scan rates) or longer imaging times than structural OCT. Imaging larger fields of view is especially challenging since the area and number of A-scans scales as the square of the dimension. OCTA cannot assess alterations in vascular permeability or leakage, which are typically visualized using FA or ICGA. In addition, OCTA signals have limited dynamic range. Current technology and OCTA methods provide limited quantitative information about actual blood flow, visualizing instead the structure of the vascular network. The appearance of OCTA image data is highly dependent on details of the OCT instrument, scan protocols, signal processing, and methods used to generate OCTA information from structural OCT data. Algorithms and display methods can vary dramatically between different instrument manufacturers and are often proprietary. Therefore, special care is required when comparing results between different instruments. Finally, OCTA images can exhibit many more types of artifacts than structural images and are therefore subject to misinterpretation (Cole et al., 2017a; Falavarjani et al., 2016; Spaide et al., 2015a). However, with understanding and careful interpretation, OCTA can provide powerful insight into disease pathogenesis and new surrogate markers for diagnosis, assessing disease progression and treatment response.

## 4. Details of OCT angiography signal processing and image generation

The previous section presented a brief overview of how OCTA works, but in order to develop a deeper understanding of OCTA, which is necessary for expert interpretation, it is important to consider OCTA data acquisition and processing in more detail.

### 4.1. Repeated scanning to generate motion contrast

In order to detect motion from blood flow, it is necessary to repeatedly image the same retinal location. OCT data is acquired in units of B-scans and therefore OCTA involves repeated B-scans at the same retinal location. A B-scan is performed with the OCT beam starting at one edge of the imaged region and successive A-scans are acquired as the OCT beam is scanned in the transverse direction across the imaged region as shown by the arrow in the en face OCT image of Fig. 1. Acquiring a B-scan image takes a time T_S_ and the A-scans which compose the B-scan image are acquired at sequential positions and at sequential times as the OCT beam is scanned. The A-scans are acquired at an Ascan rate determined by the OCT instrument. Typical commercial SDOCT instruments acquire approximately 70,000–85,000 A-scans per second (70–85 kHz A-scan rate), while SS-OCT instruments acquire 100,000 A-scans per second (100 kHz). Research instruments can operate at substantially faster acquisition speeds.

Before the B-scan can be repeated, the OCT beam must be rapidly scanned back to the initial positon without acquiring data. This is known as the fly back and requires a time, T_F_, which can be 10%–20% of the total time required to acquire the B-scan. The *acquisition time*, T_S_, for each B-scan is given by the instrument A-scan rate times the number of A-scans per B-scan. The B-scans are repeated after a time delay ΔT, the *interscan time*, which is equal to the sum of the acquisition time and the *fly back* time. Note that repeated A-scans at corresponding positions on the fundus are acquired at times separated by the interscan time.

Typical commercial instruments use OCTA acquisition protocols with two to four repeated B-scans (although the Heidelberg Spectralis uses a larger number of repeated scans), while high speed research OCTA instruments can use protocols with more repeated B-scans. OCTA volumes therefore require two or more times the data than structural OCT volumes. Alternately, in order to acquire the same volume in the same time, OCTA requires A-scan rates which are multiples of what would be required for simple structural OCT images.

### 4.2. Interscan time determines OCTA sensitivity and saturation

The interscan time, ΔT, plays a critical role in OCTA motion contrast detection (Braaf et al., 2012; Choi et al., 2015; Tokayer et al., 2013). If other parameters are kept constant, longer interscan times increase sensitivity to motion because more time has elapsed between the repeated B-scans. However, longer interscan times also mean that bulk eye motion can occur and overwhelm the motion signal from flowing blood. Conversely, shorter interscan times will decrease sensitivity to motion, but also reduce unwanted effects from bulk eye motion. Shorter interscan times are also more sensitive to flow impairment (Moult et al., 2016). Different interscan times change the range of flows that OCTA detects and using different interscan times can discriminate relative blood flow speeds, a technique known as variable interscan time analysis (VISTA) (Choi et al., 2015; Moult et al., 2016). Commercial instruments have interscan times of 4–5 ms, while high speed research instruments can have interscan times of 1.5 ms or less. The interscan time is governed by: (1) the A-scan rate of the instrument, (2) the Ascan sampling density (i.e., number of A-scans per unit distance), and (3) the B-scan dimension (i.e, the length of the B-scan). Slower A-scan rates imply longer interscan times for a given A-scan density and B-scan dimension.

### 4.3. Registration of repeated B-scans to correct eye motion

**Fig. 2.**
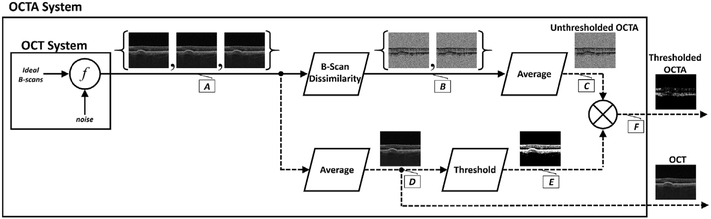
Detailed flowchart of OCTA processing. Repeated Bscans (block A) are acquired from the same location. The Bscans have noise which is combined with the image (represented by f). Motion contrast is generated by calculating dissimilarity/decorrelation between pairs of B-scans (block B) and an average is generated (block C) to increase the signal to noise. Repeated B-scans (block A) are also averaged to generate an OCT image with increased signal to noise (block D). The OCT image signal (block D) is also measured on a pixel by pixel basis and if the signal is below a threshold, then a mask (block E) is generated. This threshold mask is then used to remove invalid pixels from the OCTA image (block F) which are associated with low or noisy OCT pixels. The masked OCTA image is then displayed. OCTA images are highly dependent on processing details and parameters, however, this information is proprietary in commercial instruments (From Cole et al., 2017).

Fig. 2 shows a flowchart of OCTA processing which describes details of the processing in Fig. 1. The interscan times used in commercial OCTA instruments are long enough that eye motion can accumulate in the time between repeated B-scans (i.e., during the interscan time); and this eye motion will generate an OCTA signal of its own, obfuscating the OCTA signal generated by the flowing blood cells. In particular, there are movements of the eye by microsaccades and by expansion and contraction of the uvea from fluctuations in intraocular pressure during the cardiac cycle. To understand the effects of bulk eye motion which occurs during the interscan time, it is important to consider the optical resolution of the instrument, which, in commercial OCTA systems, are between 5 and 10 μm in the axial direction, and ~20 μm in the transverse direction (where these resolutions are the full-width-at-half-maximum, in tissue). While improved (i.e., smaller) axial and transverse resolutions are useful for resolving different vascular features in the eye, and also make the system more sensitive to blood flows, there is a tradeoff, because finer resolutions also make the system more sensitive to unwanted bulk eye motion. Improved axial resolution improves the differentiation of retinal architecture, but increases sensitivity to eye motion. To reduce the OCTA signal artifacts generated by bulk eye motion, the repeated B-scans can be registered to one another, either rigidly or non-rigidly, prior to OCTA computation. However, it is important to note that registration of repeated B-scans can only correct for motion occurring within the B-scan image plane; motion occurring out of the B-scan image plane, perpendicular to the OCT beam scan direction, cannot be corrected. Fortunately, the largest contribution from bulk eye motion is typically in the axial direction.

### 4.4. How blood flow affects OCT signals

**Fig. 3.**
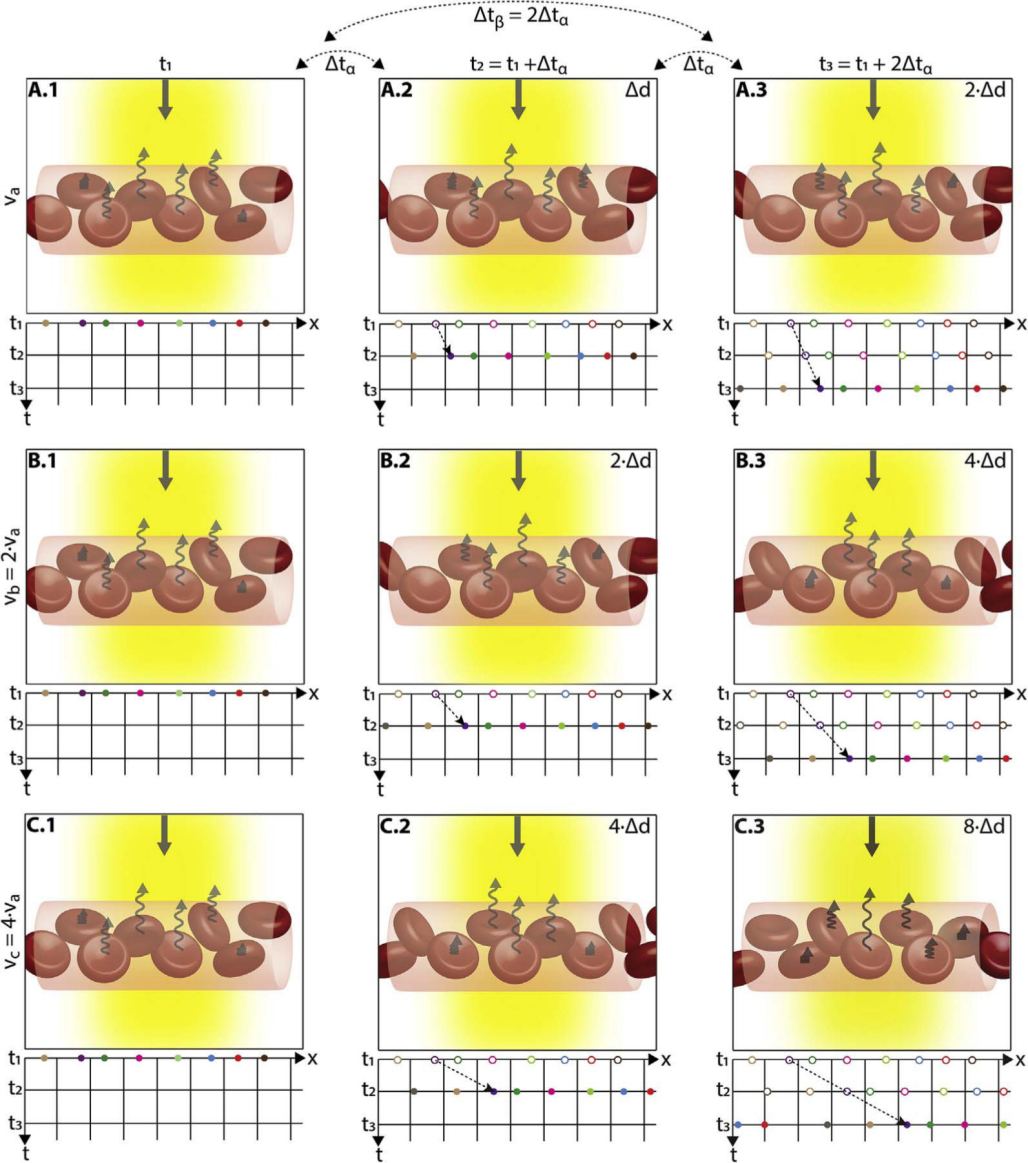
Illustration of how blood flow speed and interscan time affect OCTA signal. The transverse width of the OCT beam is shown in yellow. Black squiggly arrows indicate light backscattered from red blood cells. The rows correspond to different blood flow speeds (v_a_, v_b_, and v_c_, increasing by factors 2x in this example) and columns correspond to three equally-separated time points of the repeated A-scans (t_1_, t_2_, and t_3_). The time between repeated A-scans is Δt_α_; the time between the first and third A-scans is Δt_β_ = 2 × Δt_α_. The left panels (A.1, B.2, and C.1) are all identical, representing the initial positions of the cells; while subsequent columns show the displaced blood cells (Δd, 2Δd, 4Δd). The graphs under each panel show how the positions of the cells (x) change with repeated A-scans (different colored dots showing different cells). The displacement of the blood cells depends on flow speed and interscan time (displacement = speed × interscan time). Measurements with twice the interscan time Δt_β_ = 2 × Δt_α_ (i.e., first-to-third column) are equivalent to doubling the blood flow speed for an interscan time Δt_α_; thus, A.3 is identical to B.2, and B.3 is identical to C.2. When flow is fast v_c_ and the interscan time is long Δt_β_, a (purple) cell at the edge of the OCT beam and passes nearly to the other side (C.1 to C.3); this corresponds to the maximum distinguishable speed, or the saturation speed for that interscan time. Conversely, when flow is slow v_a_ and interscan time short Δt_α_, the cell translates very little during the interscan time (A.1 to A.2); this corresponds to the slowest detectable speed for that interscan time. By shortening the interscan time from Δt_β_ to Δt_α_ (C.3 to C.2), the cell travels half the distance and so the saturation speed is halved and it is possible to distinguish differences in flow; conversely by lengthening the interscan time from Δt_α_ to Δt_β_ (C.2 to C.3), the cell travels twice the distance and so the slowest detectable speed is halved and sensitivity is improved.

After bulk eye motion is compensated by registering the repeated B-scans, blood cell motion is detected by a pixel-by-pixel comparison of the B-scans. Fig. 3 shows a schematic which shows how interscan time affects the detection of blood flow. Long interscan times improve sensitivity to the slowest detectable flow, but also have a low saturation limit and cannot distinguish differences in faster flows. Conversely, short interscan times have poor sensitivity to detect slow flows, but have a higher saturation limit to distinguish differences in flows. Each B-scan is composed of multiple A-scans which are sequentially acquired, so for simplicity it is helpful to consider an A-scan at a given position within a B-scan. Fig. 3 shows three repeated A-scan acquisitions, separated by the interscan time. The A-scans shown are at the same position in the repeated B-scans and are also at the same retinal position. The transverse dimension of the OCT beam is shown schematically in yellow. If the OCT beam intercepts vasculature, the blood cells will contribute to the OCT signal. In order to produce a measurable change in the OCT signal, the blood cells must be in a different position relative to the OCT beam size when the Ascan is repeated after the interscan time. If the blood cells are in nearly the same position relative to the OCT beam, the change in OCT signal will be too small to be detected. The blood cells will move a distance given by their flow speed times the interscan time. Therefore, longer interscan times increase sensitivity to slow flows. Conversely, if the flow is fast, the blood cells move a distance larger than the OCT beam size when the A-scan is repeated. The blood cell that was in the OCT beam at the time of the initial A-scan has moved out of the OCT beam at the time the A-scan is repeated. Other blood cells may have moved into the OCT beam, but the blood cells are indistinguishable. Therefore flows which are above a fastest distinguishable flow or saturation limit will produce the same change in OCT signal and flow differences or flow impairment cannot be distinguished by OCTA. In order to distinguish differences in flows, the interscan time must be decreased, so that the A-scan is repeated before the initial blood cells move out of the OCT-beam.

The preceding paragraph and Fig. 3 develop intuition about the relationship between blood flow and the corresponding OCT signal. However the figure is simplified to facilitate explanation and there are some inaccuracies. The transverse image resolution is determined by the OCT beam size, which is much larger than a blood cell, so individual blood cells cannot be resolved. Blood cells scatter light from the incident OCT beam and there is a phenomenon known as laser speckle, where scattering from the blood cell as well as other structures combine to determine the OCT signal. If the blood cells move a fraction of the OCT beam size during the repeated measurement, this produces a detectable change in OCT signal even though the individual blood cell are not visualized. At any given time, the OCT signal arises from a collection or ensemble of blood cells, because blood cells are smaller than the OCT beam size and axial resolution. A detailed analysis is beyond the scope of this review, however, there has been extensive previous research on OCTA and a short overview is helpful to place ophthalmic OCTA into historical context. If the interscan time is very short and the OCT beam size is small, in principle it is possible to count blood cells as they move through the OCT beam. This technique requires an OCT with a highly focused spot, small vessels and very rapid interscan times with large numbers of repeated A-scans or images. Counting blood cell transit has been demonstrated in small animal brain imaging using stereotactic immobilization with high magnification and small imaging regions (Lee et al., 2014). However, in ophthalmology, only a small number of repeated scans are possible because of bulk eye motion and transverse resolution is limited. For example, at flow speeds of 1 mm/s, erythrocytes will move 5 μm in an interscan time of 5 ms. The typical OCT beam is 20 μm and therefore can intercept multiple erythrocytes at any given time. Blood flow is not necessarily uniform in velocity, given the pulsatile nature of blood flow. As such, erythrocyte flow causes statistical changes in the OCT signal. If large numbers of repeated scans are performed rapidly and the phase as well as the amplitude of the OCT signal is measured, it is in principle possible to characterize the flux as well as diffusive motion of erythrocytes using statistical methods (Lee et al., 2012). However, the limited amount of statistical information that is obtained with only 2–4 repeated scans makes it challenging to directly relate changes in the OCT signal to erythrocyte flux. For this reason, the majority of OCTA methods simply detect the presence or absence of flow above a sensitivity threshold and the fastest distinguishable flow is also limited. OCTA characterizes the size and geometry of microvasculature based on a nearly binary representation of flow information, rather than quantifying blood flow.

### 4.5. Motion contrast algorithms in OCTA

There are several possible methods or algorithms for using repeated B-scan OCT images to calculate motion contrast. In the absence of multiple scattering, Doppler detects motion along the OCT beam direction by measuring phase changes between repeated A-scans (Chen et al., 1997; Choi et al., 2013b; Leitgeb et al., 2003b; Wang et al., 2007b). However, flow in the retinal microvasculature for the most part is perpendicular to the OCT beam. OCTA algorithms can use the OCT signal amplitude, the OCT signal phase or both amplitude and phase (known as complex amplitude) to detect motion perpendicular to the direction of the OCT beam. The most widely used OCTA algorithms use the OCT signal amplitude. The SSADA algorithm divides the OCT signal into multiple lower axial resolution images, splitting the OCT signal spectrum to reduce effects of axial eye motion, then combining data to improve signal to noise (Jia et al., 2012b). SSADA was the first algorithm to be extensively used in a commercially available ophthalmic OCTA instrument. Algorithms which use both amplitude and phase of the OCT signal spectrum can potentially be more sensitive to small changes than those which use amplitude alone, but there is a trade-off in noise and artifacts arising from eye motion. There are many possible algorithms which can be used to generate motion contrast signals for OCTA. Differences in motion contrast algorithms, combined with differences in thresholding, display and other image processing methods will cause differences in the sensitivity and saturation characteristics of the OCTA signal vs blood flow as well as differences in the appearance of microvasculature across different instruments.

### 4.6. The relationship OCTA signal and flow

**Fig. 4.**
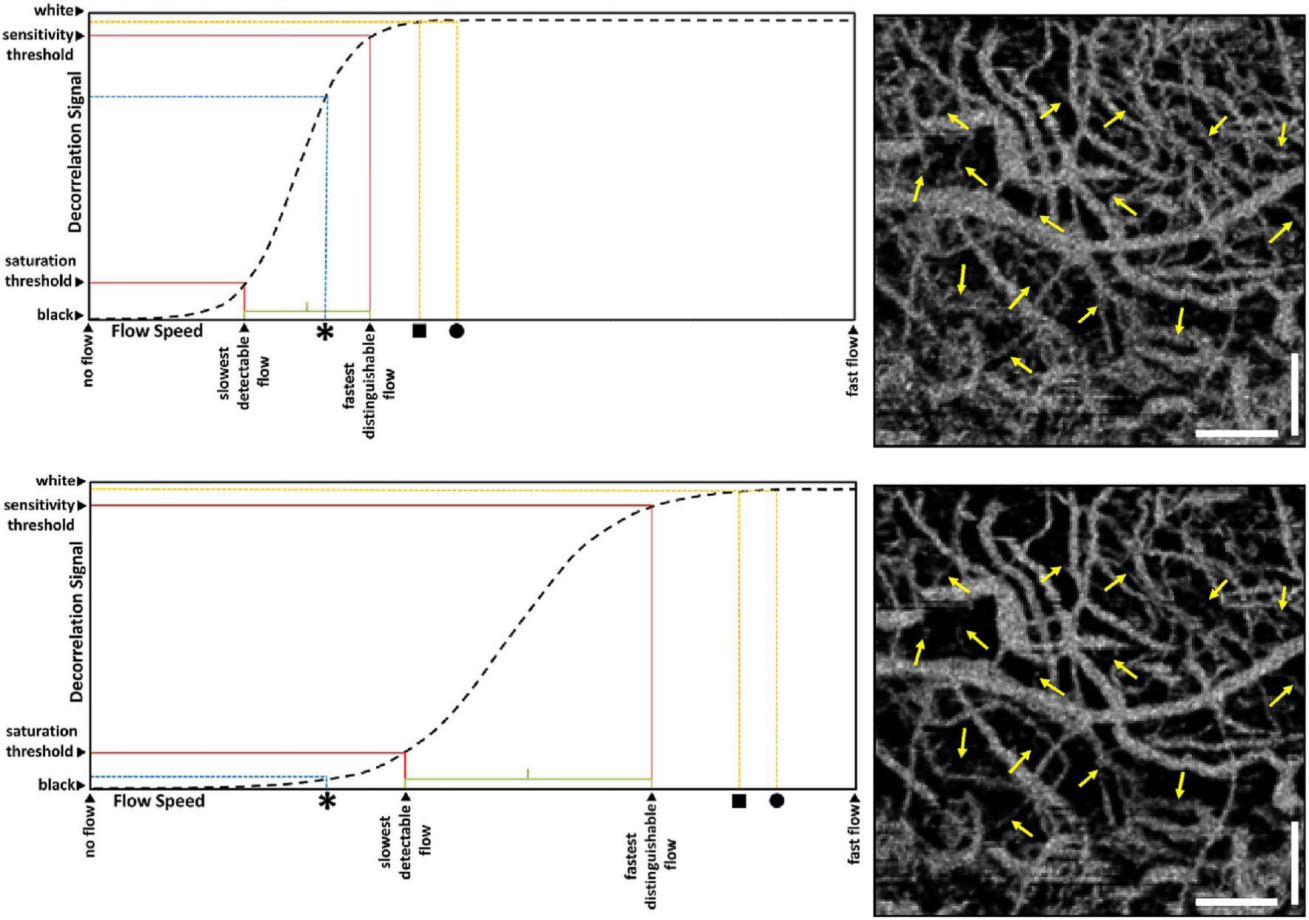
Sigmodal relationship between flow and OCTA signal. Motion contrast algorithms for OCTA typically have a sigmoidal relationship between flow and OCTA signal. Decorrelation signal (vertical axis) versus flow speed (horizontal) is shown for long (top) and short (bottom) interscan times. Current commercial instruments use long interscan times (top) and have good sensitivity to slow flows (slowest detectable flow). However, faster flows (above the fastest distinguishable flow) have a saturated OCTA signal and cannot be differentiated. If faster interscan times are used (bottom), the sensitivity to slow flow is lost, but faster flows are no longer saturated, enabling the detection of flow impairment. OCTA using slow interscan times (3.0 ms) detect large numbers of vessels, but poorly detect differences in faster flow. OCTA using fast interscan times (1.5 ms) do not detect vessels having slower flow speeds, but detect differences in faster flow. Using variable interscan times it is possible to differentiate flow impairment. The scale bars are 500 μm and the images are enlarged from a 6 mm × 6 mm field of view (Modified from Choi, Ophthalmology 2015).

The majority of OCTA motion contrast algorithms have a sigmodal like relationship between flow and OCTA signal as shown in Fig. 4 (Choi et al., 2015, 2016; Tokayer et al., 2013). The OCTA signal is typically normalized between 0 and 1. The slowest detectable flow is determined by the interscan time. An erythrocyte must move a sufficient distance within the interscan time ΔT between repeated measurements to cause the OCT signal to change. In addition, any difference in signals from one image to the next must be greater than the background noise level in order to detect the erythrocyte motion. Typically, erythrocyte motion which is even a fraction of the OCT beam spot size can be detected. This sets a *sensitivity limit*, below which slower flows cannot be detected. At the same time, the OCTA signal has a limited dynamic range over which it can discern flows. Flows which are faster than a *fastest distinguishable flow* will have the same OCTA signal (Choi et al., 2015). The signal is said to be saturated because faster flows will not increase the signal. Fast flows are visible on OCTA, but differences in flow cannot be distinguished because they are above the *saturation limit*.

The different regions in the flow vs OCTA signal curve of Fig. 4 correspond to the different regimes shown schematically in Fig. 3. When interscan times ΔT are long, as in current commercial instruments, the sensitivity is good and the slowest detectable flow is small, however, the ability to distinguish differences in faster flows is compromised. Using prototype or future instruments with higher A-scan rates, or by acquiring B-scans with fewer A-scans, it is possible to decrease the interscan time ΔT. This scales the relationship between flow and OCTA signal (Fig. 4). The ability to distinguish faster flows is improved, but the sensitivity to slower flows is compromised. Fig. 4 shows examples the VISTA technique where OCTA images are generated using variable interscan times ΔT of 3 ms and 1.5 ms. The OCTA generated using 3 ms interscan time (top) is sensitive to slow flows, visualizing more of the capillary structure, but differences in flow cannot be distinguished, while the OCTA using 1.5 ms interscan time (bottom) does not detect vessels with slower flows and can therefore distinguish these flow differences. The combination of these two images using VISTA can be used to assess which vessels have slower vs faster flows (Choi et al., 2015; Moult et al., 2014).

**Fig. 5.**
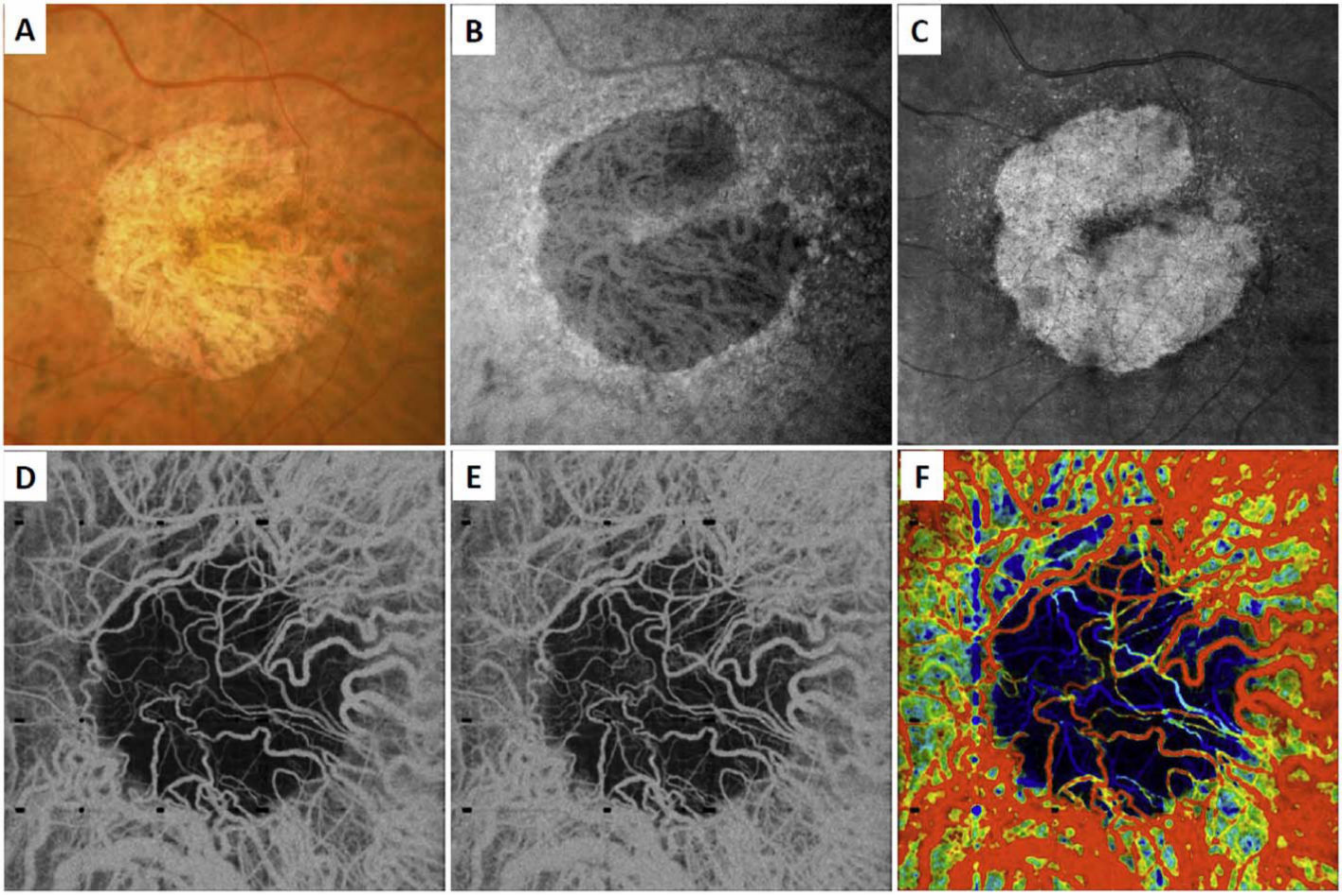
Variable interscan time analysis (VISTA) visualization. Example of VISTA visualization of choriocapillaris flow impairment in a 75-year-old GA patient. SS-OCT and OCTA acquired with 400,000 A-scans per second with 6 mm × 6 mm field of view. (A) Fundus photo cropped to OCT field of view. (B) Fundus autofluorescence. (C) OCT en face image, mean projection. (D) OCTA en face image with 1.5 ms interscan time, mean projection through ∼90 μm slab below Bruch’s membrane. (E) OCTA with 3.0 ms interscan time, mean projection through ∼90 μm slab below Bruch’s membrane. Comparing the 3.0 ms–1.5 ms interscan time images shows regions of choriocapillaris flow impairment. (F) VISTA image with red vs blue false color indicate faster vs slow erythrocyte speed. The left margin of the GA exhibits a region of flow impairment (From Ploner et al. Retina, 2016).

Fig. 5 shows an example of how VISTA can be used to assess flow impairment. The top row shows a fundus image, fundus auto-fluorescence (AF) and an en face OCT of a 75 year old patient with GA. The bottom row shows en face OCTA images of the choriocapillaris using interscan times ΔT of 1.5 ms and 3 ms. The short interscan time OCTA image shows reduced signal from the GA region as well as from the choriocapillaris on the periphery of the GA, while the longer interscan time OCTA shows additional vessels in the GA region and higher choriocapillaris signal peripheral to the GA. Longer interscan times are sensitive to slow flows but poorly distinguish flow differences. The information in the two images can be combined into a single image for ease of visualization, where relative differences in flow are mapped to a false color scale with red and blue representing fast vs slow flows respectively. The VISTA technique using false color visualization facilitates rapid interpretation and differentiates regions of flow impairment (Ploner et al., 2016).

### 4.7. Threshold masking to remove noise and attenuation artifacts

OCTA computations of motion contrast essentially measure pixel to pixel fluctuations between repeated OCT B-scans. Although the objective is to detect blood flow, many OCTA motion contrast algorithms will also detect fluctuations from noise in the OCT signals. Some algorithms use techniques to suppress noise fluctuations (Makita et al., 2016), however in general OCTA requires careful instrument design and signal processing to reduce noise sources. Even if OCT instruments have high sensitivity, all OCT images have regions such as the vitreous and choroid which have low signal and consist mainly of noise. This noise can generate false flow OCTA signals (Cole et al., 2017), and methods aimed at removing these false OCTA signals can themselves generate artifacts where OCTA signals are absent even in the presence of flow.

In order to remove pixels in OCTA volumes where there is insufficient OCT signal to obtain a valid OCTA measurement and the OCTA is produced by noise rather than real blood flow, a *threshold masking* or *thresholding* operation is performed before OCTA data is displayed. OCT signals should be above a certain threshold in order to obtain valid OCTA measurements which correspond to flow and regions which have low OCT signals, where the OCTA would be generated by noise, are thresholded and displayed as black. The OCT signal threshold is often an arbitrary level determined empirically. It is possible that some areas of slow flow will be incorrectly displayed as black on OCTA or conversely, areas of OCT noise displayed as false flow on OCTA. Fig. 2 shows how *threshold masking* is used in OCT and OCTA signal processing. The OCT signal is measured on a pixel by pixel basis in the repeated B-scans. If the OCT signal is below an empirical threshold, the corresponding OCTA pixels are assumed to be invalid and are masked (displayed as black). Valid OCTA information can only be obtained from regions which have sufficiently strong OCT signals. If OCT signals are weak, it is impossible to obtain valid OCTA information. This is referred to as an attenuation artifact. Structures which attenuate the OCT signal will cause the OCTA to be masked (because it is impossible to obtain an OCTA measurement) even if there is real blood flow present.

**Fig. 6.**
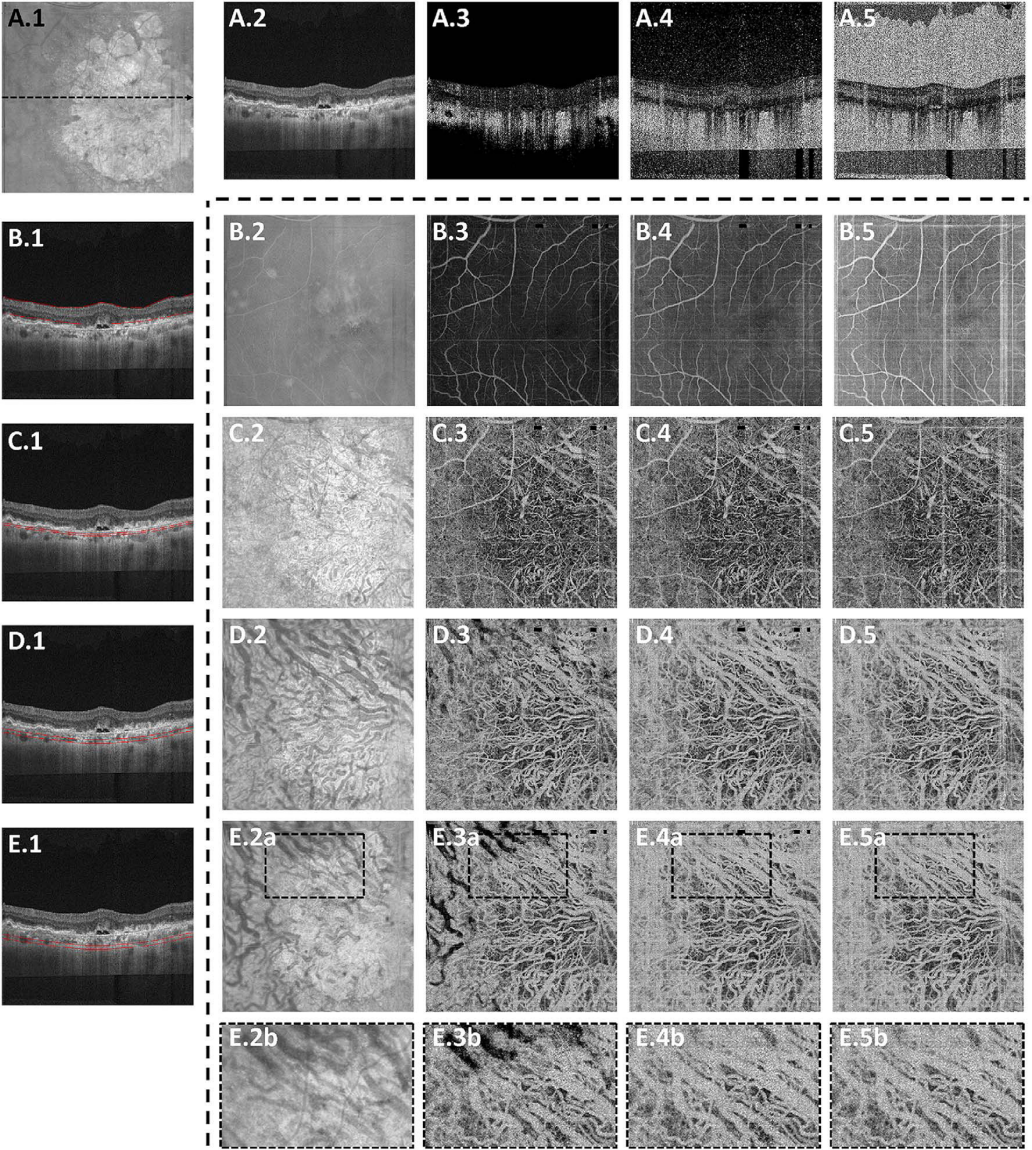
OCTA attenuation artifacts from threshold masking. Valid OCTA information can only be obtained from regions with high OCT signal. Example data from a 78-year-old male GA patient. (A.1) En face OCT, mean projection of the entire retina. (A.2) OCT B-scan at position of dashed black line in en face OCT. (A.3) OCTA thresholded at mean + 2x standard deviation (SD) of the noise in OCT. (A.4) OCTA thresholded at mean + 6x SD of the noise. (A.5) OCTA without thresholding. Rows B-E, 2–5 show en face OCTA images summed between the red contours shown on the OCT B-scan (left column). The columns 2–5 show en face OCTA thresholded at mean + 2x SD, mean + 6x SD, and unthresholded. (1–5b) are enlargements of the dashed white boxes shown in (B–E). OCTA with low threshold has masked regions where the OCT signal is low. Vitreous and sclera appear dark (A.3) and regions within the choriocapillaris appear dark (E.3a and E.3b). OCTA with high thresholds or unthresholded appears bright within the vitreous and sclera, extending below the sclera (A.5). These regions do not have flow, but OCTA decorrelations are generated by noise in the OCT image.

Fig. 6 shows how threshold levels can affect OCTA display (adapted from Cole et al., 2017). The top row (A.2 to A5) shows a representative OCT cross-section (A.1) and OCTAs with different threshold levels. En face OCTA projections of retinal layers (indicated in the left column (B.1 to E1) are shown with different thresholds (rows B.2 to B.5 through E.2 to E.5)). If the OCTA signal is displayed without threshold masking (column 5), there are high OCTA signals in the vitreous and choroid as well as below the sclera, even though there is no blood flow in these regions. This false flow artifact is produced by noise fluctuations in the OCT signal which are indistinguishable from fluctuations caused by blood flow. Setting progressively higher threshold levels (columns 3 and 4), removes OCTA artifacts from low signal regions, however, higher thresholds tend to remove OCTA signals from regions of low OCT signal, such as below the RPE. The OCTA information is invalid and is masked out because the OCT signal is attenuated.

OCT signals from choroidal vessels can be attenuated because the vessels are deeper and have low signal, largely secondary to attenuation from optical scattering by overlying tissue. The OCT signal can also be lost if a vessel has high blood flow resulting in a “fringe averaging” effect in the OCT interferometric detection resulting in low or absent OCT signal (Hendargo et al., 2011; Yun et al., 2004). Because the inner portions of large vessels show little or no reflectivity or backscattering in OCT images, a valid OCTA cannot be obtained and the image appears dark even if there is flow (E2b vs E3b). Conversely, smaller choroidal vessels have greater OCT reflectivity or backscatting from within vessels and are the corresponding OCTA signal is demonstrable. Larger choroidal vessels are also deeper in the choroid, a region with poor penetration and high scattering, which reduces OCT signal levels in the first place.

This example shows that the threshold setting can have a dramatic effect on the appearance of the OCTA data. The threshold settings can vary between instrument manufacturers. Threshold masking avoids displaying false flow OCTA signals from regions where it is invalid because of low or noisy OCT. However, threshold masking can also create attenuation artifact interpretation errors. The key point for expert interpretation is to recognize that absence of OCTA signal does not necessarily imply the absence of flow and corresponding OCT images must be visualized along with OCTA in order to confirm validity.

## 5. Visualization methods for OCTA

OCTA data is inherently three dimensional and has the advantage that is it intrinsically co-registered to three-dimensional structural OCT data. The three dimensions of a volume of tissue scanned are mapped to a representation stored in the computer memory, where numerical values correspond to OCT or OCTA signals derived from the volume of tissue. There are multiple approaches for visualizing this data.

### 5.1. En face OCTA and projection

The retina and choroid naturally are arranged in layers. The layers of the retina can be detected and demarcated by image processing methods known as segmentation, and the corresponding flow information can be shown for these individual layers. The OCTA volume containing flow data is segmented based on the tissue architectural morphology in the structural OCT. This separates layers of flow information that in turn are used to create images to display on a computer monitor. A common method is to poll columns of voxels and display the brightest voxel. This process projects the maximal intensity from each column onto a flat two-dimensional image, in a process called maximal intensity projection. The advantage of a maximal intensity projection is that it can show the flow in small vessels; the disadvantage is that it is sensitive to noise, since statistical outliers would be shown. There are other ways to select the value to display such as average or mean intensity projection, which displays the average brightness in a column of voxels and is less sensitive to noise, but does not show small vessels well. That is because the contribution to the total column made by flow in a small vessel may be only one voxel in a column of many. It is also possible to generate a projection based on the histogram distribution of values found in the column of voxels, in an attempt to balance noise versus showing flow in small vessels.

**Fig. 7.**
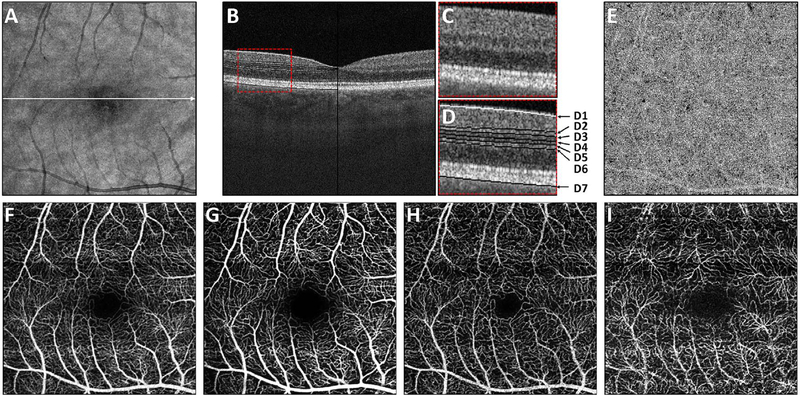
How retinal capillary plexus and choriocapillaris images are generated. Vascular layers can be independently displayed by segmenting retinal architecture in OCT structural images and projecting the OCTA over axial depth ranges in order to generate en face images. (A) En face OCT image. (B) OCT B-scan through the macula with enlargement (C) showing retinal layers. (D) Retinal layers showing segmentation contours; D1 indicates the ILM; D2 indicates contour C3 offset by half the INL width; D3 indicates the IPL-INL boundary; D4 indicates the midline between contours D3 and D5; D5 indicates the INL-OPL boundary; D6 indicates contour D5 offset by half the INL width; contour D7 indicates the Bruch’s membrane. (E) En face OCTA of the choriocapillaris was generated by projecting a thin slab below Bruch’s membrane D7. (F) En face OCTA of the total retinal vasculature, projecting from the ILM to the RPE (contour not shown). En face OCTA images of different capillary plexuses generated by summing over depth ranges. OCTA of the surficial (G), intermediate (H) and deep plexus (I) were generated by projecting between contours D1 to D2, D2 to D4, and D4 to D6 respectively. (Adapted from Choi et al. Retina, 2017).

Using this strategy, the three dimensional OCTA flow data in any given layer are summed or projected into a two-dimensional en face image that can be displayed or printed. Note that there may be more than one blood vessel layer present, but the representation shows the brightest voxel, no matter where it originates from within the segmented volume. This same flattening effect occurs with any means of selecting a value from a column of voxels such as average intensity projection. When using en face imaging, the depth information within the segmented volume is lost. However, en face imaging is powerful because of the underlying structure of the retina and provides simple representations of flow. Essentially no user input is required to obtain the images once the OCT and OCTA information is obtained, provided that the segmentation is correct, which unfortunately is often not the case in disease. Fig. 7 shows an example of how retinal capillary plexus and choriocapillaris images are generated. Although this example shows three capillary plexuses, most commercial instruments use a simpler segmentation protocol to visualize two capillary plexuses. The risk of errors increases if additional layers are segmented. In addition, it is important to note that en face OCTA images of the capillary plexuses are not always generated by projecting specific retinal layers, but rather by offsetting the projection ranges relative to the retinal layers to encompass the expected depth ranges of capillary plexuses. If these layers differ from the expected thicknesses, by normal variation or by disease, the segmentation may not be accurate. En face OCTA images do not necessary correspond to the standard definitions of retinal capillary vasculature. For example, many manufacturers incorrectly segment the vascular layers adjacent to the fovea (Spaide and Curcio, 2017). In addition to displaying the capillaries, it is also helpful to generate an en face OCTA of structures such as the outer nuclear layer which are normally avascular. This enables the presence of neovascularization, such as type 2 or Type 3 macular neovascularization to be readily visualized. En face OCTA images at different depth levels can be displayed in multiple windows or as a single image where each depth is color coded. Many manufacturers use color coding which facilities rapid reading, but the color choice is somewhat arbitrary and differs between manufacturers.

**Fig. 8.**
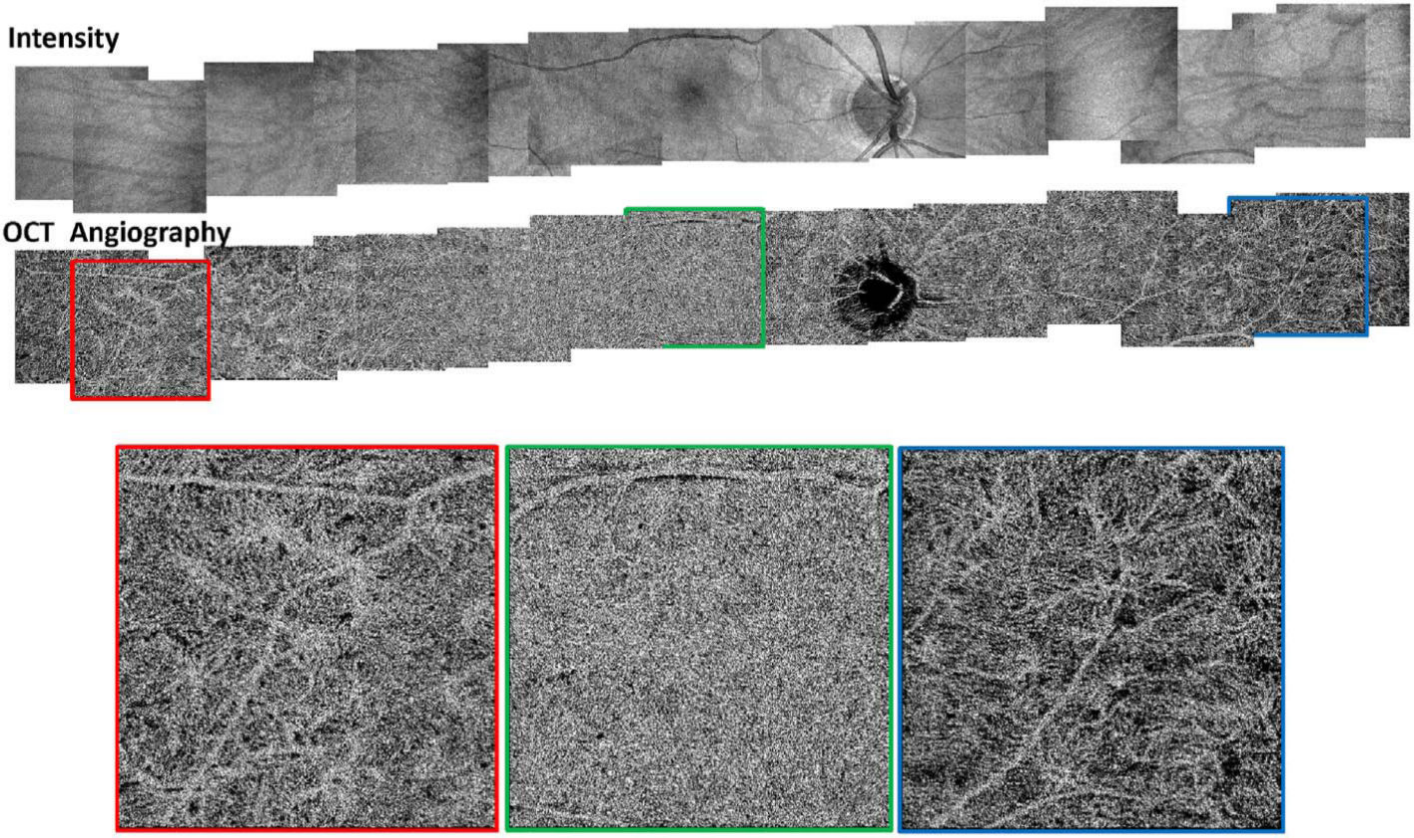
OCTA of the choriocapillaris. Mosaicked OCT and OCTA of the choriocapillaris spanning ∼32 mm across the retina. Imaging performed using an SS OCT with 400,000 A-scans per second. Four repeated horizontal B-scans of 800 A-scans each were acquired over 400 vertical positions on a 3 mm × 3 mm fields. En face OCT retinal images (top row) and OCTA of choriocapillaris (bottom row). OCTA shows that choriocapillaris has densely packed honeycomb structure near macula and sparser, lobular structure towards the periphery. (Adapted from Choi et al., 2013a).

It is also possible to visualize vasculature below the retina, such as the choriocapillaris. Fig. 8 shows an example of the choriocapillaris (Braaf et al., 2012; Choi et al., 2013a). En face OCTA images were generated by segmenting the RPE plus Bruch’s membrane position and projecting a several micrometers deep range below the RPE. Images further from the macula exhibit a lobular pattern which is characteristic of the choriocapillaris architecture. The challenge is that the choriocapillaris is extremely thin and possible errors in segmentation can result in the segmented region missing the choriocapillaris. At the same time, blood flow in the choriocapillaris produces an OCTA signal that can affect structures below it through projection artifact which can be used to assess the choriocapillaris.

### 5.2. En face vs B-scan OCTA images

**Fig. 9.**
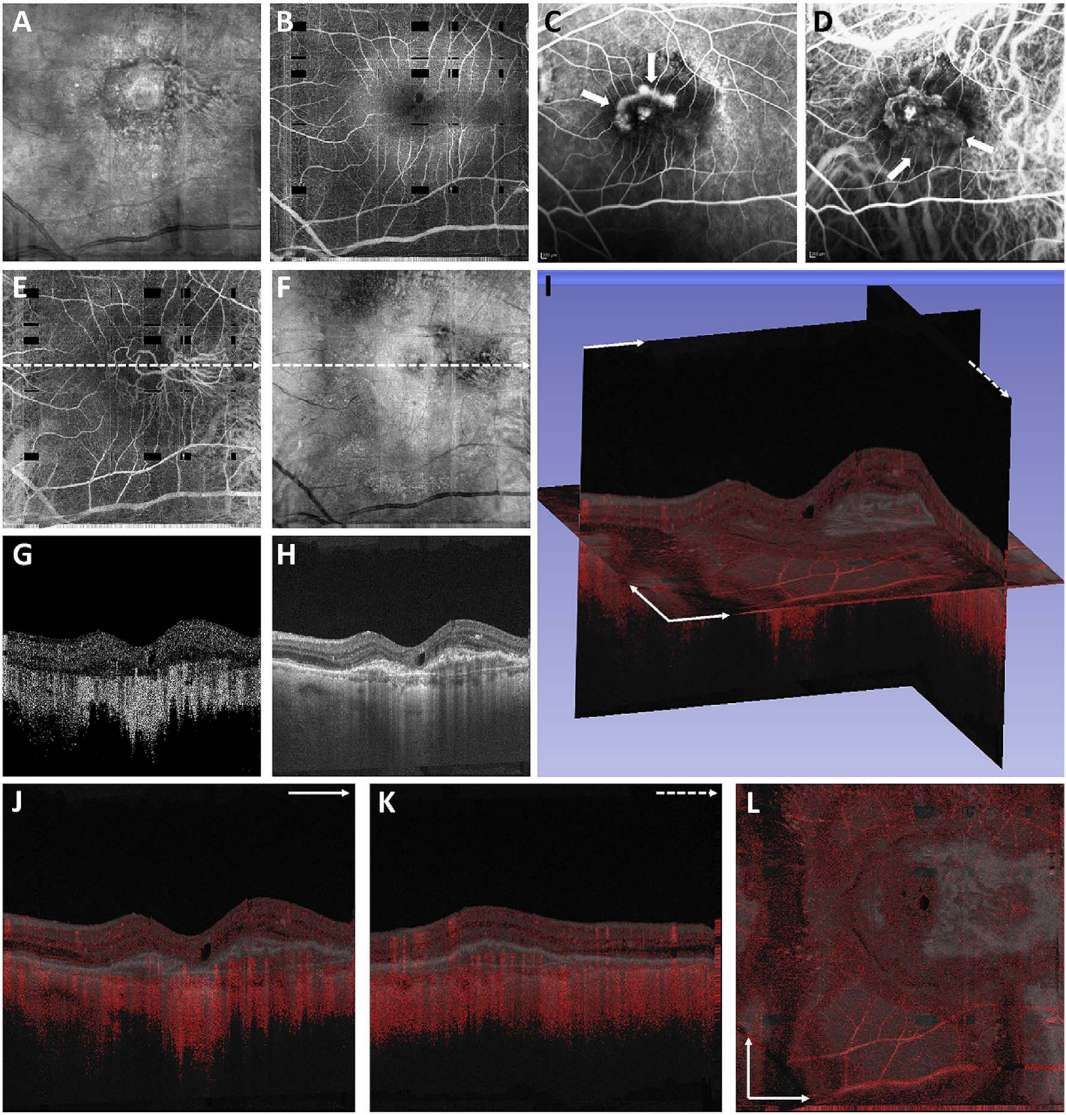
En face and B-scan visualization of OCTA. OCT, OCTA, and FA, and ICGA images from an 87-year-old with a CNV lesion exhibiting both occult and classic component. (A) En face OCT projection through the entire volume. (B) En face OCTA projection over the retinal vasculature. The dark rectangles are missing data from saccadic eye motion. (C) Early-phase FA. Arrows point to the classic component of the lesion. (D) Early-phase ICGA. Arrows point to the occult component of the lesion. (E) En face OCTA projection through the depths spanned by the lesion. (F) Corresponding en face OCT projection. (G–H) OCT and OCTA B-scans at position of the dashed arrows in E, and F, respectively. (I) Orthoplane visualization, where the OCTA is overlayed red on the OCT. (J) B-scan at the plane indicated with the solid arrow in (I). (K) B-scan at the plane indicated by the dashed arrow in the (I). (L) En face image at the plane indicated by the intersecting arrows in (L). Orthoplane visualization can reduce the risk of misinterpretation.

To date most OCTA literature has emphasized using en face OCTA viewing because of its natural correspondence with fundus imaging. However, en face OCTA is extremely susceptible to segmentation errors in the retinal layers, especially in eyes with pathology. Retinal segmentation strategies are inherently based on the anatomy of the normal retina. Normal retinas have well defined variations in architecture, while a retina with pathology can be abnormal in a myriad of ways. Given the complexity of pathologies such as edema, cyst formation, subretinal fluid, pigment epithelial detachment, neovascularization and geographic atrophy, it is currently virtually impossible for a computer algorithm to accurately identify retinal layers in the presence of all types of pathology. For this reason, viewing cross-sectional OCT scans with an OCTA flow overlay is very helpful. These OCTA B-scans typically show flow in color over a grayscale OCT image. Cross sectional viewing using B-scans can help obviate dependence on en face imaging with inherent segmentation errors and also be used to evaluate for the potential for projection artifacts. Fig. 9 shows an example of OCT and OCTA en face vs B-scan visualization of macular neovascularization (MNV) lesions with both classic and occult components. En face OCT (A) and OCTA images with a full projection (B) vs projection through the depths spanned by the lesion (E) are shown along with a cross sectional OCTA (G) and OCT (H). FA shows the classic component of the lesion (C) and ICGA the occult component (D). Panels I though L show the OCTA signal overlaid on the OCT structural image in orthoplane view (I), cross sections (J, K) and en face (L). Cross sectional OCTA images enable the location of vascular pathology to be assessed without possible segmentation errors which can cause artifacts in en face OCTA images. This approach is especially powerful because it is possible to display structural OCT cross sectional images which are intrinsically co-registered with the OCTA cross sections. The advantage of examining cross sectional images is the precise visualization of flow information versus depth in the tissue. The disadvantages are the need to scroll through numerous images and the absence of an image showing the entire extent of neovascularization in an en face sense. At the same time, if there are questionable findings in en face OCTA images, it is important to review cross sectional OCT and OCTA images in order to check for possible segmentation errors, projection artifacts or attenuation artifacts.

**Fig. 10.**
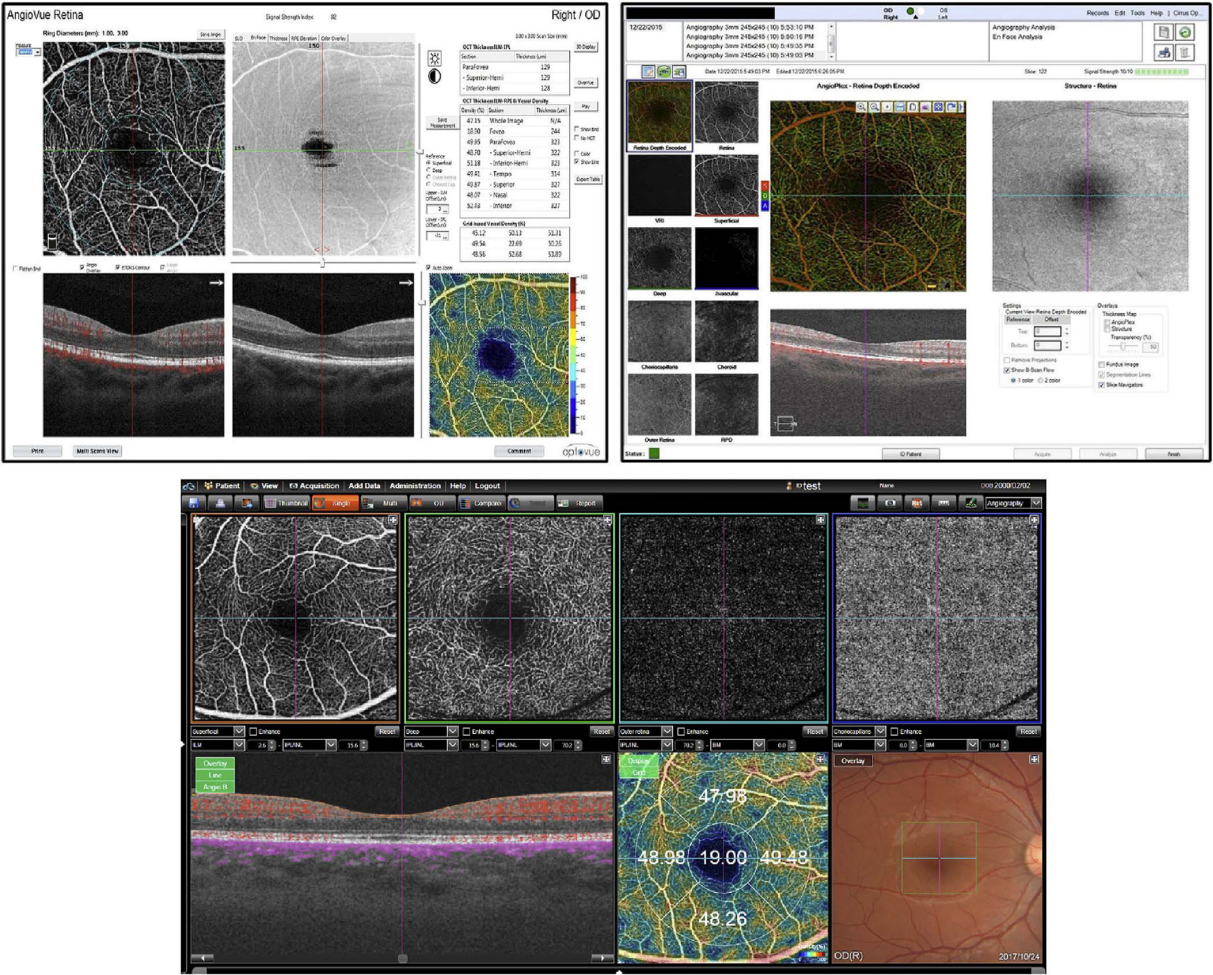
Graphic user interfaces in commercial instruments. Representative screenshots from commercial OCTA instruments, including the Optovue (top left), Zeiss (top right) and Topcon (bottom) OCTA displays. Commercial displays and user interfaces enable segmentation of retinal layers as well as simultaneous viewing of OCT and OCTA data. Many commercial instruments use false coloring to differentiate depths of vasculature or different retinal capillary plexuses. At the time of this writing, commercial software is still rapidly evolving. The challenge will be to achieve consistent notation and quantitative measurements between instruments from different manufacturers.

Commercial OCT instruments have implemented different methods for visualizing OCTA and OCT data. Fig. 10 shows examples of the graphic user interfaces for several instruments. Multiple pane displays typically show en face OCTA projections of different retinal layers as well as registered cross sectional OCT images. En face OCT projections are sometimes color coded according to layer or depth and overlaid to facilitate rapid interpretation. Cross sectional OCTA images are less frequently displayed, but are gaining popularity. Segmentation errors, which can cause errors in en face OCTA, remain a challenging problem in situations where pathology distorts the normal retinal architecture.

### 5.3. Volume rendering

The weaknesses of en face imaging include the flattening of data in any given segmented volume and segmentation errors from pathological tissue in the first place. En face imaging not only flattens the volume within the region imaged, it also merges flow information between vessels. There are three important consequences of this. First, tissue perfusion can be underestimated since superjacent or subjacent vessels may not be imaged. Second if projection is used, (that is if the data in the slab is shown as a single pixel layer) vessels separated in space but overlapping in along the projected direction can appear to be merged. Thus two vessels overlying each other that diverge can appear identical to one vessel that branches. Third, blood vessels that are not related can appear to anastomose. Thus using en face imaging with projection, it may not be possible to adequately model blood flow into and out of a volume of tissue. Abnormalities of segmentation can lead to either lack of visualization of true flow or incorrect attribution of flow to a specific layer within the retina.

**Fig. 11.**
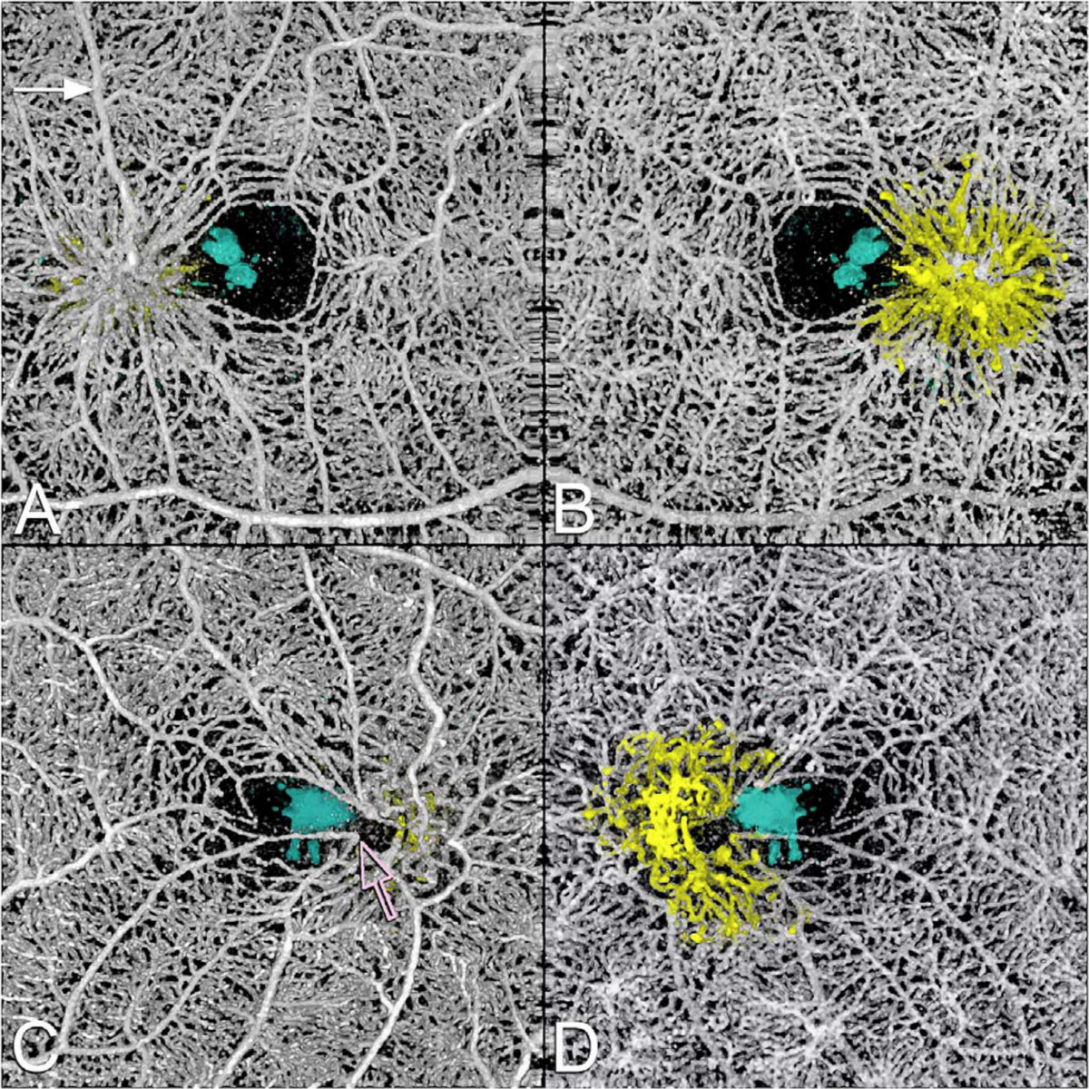
This 64 year-old male with MacTel2 had a visual acuity of 20/30 in each eye and was imaged with volume rendered OCTA with segmentation of the retinal cavitations from the structural OCT data. There are prominent right-angle veins in each eye. (A) The exit point of the right-angle vein from the substance of the retina in the right eye is at the nexus of a network of vessels, which appear to be drawn into a central focus. Retinal arterioles, venules, and small order vessels appear to be involved. The foveal avascular zone is distorted and appears to be pulled toward the temporal macula. There is a group of neighboring (and in the image, overlapping) foveal cavitations (cyan) that are on the temporal side of the foveal avascular zone. (B) Viewed from the choroidal side, the deeper penetrating vessels appear as yellow. This region corresponded to the area of late fluorescein staining. (C) More prominent traction on the vessels are evident in the left eye with pulling of the perifoveal vessels into an apex of a triangle (open arrow). (D) Viewed from the choroidal side the vessels deep to the deep vascular plexus are shown in yellow. The cystoid space in the left eye (cyan) has a complex outer boundary (From Spaide et al. Retina, 2017).

**Fig. 12.**
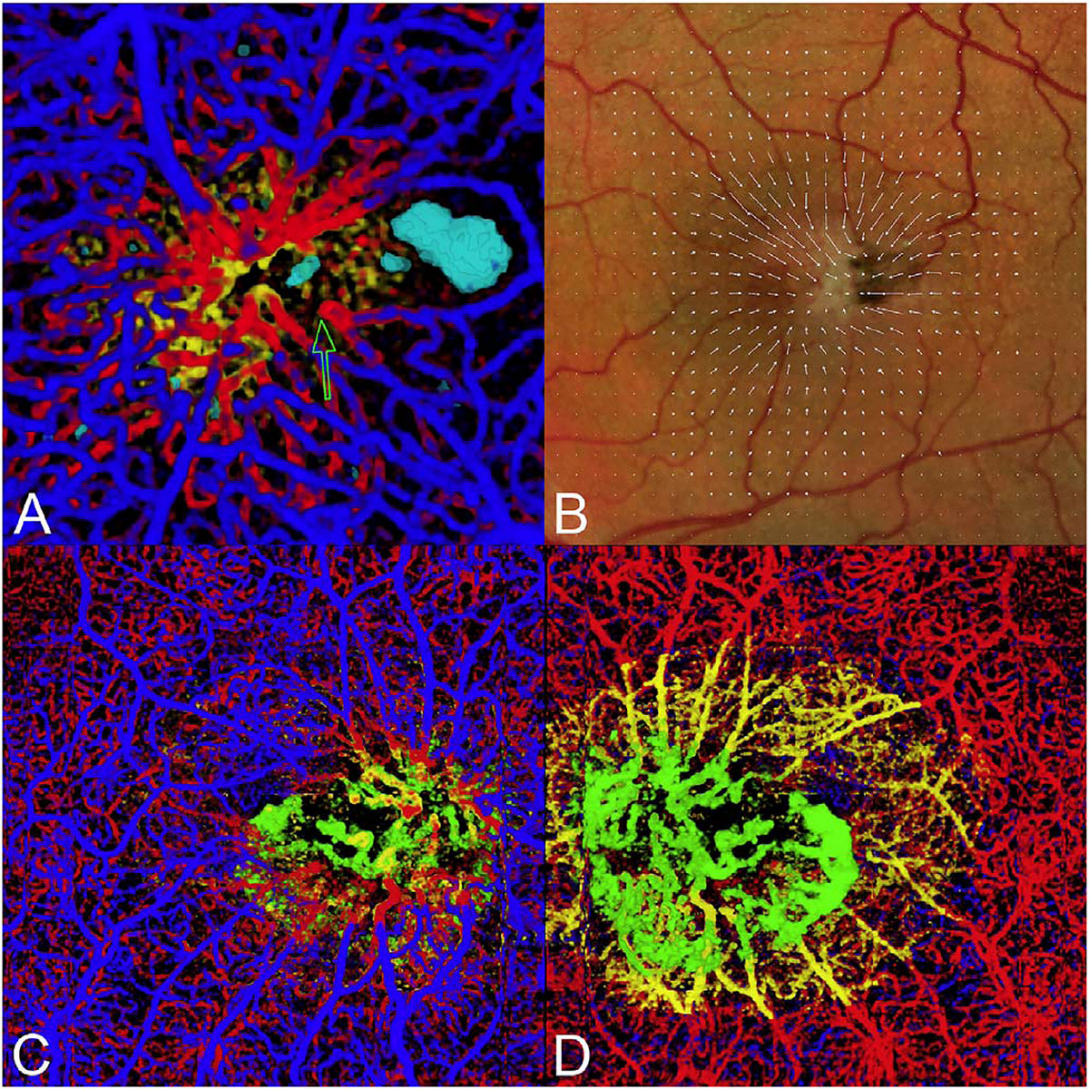
(A) Mactel2 in a 58-year-old with right-angle veins and cavitations imaged with volume rendered OCTA with segmentation of the retinal cavitations from the structural OCT data. The foveal avascular zone is smaller and displaced toward the central focus of the vascular lesion in the temporal macula. Note the vessels of the perifoveal ring are drawn toward the center of the vascular aggregate and for angular figures and their apices (one of which is shown by the open arrow) point toward the center of the vascular aggregate (Modified from Spaide et al. Retina, 2017). (B) A vector field map showing the retinal displacement over a 10 year period. The tissue displacement reveals an epicenter in the temporal juxtafoveal macula. (C) A color-coded volume rendering with the superficial vessels shown in blue, the deep plexus red, the vessels deeper than the deep plexus (as occurs in MacTel2), yellow and vessels below the level of the RPE, green. (D) When viewed from the choroid, the proliferating vessels under the RPE have an enlarged saccular nature, which is different from the vessels in the overlying layers.

One method to avoid both problems is to use volume rendering of the underlying data. Volume rendering allows visualization of all layers of flow in the retina and also permits manipulation of the data in 3 axes of rotation. Volume rendering does not depend on layer segmentation. Retinal structures such as cystoid spaces or tumefactions can be segmented from the structural OCT and integrated with the volume rendered OCTA data, since both are ultimately derived from the structural OCT (Fig. 11). This imaging modality has proven useful in evaluating cystoid macular edema due to retinal vascular causes and also macular telangiectasis type 2 (Spaide et al., 2017). Examining the relationships between abnormalities in the retina, such as cystoid spaces and vascular anatomy is relatively easy with volume rendering, whereas the same assessment would be very difficult with en face imaging. A hybrid approach can be taken in which layers are color-coded for easier interpretation of the images (Fig. 12). Volume rendering has not been implemented yet in most commercial instruments.

## 6. The role of instrumentation in OCTA

Since OCTA requires re-scanning the same retinal area, there are tradeoffs between field of view, axial scan density and acquisition time. For structural OCT images, a column of voxels is captured in one Ascan. For OCTA images, multiple repeated axial scans are needed to detect motion. Therefore, OCTA requires high axial scan rates or increased imaging times. The development of Fourier domain detection enabled a ~20 dB increase in sensitivity because signals from the full depth of the tissue are detected at once. This can be translated into a 50 to 100 times increase in imaging speeds as compared with early time domain detection (Choma et al., 2003; de Boer et al., 2003; Leitgeb et al., 2003a). These high speeds were critical for OCTA because of the high data requirements which increase in proportion to area as wider fields of view on the retina are imaged.

### 6.1. Spectral domain and swept source detection

Spectral domain OCT (SD-OCT) uses a spectrometer and a line scan camera for detection. The limited spectrometer resolution causes the detection sensitivity to vary for OCT signals at different axial ranges, a phenomenon known as sensitivity roll off. Axial scan rates are determined by the camera reading rate. Speeds of 300,000 axial scans per second have been demonstrated in research systems (Potsaid et al., 2008), however, commercial OCT instruments typically operate at 70,000 axial scans per second. Swept source OCT (SS-OCT) uses a frequency swept laser and a high-speed detector, without requiring a spectrometer. The sensitivity roll off is much less in SS-OCT compared with SD-OCT because frequency swept lasers can have narrow frequency linewidths. Although the detection system for SS-OCT is less expensive than the spectrometer and line scan camera used for SD-OCT, laser light sources used in SS-OCT are currently expensive, making SSOCT costlier. SS-OCT using commercially available lasers (short cavity lasers) has been demonstrated at 100,000 axial scans per second several years ago (Potsaid et al., 2010). The next generation SS-OCT systems based on vertical cavity surface emitting lasers (VCSELs) can achieve 400,000 to 500,000 axial scans per second (Grulkowski et al., 2012). Akinetic lasers capable of 400,000 axial scans per second through software control are entering the market and may be useful for future instrumentation (Chen et al., 2016b; Xu et al., 2017). Akinetic lasers have high sweep linearity, extremely low phase jitter, and a very narrow linewidth. Record imaging speeds of more than 1,000,000 axial scans per second have been demonstrated using Fourier domain modelocked laser technologies (Klein et al., 2011; Mohler et al., 2015). Safe ocular exposure limits vary with wavelength and longer wavelengths have higher permissible exposure (typically measured at the cornea). At the same time, increases in imaging speed will decrease the OCT signal and speed is ultimately limited by allowable light exposure and signal to noise requirements. Very high scan speeds can be obtained by sacrificing signal and averaging multiple images. However since OCTA requires OCT images with high signal to noise ratio, there will be an upper limit to the speed.

### 6.2. Increased image penetration with long wavelength OCT

Historically, OCT imaging was performed at 840 nm because superluminescent diode light sources used for laser gyroscopes were at these wavelengths. In SD-OCT, 840 nm also had the advantage that it was detectable using silicon based CCD or CMOS camera technology. The wavelength ranges which can be used for retinal imaging are constrained by water absorption in the vitreous. Longer wavelength light at 1050 nm has been shown to have reduced attenuation from ocular opacities and improved penetration into the choroid (Povazay et al., 2003, 2007; Unterhuber et al., 2005). This wavelength was chosen because it is in a transmission window of the eye, as chiefly governed by water absorption, between a 950 nm absorption peak and rapidly increasing absorption at 1100 nm. Thus, the usable bandwidth of a 1050 nm light source may be limited, or at least affected by water absorption as bounded between these two peaks. Early studies used time domain detection, however SD-OCT at 47,000 axial scans per second was demonstrated a decade ago using InGaAs camera technology which was sensitive to longer wavelengths (Povazay et al., 2007; Puvanathasan et al., 2008). SS-OCT can achieve higher speeds as well as operate at 1050 nm wavelengths. Although higher light exposure is permissible at 1050 nm as compared with 840 nm, there is also greater absorption of longer wavelength light by water. There is still a net benefit, particularly when imaging the choroid because of decreased scattering by pigment in the RPE and the choriocapillaris.

**Fig. 13.**
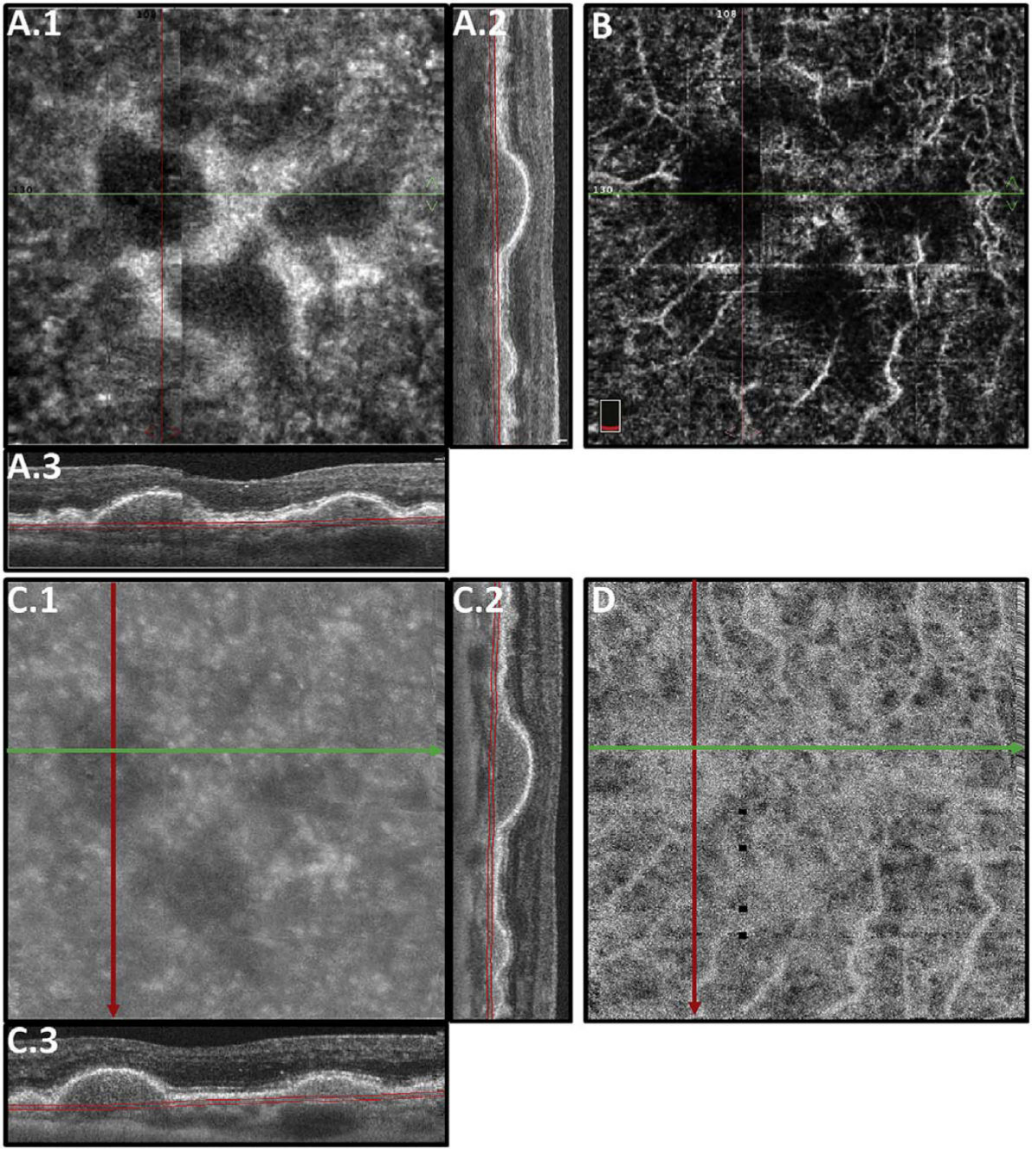
The effects of imaging wavelength on image penetration and attenuation artifacts. (A) OCT images from a spectral domain SD-OCT instrument operating at 840 nm wavelength compared with (C) images form a swept source SS-OCT instrument at 1050 nm. The en face OCT images (A.1 and C.1) are from the depth ranges shown in the respective cross-sectional images (A.2, A.3 and C.2, C3). B-scans at 840 nm (A.2 and A.3) exhibit more attention below the retina and drusen than B-scans at 1050 nm (C.2 and C.3). OCTA images at 840 nm wavelength and 1050 nm are shown in (B) and (D) respectively. The OCTA images represent the choriocapillaris and are from depth ranges shown in the respective cross-sectional images (A.2, A.3 and C.2, C.3). Drusen produce OCT signal attention for structures below them. The attenuation is appreciable for 840 nm as seen in the en face (A.1) and cross-sectional images (A.2, A.3) and corresponding regions of the OCTA (C) do not have valid OCTA signal. Attenuation is less at 1050 nm as seen in the en face (C.1) and cross-sectional images (C.2, C.3) and corresponding regions of the OCTA (D) show the presence of signal. Projection artifacts from retinal vasculature are observed OCTA images at both wavelengths.

Fig. 13 shows an example which demonstrates differences in image depth between 840 nm and 1050 nm wavelength OCT and OCTA. Fig. 13 A-B are acquired with 840 nm show OCT and OCTA projections over the choriocapillaris depth range while Fig. 13 C-D are acquired at 1050 nm and show corresponding OCT and OCTA projections. The OCT images at 840 nm exhibit attenuation under the drusen which is evident in the en face and cross sectional images and causes a loss of OCTA signal in the en face OCTA. As note previously this has been referred to as an attenuation artifact where flow is present but not displayed in the OCTA. Conversely, the OCT images at 1050 nm have higher signal under the drusen and the corresponding en face OCTA shows that the choriocapillaris appears intact. There may be an artifactual increase in brightness in the 1050 nm images because of increased fluctuation in the OCT below the drusen, possibly caused by lipid in the drusen. The artefactual loss of the choriocapillaris signal or attenuation artifact in the 840 nm OCTA image is the result of threshold masking because of low OCT signal. This example demonstrates that 1050 nm wavelengths can have superior image penetration and less attenuation than 840 nm wavelength. It also points out that expert interpretation of OCTA images requires the examination of corresponding OCT images to confirm that sufficient OCT signal is present to obtain valid OCTA data.

## 7. Artifacts in OCTA

### 7.1. Low signal strength

Any medical image information contains information derived from the object or tissue being investigated and also contains spurious alterations in the information arising from a variety of sources. The ratio between the signal, which is the information component directly related to the tissue being imaged, and the noise or non-signal component, is a parameter which can be used to characterize the information content. For example, cataracts or media opacities decrease the signal to noise ratio because they decrease the OCT signal, but don’t directly influence the instrument noise. The grayscale range of the display device is adjusted to display the image. In this process the image will appear noisier, since the signal is a smaller proportion of the whole. Thus, while low signal is a defect and not an artifact, it is accompanied by greater noise or “snow” in an image, which is an artifact. In some situations, it is possible to detect the useful information even when the signal is weak and large amounts of noise are present. However the human visual system requires a higher signal to noise ratio, about 5 depending on the spatial frequency as defined by the Rose criterion (Burgess, 1999), in order to accurately identifying features in noisy environments. Ideally the signal to noise ratio should be as high as possible to allow easy, confident interpretations of images.

Instrument manufacturers report a signal strength score, but the derivation of how these scores are calculated is often proprietary. Lower signal strengths compound the difficulty of clearly and unequivocally being certain smaller vessels are being visualized in their entirety. Smaller vessels produce smaller signals, which may be lost in background noise. This loss may not be obvious as many instruments take steps to reduce the appearance of noise in the images they display. In reducing noise, the signal from smaller vessels may be attenuated as well, leaving no signs of their existence. The approaches to reduce noise are manufacturer specific and there is limited published information to enable understanding of what the instruments actually are doing. Diffuse media opacity, most commonly secondary to cataract, can account for low signal strength, but other factors such as dry eyes or incorrect positioning the OCT instrument relative to the eye can also decrease signal strength. Therefore reimaging the patient after ocular lubrication or optimizing the OCT imaging technique can often improve the signal strength. Some commercial OCTA instruments report what seems like spuriously high signal strength scores if the patient has numerous eye movements. Defocus can occur through various mechanisms such as operator error or astigmatism in the optical system and this can cause a general decrease in signal strength.

### 7.2. Localized loss of signal strength

Vitreous floaters, even if relatively transparent, can produce aberrations on the OCT beam and reduce the OCT signal, even though the actual attenuation of light may be small. This produces a region of the retina where the reflected or backscattered light is not well collected by the OCT interferometer. Thus, the shadowed areas are darkened to the point where flow may not be visible. Besides large floaters, wisps of vitreous hemorrhage, inflammatory cell aggregates, and potentially early amyloidosis can also cause shadowing. With discrete floaters it is often possible to either wait for the floater to leave the field of view or have the patient move their eye to shift the floater to a position not in the image area. The loss of signal can be related more to loss of spatial coherence or mode structure and not as much to light absorption. The aberration means that the reflected or backscattered OCT beam cannot be collected efficiently by the optical fiber in the OCT interferometer because it is no longer a diffraction limited focused spot. Tumefactions can cause a regional defocus with a localized loss of signal, which can attenuate the image from blood vessels in regions other than the plane of focus.

### 7.3. Movement of the eye

OCTA signals are generated by detecting motion between repeated B-scans using decorrelation or fluctuations on a pixel by pixel basis. Movement of the patient’s eye, head, or body results in widespread decorrelation over the entire B-scan. The clinically evident decorrelations due to bulk motion and not blood flow are referred to as motion artifacts. Movement is a very important source of artifacts in OCTA images. OCTA images are typically viewed en face and have much higher contrast than OCT images, therefore eye motion produces shearing distortion or gaps in en face images that are much more evident and distracting than in en face OCT images. For the sake of discussion, movement will be divided into two broad categories. The first category is caused by gross movement of the eye and the second is caused by movement within the eye.

The eye moves by action of the extraocular muscles and also from movements of the head, neck and body. This motion can be broken down into components. For small motions in the field of view of an instrument, the motion can be approximated as largely translational. Although the eye rotates about a horizontal or vertical axis through its center, the depth range of the retinal vasculature imaged is much smaller than the radius of rotation. Saccades are only one form of eye motion. Another is slow drift, in which the eye moves off of fixation. In eyes with good visual acuity, the velocity range of slow drift is substantial, and the speed and amplitude of slow drift in eyes with amblyopia or scotomas can be much greater, as much as 3° per second (Yang et al., 2003b, 2003c; Zhao et al., 2000).

#### 7.3.1. Tracking and software correction

Every manufacturer of OCT angiography instruments now has some form of active eye tracking. With eye tracking, the eye position is rapidly measured and corrective measures are taken if the motion is greater than some established threshold (Ferguson et al., 2004; Hammer et al., 2005; Vienola et al., 2012). Alternate approaches rescan portions of the fundus affected by a saccade and the rescanned section is stitched into the original scan, replacing the region affected by the eye movement. The speed of detection and the delay in modifying the OCT scan, known as latency, differs between manufacturers. These methods were first implemented commercially in the Spectralis OCT using scanning laser ophthalmoscopy and later in the Cirrus OCT using a line scan laser ophthalmoscopy. Optovue and Topcon use a fundus image from an infrared camera. Eye tracking has the important advantage that it can extend the available imaging time beyond the few seconds when patients can fixate without saccades or blinking. Eye tracking also facilitates registration of one image to another taken at a later date. Accurate comparisons over time can be performed.

**Fig. 14.**
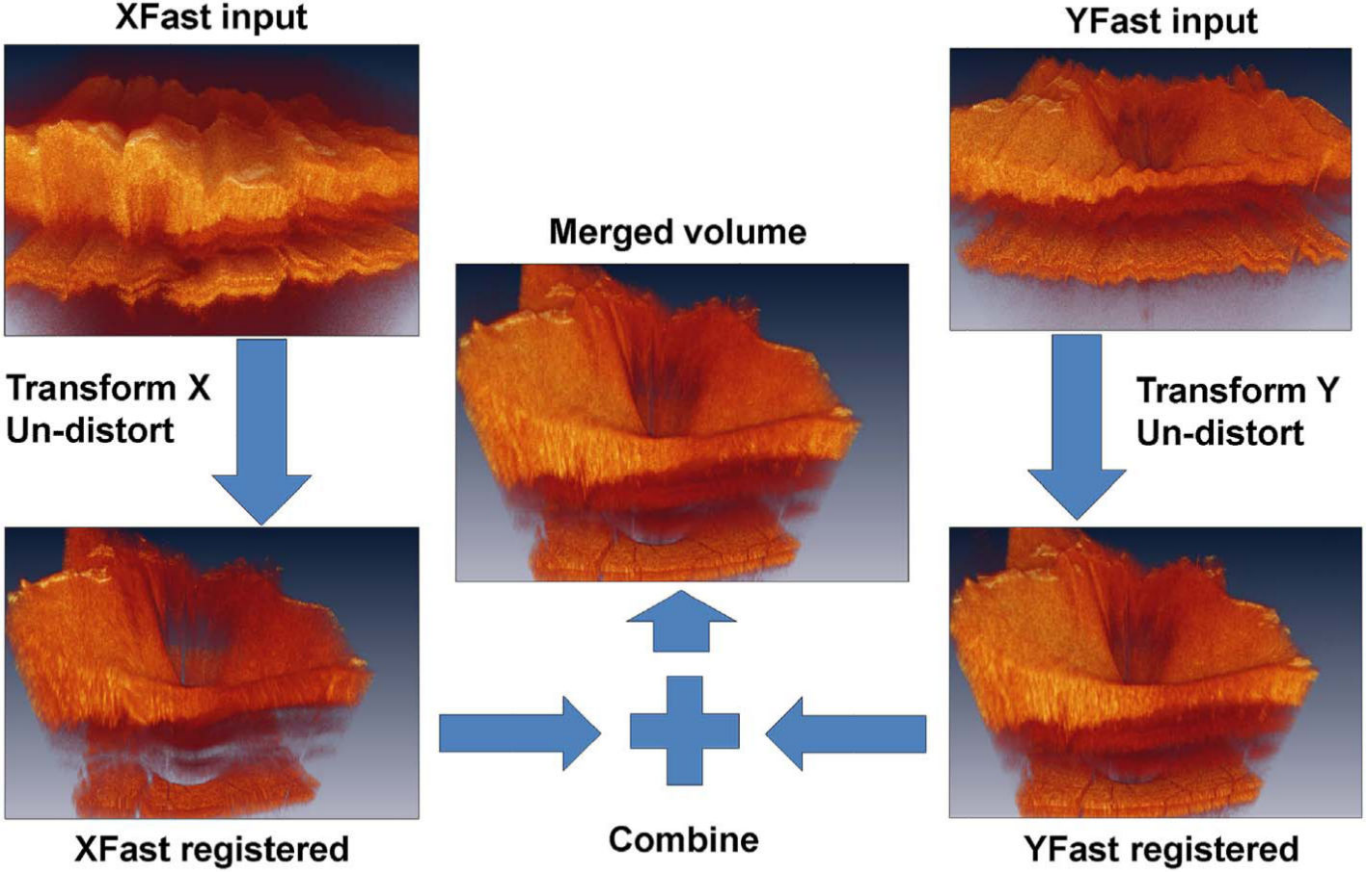
Schematic showing how software motion correction works. Software motion correction can estimate and compensate for eye motion on a per A-scan basis in all three dimensions. Two volumes with perpendicular raster scans are acquired (XFast vs YFast raster directions). These two volumes will have motion distortion in different directions. Displacements are estimated for all A-scans in each of the volumes with the objective of generating undistorted, motion corrected volumes. The two volumes are assumed to be motion corrected if they are similar. The A-scan displacements are calculated using iterative, multi-resolution techniques with a nonlinear optimizer and the resulting motion corrected volumes are merged or averaged to increase the signal to noise. Unlike eye tracking, software methods can correct motion in all three dimensions and can be implemented on different instruments since they do not require hardware modifications.

**Fig. 15.**
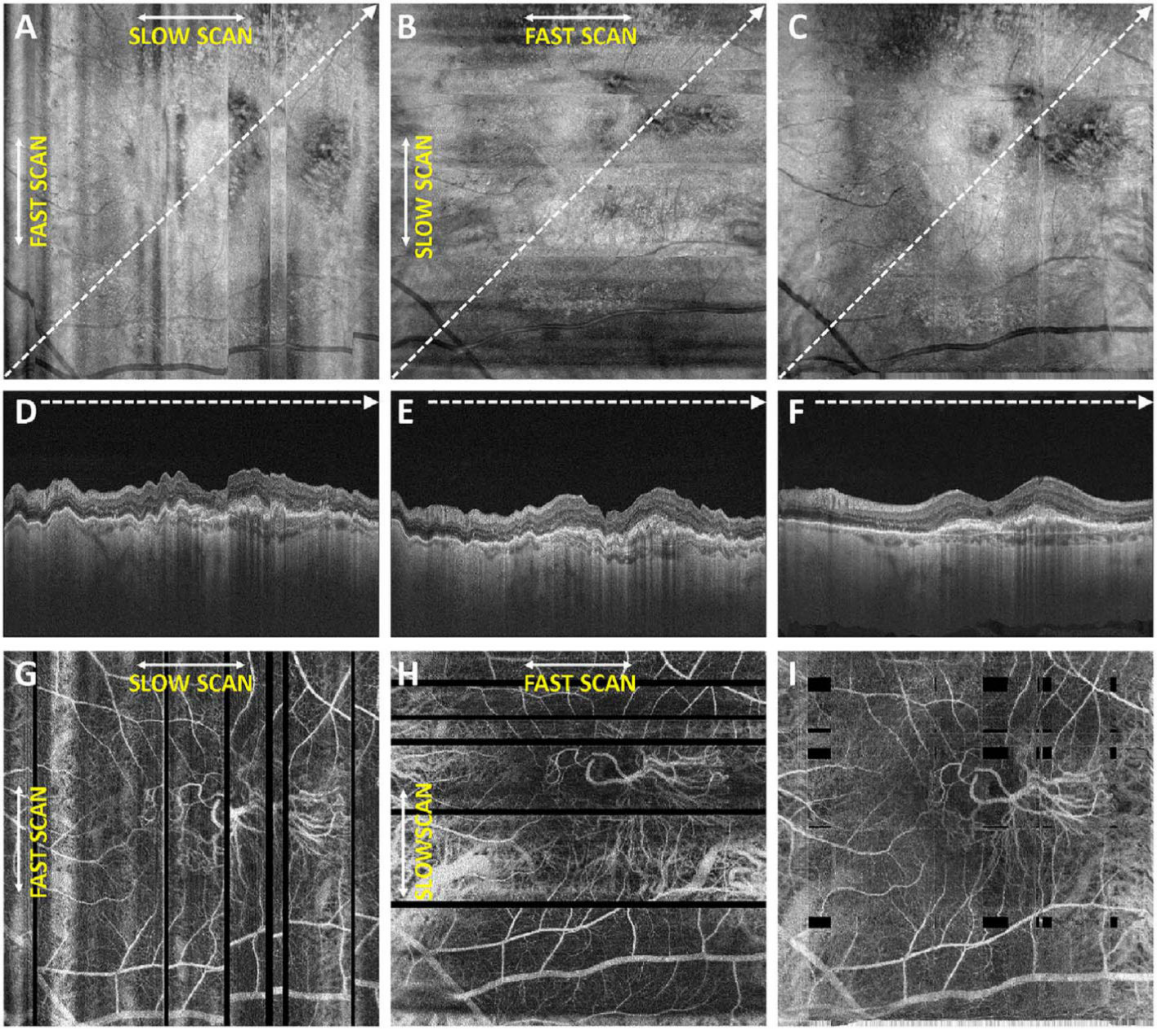
Example of software motion correction and artifacts. Effect of patient eye motion and software motion correction on en face OCT and OCTA data. Data is from an 87-year-old patient with a CNV lesion exhibiting both occult and classic components. (A) En face projection of the Yfast OCT volume through the depths spanned by the lesion. (B) En face projection of the X-fast volume through the depths spanned by the lesion. (C) En face projection of the motion corrected and merged OCT volumes (A) and (B), with the projection is taken through the depths spanned by the lesion. (D–F) OCTA volumes corresponding to (A–C), respectively. The image data (C, F, I) is motion corrected in all three dimensions and signal to noise is improved. However, there are regions where data is missing due to eye motion. These are evident as rectangular gaps in the en face OCTA image (I)

Software registration and motion correction represents a second, complementary method for eye motion correction. Since there is always a latency in eye tracking, software techniques have the potentially can correct for some errors that are not correctable by eye tracking. Fig. 14 shows a software motion correction technique which estimates and corrects for eye motion (Kraus et al., 2012, 2014). Two OCT volumes are acquired using different raster scan patterns. One volume is scanned using a horizontal raster which is typically used in OCT and a second volume is scanned using a vertical raster pattern. Since eye motion accumulates with time, each volume is assumed to be most accurate in the direction of the “fast” scan and has progressively more motion artifacts from B-scan to B-scan in the “slow” scan direction. Software motion correction estimates the displacements of each A-scan in each volume, applying the displacements to the two volumes and then comparing them for similarity. If the displacements which correct for eye motion are accurate, then the two motion corrected volumes will match. Fig. 15 shows an example of software motion correction. En face OCT images of the two input raster scans are shown in 15 A and B with corresponding cross sections 15 D, E. The motion corrected, merged image is shown in 15 C and F where correction of axial eye motion can be clearly seen in the cross-sectional image. Fig. 15 G, H, I show corresponding en face OCTA images before and after motion correction and merging. The en face OCTA image in Fig. 15 illustrates an important limitation of software motion correction, eye motion produces gaps in each of the volumetric data sets where some regions of the retina are not imaged. These gaps in data appear as horizontal or vertical defects in each of the data sets, and appear as rectangles in the merged data set. Since OCTA images are high contrast and continuity of vascular features in the en face plane is important, these data gaps are especially evident. The motion control software can introduce artifactual errors of its own into the image with creation of quilting (a patchwork appearance of the image) and vascular doubling as a consequence (Spaide et al., 2015a).

**Fig. 16.**
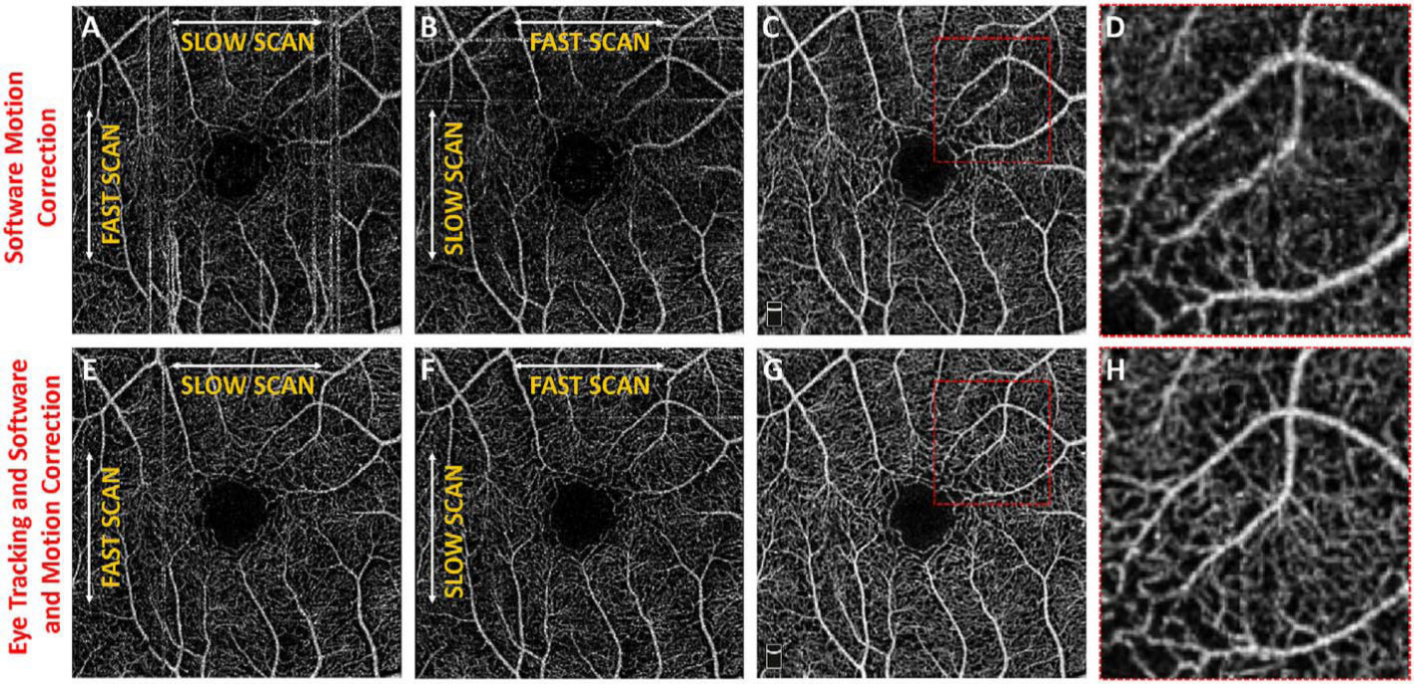
Combined software motion correction and eye tracking. Top row shows software motion correction only, bottom row shows eye tracking and software motion correction. En face OCTA with perpendicular raster scans (A) and (B) have discontinuities from eye motion. Eye tracking reduces the effects of eye motion in (E) and (F). Reducing motion in the initial data improves the results of software motion correction (G, H) compared with software motion correct without eye tracking (C, D). Eye tracking enables longer data acquisition times and large data sets, while also avoiding gaps in data which can occur with software motion correct alone.

The combination of eye tracking to software motion correction has been shown to reduce artifacts associated with software motion correction alone (Camino et al., 2016). Fig. 16 shows a comparison of software motion correction (top row) versus eye tracking and software motion correction (bottom row). Eye tracking and software motion correction reduces white line motion artifacts, gaps in data and residual motion error compared with software motion correction alone. There are high frequency eye movements in some patients, either from a tremor or fast nystagmus that are beyond the capabilities of current eye tracking, software correction or combined approaches. There are a number of possible strategies to use eye tracking and software to compensate for the image degradation caused by eye motion, and individual manufacturers maintain their approaches as proprietary.

### 7.4. Movement within the eye

**Fig. 17.**
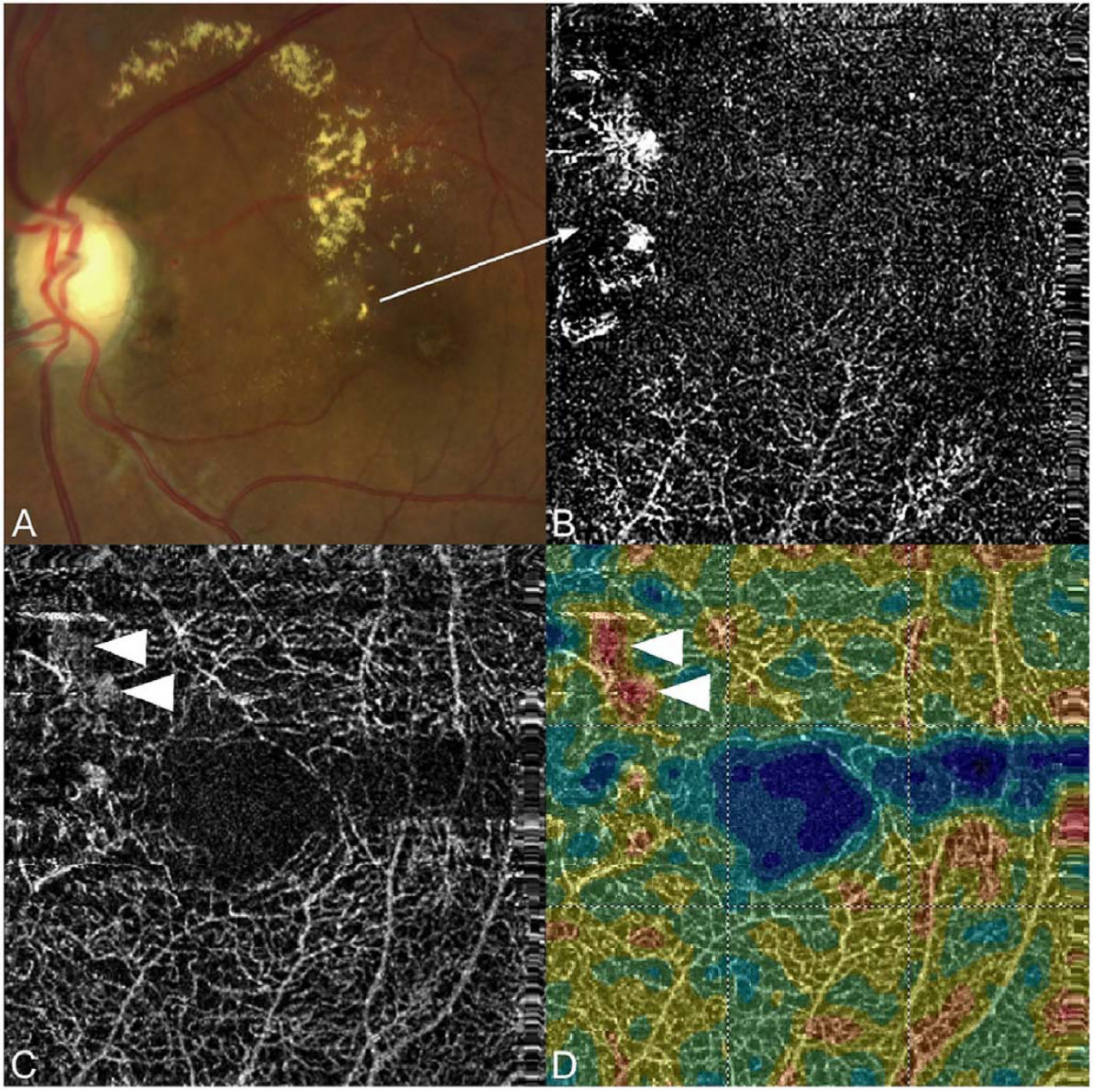
OCTA artifacts from lipid deposits. OCTA decorrelation artifacts generated by intraretinal lipid in a patient with radiation retinopathy affecting the peripapillary retina. (A) There is an area of edema ringed by lipoprotein precipitates in this color image taken a few months prior to OCTA imaging. (B) OCTA projection, segmented at Henle’s fiber layer which is normally an avascular area. Note the lipid generates a high decorrelation signal. (C) Segmented, albeit somewhat inaccurately at the deep vascular plexus, there are artifacts from the lipid as well (arrowheads). (D) Note that even at this level, the false color flow density map shows the lipid as high flow regions (arrowheads). Lipoprotein precipitates generate motion contrast, which resemble blood flow on OCTA.

**Fig. 18.**
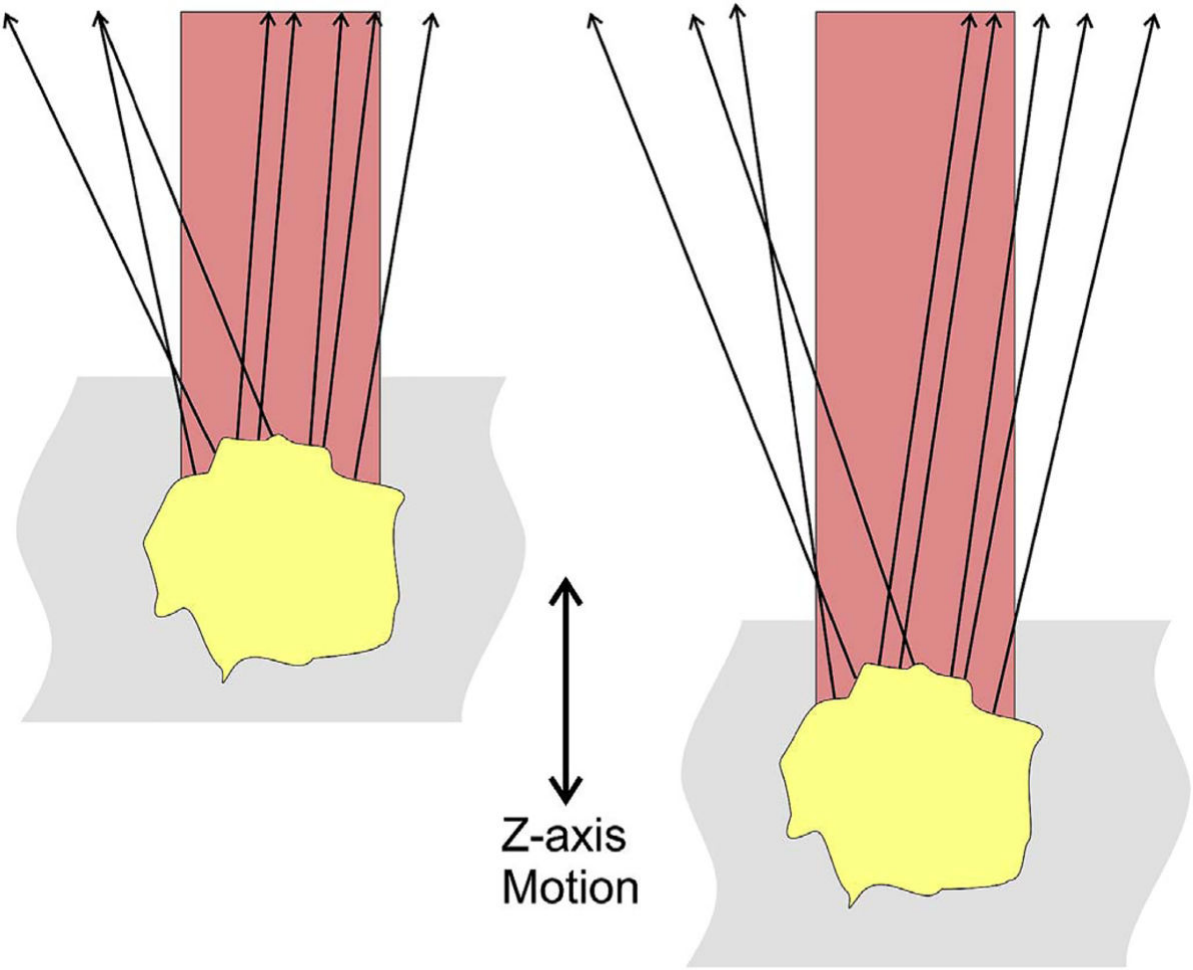
Schematic showing how changes in axial position of a reflective structure, such as a lipoprotein granule, could cause subtle changes in the intensity of the detected reflection. This could cause a decorrelation, which would incorrectly be rendered as a flow signal. Similar changes in reflectivity could occur with transverse motion.

**Fig. 19.**
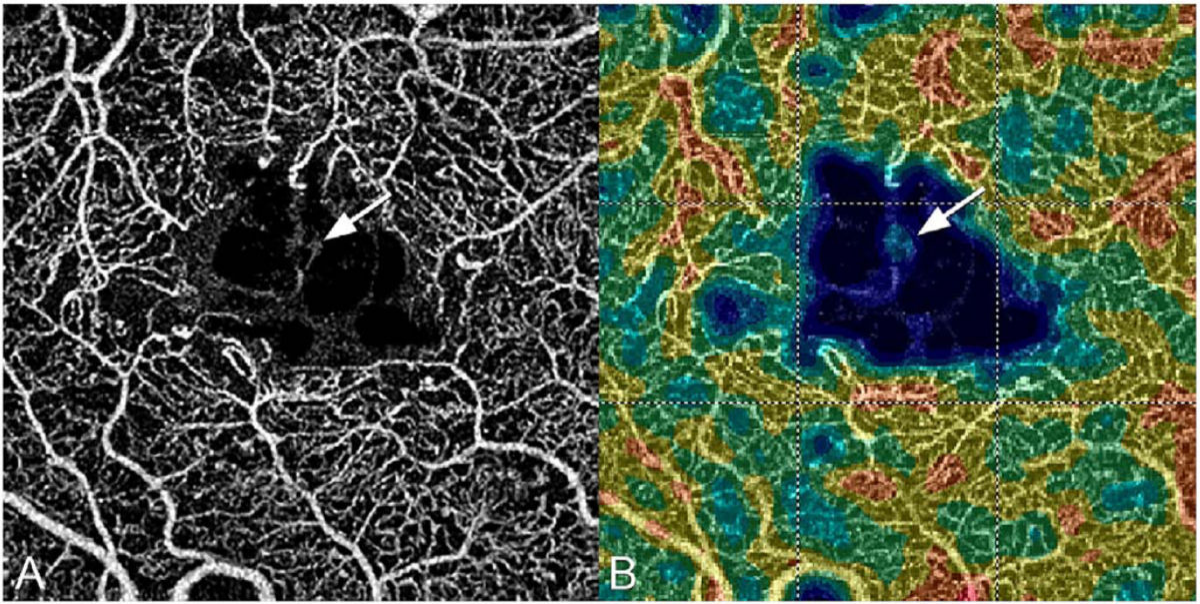
OCTA artifacts from cystoid macular edema. OCTA decorrelation artifacts can be generated by the walls around cystoid spaces. (A) OCTA artifact in an eye with diabetic cystoid macular edema (arrow). There are no blood vessels in the wall of the cystoid space. (B) The false color flow density map shows a low level of flow in the corresponding area (arrow) although no flow is present. These structures exhibit small displacements between repeated B-scans, generating false OCTA signals.

The position of the retina is constantly changing in the axial direction due to pulsatile expansion of the choroid and possible fluctuations in intraocular pressure from the cardiac cycle. Prior to OCTA signal calculation, most OCTA algorithms perform a motion correction of repeated B-scans to reduce the effects caused by bulk eye motion. In OCTA there are some subtle effects that could be magnified. The filling of choroidal lobules is pulsatile and out of phase with each other as well as varying over time (Flower, 1993; Flower et al., 1995). The posterior pole is a curved surface and the underlying choroid is not uniform in thickness, producing a non-uniform axial motion of the retina. Reflections from retinal features or pathology could produce false OCTA flow signals because of variations in OCT signals which are unrelated to blood flow within the pathology itself. This axial motion artifact from smaller particles is very common and is seen in such conditions as diabetic retinopathy and veno-occlusive disease. There are two related artifacts; the first is decorrelation with larger lipid deposits in the retina (Fig. 17). Light may reflect from the surface of the deposits as well as from internal confines, creating a decorrelation because of very small changes in the angles of reflection (Fig. 18). The second is generation of an OCTA decorrelation signal from the walls of cystoid macular edema (Fig. 19). Vascular density maps created by various OCT angiographic instruments are confounded by both of these artifacts and may give the impression that perfusion is better than it really is. Axial motion artifacts can be difficult to discern or compensate because the motion of the choroid is not necessarily uniform or predictable. Transverse motion can also cause a change in reflectivity that would not be compensated for by eye tracking.

### 7.5. Projection artifacts or decorrelation tails

**Fig. 20.**
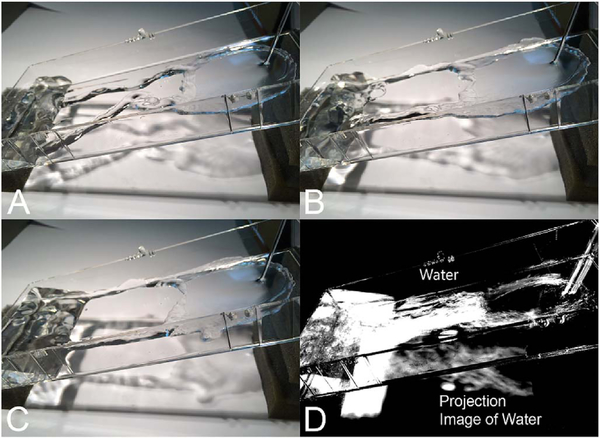
Demonstration showing how OCTA projection artifacts are generated. (A–C) Water is flowing in the clear channel and in repeated images over time, are seen as a region of variable reflections. However, the light transmitted through the water illuminates the background and the illumination also changes over time. The variance (D) of the images can be computed to show motion contrast. The changing background illumination is detected even though the background itself is not moving. Similarly, flowing blood cells can generate motion contrast for stationary structures which are below them. (From Spaide et al., 2015a).

Projection artifacts, also referred to as decorrelation tails, are one of the most important artifacts in OCTA. In order to image deeper structures such as the photoreceptors or RPE, the OCT beam passes through retinal blood vessels where it may be reflected, absorbed, refracted or scattered. Light reaching deeper structures is influenced by overlying retina features, in the case of blood vessels, the transmitted light has time varying fluctuations because it propagates through flowing blood. The deeper features are illuminated by this fluctuating light. Even if these features do not themselves change with time, the changing illumination can create a decorrelation from one B-scan to the next, creating a false impression that there is blood flow in reflective layers below a vessel. Fig. 20 shows an experiment which demonstrates how flowing water can create time varying images of stationary structures below it. To create a projection artifact, a source of varying light is needed, for example a blood vessel, combined with a reflective surface to act as a projection screen. There are several layers in the fundus that act as natural reflectors, such as the plexiform layers and the retinal pigment epithelium. In high myopes, the sclera can exhibit projection artifacts from the choroid. Sometimes projection artifacts can be used to enhance visualization. The choroidal vasculature can sometimes be imaged in high myope eyes better by looking at the projection artifact than by visualizing the choroid directly. This is because the choroid is difficult to isolate using current segmentation algorithms in commercial instruments and the sclera is a good reflector, enhancing the projection effect (Maruko et al., 2016). The choriocapillaris is dense vascular structure which produces strong OCTA projection artifacts for choroidal structures below it. For example, the stroma between larger deeper vessels in the choroid appears bright, presumably from a projection artifact from the choriocapillaris.

**Fig. 21.**
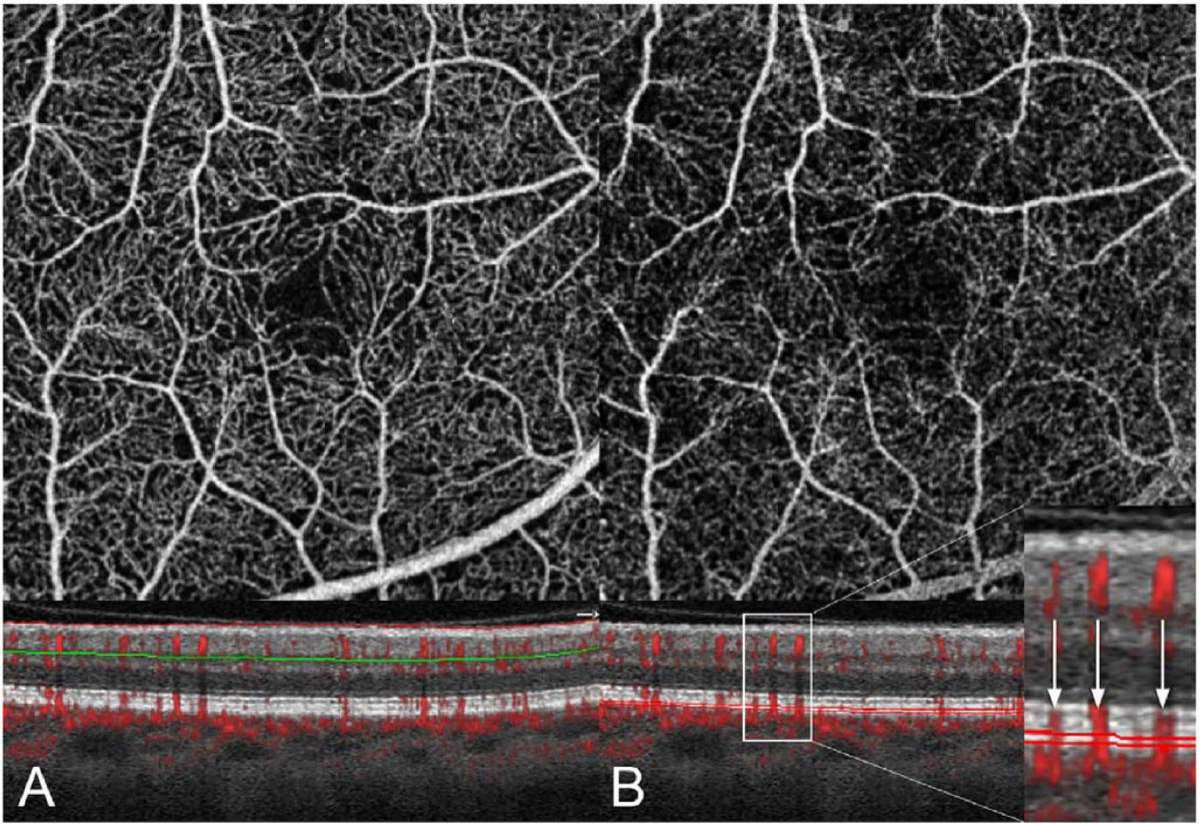
Projection artifacts in OCTA. (A) The inner vascular plexus is shown in an en face OCTA image (top), along with a B-scan and flow overlay (bottom). (B) An en face OCTA at the level of the RPE exhibits a flow signal that looks like the inner vascular plexus. The B-scan with flow overlay and an enlarged inset are shown (bottom). The vessels with flow in the inner plexus produce projection artifacts in the RPE and subretinal structures which are stationary (arrows).

The classic example of a projection artifact occurs when visualizing the retinal blood vessels at the level of the RPE (Fig. 21), and nearly every OCTA image exhibits these projection artifacts. Examining the cross-sectional B-scan image along with the cross sectional OCTA which overlays flow information is one way to estimate the amount of projection artifact present. The projection artifact occurs at reflective layers, such as the RPE which directly underlie the more superficial vessel. The reflective structure can also be drusen, focal pigmentation, or lipid, so care should be used in assessing the OCTA appearance of flow in the deep retina or subretinal space. (Lipid can serve as a screen for projection artifact in addition to having axial motion artifact.) The projection artifacts from the retina are attenuated by the RPE, so that choriocapillaris images often have only subtle projection artifacts. More significant retinal vessel projection artifacts occur on the choriocapillaris is in cases where the overlying pigment is attenuated or counterintuitively, in cases where the signal strength for the scan is very high.

**Fig. 22.**
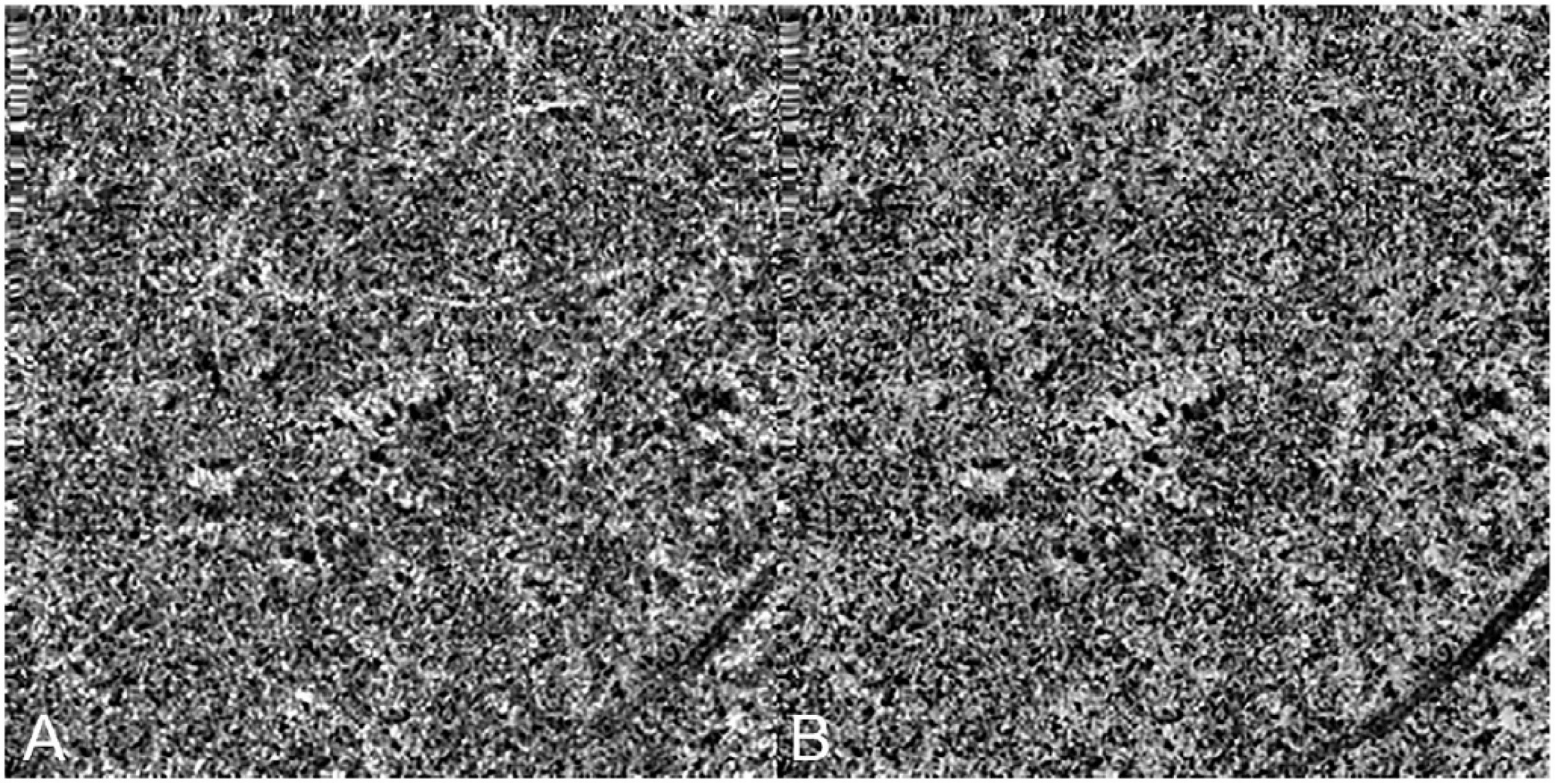
Projection artifact removal. The en face OCTA image in (A) is at the level of the choriocapillaris, but exhibits projection artifacts from the retinal circulation. These projection artifacts can make interpretation difficult because vascular features can appear in structures without vasculature. (B) The OCTA signal from the retinal circulation was used to remove the projection artifact from the en face OCTA image of the choriocapillaris. Manufacturers have implemented different algorithms to reduce or remove projection artifacts.

Multiple approaches have been investigated for removing or reducing projection artifacts (Zhang et al., 2015a, 2016). A number of techniques have been implemented in commercial instruments and the exact algorithms are proprietary. One of the simplest methods is to subtract the OCTA en face image of the superficial vessels from a deeper layer. This removes the bright projections, but replaces them with dark versions of the vessels, producing an unacceptable artifact in its own right. Other methods borrow from techniques used in microscopy and astronomy. The idea is that the reflectance from a sample is related to the amount of light available, changes in thickness of the sample, and the optical transmission of the instrument making the image. For example, if one part of the sample is illuminated half as much as another part, that region would be darker. We can compensate numerically for some of these alterations by dividing the image of the sample by a profile established for the illumination. This can be adapted to OCTA. The image of the outer retina, which ordinarily has no vessels, suffers projection artifact from the overlying retina. As a consequence, there will be ambiguity in OCTA images of MNV from this region. By taking the image of the outer retina and dividing that by an image of the retinal vasculature, the projection artifact can be reduced (Zhang et al., 2015a). This method also can lead to dark vessel artifacts. However, if the division is done in a graded manner by adjusting the opacity of the dividing layer, fairly good results can be obtained. In addition to this modification, the approach can be generalized to remove artifacts from other layers. For example, Fig. 22 shows an image of the choriocapillaris without and with projection artifact removal. The problem of the slab based approach is that segmentation is needed and this segmentation can have errors. In addition, the weighting of the overlying blood vessels in terms of their contribution to the projection artifact cannot be easily modified with this approach. A more recent method to reduce OCTA projection artifacts operates on individual Ascans and is known as projection resolved OCTA (PR-OCTA). This algorithm identifies OCTA voxels with flow by normalized value of the OCTA decorrelation with OCT intensity and comparing it to shallower voxels in the same A-scan (Zhang et al., 2016). The values deeper than the peak level are converted to zero. Projection resolved OCTA has been shown to preserve the continuity of deeper vascular layers better than previous methods. This technique theoretically would have difficulties in regions where more than one layer of vessels exist. Research on this topic is ongoing and it is likely that algorithms will continue to improve.

### 7.6. Segmentation artifacts

The most important set of artifacts are secondary to segmentation errors (Spaide et al., 2015a). En face images are created by selecting one or more layers of the retina and viewing the vessels summed or projected over the desired layers. The volumetric retinal OCT data is first processed to detect various retinal layers using a software image processing approach known as segmentation. The strategy to detect layers initially starts with healthy eyes and good quality OCT data. Potential parameters to differentiate layers can be based on reflectivity, texture, or other attributes, directly or using Bayesian inference, deep learning, etc. Continuity of layers is also often used in segmentation algorithms. The problem is that these parameters rarely are effective in eyes with pathology which disrupts the normal retinal architecture. OCTA data can have surprisingly serious errors from incorrect segmentation. The typical problems associated with segmentation errors are that layer thickness can be thinner or thicker than normal, there can be an absence of layers, or altered curvature of the eye, such as in high myopes.

**Fig. 23.**
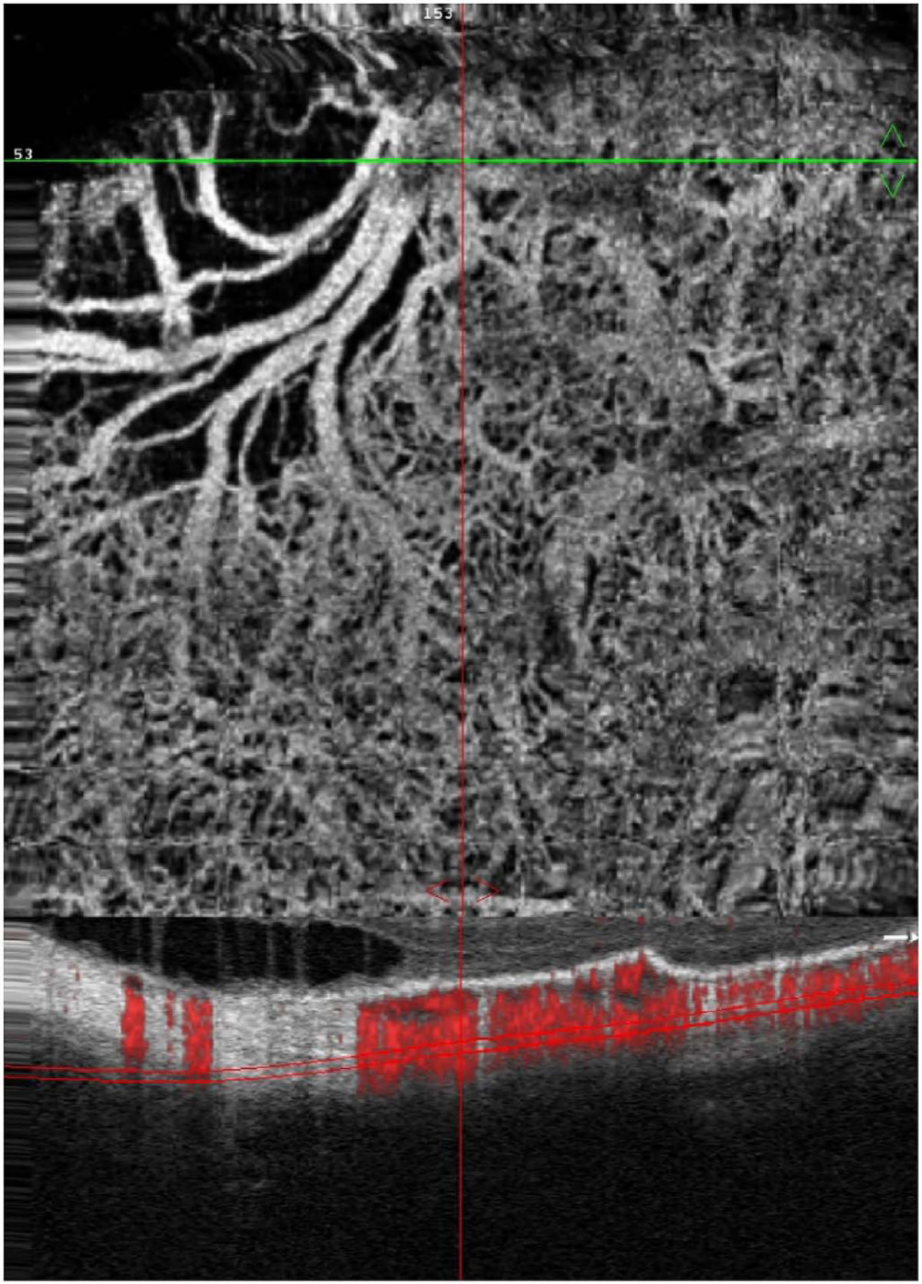
Segmentation errors. Example showing segmentation error in a patient with high myopia. The en face OCTA image (top) shows blood vessels of many different sizes. The upper left does not appear to show any flow. (Bottom) The segmentation contours, shown in a B-scan with a flow overlay, exhibits serious errors. The segmentation contour was intended to be the choriocapillaris but deviates into the choroid, sclera and is outside of the eye entirely (left side). This produces the black area in the upper left corner of the en face OCTA image because the image contains information from the wrong depth. Segmentation errors can cause severe errors in interpretation. The risk of errors can be reduced by viewing cross sectional images to confirm the integrity of the en face projection images.

Automatic segmentation of vascular layers in a high myope with a staphyloma is rarely correct with current instruments, even in the absence of complicating factors such as choroidal neovascularization (CNV) or schisis. Fig. 23 shows a high myope with the choriocapillaris as the intended segmentation. Note the segmentation in the B-scan with the OCTA flow overlay is in the sclera and on the left side of the image, the segmentation leaves the eye entirely. Eyes with pronounced macular edema secondary to diabetic retinopathy or veno-occlusive disease are almost never segmented correctly. The edematous layers are thicker than the segmentation slabs used and the segmentation slabs are often not in the correct layer to begin with. This yields a vascular image, but often without clinical meaning because the image is a merger of disparate vascular layers.

Interpretation errors from incorrect segmentation can be avoided by viewing the segmentation contours in sequential OCT B-scan images to confirm that they are correctly located. Many instrument manufacturers have software that enables hand correction of segmentation errors and propagation of corrections across multiple B-scans, however this can be a time consuming process. Orthoplane viewing will reduce interpretation errors in OCTA because it enables vascular features to be localized in depth, while also viewing en face structure across the fundus. Research in segmentation is ongoing, however because of the complexity of retinal pathology and the pronounced distortion of architecture that is associated with advanced disease, segmentation is likely to remain a challenging problem.

## 8. The multimodal imaging approach

The last two decades have witnessed dramatic advances in retinal imaging with a variety of new technologies, such as OCT, autofluorescence, and confocal multicolor imaging, becoming widely available to complement previous imaging technologies such as flash fundus photography and dye-based angiography. The capability of each, in turn, was extended by newer wide-angle imaging technology. OCTA is in the succession of ocular imaging techniques, but enters into a family of inter-related modalities used to evaluate ocular health and disease. As such no one modality reveals all information about every disease. It is imperative to keep the findings of any one modality in perspective by integrating that information with potentially useful data obtained by other imaging methods. The principles and merits and limitations of these various imaging technologies are briefly reviewed in this section, particularly with regards to their visualization of the retinal microvasculature.

### 8.1. Color fundus photography

**Fig. 24.**
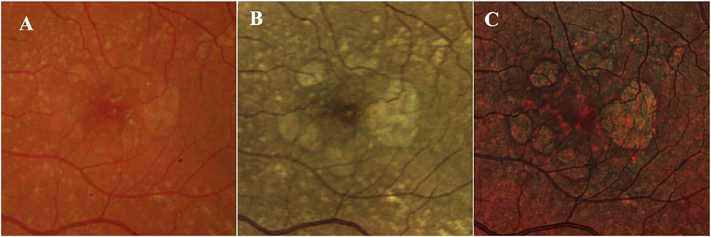
Fundus photos of the left eye of a patient with advanced non-neovascular age-related macular degeneration and geographic atrophy (GA). A. Image with a typical flash white-light fundus camera system (Kowa VX-20). Note natural appearance of the retinal blood vessels and drusen. The borders of the GA are difficult to discern. Confocal white light (B, Centervue Eidon) and multicolor (C, Heidelberg Spectralis) of the same eye. The appearance of the color is different, especially for the multicolor image, but the borders of the atrophy are easier to discern with the confocal systems.

Color fundus photography has been the centerpiece of imaging in ophthalmic clinical trials and clinical practice for several decades (Klein et al., 1991). A major advantage of color fundus photography is that it replicates the view of the retina that the physician sees when examining the patient with ophthalmoscopy. As such, color fundus images are easy to interpret and are useful tools for documenting findings seen on examination, and vessels and vascular abnormalities are visible as reddish structures (Creuzot-Garcher et al., 2014; Higgs et al., 1991). Color photography can produce realistic colors by using combinations of red, green, and blue (Fig. V1-A). Though the larger retinal blood vessels are evident on color photography, there is insufficient contrast to visualize the capillary circulation. It is possible to look at the individual color channels. Examining the green channel, which is created by wavelengths in the green portion of the spectrum shows a monochromatic image in which blood vessels are dark because blood absorbs green light. Color fundus photographs have traditionally been captured using flash-based systems using white light, with instruments ranging from large table-top devices to smartphones (Němčanský et al., 2014). A limitation of flash-based systems is that they are prone to a variety of image artifacts and quality problems such as poor contrast, lighting irregularity, and over- and under-exposure (Heaven et al., 1993; Johnson et al., 2003). Many of these problems are easily addressed by an experienced photographer. Some of these quality issues have been addressed by the recent introduction of devices using white light but featuring a confocal pinhole (Malinovsky, 1996) (Fig. 24-B). Pseudocolor images can be generated by scanning laser ophthalmoscopic (SLO) acquisition through the use of multiple lasers of different wavelengths (Moussa et al., 2015; Tan et al., 2016a). Some SLO systems use infrared instead of actual red light and bluish green light instead of blue light. The resultant images are therefore pseudocolor representations (Fig. 24-C). SLO systems require more time per channel to acquire images increasing the possibility of image distortion caused by patient movement. The differential ability of these newer pseudocolor imaging approaches to visualize retinal features seen on standard color photographs is still under investigation. The theoretical resolution obtained with a SLO system may exceed that of a fundus camera system.

### 8.2. Autofluorescence imaging

**Fig. 25.**
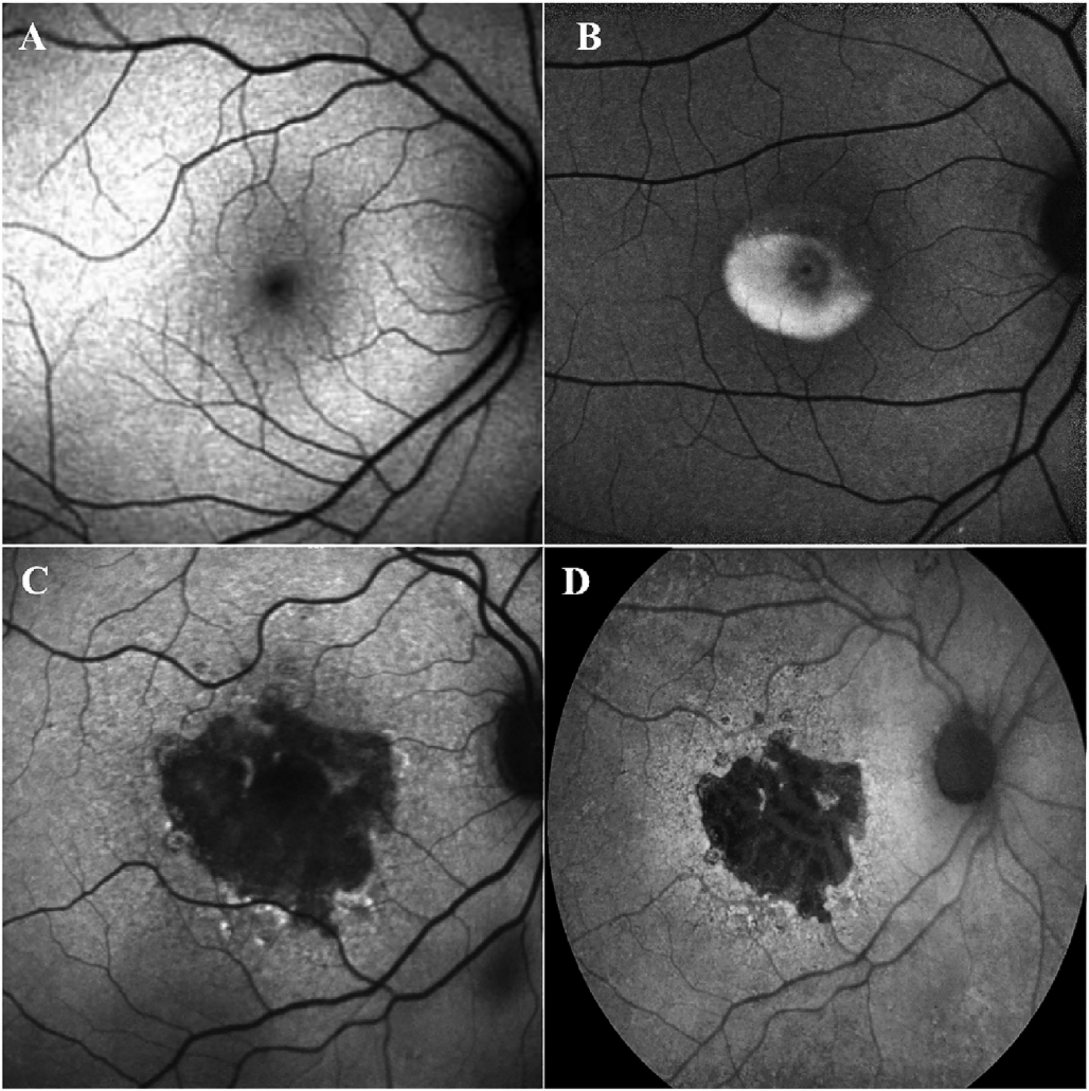
Fundus autofluorescence (AF) imaging. A. Blue-light scanning laser ophthalmoscope FAF image of a normal right eye. Retinal blood vessels are dark and seen in sharp contrast as they block the AF from the retinal pigment epithelium (RPE). The optic nerve head is similarly dark due to the absence of RPE at this location. In addition, as blue light is absorbed by the macular pigment, the central macula (fovea) is also relatively hypo-autofluorescent. B. FAF image of the eye of a patient with Best’s vitelliform macular dystrophy. The vitelliform lesion is intensely autofluroescent. Blue-light scanning laser ophthalmoscope (C) and green-light flash fundus camera (D) FAF imaging of a patient with geographic atrophy (GA). The high contrast of FAF imaging, with absence of the AF in the region of photoreceptor and RPE loss allows the borders of the atrophy to be precisely defined.

In AF imaging (Fig. 25), the retinal tissue is exposed with light of a given set of wavelengths, and fluorophores in the tissue intrinsically emit light with a longer wavelength (Delori et al., 2001; SchmitzValckenberg et al., 2008; Weinhaus et al., 1995). This is in contrast to standard reflectance imaging, where the reflected light is of the same wavelength. Thus to isolate the AF signal, barrier filters are also introduced into the light return path to exclude reflected light. By the introduction of appropriate excitation and barrier filters, Spaide and colleagues demonstrated that a standard flash fundus camera can capture AF images (Spaide, 2003) (Fig. 25-D). The wavelengths available for AF imaging using a flash fundus camera, however, are restricted by the properties of the anterior segment of the eye. For example a yellowish cataractous lens can demonstrate significant AF for blue lightthis signal may be difficult to differentiate from AF originating from the retina and reduce the overall image contrast. Shifting the excitation to the green portion of the visible light spectrum reduces the amount of lens derived autofluorescence. Another strategy to suppress this interfering signal from the lens, however, is to introduce a confocal pinhole (Fig. 25-A), which allows for rejection of AF from the crystalline lens. Confocal systems image light from conjugate planes and therefore accept light from a narrow depth within the eye. The AF image can be further improved by using SLO acquisition and averaging multiple frames collected over time to enhance the signal-to-noise ratio. Using a confocal SLO allows blue light to be used for excitation; it is a simple matter to use the same excitation wavelengths for FA.

The AF signal has been of particular interest as much of it appears to originate from lipofuscin accumulating in the RPE, and may thus provide information regarding RPE function and physiology. AF is known to increase slowly over time with age, but can be dramatically increased in certain diseases such as Stargardt’s disease (Strauss et al., 2016a, 2016b). Increased AF may be produced by fluorophores in locations other than the RPE. A common pathophysiologic mechanism is observed with detachment of the macula. The photoreceptors are separated from the underlying RPE, which ordinarily phagocytizes the outer segments. These can accumulate and over time develop increasing amounts of fluorophore as seen in central serous chorioretinopathy or vitelliform macular dystrophy (Fig. 25-B) (Chung and Spaide, 2004; Ferrara et al., 2010). Decreased AF may be observed as a result of lack of excitation light reaching the fluorophores – this is observed in the normal macula with absorption of blue light by luteal pigment. Decreased or absence of AF may also be an indication of loss of the RPE and photoreceptors, with geographic atrophy being the prime example (Bearelly and Cousins, 2010; Holz et al., 2001). The high contrast of AF imaging for identifying areas of atrophy (Fig. 25-C and D) has driven the use of this technology as an efficacy end point for major trials. With blue light AF, areas of decreased AF must be distinguished from blood vessels which appear dark due to blocking of the signal from the underlying RPE as well as from macular pigment (at highest density in the fovea) which absorbs blue light (Fig. 25-A). Alternate AF imaging wavelengths (green or near infrared) can mitigate this absorption effect because they are outside of the absorption spectrum of macula pigment. Although, there is good contrast for visualizing blood vessels on AF images, the capillaries are not visualized.

### 8.3. Dye-based angiography

**Fig. 26.**
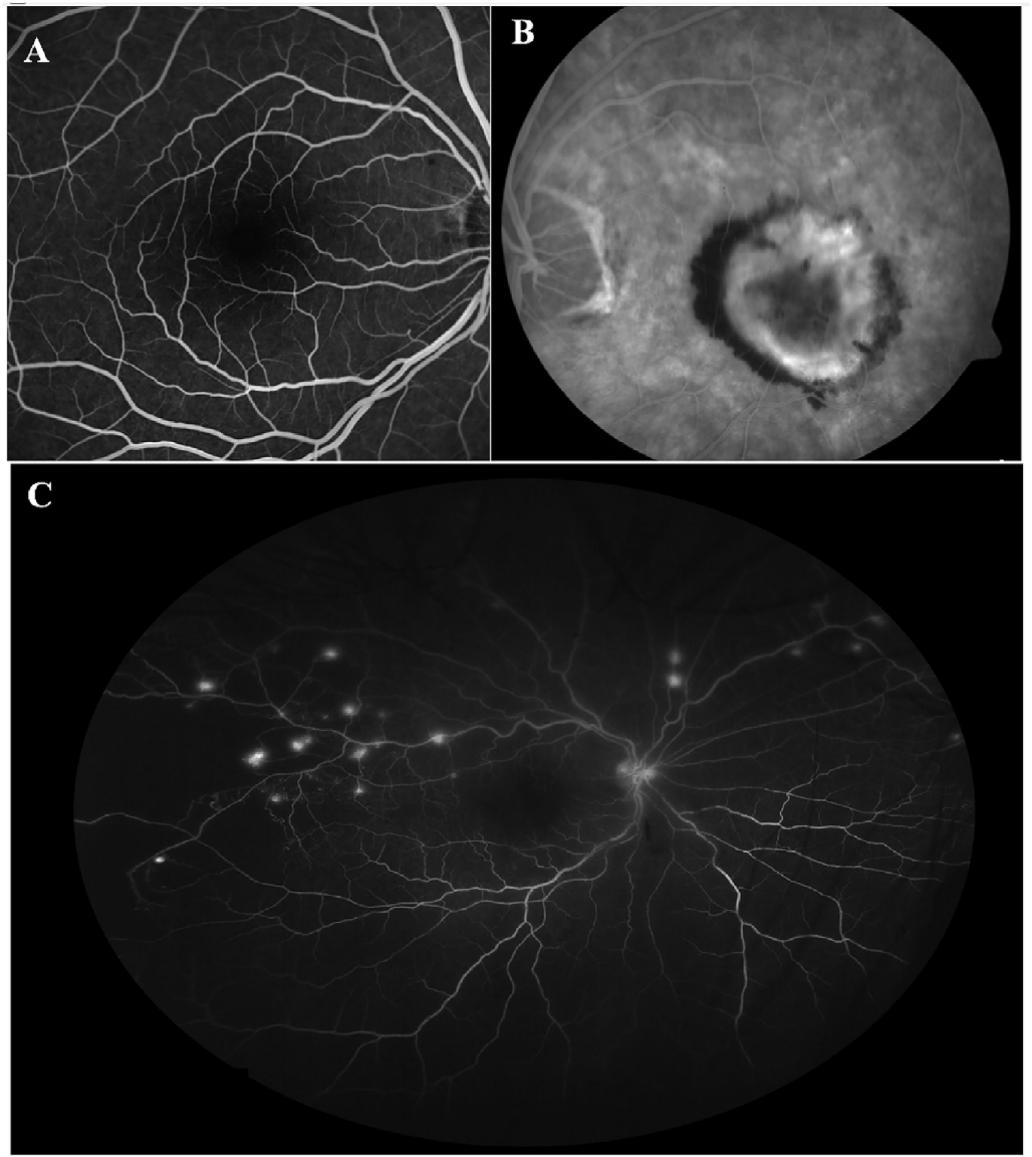
(A) Flourescein angiogram of the macula of a normal eye using a confocal scanning laser ophthalmoscopic system. The parafoveal capillaries and the foveal avascular zone may be discerned, but the capillary circulation outside this region are not evident. (B) Fluorecein angiographic image of an eye with a classic choroidal neo-vascularization (CNV). Although hyperfluroescence from the vascular lesion is evident, the microvascular network of the CNV lesion is not apparent. Leakage of dye, however, is evident. (C) Widefield fluorescein angiographic image (Optos 200Tx) of an eye with diabetic retinopathy. The eripheral non-perfusion temporally can be recognized by the absence of the large vessels and the feature-less appearance of the retina. The capillary circulation outside of the central macula is not evident. Multiple small areas of retinal neovascularization are identified by the hyperfluorescent leakage of dye, but the fine vessels of the neovascular tufts cannot be seen.

To enhance contrast for visualizing the capillary circulation, exogenous intravenous fluorescent dyes may be used. FA in humans was first described by Novotny and Alvis in 1961 (Novotny and Alvis, 1961), and has revolutionized the study of retinal diseases (Chakravarthy et al., 2007). Fluorescein absorbs blue-green light and emits green light similar to lipofuscin, but the magnitude of the signal is several orders greater than intrinsic AF. FA for the first time provided in vivo visualization of the retinal capillary circulation with exquisite detail (Fig. 26-A). Careful comparisons with retinal whole mounts by Weinhaus et al., however, revealed that visibility of the capillaries rapidly decreases with increasing distance from the foveal center, especially for the deeper capillary circulation (Weinhaus et al., 1995). In addition, capillary visualization rapidly dropped with capillary size, with only 40% of capillaries under 4.5 microns evident on FA. Using adaptive optics to improve transverse resolution can dramatically enhance capillary visualization and adaptive optics-based FA (Chui et al., 2014; Pinhas et al., 2014) has demonstrated the radial peripapillary capillary network not visible on conventional FA. Adaptive optics imaging, however, is limited by a small field of view, expense, and lack of commercial instruments for FA. In addition, it also has limited depth resolution and thus the examiner would need to preselect imaging the superficial vascular versus the deeper capillary circulations. Despite these limitations, FA imaging offers many other advantages including dynamic information regarding the transit of blood as well as identification of dye leakage from disruption of the blood-retinal barrier by disease (Fig. 26-B). It is simple, widely available, captures large areas of the fundus in a single image, and each image can be captured in a fraction of a second. For example, a high quality angiogram can be obtained in a patient with nystagmus even though the eye is in constant motion.

FA imaging is also limited in that it provides limited information about the choroidal circulation. This is because fluorescein is a small molecule and it rapidly exits from the fenestrations of choriocapillaris which creates an early “choroidal blush” which obscures details of the deeper choroid (Mames et al., 1991). This problem may be addressed by the use of an alternative dye, indocyanine green (ICG), which is imaged in the infrared wavelength regime. ICG is significantly protein bound and thus remains within the choroidal vasculature in the absence of disease, providing excellent visualization of the medium and largersized choroidal vessels (Yannuzzi et al., 1993). Because the resolution is limited, the fine details of the choriocapillaris cannot be seen. The image in dye based angiography changes over time after the injection and additional information can be gleaned from these images.

Although dye-based angiography has been the gold standard for imaging the retinal and choroidal circulation for several decades, other limitations of the technique must also be considered including the need for intravenous injection, allergic and anaphylactic reactions, and the need for skilled personnel to perform the injections.

### 8.4. Wide angle imaging

A limitation of conventional color fundus photography and dyebased angiography is the field of view (typically 30–50°) which allows only a portion of the fundus to be sampled at one time. This can be partially overcome by obtaining multiple images and montaging them together. It may still be difficult, however, to access the far periphery beyond the equator. In addition, for dynamic studies such as dye-based angiography, the separately-acquired images may be from different phases of the angiographic sequence. These limitations are largely addressed by the recent broad availability of widefield imaging (Fig. 26C), including contact and non-contact (Spaide et al., 1998; Staurenghi et al., 2005) devices such as the Optos instruments (Klufas et al., 2015; Nagiel et al., 2016) and the wide-angle module for the Heidelberg Spectralis (Witmer and Kiss, 2013). Wide-angle imaging, however, does not overcome the other limitations of pseudocolor photography and dye-based angiography. In fact, the larger field of view can further limit resolution and impair visualization of the finer capillaries (Tan et al., 2016c).

### 8.5. Optical coherence tomography

**Fig. 27.**
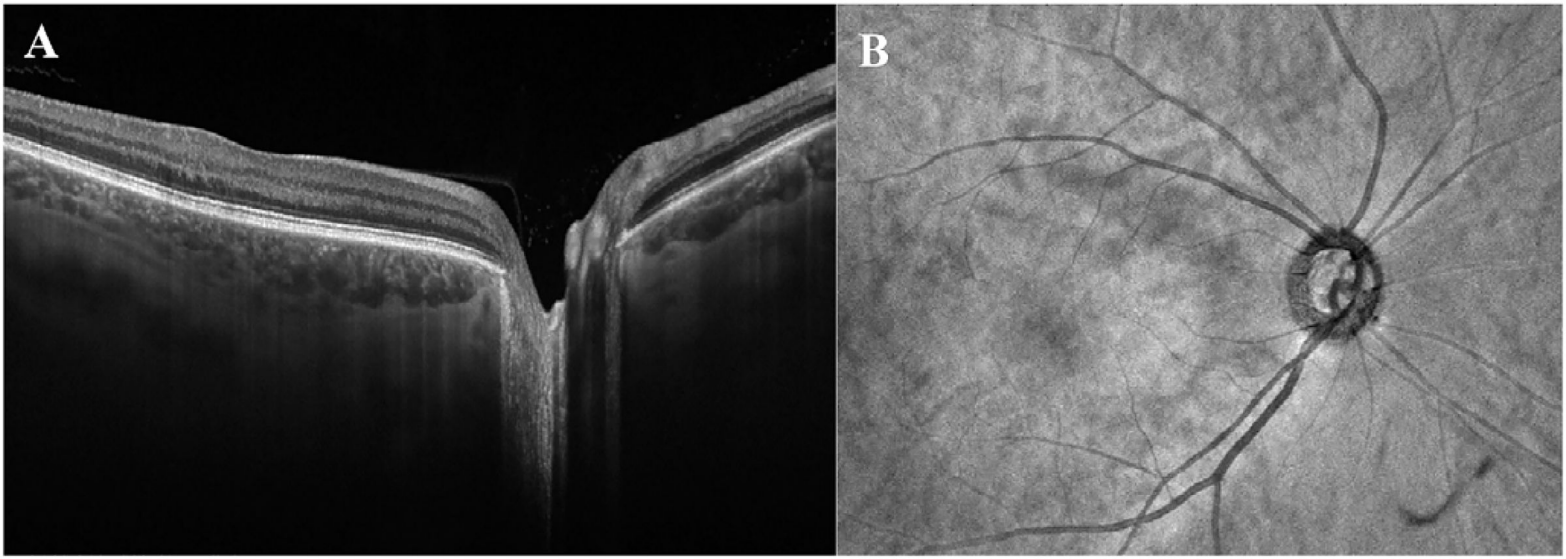
(A) Swept source OCT (Topcon Triton OCT) B-scan of the macula and optic nerve of a normal eye. The choroid and lamina cribosa of the optic nerve are well-seen as are the various layers of the neural retina. The hyper-reflective oval profiles of the large vessels of the optic nerve head are also evident. (B) En face OCT from a dense swept source OCT acquisition (1024 × 1024 A-scans). Though the retinal vessels are well-seen, even at this “megapixel” resolution, the retinal capillaries cannot be visualized.

OCT has transformed ophthalmic imaging over the last two decades. The many advantages of OCT include the excellent axial resolution (allowing the depth of structures and abnormalities to be precisely defined), speed of acquisition, patient comfort, and limited training required for high-quality imaging. By providing detailed visualization of the various retinal and choroidal layers, OCT has transformed our understanding of numerous retinal diseases (Pappuru et al., 2012). Larger retinal blood vessels are well seen on both structural OCT B-scans and en face OCT images (Dansingani et al., 2016). Current structural OCT, however, lacks the transverse resolution or contrast to visualize the capillary circulation (Fig. 27). The signal arising from the internal portions of deeper larger choroidal blood vessels is very low due to the loss of signal with depth, scattering by the RPE and choriocapillaris, and because blood is not a good reflector of infrared light. Loss of signal with depth can be partially compensated for with enhanced depth imaging (EDI) or swept source (SS) OCT (Tan et al., 2016b). The net result is the blood flow cannot be visualized within the larger choroidal vessels if the RPE and choriocapillaris is intact. Nonetheless, the fine details and organization of the choriocapillaris are difficult to discern with structural OCT and so are not well imaged with OCT no matter what the implementation, be it SD or SS OCT.

## 9. Normal circulation in the eye

The retina has one of the highest oxygen consumption rates of any tissue in the body and is highly susceptible to irreversible injury during states of vascular compromise (Anderson Jr., 1968; Yu et al., 2007). The vascular supply to the eye is provided by the ophthalmic artery which originates from the internal carotid artery. The ophthalmic artery branches to form the central retinal artery, the short and long posterior ciliary arteries, and the anterior ciliary arteries (Hayreh, 1975; Kiel, 2011). Blood enters the choroid through the posterior ciliary arteries. The short posterior ciliary arteries, generally 15–20 in number, perforate the sclera around the optic nerve then arborize to form the arterioles of the outer layer of the choroid. These arterioles terminate in the choriocapillaris (Hayreh, 1975; Kiel, 2011). The choriocapillaris is a single continuous layer of wide-lumen fenestrated capillaries arranged in one thin sheet plane creating an anastomotic network. The basement membrane of the capillary endothelial cells forms the outermost layer of Bruch’s membrane (Nickla and Wallman, 2010).

**Fig. 28.**
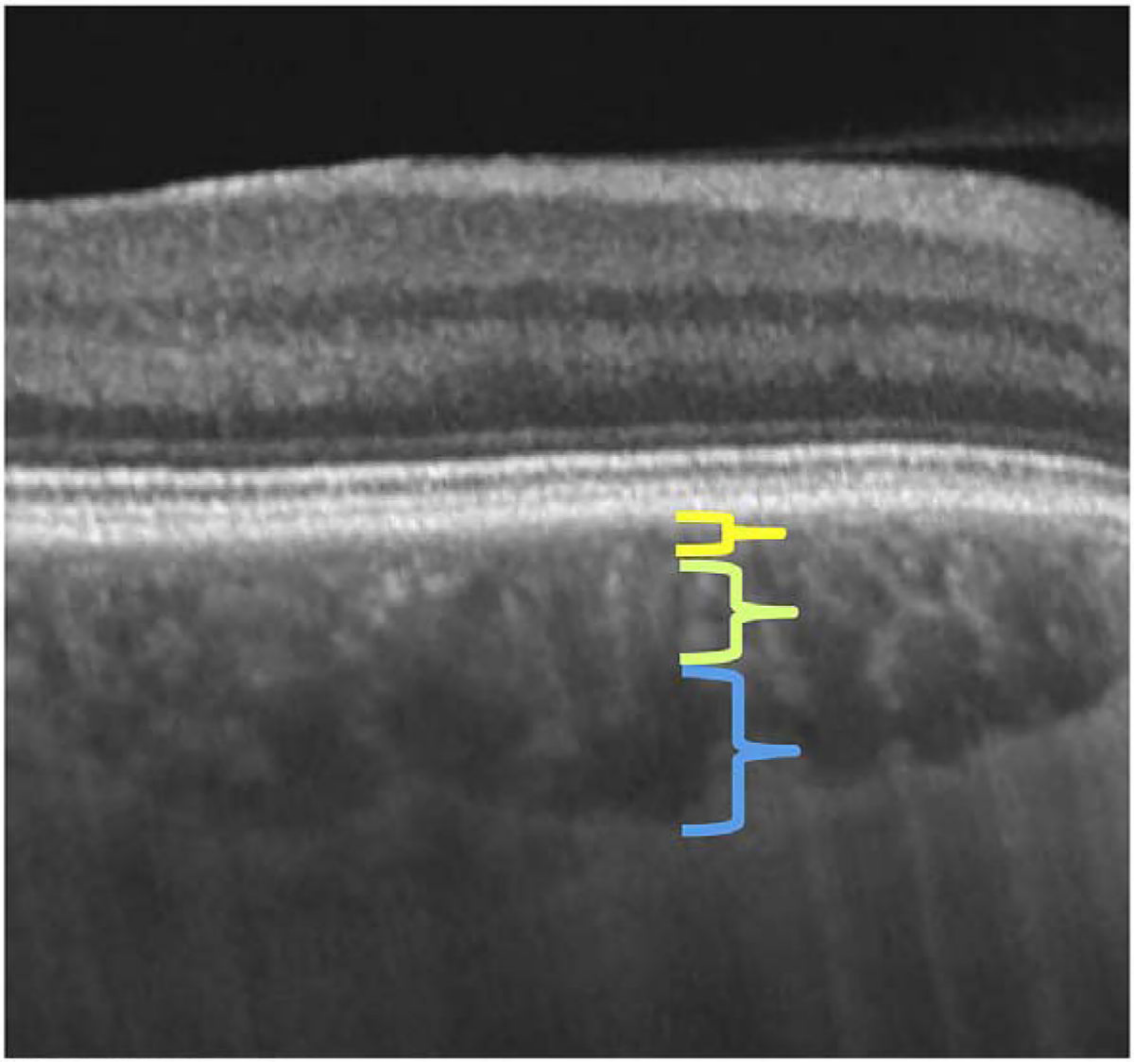
Swept source OCT B-scan (Topcon Trion OCT) of a paramacular region of a normal eye. The approximate vertical dimensions of the various vascular layers are shown with colored calipers: what is referred to as the choriocapillaris (yellow), Sattler’s layer of medium-sized vessels (green), Haller’s layer of large-sized vessels (blue). Note, no clear demarcation can be discerned between these various layers and the depth of the choriocapillaris is much less in life than what is commonly depicted in OCT sections.

Blood is collected by postcapillary venules, which anastomose creating medium-sized vessels in Sattler’s layer and larger vessels in Haller’s layer (Nickla and Wallman, 2010). Sattler described the histologic structure of the choroid in detail, including the choriocapillaris, which he concluded was a layer of capillaries that had an endothelial lining similar to small veins. He also found several layers of elastic fibers, some forming a membrane under the choriocapillaris that contained small arteries and medium sized veins. Other anatomists were less able to visualize the elastic membranes, but named the region of vessels in the middle of the choroid, Sattler’s layer anyway. Haller corrected a previous anatomic assumption by positing that the outer vessels coursing toward the ‘vasa vorticosa’ were actually veins, not arteries. The entire layer of outer vessels of the choroid was given the name Haller’s layer, even though the outer layer of the choroid contains more than large veins. The names Sattler’s layer and Haller’s layer are used to this day, although there are no real anatomic boundaries between them (Fig. 28). In addition, the choriocapillaris layer is often shown as a layer many times thicker than its true anatomic depth.

The long posterior ciliary arteries, generally two, the medial and the lateral according to their relationship with the optic nerve at the point of penetration into the sclera, run anteriorly between the sclera and choroid with anastomotic connections from the anterior choroid. They join the anterior ciliary arteries forming the major arterial circle of the iris. The anterior ciliary arteries travel with the extraocular muscles and perforate the sclera near the limbus to join the major arterial circle of the iris (Hayreh, 1975; Kiel, 2011). The venous drainage from the anterior segment is directed posteriorly into the choroid where blood is collected in venules, which coalesce into collecting channels, or ampullae, of the vortex veins (commonly four in number, but may vary).

The central retinal artery travels in the optic nerve, perforates the sclera and then progressively branches into smaller retinal arterioles. The central retinal vein, derived from retinal venules and vein, leaves the eye with the optic nerve parallel and counter-current to the central retinal artery draining the retinal tissue. From the optic nerve to the extreme periphery, the retinal arterioles provide retinal capillaries which supply the inner two-thirds of the retina, as the outer third is supplied by the choriocapillaris.

**Fig. 29.**
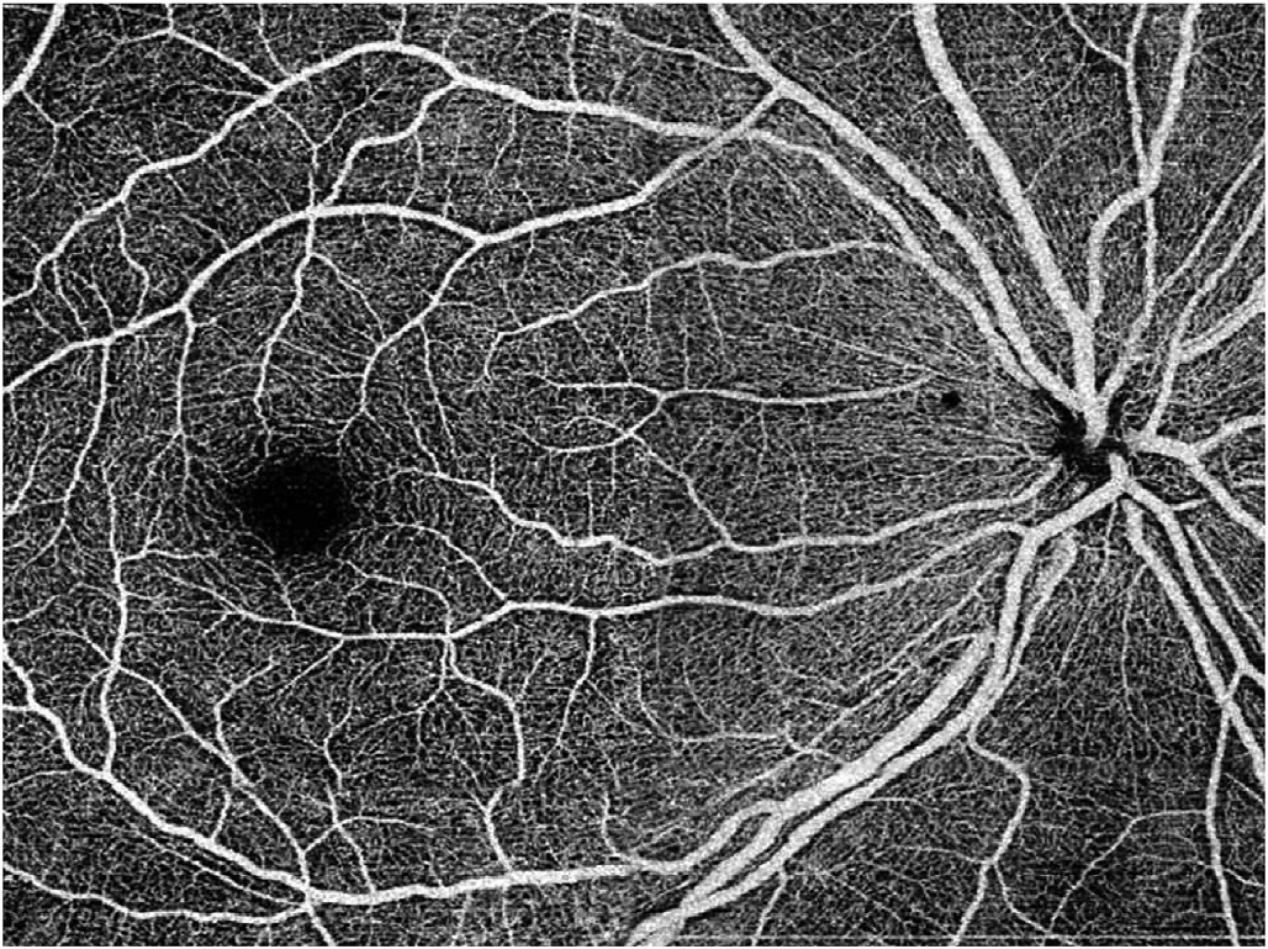
Swept source montage OCT-Angiogram illustrating the superficial capillary circulation of the macular and peripapillary retina. Whereas the central macular circulation is oriented in a more circular fashion around the foveal avascular zone, the superficial peripapillary capillaries have a distinct radial orientation resembling the orientation of the retina nerve fiber layer.

The precise organization of vascular structures must achieve neuronal nutrition without compromising the optical properties of the pathway transmitting light to the photoreceptor layer in the outer retina (Tan et al., 2012). In particular, the foveal avascular zone contains no blood vessels but is surrounded by a continuous network of capillaries. Four morphologically varied retinal vascular networks have been identified. In the nerve fiber layer, long capillary segments are oriented parallel to the direction of retinal ganglion cell axons and is called the radial peripapillary capillary network or plexus (Fig. 29). In the retinal ganglion cell layer, a dense meshwork of vessels is arranged in a lattice pattern with reduced inter-capillary spaces (Chan et al., 2012; Tan et al., 2012). These capillaries are supplied by a branching vascular network starting with the arcade vessels that successively branch in a fractal manner to finally reach the capillaries. In a balanced manner, smaller collecting venules anastomose into larger venules and eventually form the veins in the arcades. This circuit is known as the superficial vascular plexus (SVP) (Fig. 29). Recently the RPCP and SVP taken together have been proposed to be termed the superficial vascular complex (SVC) (Campbell et al., 2017), but the need for this grouping is not apparent. The RPCP and the SVP have different anatomic features as dictated by their respective retinal layers. From the superficial vascular plexus small capillary sized vessels dive deeper into the retinal tissue. These anastomose with capillary-sized vessels forming networks on either side of the inner nuclear layer. These vessels send small draining vessels up through the substance of the retina that then merge with the retinal venules. The two layers are the intermediate and deep capillary plexuses. The anatomy of these layers is not fully delineated, but the intermediate capillary plexus has an irregular shaped loop configuration with vertical and oblique orientation (Tan et al., 2012). In the deep capillary plexus, vessels are arranged in a one-dimensional curvilinear mesh. The distribution of capillary networks within the retina is correlated with the metabolic demands of soma, dendrites and neuronal layers (Kiryu et al., 1995; Yu and Cringle, 2001). The superficial vascular network is the only one with arterioles or larger venules. Because the intermediate and deep plexuses are composed of similarly sized vessels and are separated by only a small distance, they are sometimes grouped together as the deep capillary plexus or deep vascular complex (DVC) (Campbell et al., 2017).

## 10. Vascular layers in the fundus as imaged by OCTA

**Fig. 30.**
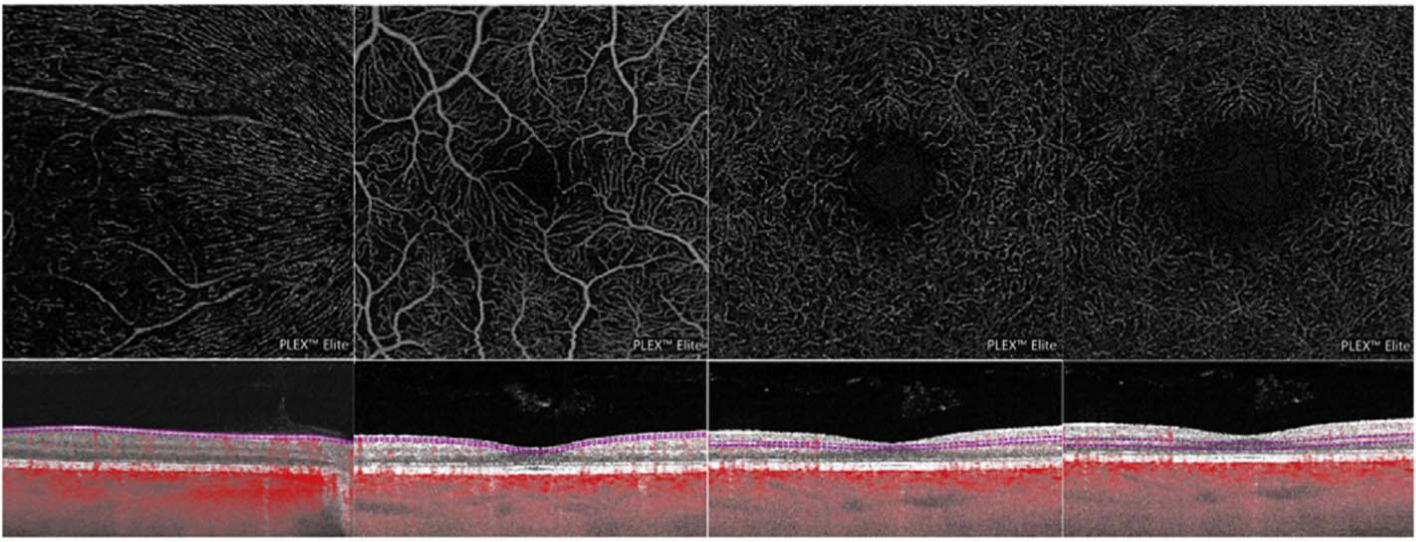
Optical coherence tomography angiography shows four morphologically varied retinal capillary networks along the maculo-papillary axis. A radial peripapillary capillary plexus is located in the nerve fiber layer slab (A). The superficial vascular plexus slab is found in the ganglion cell layer, and is segmented as the inner 80% of the ganglion cell complex, excluding the nerve fiber layer (B). The intermediate capillary plexus is segmented between the outer 20% of the ganglion cell complex to the inner of the inner nuclear layer (C). Finally, the deep capillary plexus is segmented between the outer 50% of the inner nuclear layer and the outer plexiform layer (D).

The high-resolution in the axial direction has allowed the various layers of the retinal and choroidal microcirculation to be selectively evaluated (Choi et al., 2017; Kuehlewein et al., 2015c; Spaide et al., 2015b; Tan et al., 2016c) (Fig. 30). As noted above, the vascular architecture is arranged in 3-dimensions, and to some extent one could produce many “different layers” by selecting various boundaries. In addition, the number of layers that may be visualized is highly dependent on location, as areas of thicker retina (e.g. parafoveal) will, not surprisingly, require more layers of vessels for adequate blood supply (Kuehlewein et al., 2015c). In fact, Snodderly noted that if desired, one could define up to 8 layers of capillaries in the temporal peripapillary retina (Max Snodderly and Weinhaus, 1990). Though there is some degree of arbitrariness in defining the number of retinal capillary layers, a higher density of vessels within certain structural layers of the retina, does lend itself to defining specific vascular layers which may have a benefit of enhancing our understanding of specific diseases as described in subsequent sections (Al-Sheikh et al., 2017). In a normal retina these layers may be segmented for inspection and analysis.

### 10.1. Retinal circulation

Our ability to confidently resolve the microcirculation into specific layers has been somewhat impaired by projection artifact as described in the previous section. The recent introduction of techniques for projection removal, though still needing further validation, have suggested that the macular retinal circulation can be visualized in its principle component parts, the radial peripapillary capillary plexus, the superficial vascular plexus, the intermediate capillary plexus, and the deep capillary plexus (Campbell et al., 2017; Zhang et al., 2016). In the peripapillary region, the radial peripapillary capillary network supplies the unmyelinated nerve fibers there (Snodderly et al., 1992). The metabolic demands are great to maintain electrolyte concentration gradients for the axons in the absence of myelin. As a consequence, the radial peripapillary capillary network is correspondingly dense. These vessels are located around the nervehead and extend for a few millimeters along the arcades. Despite the anatomic location of the radial peripapillary capillary network in the most superficial portions of the retina, which itself is transparent, the radial peripapillary capillary network is not particularly visible during FA. One possible reason is that the nerve fibers may cause so much scattering and diffusion of light the individual capillaries are not visible. The intermediate and deep vascular plexuses have a spider like character (Paula et al., 2010; Weinhaus et al., 1995). The small diameter of the feeding vessels and the loose netting like character of the vessels do not appear to be optimized for oxygen delivery, although that would be at least one expected function to supply the metabolically active retina. Lack of perfusion of these layers has been considered to be a cause or contribute to retinal ischemic diseases (Chen et al., 2015). The intermediate and deep plexuses can be visualized with OCTA, although without projection artifact removal the layers are contaminated by projections from the inner retina and they are hard to separate from each other. In ischemic diseases there is varying opacification and whitening of the retina, compounding problems of both visualization and of projection artifact.

**Fig. 31.**
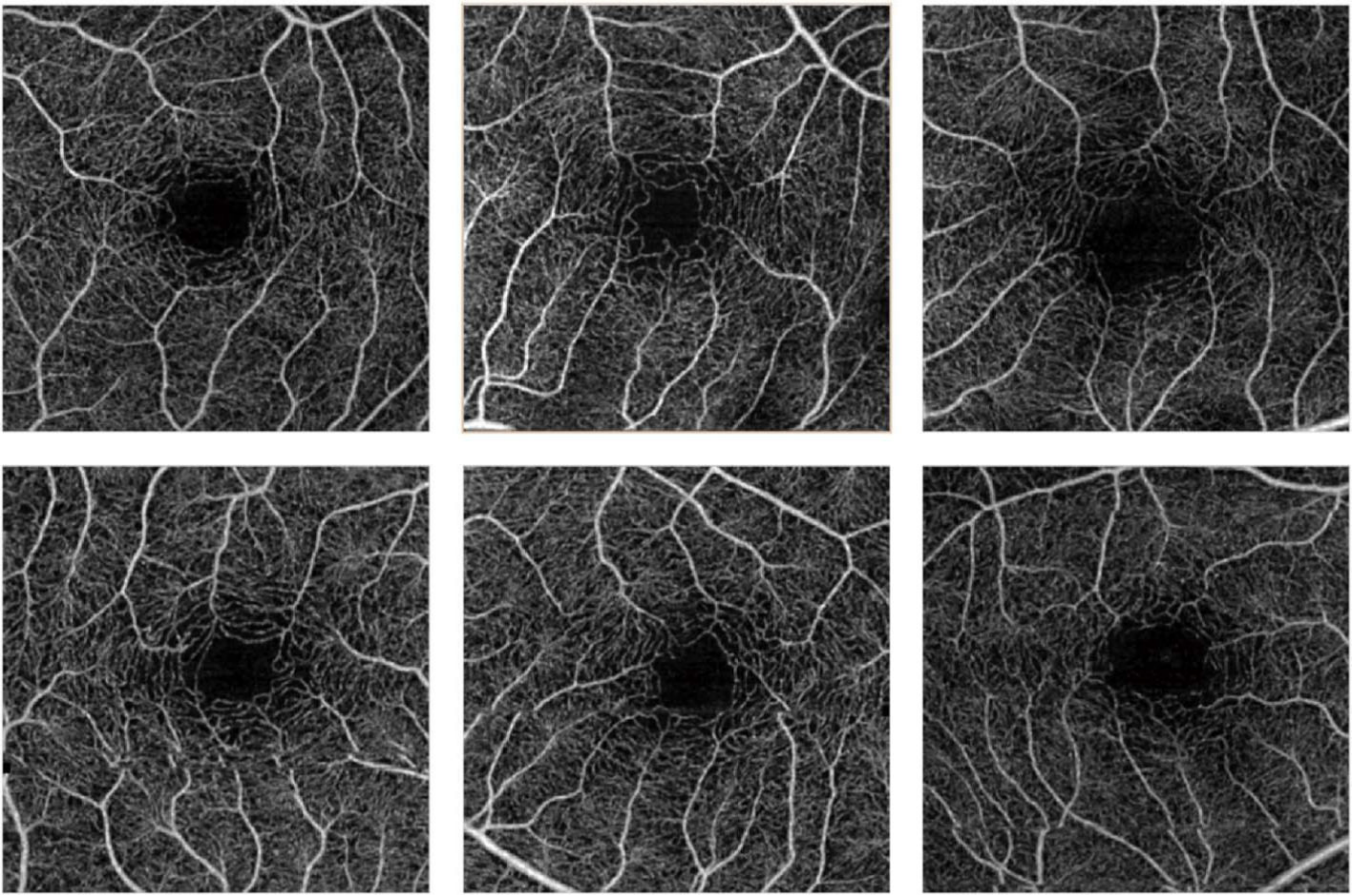
Macular OCT angiograms (full retinal thickness) from one eye of six normal subjects. Note considerable variation in the size and shape of the foveal avascular zone (FAZ). OCT = optical coherence tomography.

The foveal avascular zone (FAZ) represents a region of absent capillaries at the center of the fovea and can show considerable variation in dimension even among normals (Fig. 31). The layers of vessels in the retina terminate as they approach the center of the fovea. The superficial vascular plexus, which invests the ganglion cell layer, terminates slightly further from the center of the fovea than does the deep vascular layer. Polyak considered the perifoveal vascular ring to be part of the deep capillary plexus. At the time of this writing, most commercial OCTA instruments incorrectly segment the vascular layers in the central macula (Spaide and Curcio, 2017). The superficial vascular network is correctly identified, for the most part, outside of the central macula. The superficial vascular plexus terminates near the upper edge of the clivus. Instead most instruments segment a relatively uniform thickness of the inner retina through the central macula, even through the foveal avascular zone. The net effect of this is to include all of the superficial vascular plexus and much of the inner deep plexus together as what would be considered the superficial plexus. A secondary effect of this segmentation is the deep capillary plexus is imaged to start at some arbitrary distance distal to the edge of the perifoveal capillary ring. Improvements in segmentation algorithms will correct this error. Independent of segmentation, OCTA is a great way to image the perifoveal capillary network. It provides a high-resolution method to evaluate the size and character of the perifoveal capillary network in health and disease.

### 10.2. The choroid

#### 10.2.1. Choriocapillaris

Imaging the vessels in the choriocapillaris in vivo using standard modalities is difficult because of light scattering within overlying tissue, particularly the retinal pigment epithelium (RPE). Although the choriocapillaris is composed of relatively large-diameter capillaries, they are interconnected in a densely packed arrangement with very small intercapillary pillars (Fryczkowski, 1994; Singh Hayreh, 1974). The size of the separation is often less than the lateral resolution afforded by dye-based angiography. Fluorescein readily leaks from the choriocapillaris. The flow in the choriocapillaris is fast and because of the prominent leakage vascular structure is obscured. Efforts to slow the flow, such as increasing the intraocular pressure, result in a fluorescein angiogram that shows a lobular pattern to the filling, but the actual vessels in any given lobule are not seen (Singh Hayreh, 1974). Although indocyanine green dye shows less leakage than fluorescein, it still leaks from the choriocapillaris and stains Bruch’s membrane and the choroidal stroma (Chang et al., 1998). Optical coherence tomography (OCT) has high axial resolution, but without adaptive optics, the lateral resolution is insufficient to visualize the choriocapillaris clearly in the posterior pole. While OCTA has comparable lateral resolution to structural OCT, it can detect choriocapillaris blood flow, producing contrast between the RPE and choriocapillaris (Choi et al., 2013a; Motaghiannezam et al., 2012; Sohrab et al., 2012; Spaide et al., 2015b).

**Fig. 32.**
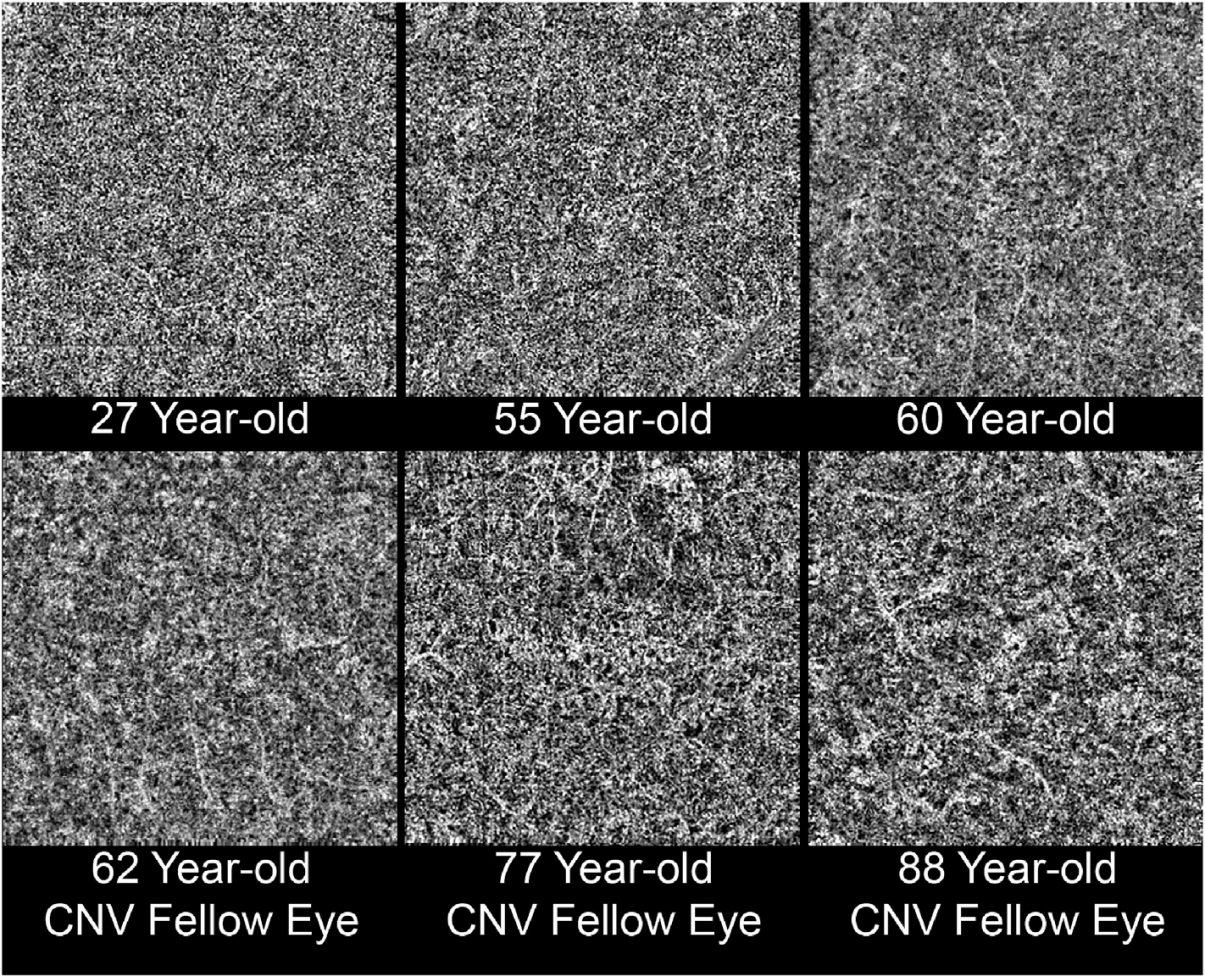
Choriocapillaris images from subjects of varying ages obtained using optical coherence tomography angiography of the central macula. Each image represents an area of 3000 × 3000 μm. The bright areas correspond to high flow signal while the dark regions are called flow voids and represent areas where there is a lack of flow signal. The age range is from 27 years (upper left) to 88 years (lower right). The bottom row of images shows not only those of increasing age, but also with choroidal neovascularization in the fellow eye. Reprinted from Spaide (2016a).

When the inner most thickness of the choroid is imaged with OCTA a granular image is obtained that is suggestive of the choriocapillaris (Fig. 32). Since OCTA has similar resolution to structural OCT, it may seem paradoxical that flow can be detected. In the early days of radio it was common to for enterprising individuals to make crystal radios, which could passively detect radio signals, at the time often originating from a radio station in the local town. The selectivity of the crystal radio was typically limited, which means the ability to differentiate closely spaced stations on the radio frequency spectrum would be impaired. In this case, both stations would be heard, and it would not be possible with the primitive tuning used in early crystal sets to separate them. In an analogous manner even though structural OCT may not be able to separate closely placed capillaries, an OCTA flow signal may still be detected. This signal is used to create an image of the choriocapillaris.

**Fig. 33.**
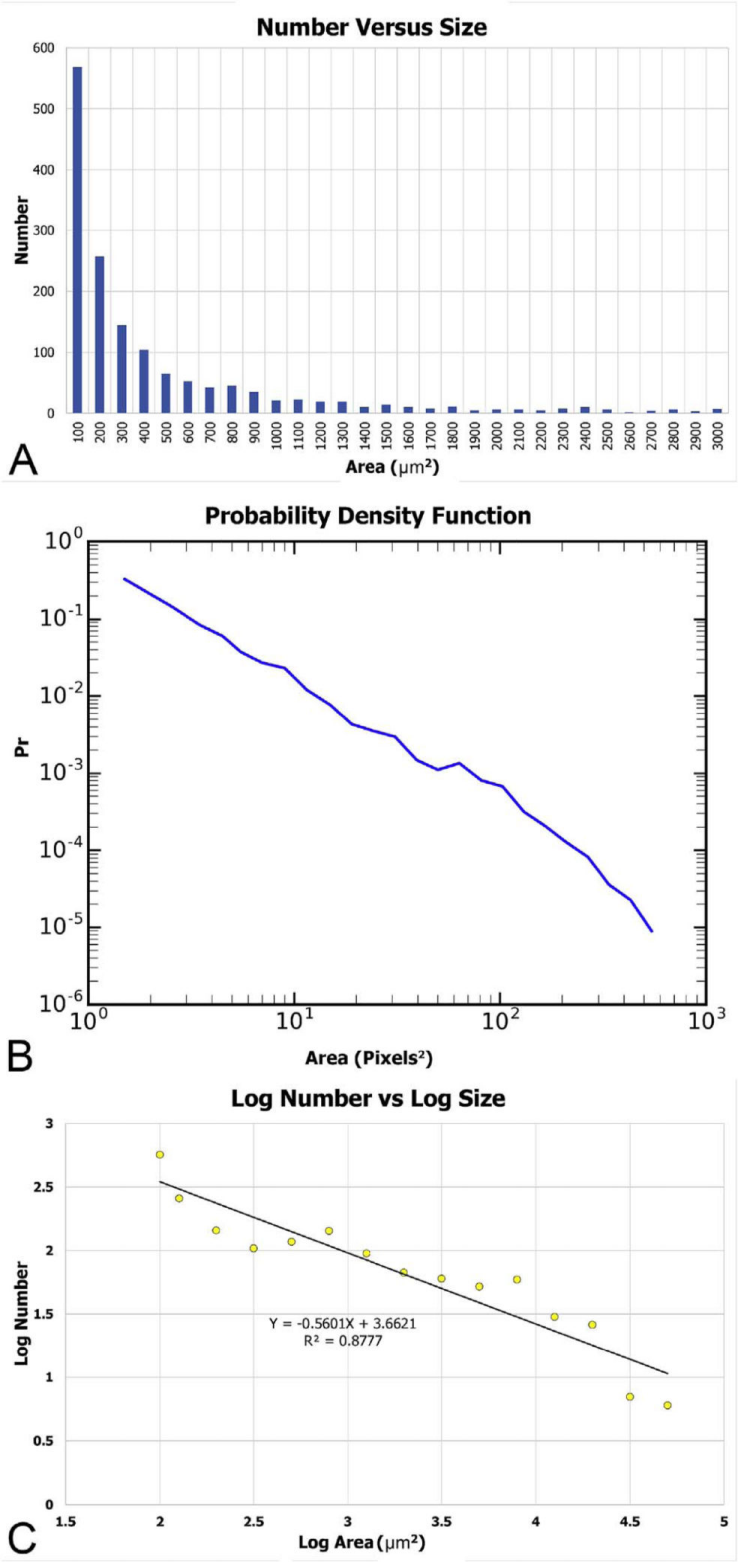
Number and sizes of the thresholded flow voids of a representative eye. (A) The distribution of the number of flow voids versus linearly binned sizes shows a high amount of skew with a long tail. (B) The probability density function shown in a log-log plot is nearly linear, demonstrating the power law characteristics. Because of the finite size of the image and the imaging of deeper vessels that have a looser packing density the actual relationship was hypothesized to be a truncated power law, which was confirmed by maximal likelihood methods. (C) The log number versus log size of the flow voids is shown in a log-log plot with logarithmic binning showing the linear relationship characteristic of a power law. Reprinted from Spaide (2016a).

The resultant image shows a granular pattern of bright and dark areas of varying sizes (Fig. 32). The anatomic structure of the choriocapillaris does not appear to explain the dark areas, since the individual capillaries in the choriocapillaris can’t be seen. For there to be a dark area there has to be a relative decrease in local flow signal, either because of low or absent flow in the small region. As such, these small areas are called signal voids (Spaide, 2015a). The number of signal voids at any given size can be analyzed (Fig. 33). When the number of flow voids is compared to the size, a peculiar relationship is seen. Over log units of range, the log number of voids is seen to be linearly related to the log size of the voids. The fixed slope in the log transform relationship means the data showed similarity across units of scale, known as scale invariance, which is equivalent to stating the process is fractal in nature. This simple relationship, known as a power law distribution has numerous implications in terms of physiology of choriocapillaris flow, potential mechanisms of flow alteration in aging and disease, transitions to late-AMD, and grading of changes associated with disease (Spaide, 2016a). This novel non-invasive approach may have utility in evaluating ocular disease, and possibly may be a method to evaluate systemic microvascular health.

**Fig. 34.**
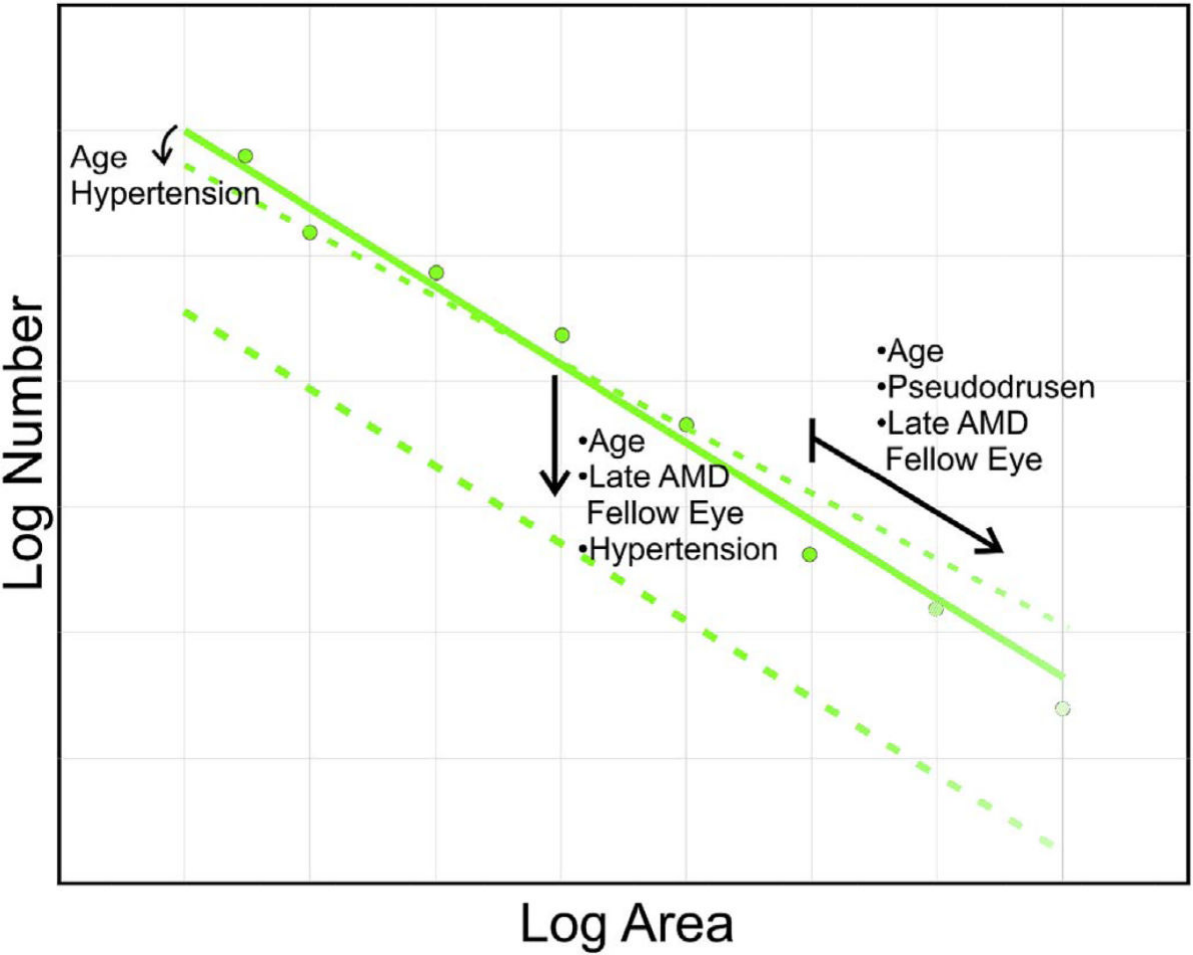
In a log-log plot the data follows a y = mx + b slope intercept relationship and provides parameters that can be evaluated using statistical analysis. The significant predictors of slope were age and hypertension; increasing age and the diagnosis of hypertension were both associated with decreasing slope. The parameter b represents the offset, which is decreased by increasing age, the diagnosis of hypertension, and the presence of late age related macular degeneration (AMD) in the fellow eye. Flow voids having larger regions, as would be shown along the right side of the slope, are more likely to occur in older patients, those with pseudodrusen, and in association with late AMD in the fellow eye. Reprinted from Spaide (2016a).

**Fig. 35.**
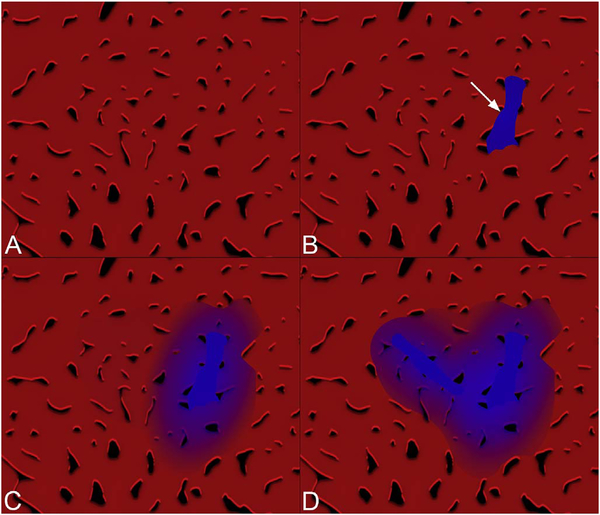
Potential explanation for observed flow patterns in the choriocapillaris. (A) The choriocapillaris is a dense mesh of capillaries as shown in red and in the posterior pole the intercapillary pillars are small (shown in black). (B) The presence of ghost segments (white arrow) occurs with age, but is more common in eyes with drusen and with pseudodrusen. (C) The induced flow abnormality is larger than the segment and is shown in as the blue gradient. (D) To produce a power law relationship, two features need to be present. Additional segments need to become nonfunctional over time and the location of these nonfunctioning segments should have a tendency to occur in the vicinity of an already nonfunctional segment. Reprinted from Spaide (2016a).

Given the skewed (non-normal) nature of the distribution, calculation of mean and standard deviation is not possible. A trend line can be fitted to the data as shown in a log-log plot and this trend line has a simple slope intercept formula log (number) = *m*log (size) + *b*. Using generalized estimating equations the predictors of m were age and hypertension. The predictors of *b* were age, hypertension, and a diagnosis of late AMD in the fellow eye and for large signal voids, age, late AMD in the fellow eye, and the presence of pseudodrusen. A consolidated summary of the findings in terms of a slope intercept schematic is shown in Fig. 34. The flow in any given region of the choriocapillaris plexus would be expected to be decreased if one or more segments are non-functional (Fig. 35). By analogy the traffic in Manhattan is greater in the region around a street closure. For a power law relationship to occur there should be additional regions of capillary flow abnormalities and these need to occur in the region of previous problems. Stated in a more formal way, a Barabási–Albert model scale free networks (that is those that show a power law relationship) is formed if there are additional nodes and the new nodes show preferential attachment to older nodes. In this case a node would represent an area of decreased flow (Barabási and Albert, 1999).

Our understanding of the blood flow in the choroid underwent a large advance in the 1970s because of the seminal work of Sohan Singh Hayreh (Hayreh, 1974, 1975; Singh Hayreh, 1974). He established that choroidal blood flow was segmental in nature with arterioles branching to smaller vessels that supplied discrete units of capillaries in the choriocapillaris, known as lobules. By elevating the intraocular pressure to slow down the flow he could show the filling of the choriocapillaris occurred in quantized units of area corresponding to the anatomic size of the lobules. Thus the choriocapillaris appears to be a uniform meshwork of densely packed and interconnected capillaries when viewed from the retinal side, but functions in a lobular mode in terms of blood flow. The morphology of some disease presentations have been theorized to be influenced by the lobular nature of choriocapillaris flow (de Venecia et al., 1980; Deutman, 1983; Holt et al., 1976).

Histologic evaluation of the choriocapillaris was done by Mullins and coworkers (Mullins et al., 2011). They found evidence of ghost vessels, which essentially are nonfunctional capillary segments, in the choriocapillaris meshwork. The number of ghost vessels was positively correlated with age and the presence of drusen. Curcio and coworkers found there were ghost vessels in older eyes and the proportion of ghost vessels increased in eyes with basal linear deposit, mounds of which appear as drusen, and also in eyes with subretinal drusenoid deposits, which appear clinically as pseudodrusen (Curcio et al., 2013). Biesemeier and colleagues found choriocapillaris loss in histologic section in controls and more extensive loss in eyes with AMD (Biesemeier et al., 2014). A major difficulty is tying in cross-sectional images showing choriocapillary absence with flow through the capillary network. Flow in the ghost vessels is assumed to be absent – the vessels do not appear to be viable, and they contain no blood cells. Blood flow is lacking in the specific ghost vessel segment, but also could be compromised in vessels adjacent to the occluded segment. Thus the area of abnormal flow would be the same size or larger than the affected segment, but not necessarily affecting the whole lobule. The flow abnormalities implied by the signal voids are almost always smaller than the size of a choriocapillaris lobule. There is a wide range of estimates for lobule size in the macular region of humans, but typically range from 400 to 800 microns in diameter (Fryczkowski, 1994; Olver, 1990; Yoneya and Tso, 1987; Yoneya et al., 1983). The signal voids range in size over several log units starting with 100 μm^2^, with only the largest exceeding the size of a choriocapillaris lobule. The skewed distribution of values for the size of the signal voids in the choriocapillaris because of the power law distribution also means simple descriptors such as mean size and standard deviation are not mathematically rational; there is no characteristic size or scale for a scale free system.

Using microspheres and by evaluating tracer washout, myocardial tissue shows features that imply the flow must have a power-law characteristic, although these can’t be directly imaged in vivo (Bassingthwaighte and Beard, 1995; Beard and Bassingthwaighte, 1998, 2000; Kendal, 2001; King et al., 1996). The high axial resolution and high transverse pixel density in OCT images possible make direct observation of the power-law characteristics of the flow voids in the choriocapillaris possible in vivo. The signal voids in the choriocapillaris showed measurable changes in association with age and hypertension. Additional research into choriocapillaris flow characteristics may help evaluate the possibility that the choriocapillaris, the densest layer of capillaries in the body, could serve as a gauge of microvascular abnormalities in the body in general.

Decreases in local choriocapillaris perfusion of the choriocapillaris may put the overlying retinal pigment epithelium at risk, which may lead to further compromise of the choriocapillaris. In dynamic systems self-similarity following a power law characteristic can develop in a robust way from self-organized criticality. This was first modeled using sand falling onto a pile and observing small avalanches of sand (Bak et al., 1988), and later discoveries found power law distributions explained frequency and severity of real avalanches, earthquakes, material failure, transitions in shape memory metals, cancer metastasis, biologic evolution, traffic jam propagation, and epidemic transitions of disease (Bak, 1996; Hüfner and Mukhopadhyay, 1986; LukierskaWalasek and Topolski, 2014; Nagatani, 1998; Newman, 2005; Vives et al., 1994). In a loose analogous sense, late AMD may be viewed as a phase change influenced in part by a positive feedback loop between the power law related flow heterogeneities in choriocapillaris flow and the retinal pigment epithelium.

#### 10.2.2. Sattler’s and Haller’s layers

**Fig. 36.**
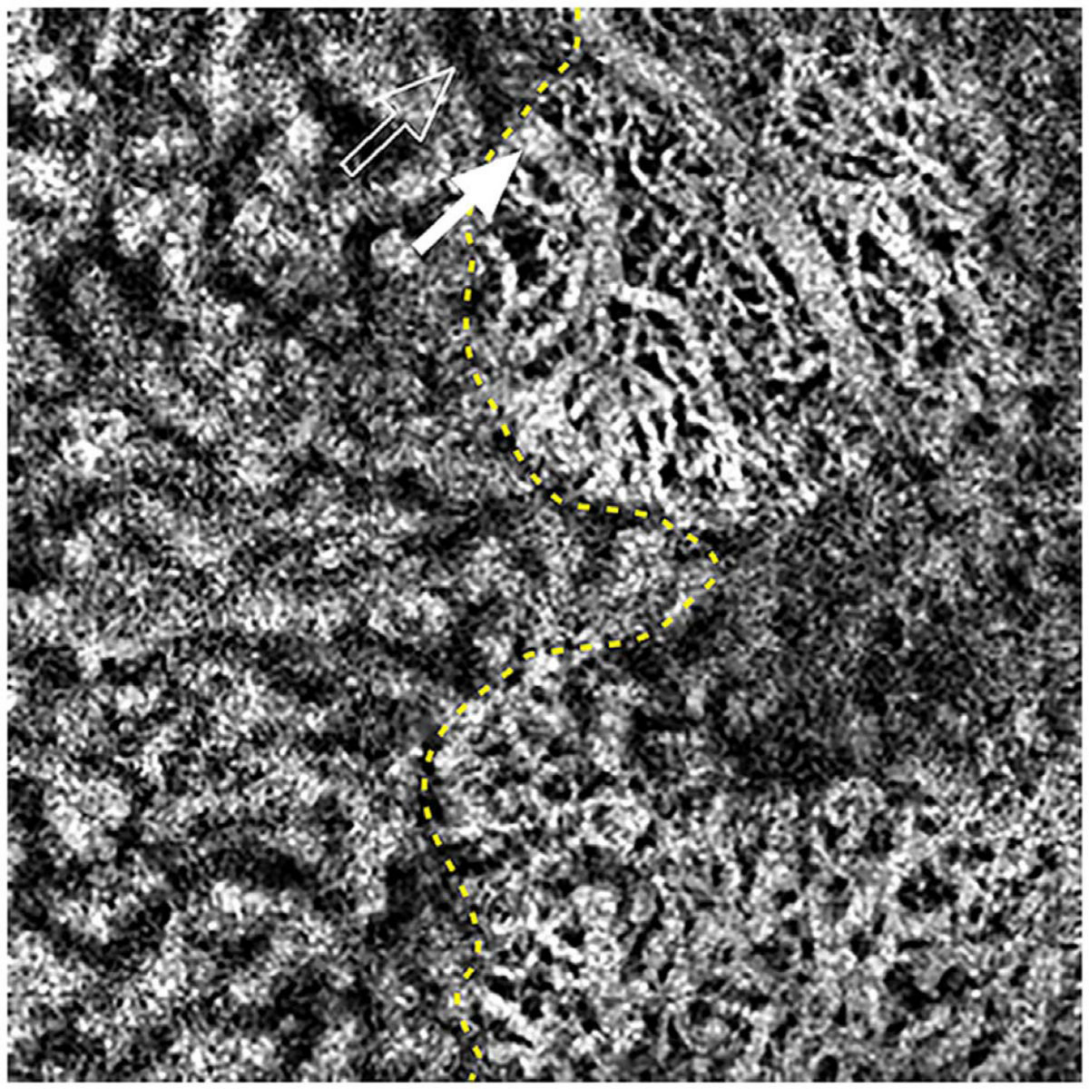
An OCTA image of the submacular choroid obtained in a patient with geographic atrophy. There is atrophy to the right of the dashed yellow line. The larger choroidal vessels show visible flow signal while they do not in the areas with extant retinal pigment epithelium. Note the course of one large choroidal vessel from an area under the RPE (open arrow) to the area of atrophy (white arrow.).

**Fig. 37.**
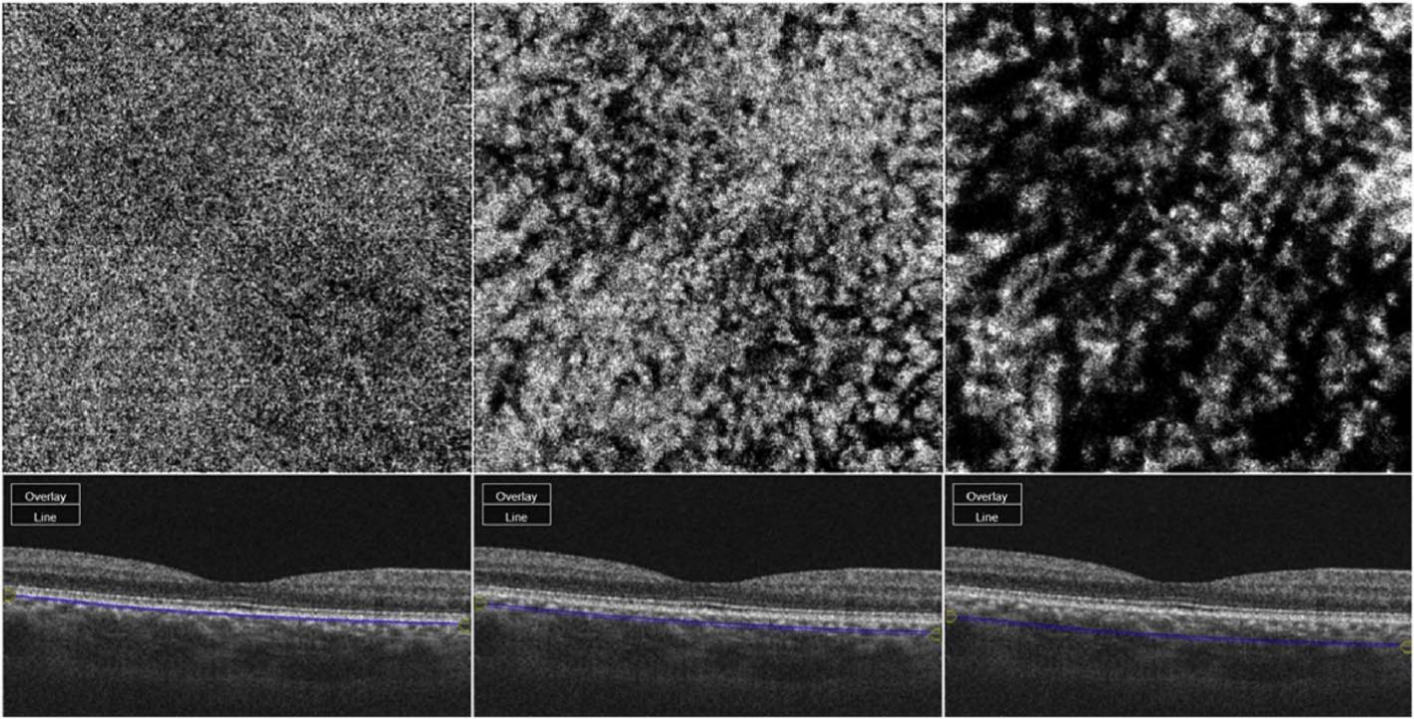
Swept source OCT angiogram (OCTA) of the macular choroid of a normal eye. The upper panels show the slab en face OCTA image through the vascular layer of interest. The lower panels demonstrate the slab (blue-lines) location of the en face image on a corresponding B-scan. The en face OCTA is shown at the level of the choriocaillaris (left panel), presumed Sattler’s layer (middle), and presumed Haller’s layer (right). The choriocapillaris has a granular texture, but the individual capillaries are not seen. Also, it is important to note that Sattler and Haller layers as histologically defined do not have precise borders or a precise axial position relative to the RPE, and hence these slabs represent approximations. The larger choroidal vessels appear dark, presumably due to loss of signal with depth, projection artifact and decorrelation from overlying retinal vessels and choriocapillaris, and image processing or thresholding artifact.

Flow in deeper layers of the choroid is not detected by OCTA, mostly because of scattering by the pigment in the RPE and by the choriocapillaris. The flow in vessels in the deep choroid is visible if there is geographic atrophy (Fig. 36). In some eyes, particularly in those with thin choroids such as high myopes, OCTA can show flow in the medium sized vessels of Sattler’s layer. However, because of signal loss, fringe wash-out, and thresholding used in signal processing, the vessels in Sattler’s layer and certainly in Haller’s layer appear dark in normal eyes (Fig. 37). They may appear bright (showing evidence of flow), however, in areas with overlying RPE loss or depigmentation with absence of the choriocapillaris allowing greater penetration of light into the choroid. As a result, in eyes with high myopia or atrophy, the deeper choroidal vessels are well seen.

Swept source OCT, by offering less sensitivity loss with depth has the theoretical advantage of offering better visualization of the choroid (Ferrara et al., 2016). Even with swept source OCT, flow in the larger vessels cannot be visualized without atrophy of the RPE and choriocapillaris. However, careful comparative studies are still required to best define its advantage in this setting. The method of OCTA acquisition may also impact the quality of the choroidal imaging. Gorczynska et al. compared amplitude-decorrelation (AD) and phase-variance (PV) based methods for imaging the retinal and choroidal circulations (Gorczynska et al., 2016). They observed better signal strength in the choroid with the PV-based methods but also more overlying retinal vessel projection artifact. However, visualization was relatively similar between methods.

## 11. Optical coherence tomography angiography in ocular diseases

### 11.1. Diabetic retinopathy

Diabetic retinopathy is a disease that has profound effects on the retinal vasculature (Antonetti et al., 2012), is rapidly becoming more prevalent world-wide, and, as such, is a disease for which OCT angiography may be profoundly useful. The current gold standard for assessing the severity of diabetic retinopathy consists of discrete stages and relies on comparing the numbers of various vascular features with standard reference photographs. This grading system was shown to correlate with the risk of disease progression (Wilkinson et al., 2003). Clinical grading of retinopathy in large epidemiologic was dependent primarily on vascular features seen in color photographs. Clinical evaluation traditionally used FA as well (Panel, 2003). Many features of diabetic retinopathy have the potential to be imaged and more accurately classified with OCTA. In addition by showing us various retinal vascular layers, OCTA offers the opportunity for greater understanding of disease processes.

Clinical features of non-proliferative diabetic retinopathy (NPDR) include the presence of microaneurysms, intraretinal hemorrhages, intraretinal microvascular abnormalities, venous beading, cotton wool spots, hard exudates and neovascularization (Group, 1991c). Of these, the presence of cotton wool spots and hard exudates have not been associated with an increased risk of progression to PDR and therefore NPDR is stratified into mild, moderate and severe based on the presence and severity of the first 4 features. On the other hand, proliferative diabetic retinopathy is characterized by the presence of neovascularization of the retina or the iris (Group, 1991c).

In addition to the clinical features, angiographic features using FA are enlargement and irregularity of the foveal avascular zone (FAZ), microaneurysms, capillary loss, capillary dilation, intraretinal microvascular abnormalities, focal narrowing and pruning of arterioles, and retinal neovascularization. FA is also useful in the setting of diabetic macular edema and may show leaking microaneurysms, diffuse capillary leakage, and capillary nonperfusion associated with the areas of retinal thickening (Group, 1991a, b). FA is also invaluable clinically for the evaluation of macular ischemia.

#### 11.1.1. Optical coherence tomography angiography

**Fig. 38.**
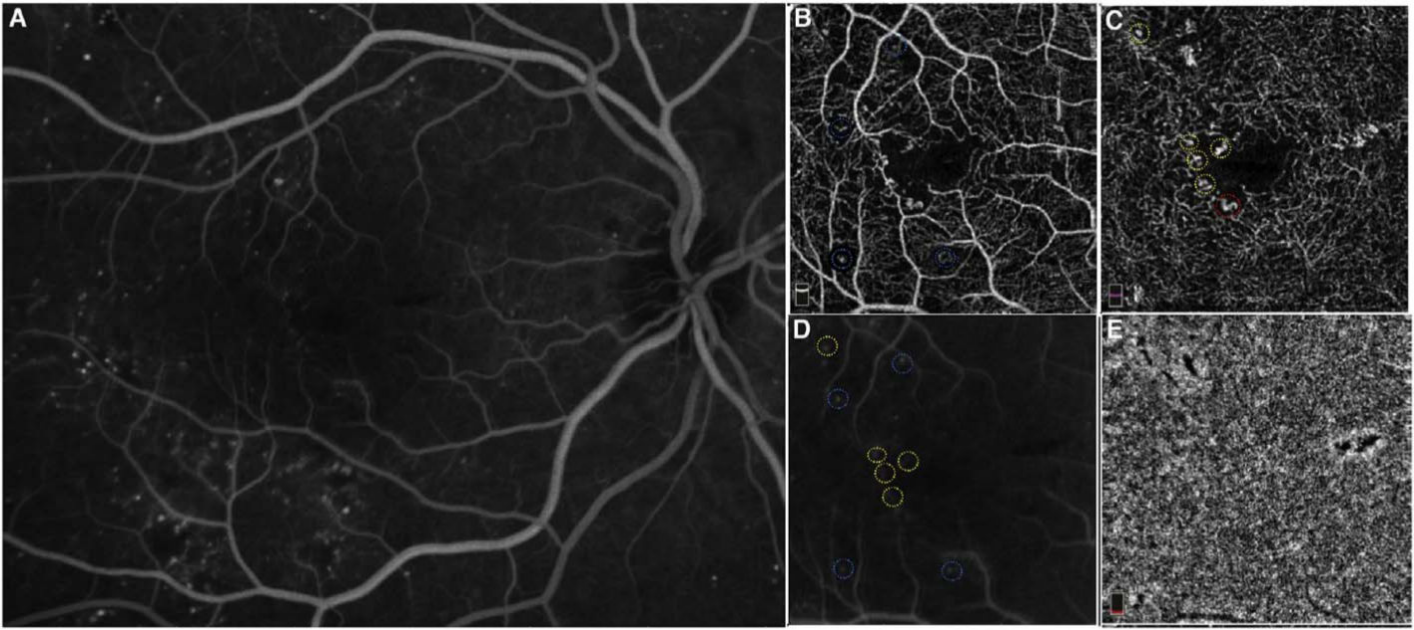
Fluorescein angiography (FA) of a diabetic eye (A) and the corresponding OCTA image segmented at the superficial retinal capillary plexus (B) and the deep capillary plexus (C). Note the microvascular changes seen on the OCTA including microaneurysms, vascular dropout and a ragged appearance of the foveal avascular zone. Figure D is the FA cropped to a 3 × 3mm size to correspond to the OCTA image. Blue circles show microaneurysms visualized better on the superficial capillary layer of the OCTA and yellow circles show some examples of microaneurysms visualized better on the deep retinal capillary layer of the OCTA. The red circle demonstrates a microvascular abnormality that spans both the superficial and the deep plexi. Note that there are some microaneurysms that are seen on the FA but not seen on the OCTA. Figure E is a corresponding image of the choriocapillaris showing areas of choriocapillaris dropout.

One of the major limitations of FA is that light scattering in the retina renders it unable to capture the alterations in the deep capillary plexus of the retina, which, per histopathologic studies, is the site of a majority of the changes that happen especially early on in diabetic retinopathy (Moore et al., 1999). Besides the other obvious advantages of OCT angiography (patient comfort, lower patient risk, shorter acquisition time), OCT angiography is able to penetrate into the retinal layers and image the various different capillary plexuses, thereby providing us the unique ability to reconstruct and view the retinal vasculature in a 3-dimensional fashion, as well as to visualize in isolation the individual retinal plexuses. This may be critical in diabetes, since this enables us to evaluate independently the changes that occur in the superficial, intermediate, and deep capillary plexuses of the retina. Individual visualization of the retinal plexuses allows for better identification of the changes associated with diabetic retinopathy such as capillary dropout and enlargement of the foveal avascular zone. In the future it may allow for a better understanding of disease pathogenesis and progression as well as a better way to screen for and follow diabetic vascular changes (Fig. 38) (Choi et al., 2017; Salz et al., 2016).

**Fig. 39.**
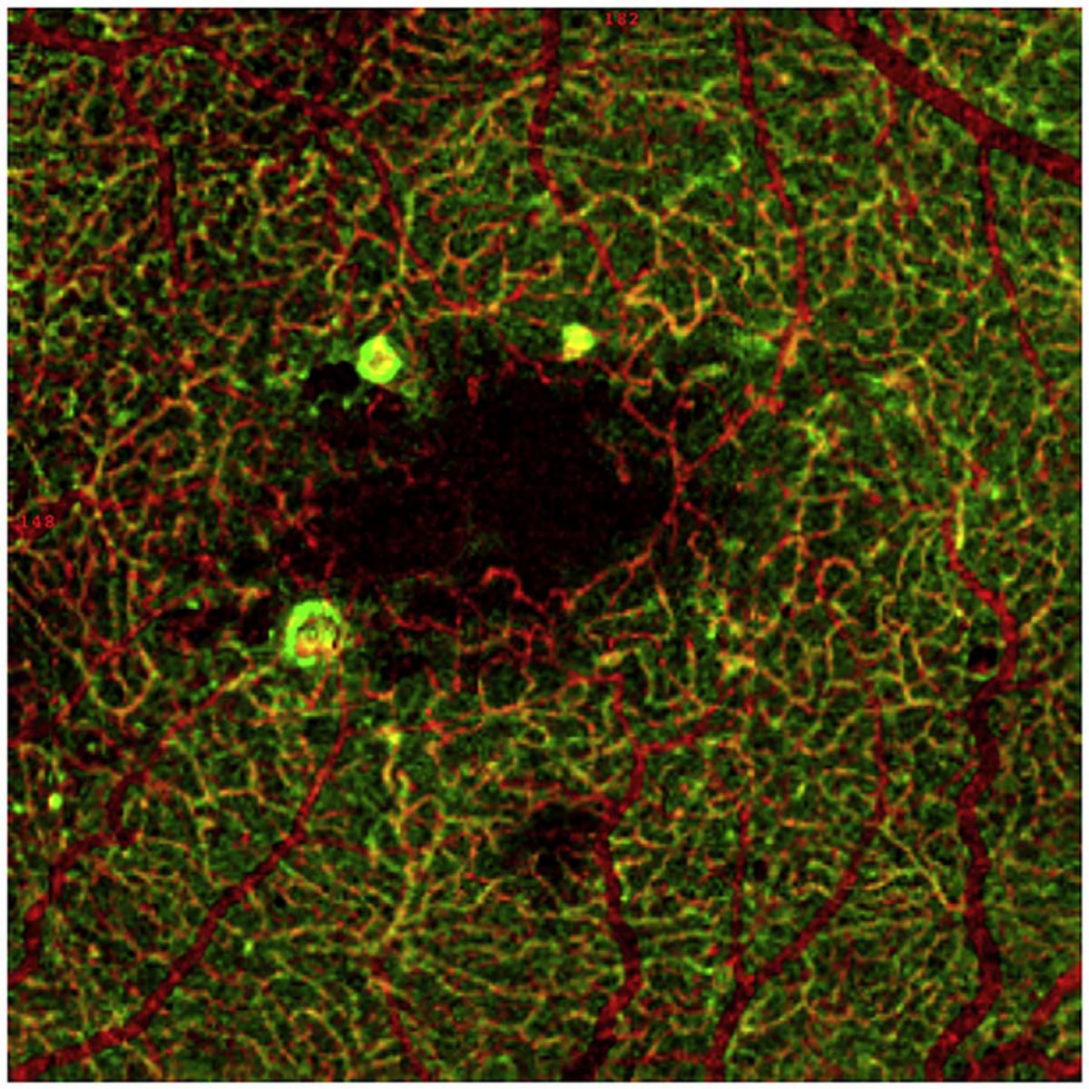
This is an overlay of the OCTA of diabetic microaneurysms with the size of the microaneurysm as measured on the structural OCT scan. The superficial vascular plexus flow is shown in red while the structural OCT information is shown in green. Signal common to both is yellow. Note the microaneurysms visible centrally have yellow cores with a relatively thick wall. Microaneurysms may have thrombi and regions of slow flow that may be below the detection threshold of OCTA.

Microaneurysms that are seen as focal dilations of the retinal capillaries on FA are a hallmark of the disease and can be visualized on OCTA as small focal dilations of the vessels (Fig. 38) (Ishibazawa et al., 2015; Jia et al., 2015; Matsunaga et al., 2015). However, studies show that only around half of the microaneurysms seen on FA are visualized on OCTA (Ishibazawa et al., 2015; Miwa et al., 2016; Salz et al., 2016). This is thought to be because of the inability of OCTA to measure slow flow. This is demonstrated in Fig. 39 which is an overlay of the ‘flow area’ of microaneurysms as seen on OCTA to true area as visualized on structural OCT scans. Imaging very slow flow may be limited by bulk eye motion (Moult et al., 2016). In addition, some of the volume of a microaneurysm may be occupied by thrombus, which has no flow. Microaneurysms that appear hyporeflective in structural OCT are more likely to be not visualized by OCTA (Parravano et al., 2017). Similarly, other vascular abnormalities such as areas of capillary dropout, tortuosity and enlargement of the foveal avascular zone are visualized in better detail and with greater resolution than on FA (Ishibazawa et al., 2015; Miwa et al., 2016). Pre-retinal neovascularization can be seen as vessels projecting into the hyaloid cavity and intraretinal microvascular abnormalities are seen as abnormal vessels still within the retinal plane (Figs. 34 and 40).

#### 11.1.2. Vascular perfusion

**Fig. 41.**
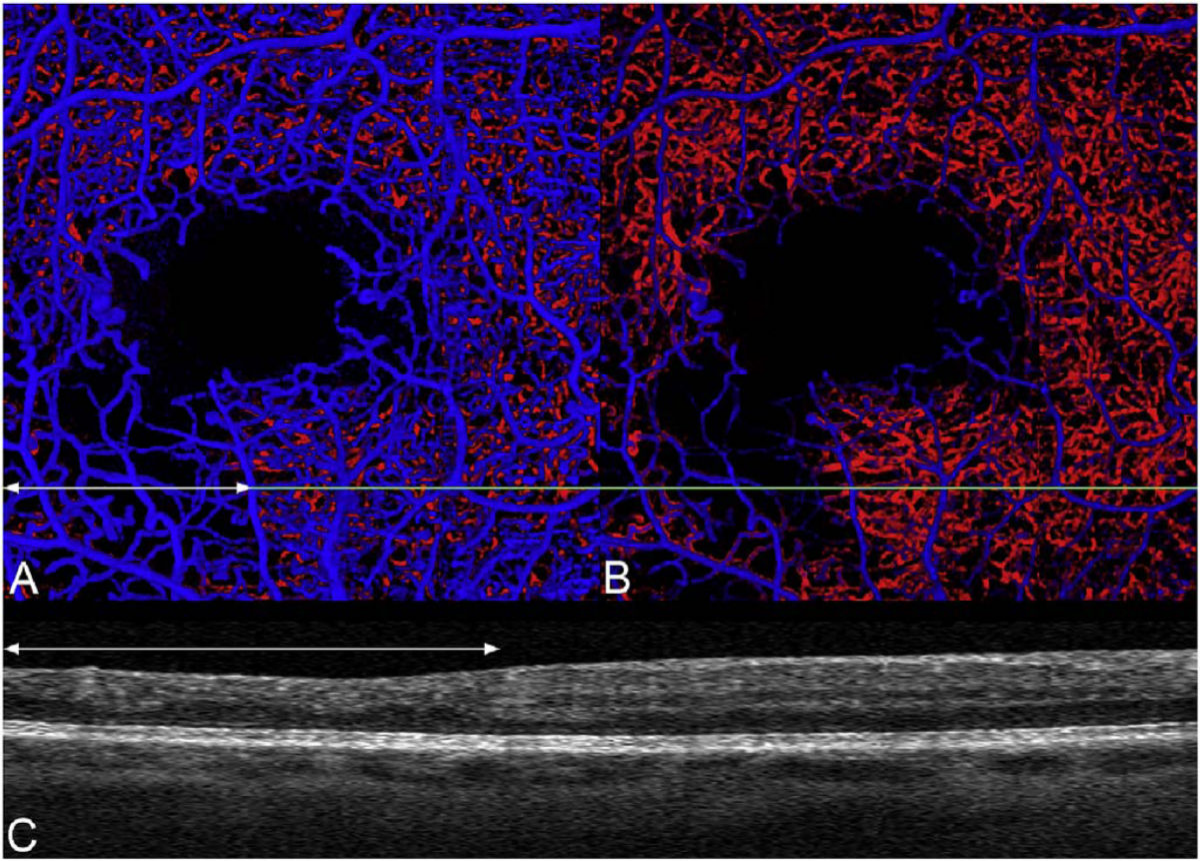
Volume rendered optical coherence tomography angiography of the right eye with areas of absent flow signal. (A) There is attenuation of vascular flow density in the inferior temporal macula of the inner vascular plexus. The double white arrow shows the extent of absence of underlying flow signal from the underlying deep plexus. (B) The opacity of the inner vascular layer was reduced to allow visualization of the deep plexus. Note the absence of flow signal inferotemporally. (C) The structural optical coherence tomography image used to derive the flow data. The double white arrow is scaled to show the same lateral extent as the double white arrow in the picture in the upper left. There is absence of laminar detail associated with retinal thinning, which is consistent with the entity disorganization of retinal inner layers (DRIL). Note the inner nuclear layer is not clearly visible and as such more than the inner retinal layers are involved. Reprinted from Spaide (2015c).

The advent of OCTA and the ability to study the 3-dimensional vascular changes in vivo provides us with important insights into the pathogenesis of diabetic retinopathy and allows for in-vivo visualization of changes that were previously only seen in histopathologic specimens. For example, Scarinci et al. reported that capillary dropout in the deep capillary plexus imaged by OCTA is associated with macular photoreceptor disruption on SDOCT in patients with diabetic retinopathy (Scarinci et al., 2016), as was found years earlier by histopathology (Tso, 1982). OCTA has confirmed the histopathologic observation (Moore et al., 1999) that many of the vascular changes in diabetics, such as the development of microaneurysms, happen sooner and are more severe in the deep capillary plexus than in the superficial capillary plexus and that there may possibly be an even more pronounced increase in the number of microaneurysms in the deep plexus of patients with diabetic macular edema (DME) (Hasegawa et al., 2016). DME may be associated with a profusion of microaneurysms especially in the deep retinal plexus, and it has also been associated with loss of vascular perfusion in the area of the edema that does not usually recover with resolution of the edema (Bradley et al., 2016; Spaide, 2015c). While confirming the heterogeneity of the size of the foveal avascular zone (FAZ), OCTA has shown us that, on average, the size of the FAZ tends to increase with increasing severity of diabetic retinopathy (Di et al., 2016; Jia et al., 2014; Kim et al., 2012; Schwartz et al., 2014; Takase et al., 2015) confirming studies done with FA decades earlier (Bresnick et al., 1984). Automated vascular density algorithms developed for OCTA demonstrate a decrease in the vascular density in both the superficial and deep retinal plexuses of patients with diabetic retinopathy (Agemy et al., 2015; de Carlo et al., 2015b; Hwang et al., 2016). There is an alteration in the pattern of vessels in the deep plexus (Bonnin et al., 2015; Couturier et al., 2015). These reductions in the deep plexus, in particular, have been shown to correlate with disorganization of the retinal inner layers (DRIL) which is associated with decreased visual function (Spaide, 2015c) (Fig. 41). In addition, cystoid macular edema seems to preferentially occur in regions with absent deep plexus flow (Mané et al., 2016; Spaide, 2015c). Moreover, these microvascular alterations in diabetic retinopathy can begin very early. OCT angiography is able to detect vascular changes in patients with diabetes but without clinically detectable diabetic retinopathy (de Carlo et al., 2015b; Dimitrova et al., 2017). It has also been demonstrated that vascular dropout in the choriocapillaris of diabetics, previously described on histopathologic sections (Farkas et al., 1970; Fryczkowski, 1987), can also be observed on OCTA (Fig. 38) (Choi et al., 2017).

Another advantage of obtaining a reliable, reproducible and repeatable vascular map is that image processing techniques can then be applied to get quantitative parameters of the vasculature in diabetics. Vascular density maps initially developed for adaptive optics imaging have been applied to patients with diabetic retinopathy and have been shown to correlate to the severity of diabetic retinopathy (Agemy et al., 2015). Other approaches have also been investigated including intercapillary area as well as investigation of the branching patterns of the capillary network (Cheung et al., 2009). The intercapillary area in diabetics shows increased heterogeneity with increasing levels of diabetic retinopathy (Schottenhamml et al., 2016). Ultimately, these techniques may provide an objective, automated and quantifiable measure of the progression of retinopathy over time and may be useful adjuncts in the clinical and clinical trial setting.

One of the challenges in the current treatment paradigms of diabetic macular edema is that treatment is often initiated at a time when vascular damage has already occurred. Treatments may reverse some but not all of the vision loss, and may not reverse many of the vascular changes that are seen to occur in diabetic macular edema. The ability to visualize vascular changes and to perhaps predict which of these changes will correlate with vision loss may enable us to intervene earlier with disease modifying agents to try to prevent irreversible damage to the retina.

#### 11.1.3. Diabetic macular edema

**Fig. 42.**
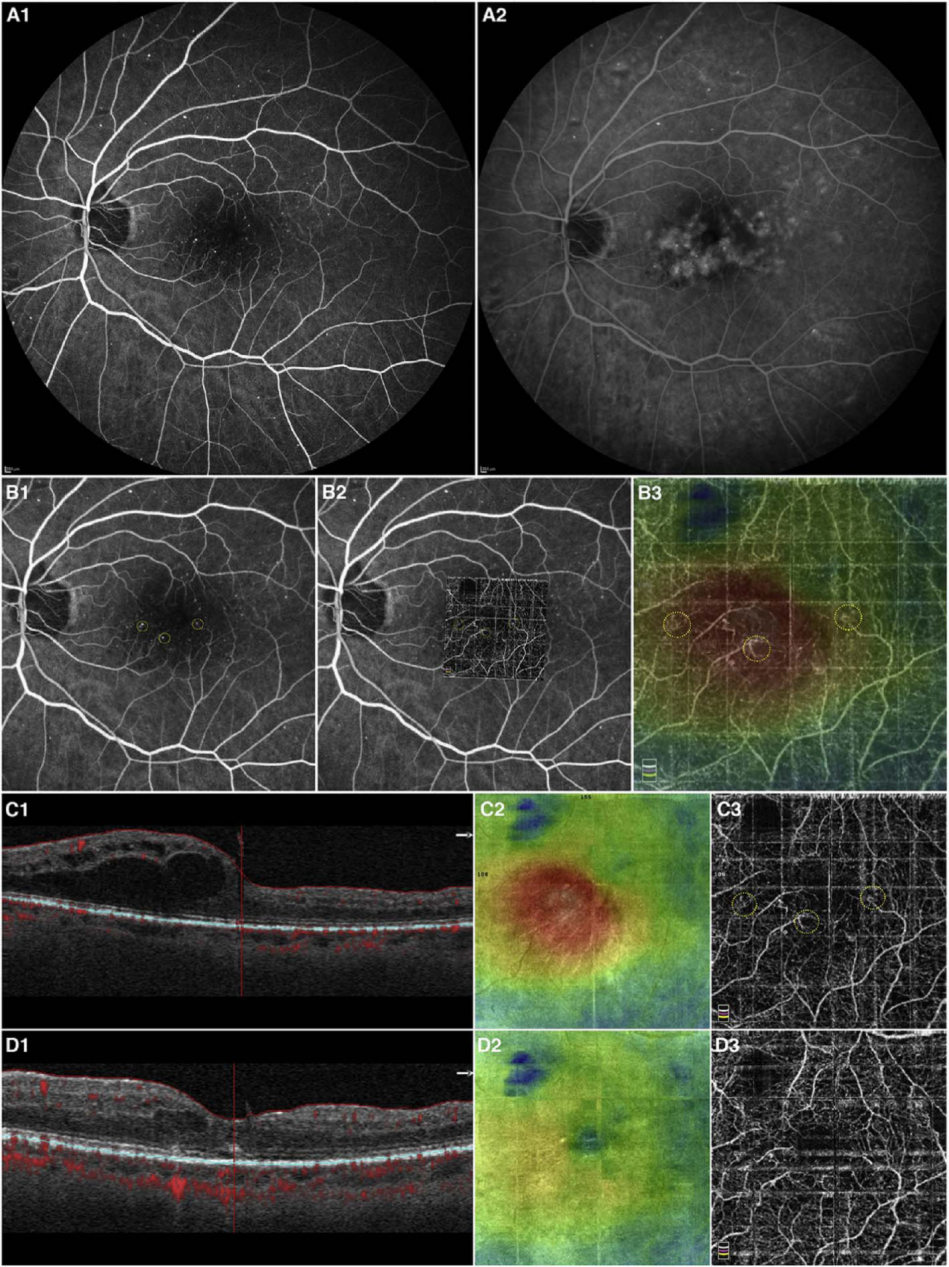
Images of a patient with diabetic macular edema. (A) FA early frame (A1) and late frame (A2) showing that some microaneurysms are leaking while others are not. This is somewhat difficult to identify because of previous focal laser. B1 is a magnified FA image. The 3 mm × 3 mm OCT angiogram is overlaid on the FA image (B2) to show which microaneurysms on the OCT angiogram correspond to those on the FA. Note that not all microaneurysms seen on the FA are seen on the OCTA. (B3) The OCT thickness map is superimposed over the OCTA image to show that there is thickening due to leakage at and around some microaneurysms, while the non-leaking microaneurysms do not show any fluid accumulation. (C1-C3) OCT b-scan, OCT thickness map and OCTA image from the same patient visit in A-B showing edema. (D1–3) OCT b-scan, thickness map and OCTA from the same patient in A-B after intravitreal injections of anti-vascular endothelial growth factor agents. The edema has improved.

One of the limitations of OCTA is its inability to detect leakage and therefore its inability to differentiate between leaking and non-leaking microaneurysms in diabetic macular edema (DME). A second limitation of en face imaging is that segmentation defects are common in edematous maculas. The diagnosis of DME is typically determined by structural OCT, reducing the need to image leakage, per se. Treatment of DME currently relies primarily on intravitreal agents and not as much on focal laser (Panel, 2003; Virgili et al., 2017; Wells et al., 2016), detection of leaking microaneurysms has become of less critical importance. The OCTA images are intrinsically co-registered with structural OCT, so specific microaneurysms can be registered with areas of cystoid change and edema, thereby clearly showing the relationship of the edema and the cystoid spaces to the microvascular alterations, without the obfuscation in detail that may occur as a result of leakage in FA (Hasegawa et al., 2016) (Fig. 42). The response of these microaneurysms to treatment can also be followed over time (Falavarjani et al., 2017b).

OCTA can also be invaluable in the evaluation of patients with proliferative diabetic retinopathy (Choi et al., 2017; de Carlo et al., 2016; Ishibazawa et al., 2016; Savastano et al., 2016; Stanga et al., 2016). Retinal neovascularization is seen to project into the vitreous cavity. This distinguishes it from intraretinal microvascular abnormalities, which is intraretinal and does not extend past the posterior hyaloid. Areas of neovascularization can be precisely quantified on OCTA, since the margins are not obfuscated by leakage as happens on FA. Moreover, these areas of NV are seen to decrease in size or regress on treatment with laser and anti-vascular endothelial growth factor (VEGF) agents. These can subsequently be followed up and serve as markers of VEGF levels in the vitreous, guiding re-treatment decisions.

A study using VISTA on OCTA to study blood flow speeds in the retinal vasculature of patients with diabetes found relatively slower blood flow in microaneurysms and neovascularization (Moult et al., 2016). Studies on patients with diabetic retinopathy show slow and turbulent flow in areas of microaneurysms and vascular pruning, compared to the speed of flow in similar sized vessels without these alterations (Ishibazawa et al., 2016). Moreover, OCT based Doppler techniques suggest a reduction in total retinal blood flow in patients with diabetic macular edema, which is consistent with the extensive peripheral non-perfusion that is often seen on wide field imaging in these patients (Lee et al., 2017; Sadda et al., 2017a).

Also intriguing is correlating the OCTA features in diabetic retinopathy with systemic control. Some early studies have suggested that worse diabetic control may be associated with worse OCTA features (Bhanushali et al., 2016). However, larger studies controlled for multiple confounding factors such as duration of the diabetes and other systemic risk factors are needed to better understand this relationship and to better define the potential role of OCTA in systemic screening. In the future, application of ‘big data’ techniques to diabetic eye disease may be able to identify risk factors not only for diabetic retinopathy progression but also, possibly, for systemic morbidity in these patients.

OCTA has a relatively small field of view compared to FA, which can make it unsuitable for assessing areas of non-perfusion or peripheral neovascularization in diabetic patients. It is theoretically possible to image patients all the way to the mid-periphery using image montaging on OCTA. Wide field imaging has been achieved on commercial OCTA devices using strategies such as montaging multiple images. This becomes clinically practicable with the integration of eye tracking into OCTA machines (Camino et al., 2016; Coscas et al., 2016b; Lauermann et al., 2017; Rosenfeld et al., 2016; Zhang et al., 2015b). Moreover, with the introduction of the higher speed OCTA machines, larger scanning areas such as 12 mm × 12 mm still give reasonable quality images and allow for evaluation of larger areas. On currently available devices, the field of view is still very much less than it is with ultra-wide field FA (Nagiel et al., 2016; Wessel et al., 2012).

#### 11.1.4. Clinical trials

OCTA is currently being integrated into clinical trials of patients with diabetic retinopathy. While it provides important information on diabetic retinopathy progression, integration into clinical trials and use in a reading center faces some challenges that will need to be overcome before OCTA can be integrated in wide scale clinical trials. Because the various different commercial devices use different algorithms to compute OCTA images, and because there are various post-processing steps employed after the analysis, comparative studies are needed to evaluate whether quantifiable metrics applied to the vasculature on OCTA are comparable between devices. Segmentation challenges in patients with altered retinal anatomy, such as in diabetic macular edema, also make standardized evaluation and quantification challenging. However, being cognizant of these challenges allows for the use of strategies that achieve acceptably reproducible and reliable use of OCTA in the clinical trial and reading center setting. For example, a study that assesses alterations in vessel density over time may allow patients to only followup on the same device every time.

OCT angiography provides us with the exciting ability to correlate structural and retinal vascular information and to better understand the role of the different retinal vascular plexuses the evolution of diabetic retinopathy and diabetic macular edema. In the research and drug development setting, this is a powerful tool, particularly since anti-angiogenic therapies have been shown to have an effect on the underlying background retinopathy. As OCTA lends itself to quantification of perfusion through vascular density maps, and the potential for feature identification such as identifying microaneurysms or specified regions of non-perfusion, there may be opportunities to more precisely describe the evolution of the retinopathy. To that end, while OCTA has been utilized in cross sectional studies, its true power will be unleashed when it is integrated into longitudinal studies. In this setting, it has the potential to identify markers that predict worsening in patients with diabetic eye disease. In a clinical setting, OCTA can be used to follow patients with diabetic macular edema or ischemia over time, and provides a rapid, non-invasive way to repetitively monitor evolution of vascular changes.

### 11.2. Retinal vein occlusion

In veno-occlusive disease there is an impediment to venous outflow leading to decreased perfusion of and increased back pressure in the retinal circulation. It is the second most common retinal vascular disease. A vein occlusion may occur at any level of the vascular circuit in the eye. Blockage of the main trunk leaving the eye creates a central retinal vein occlusion, while blockage of a branch of the vein produces, appropriately enough a branch retinal vein occlusion. A hemi-central retinal vein occlusion is caused by the blockage of primary superior or inferior branches of the retinal vein, and produces altitudinal effects. The ophthalmoscopic signs are the familiar venous dilation, tortuosity, intraretinal hemorrhage, retinal edema and hemorrhage in the distribution of the occluded vein. Central retinal vein occlusions were analyzed for gross measures of perfusion and divided into “ischemic” and non-ischemic” forms (Group, 1995, 1997). Since then it was determined that many of the manifestations of veno-occlusive disease could be emulated by increased intraocular VEGF levels (Tolentino et al., 1996). Blocking the increased VEGF agents with pharmacologic agents reduced the disease manifestations. As such an ocular disease caused by a vascular occlusion that causes increased VEGF levels could hardly be called “non-ischemic”. Treatment with intravitreal anti-VEGF agents or corticosteroids causes a reduction or resolution of the edema (Campochiaro et al., 2011; Ip et al., 2009; Scott et al., 2017). This edema typically returns as the intraocular drug concentration decreases. The cystoid spaces recur in the same locations as they once were (Spaide, 2016c).

Color fundus photography shows a blood and thunder appearance of edema and multiple intraretinal hemorrhages with dilated tortuous veins. There is often perivenular retinal whitening (Browning, 2002). FA shows delayed filling in the distribution of the involved retinal vessels. The veins are dilated and tortuous. There is leakage from capillaries during the fluorescein angiogram with dye accumulating in the substance of the retina or within cystoid spaces. OCT shows thickening of the retina with accumulation of fluid in cystoid spaces. There are variable amounts of retinal vascular non-perfusion present. In a branch or hemi-central retinal vein occlusion hemorrhages often persist at the border of the perfused and non-perfused regions. If there is extensive retinal nonperfusion it is possible that retinal neovascularization can be seen as well. Treatment with anti-VEGF agents causes a reduction in the amount of leakage and also regression in the retinal neovascularization as well.

Optical coherence tomography shows dilated venous segments within edematous tissue. Collateral vessels can be seen and these vessels may meander through more than one layer (Muraoka et al., 2014). There may be cystoid spaces present. Some patients with veno-occlusive disease have large amounts of edema, which can be associated with serous detachment of the retina. There may also be loss of the ellipsoid zone. Treatment with anti-VEGF agents causes reduction in the edema leading to a reduction in retinal thickness. After resolution of edema there may be abnormally thin inner retinal layers, reflecting the ischemic loss of tissue there (Ford et al., 2014; Ho et al., 2016; Patel et al., 2016; Yeh et al., 2015).

#### 11.2.1. Optical coherence tomography angiography

Optical coherence tomography highlights the dilated tortuous venous segments. The microvascular complications may be obscured by intraretinal hemorrhages. Areas of vascular non-perfusion are detectable using OCTA. The difficulty with en face imaging is that selective engorgement of retinal layers occurs and these layers are typically not segmented correctly. En face imaging typically only shows part of any one layer and since the segmentation is not correct, manually expanding the depth of the segmentation often includes regionally varying amounts of alternate vascular layers. The resultant en face vascular images may not show accurate representations of the actual flow characteristics in the tissue. The authors of some publications did not appear to consider that incorrect segmentation could occur (Suzuki et al., 2016). However, the findings obtained by conventional methods of examination can also be imaged using OCTA (Novais and Waheed, 2016), and given the depth resolved nature of OCTA, it is possible to learn more about various retinal vascular layers in this condition. In addition to en face imaging volume rendering may help visualize the nature of vascular perfusion abnormalities. In areas of chronic edema there is pronounced loss of the deep vascular plexus (Spaide, 2016c; Tsuboi et al., 2017).

The amount of microvascular occlusion appears to be more pronounced in the deep vascular plexus as compared with the superficial plexus and appears to also be related to the appearance of paracentral acute middle maculopathy (Sellam et al., 2017b). Following anti-VEGF treatment most studies showed the vascular density does not change (Falavarjani et al., 2017; Rodolfo et al., 2017b), although some studies found a slight decrease in vascularity following treatment (Glacet-Bernard et al., 2016; Sellam et al., 2017a). Spatial correlation showed a loss of the ellipsoid band in regions where there was a loss of the deep capillary plexus (Kanakis et al., 2017). The foveal avascular zone can be enlarged after vein occlusion (Adhi et al., 2016; Kang et al., 2016; Salles et al., 2016; Wons et al., 2016). The quantity of residual perfusion is correlated with visual acuity (Kang et al., 2016; Samara et al., 2016). The deep vascular plexus appears to be affected more than the superficial plexus (Coscas et al., 2016a), particularly in areas of cystoid macular edema. Volume rendering can overcome some of these problems since it doesn’t rely on segmentation. Retinal neovascularization is obvious as vascular proliferation above the internal limiting membrane. Treatment with anti-VEGF agents causes a decrease in the amount of edema, but no significant improvement in vascular perfusion once any potential abnormalities in segmentation are corrected. In branch retinal vein occlusion, visual acuity was related to parafoveal areas of nonperfusion and abnormalities in perfusion of the superficial and deep vascular plexuses (Kadomoto et al., 2017). Segmentation in en face imaging often is not correct in areas of inner retinal thinning.

### 11.3. Retinal artery occlusion

Acute occlusion of the main or branch retinal artery causes a reduction in flow in the affected vessel ranging from reduced to complete loss of low. The occlusion can be secondary to embolism, thrombosis, elevated intraocular pressure, especially from direct pressure or inflammation. The occlusion from any of these causes can be temporary, inducing damage in minutes even though flow may be restored later. Central retinal artery occlusion causes ischemic necrosis, whitening, and opacification of the inner retina. The opacification may persist for hours to days and causes the cherry-red spot appearance because the central fovea remains unchanged in color. Mild retinal artery occlusions are often overlooked because of the modest amount of associated retinal opacification. About ¼ of patients have a cilioretinal artery, which may perfuse a variable amount of the maculopapillary bundle and nasal fovea.

#### 11.3.1. Optical coherence tomography angiography findings

OCTA findings of a limited number of cases have been reported, with findings that for the most part would be expected given the known pathophysiology and findings in other means of imaging (Damento et al., 2016; Hwang et al., 2017). Of interest was the finding that variable amounts of nonperfusion in the superficial and dep plexuses occur and these areas do not necessarily overlap. Paracentesis in one case showed a dramatic improvement in retinal blood flow. As with other forms of imaging, some reperfusion can be seen during follow-up after arterial occlusion (Bonini Filho et al., 2015a).

### 11.4. Retinal vascular cystoid macular edema

Cystoid macular edema (CME) is one of the leading causes of vision loss related to retinal vascular disease. There are more than 1/3 of a billion people in the world with diabetes; this number is expected to reach nearly 600 million by 2035 (Guariguata et al., 2014; Hu, 2011). The most common sequella of diabetes causing vision loss is macular edema (Aiello, 2003; Bresnick, 1983; Davidson et al., 2007; Ferris and Patz, 1984). The second most common retinal vascular disease is retinal vein occlusion (Cugati et al., 2006; Klein et al., 2008), of which cystoid macular edema is a common vision threatening consequence. The pathophysiologic steps involved in CME from retinal vascular disease are not completely known. Elucidation of the pathophysiology of CME can lead to increased understanding of the disease and potential for new therapeutic insights for a vision threatening condition expected to become more common.

Fluorescein angiographic abnormalities seen in diabetic macular edema include capillary non-perfusion, microaneurysms and capillary telangiectasis, with leakage of fluorescein from segments or regions of capillaries. In retinal vein occlusions the vascular abnormalities include capillary non-perfusion, capillary telangiectasis, with leakage of fluorescein from segments or regions of capillaries with dilated and tortuous venous segments. The pattern of diabetic macular edema shows poor correlation to the location of microaneurysms (Blair et al., 2008) and only a modest correlation to fluorescein leakage patterns (Bolz et al., 2009; Neubauer et al., 2007). In evaluating normal monkeys using histopathology, Weinhaus et al. (1995). determined that FA also did not show deeper planes of vessels in the retina. The deep capillary plexus was shown to be not visible in fluorescein angiograms in humans (Spaide et al., 2015c). Our ideas of the pathophysiology of retinal vascular CME have been formed using FA, which doesn’t visualize all relevant vessel layers in the retina. OCTA offers the ability to image all relevant vascular layers on the retina and is not affected by fluorescein leakage.

**Fig. 43.**
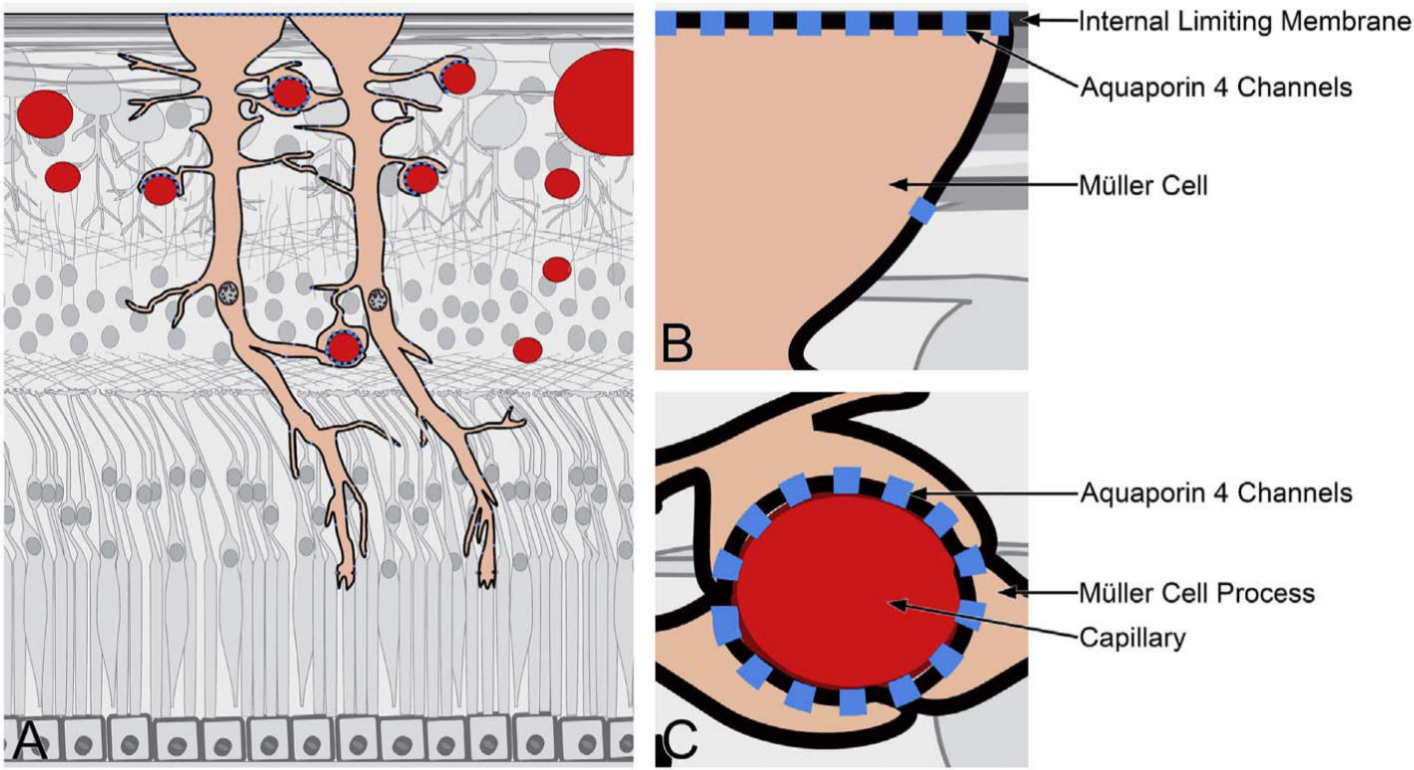
Müller cell schematic. (A) Müller cells traverse most of the thickness of the retina and are the major determinant of retinal fluid movement. Müller cells extend from the inner retina to the inner segments of the photoreceptors, where they form junctional bonds with the photoreceptors. This establishes a boundary at the outer border of the retina that has limited permeability to the passage of water. (B) The footplates of the Müller cells form the internal limiting membrane of the retina. At the inner border of the Müller cell there are highly concentrated aggregates of aquaporin 4 channels (schematically shown as blue rectangles) that colocalize with potassium channels (not shown). There are aquaporin channels scattered over the surface of the Müller cell, but at a much lower frequency. (C) Müller cell processes surround the retinal vessels of the inner and deep vascular layers. The appositional surface has densely packed aquaporin 4 channels along with potassium transport channels (not shown). Reprinted from Spaide (2016b).

**Fig. 44.**
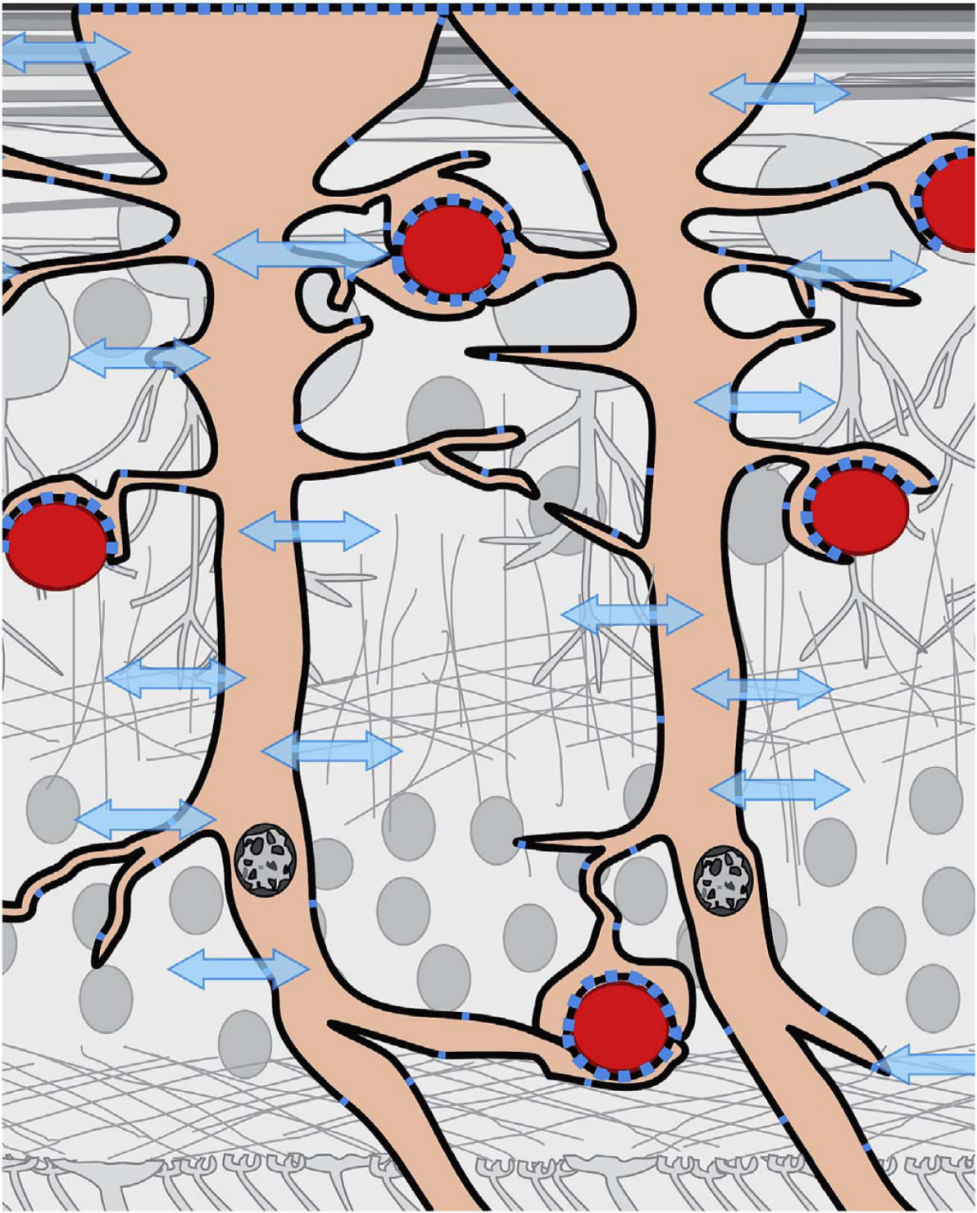
Schematic drawing of the retina highlighting the Müller cells, which can buffer both K+ ions and water (double arrows), which move in tandem. Reprinted from Spaide (2016b).

**Fig. 45.**
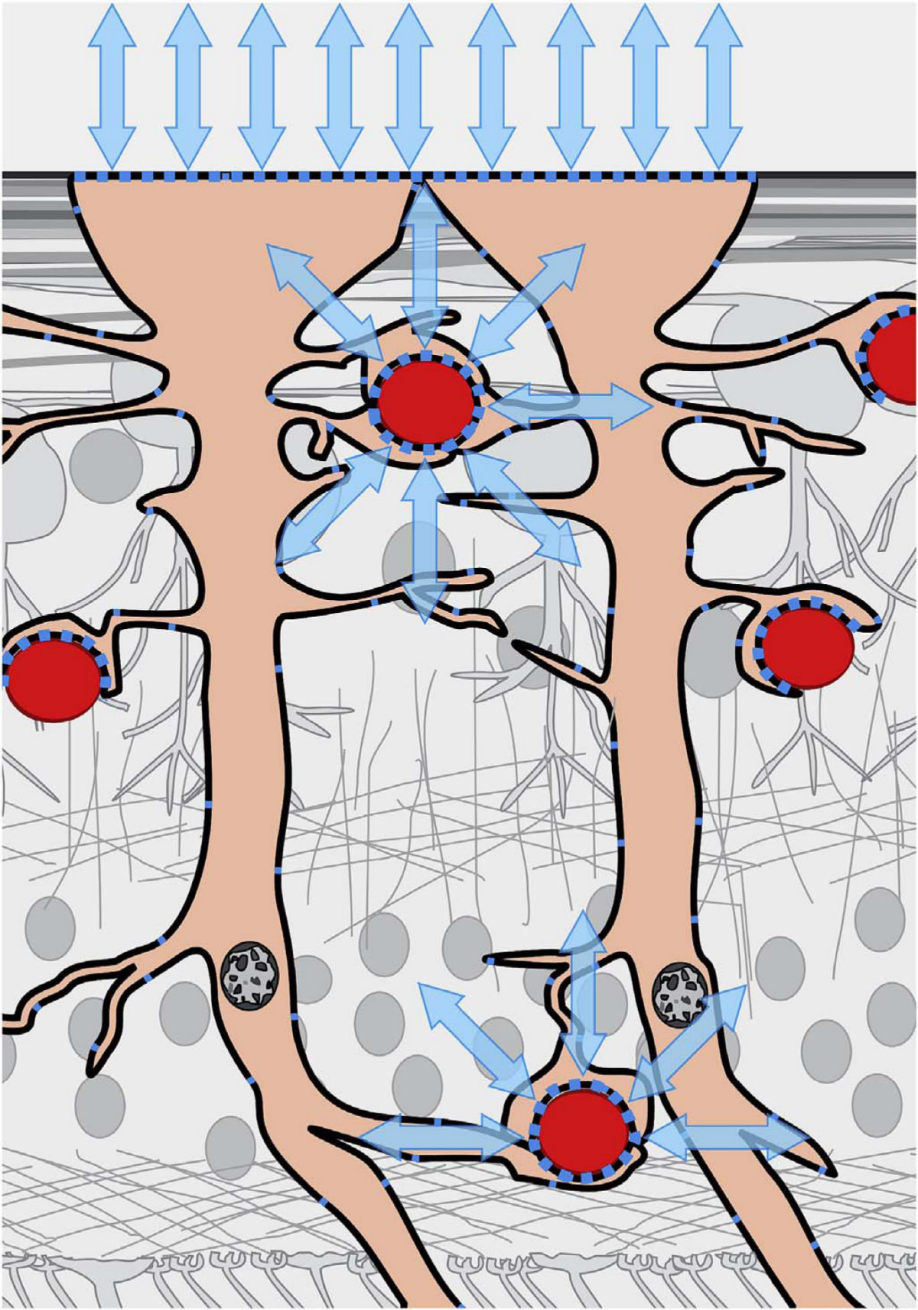
Excessive water, potassium, or both may be transported to the vitreous or to the superficial or deep plexus via potassium pumps and aquaporin 4 channels. The spatial buffering of potassium by this mechanism and that shown in Figure is called the potassium siphon.

Fluid entry into and out of the macula may occur at four main sites: the outer retinal border with the subretinal space, the inner retinal border with the vitreous, and the superficial and deep vascular plexus. The outer retina has the external limiting membrane, which is the term given to the linear aggregate of junctions between the outer portions of Müller cells and the inner segments of the photoreceptors (Fig. 43). These junctions show labeling of occludin, junctional adhesion molecules, and zonula occludens-1, typical of tight junctions and also have proteins associated with adherens junctions (Campbell et al., 2006; Matsunaga et al., 1988; Paffenholz et al., 1999). If the external limiting membrane is intact, exudation from the below the retina would cause serous detachment of the retina; if the external limiting membrane is defective there can be passage of fluid into the outer retina. The external limiting membrane may serve as a barrier for fluid leaving the retina to be pumped from the subretinal space by the retinal pigment epithelium (RPE). Fluid can be produced internally within the retina from metabolism; oxidation of glucose, a preferred substrate of the retina, generates 6 H_2_O. Metabolism of fatty acids produces a large numbers of water molecules. Müller cells are one of the most important actors in controlling homeostasis of the water and ionic concentrations in the retinal interstitial areas. The aquaporin tetramers are densely packed at the Müller cell footplate and on the Müller cell processes surrounding retinal vessels in the superficial and deep vascular plexus. When potassium ions are transported, water is induced to move as well through aquaporin 4 channels (Figs. 44 and 45) (Jin et al., 2011).^218^ Thus Müller cells can pump fluid to and from the blood supply provided by the superficial and deep plexus and also the vitreous cavity. Müller cells have huge amounts of active carbonic anhydrase (Newman and Reichenbach, 1996; Newman, 1994; Ochrietor et al., 2005), which is important in regulation of pH, CO_2_ and bicarbonate management, and water transport (Ogilvie et al., 2007). Bicarbonate related transport mechanisms are present in Müller cells (Newman, 1999) and also are involved in or effect transport of K^+^ and Na^+^ ions.

**Fig. 46.**
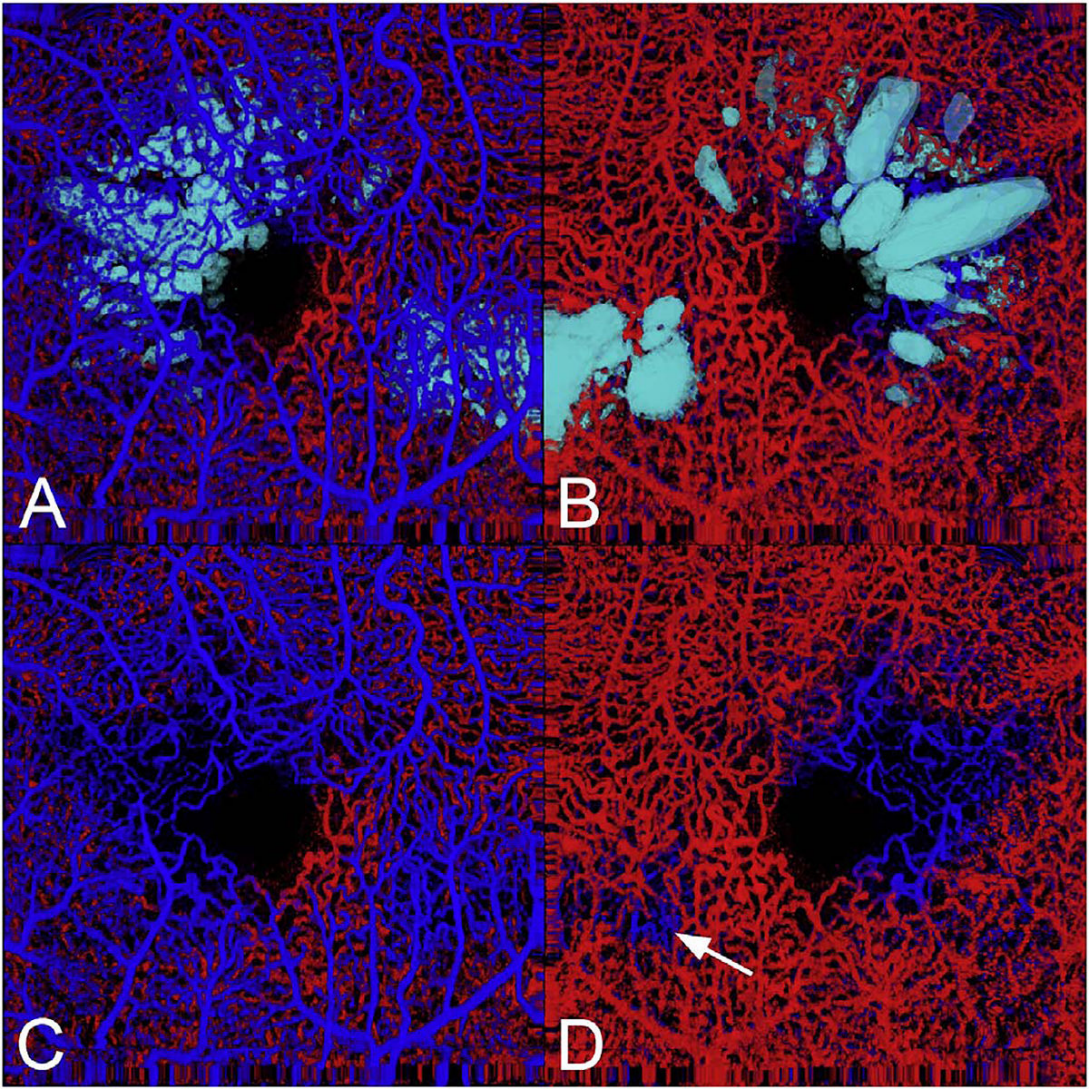
Varying sized flow voids in the deep vascular plexus in eyes with diabetic macular edema. (A) The cystoid spaces (in teal) are integrated into the volume rendered vascular structure. The cystoid spaces are located below the superficial vascular plexus. (B) The data set was rotated to show the view of the retina from below. The structure of the cystoid spaces is evident and there is a loss of flow signal from the deep vascular layer larger in extent than the cystoid spaces. (C) The imaging data from the cystoid spaces was not shown. The absence of the deep plexus under some areas of the superficial vascular plexus can be seen. (D) With the dataset rotated so the deep plexus is on top, the group of larger cystoid spaces is seen to correlate to a region in which the deep plexus flow data is not apparent. The smaller region of edema has an even smaller flow void (arrow). Reprinted from Spaide (2016b).

**Fig. 47.**
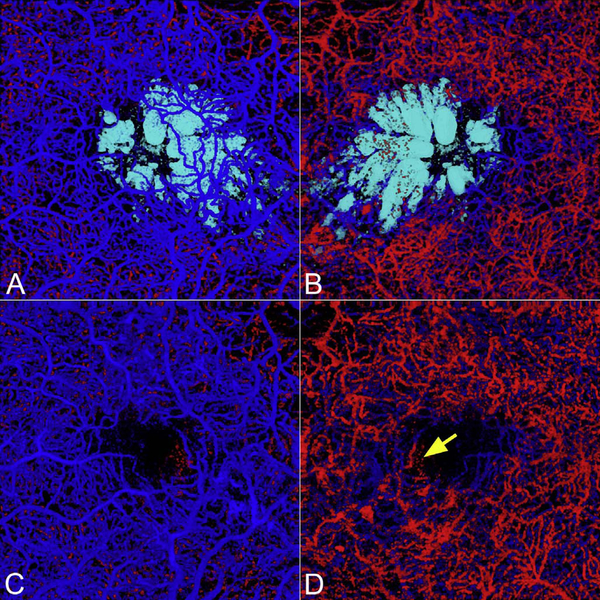
Volume rendered angiographic and structural optical coherence tomography of central retinal vein occlusion. (A) View of the retina from the vitreous side; the superficial plexus is blue, the deep plexus red, and the cystoid spaces are cyan. Note the decreased vascular density overlying areas of cystoid edema. (B) When viewed from the underside of the retina the extent of the cystoid spaces is easier to visualize. (C) After an intravitreal injection of ranibizumab the cystoid edema has temporarily resolved. The vascular abnormalities in both the superficial vascular plexus and deep plexus show significant abnormalities. (D) There are several subtle aspects in the macular regions. Thinning of the macula allows some of the superficial vessels to be at the same level of the surrounding deep plexus. These vessels can have segments that are red (open arrowheads). Small particulate areas of decorrelation (white arrow) likely represent protein or lipoprotein aggregates. Larger collections are also present (yellow arrow). Note the small and large particles are not vessels because they are spherical, don’t connect to any other structure, and don’t necessarily have sizes corresponding to the surrounding vessels. Modified from Spaide (2016b).

**Fig. 48.**
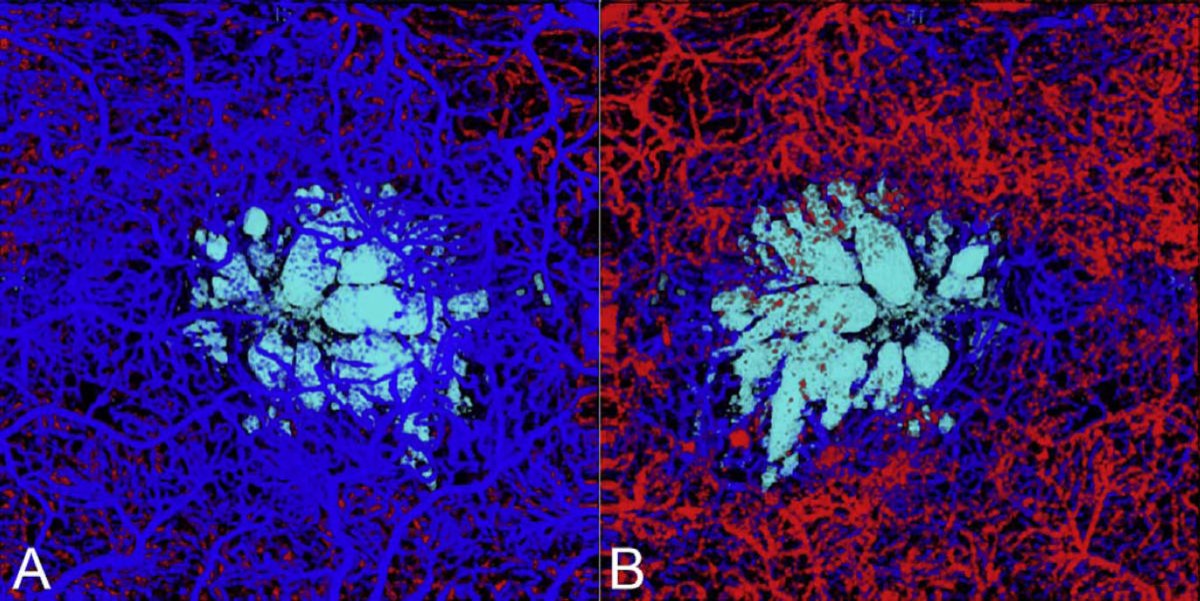
The patient had a recurrence of edema visible from the top (A) and bottom (B) of the retina. Note the similarity in the distribution of cystoid spaces to that seen in Fig. 47. Note the particulate dots of decorrelation that are not attached to any vascular structure. Reprinted from Spaide (2016b).

**Fig. 49.**
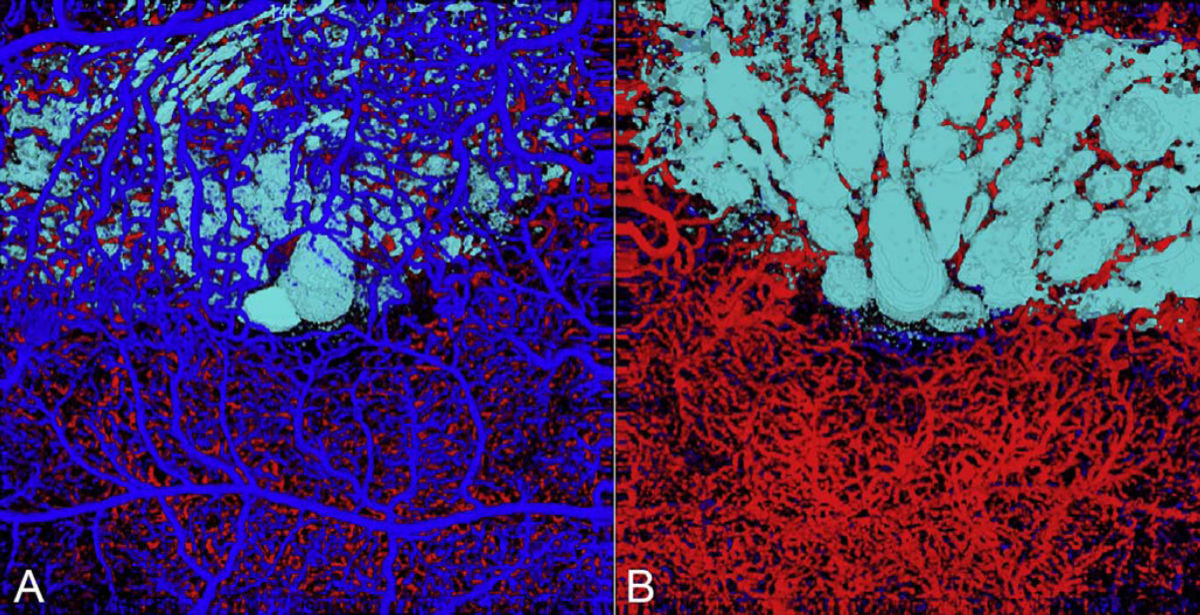
Volume rendered angiographic and structural optical coherence tomography of an eye with a branch retinal vein occlusion. (A) The superficial vascular plexus is shown in blue, the deep in red. The cystoid spaces were derived from the matched structural optical coherence tomography scan and are shown in cyan. Some of the deep vascular plexus is seen above the cystoid spaces (open arrows). (B) The cystoid spaces are easier to visualize when the imaged is flipped about the vertical axis. Note the extent and distribution of the cystoid spaces.

**Fig. 50.**
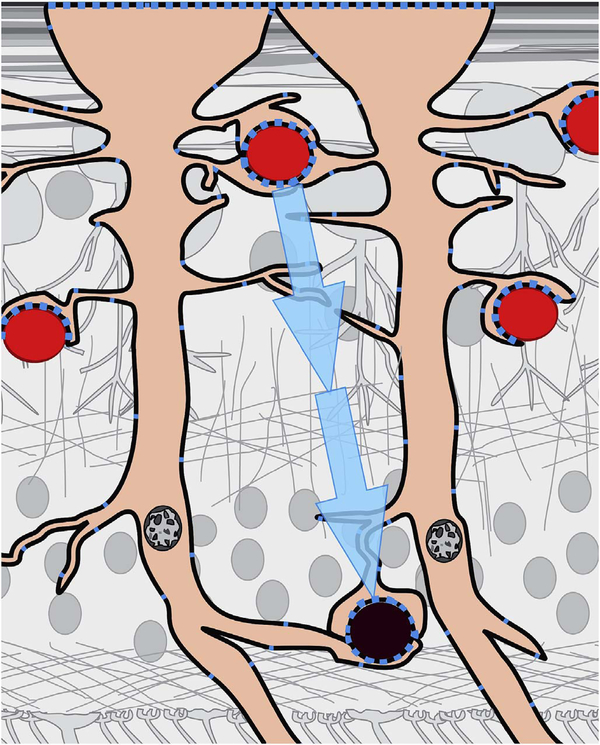
Fluid is potentially removed from the retina by action of the Müller cells and the deep vascular plexus. There is no functional mechanism to clear excess fluid through the deep plexus if that layer has no flow. Reprinted from Spaide (2016b).

In retinal vascular CME FA demonstrates increased leakage from the superficial vascular plexus. OCT angiography, particularly combined volume rendered angiographic and structural OCT, shows cystoid spaces in diabetic retinopathy and veno-occlusive disease occurs in regions where there are abnormalities in the inner vascular plexus such as telangiectasis and microaneurysms and regions where there is decreased or absent deep plexus flow (Figs. 46–50) (Spaide, 2015c, 2016b, c). Treatment with resolution of the edema is not associated with significant restoration of flow in the compromised deep plexus and when edema returns it occurs in the same areas of decreased or absent deep plexus flow. The vessels of the deep plexus are not simply displaced by the cystoid spaces. There is no evidence of vessels ‘piled-up’ or aggregated around the outer borders of the cystoid spaces, either by the present OCT angiography or past fluorescein angiographic or histopathologic studies. On the contrary, the vessels around the areas of cystoid spaces often show poor deep vascular plexus flow surrounding areas of absent flow in OCT angiographic imaging. In larger areas of lack of perfusion in both the inner and deep vascular layers, there is generally no edema (Spaide, 2016b).

Increased leakage from the retinal vessels as seen during FA is an integral feature of retinal vascular CME. Leakage does not necessarily mean CME, so other factors must be involved. The formation of CME implies an increase in fluid accumulation within the macula that during the formation of the edema outpaces the ability of fluid removing mechanisms to compensate. One mechanism by which fluid is removed from the retina is the pumping ability of the RPE. There are some physiologic considerations that mitigate against the role of the RPE in many cases of retinal vascular CME. The external limiting membrane limits passage of water in both directions, and so would impede the ability of the RPE pump to effectively remove water from the mid-retina. The passage of water and solutes is also partially restricted by the outer plexiform layer, which would form another barrier of sorts between where the cystoid spaces are most commonly found, the inner nuclear layer, and the RPE.

**Fig. 51.**
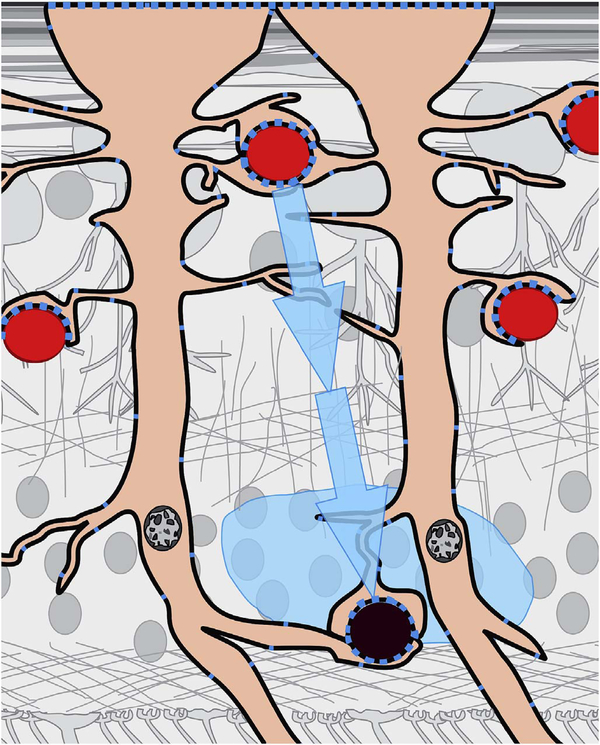
Without a mechanism to evacuate excess fluid, cystoid spaces are created. Reprinted from Spaide (2016b).

In normal circumstances fluid production may originate from the superficial retinal vascular layer that courses through the interstitial tissue of the retina to be absorbed through the actions of Müller cells and the deep capillary plexus (Spaide et al., 2017). Increased leakage from the superficial vascular layer (Fig. 46) could potentially reach a level that the fluid removing capabilities would be overwhelmed. The clinical observation that cystoid spaces occur in regions that have decreased or absent deep plexus flow suggests the deep plexus may be involved in removal of fluid from the retina as potentially mediated by the deep plexus (Figs. 47–49). If there is absent flow in the deep plexus the Müller cells couldn’t traffic excess fluid out of the retina from that site. This would lead to accumulation of fluid within the retina as constrained by barriers to hydraulic conductivity. The deep plexus vascular plexus may also be a source of excess fluid. Leakage from the deep plexus (Figs. 50 and 51), something that can’t be quantified or imaged at present, could contribute to the excess fluid in the middle layers of the retina and would cause fluid loading into the deeper portions of the retina. This may play a role in the development of serous detachment. It is also possible leakage from the deep plexus may occur in acute Irvine-Gass syndrome.

### 11.5. OCT angiography in non-exudative AMD

Non-neovascular AMD is characterized by abnormalities of the RPE, Bruch’s membrane, outer retina and the choriocapillaris and may be associated with normal or compromised vision. Early non-neovascular AMD can either progress to advanced dry AMD (atrophy) or to neovascular disease. OCT angiography has provided us the unique ability to correlate vascular changes to structural changes and to follow them longitudinally in vivo. It has also led to the confirmation of the previously described asymptomatic MNV (Bird et al., 1995; Group, 2008; Hanutsaha et al., 1998).

#### 11.5.1. Pitfalls to the evaluation of choriocapillaris with OCTA

The evaluation of the choroidal vasculature is of interest in the study of non-exudative age related macular degeneration. However, evaluation of structures beneath the RPE on OCT is confounded by shadowing from the RPE and the structures overlying the choriocapillaris, such as drusen and RPE clumping. Therefore it is very important to evaluate OCTA images of the choriocapillaris with care to avoid attenuation artifacts when evaluating for choriocapillaris dropout (Mullins et al., 2011). OCTA images must always be evaluated with the corresponding *en face* OCT structural scans segmented at the same level as the choriocapillaris. Dark areas on the OCTA may represent areas of true choriocapillaris loss or may represent areas of shadowing arising from attenuation of OCT signal (Cole et al., 2017). When there is a low or noisy OCT signal, accurate OCTA decorrelation signals cannot be obtained and these regions are threshold masked to remove the invalid OCTA data. Therefore, in regions with low or noisy OCT signals, the absence of an OCTA signal does not necessarily represent choriocapillaris loss. When evaluating OCTA with the corresponding structural *en face* OCT scan, if there is a dark area on the OCTA and the corresponding area on the OCT is dark as well, this area represents signal loss and an attenuation artifact. However, if the area is dark on OCTA, but the en face OCT shows the presence of signal, the OCTA measurement is valid and there is with true loss or flow impairment of the choriocapillaris. Areas of ambiguity, with some, but not all loss of signal, must be interpreted with extreme caution, since threshold masking in these areas may give a false impression of vascular atrophy or flow impairment (Cole et al., 2017). Alternatively, the presence of low OCT signal but above the threshold level may give an OCTA decorrelation arising from noise, making it appear as if there is flow, when there is none. Overall, spectral domain OCTA is more prone to attenuation artifacts than the longer wavelength swept source OCT devices, and therefore the choriocapillaris can be more reliably visualized on swept source OCTA than on spectral domain OCTA (Bloom and Singal, 2011; Choi et al., 2013a; Lane et al., 2016; McLeod et al., 2002; Pepple and Mruthyunjaya, 2011).

#### 11.5.2. Non-neovascular AMD

**Fig. 52.**
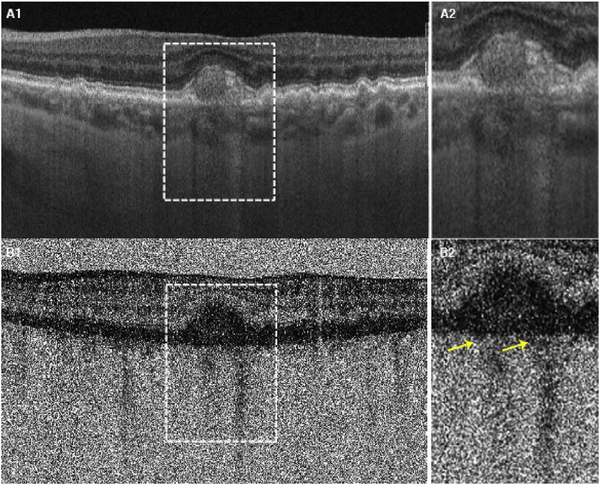
Choriocapillaris loss under drusen. A1 is the intensity B scan image and A2 is a magnified view of the druse in A1 showing good penetration of light under the druse (i.e. no shadowing). B1 is the angiographic image and B2 is a magnified angiographic image. Note the loss of choriocapillaris flow on the OCTA image noted under the druse (yellow arrow).

Drusen are the hallmark of age related macular degeneration and appear clinically as yellow-white excrescences deep to the retina varying in size, number and location. In OCT images, drusen appear as discrete excrescences overlying the inner collagenous layer of Bruch’s membrane and with the RPE draped over them. Histopathologic studies by Mullins and group have shown that there is a trend to decreasing choriocapillaris density with increasing AMD severity (Biesemeier et al., 2014; McLeod et al., 2002; Mullins et al., 2011). Structural OCT can be used to evaluate the volume of drusen, thereby quantifying drusen burden, as well as the integrity of the RPE and the outer retinal layers overlying the drusen. OCT angiography shows loss of choriocapillaris associated with some but not all drusen (Sarks et al., 1999; Zweifel et al., 2010) (Fig. 52). Another interesting feature to note is that the highly reflective RPE overlying drusen can cause the appearance of intense projection artifacts from the overlying retinal vessels. These may make it appear as if there is neovascularization in association with the drusen when there is none.

**Fig. 53.**
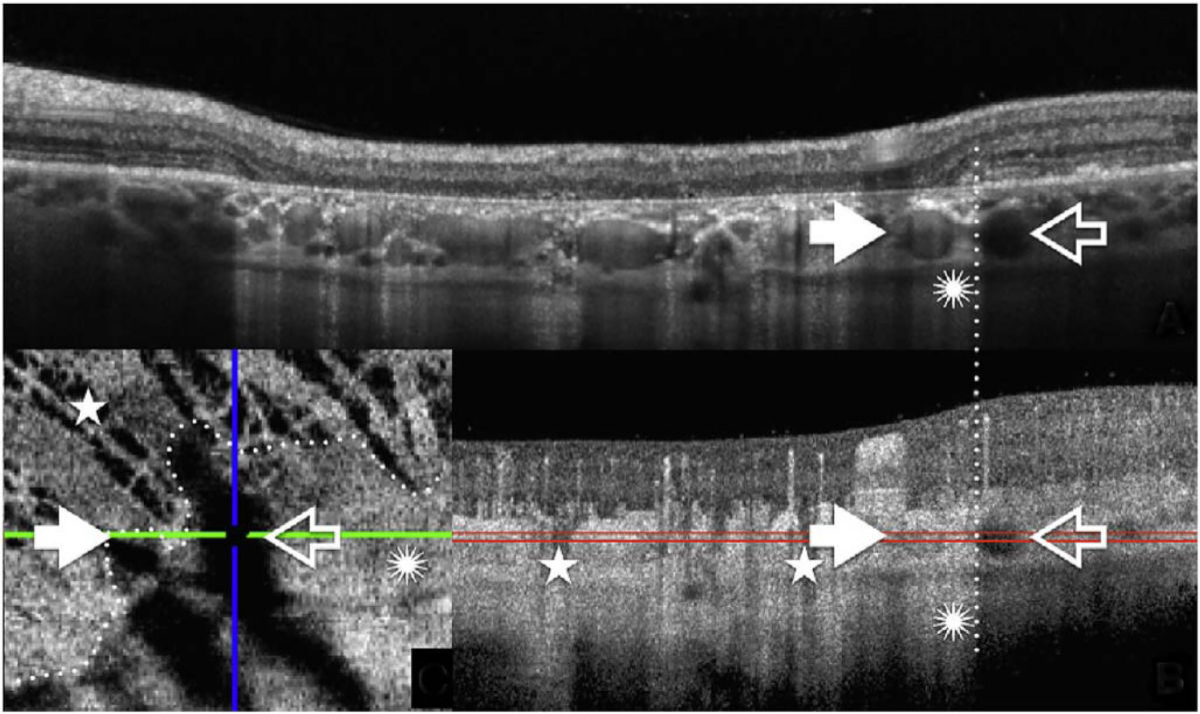
A. structural OCT B-scan. The black arrow shows choroidal Haller layer vessel not penetrated by the light. White arrows show choroidal Haller layer vessel penetrated by the light. Dotted line shows the separation between the area with and without RPE. Asterisk shows the effect of the projection artifact of the choriocapillaris B: OCTA B-scan. Stars identify area without choriocapillaris. C: OCTA en face image of the same region sampled at the level of the Haller layer as shown by the red lines.

**Fig. 54.**
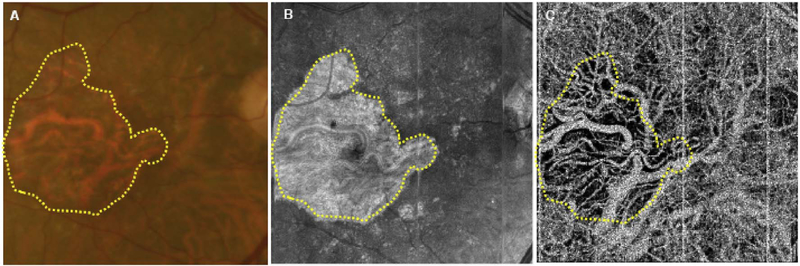
Choriocapillaris loss under geographic atrophy. This figure shows a color fundus photo (A) and a fundus autofluorescence image (B) of a patient with geographic atrophy (GA). The area of GA is outlined. Image C is an OCTA of the choriocapillaris slab underlying the area of GA outlined in yellow. Loss of choriocapillaris can be seen, and the larger choroidal vessels are seen occupying the space that would ordinarily be occupied by the choriocapillaris. Also note that there appears to be some CC thinning in some of the areas surrounding the area of atrophy.

**Fig. 55.**
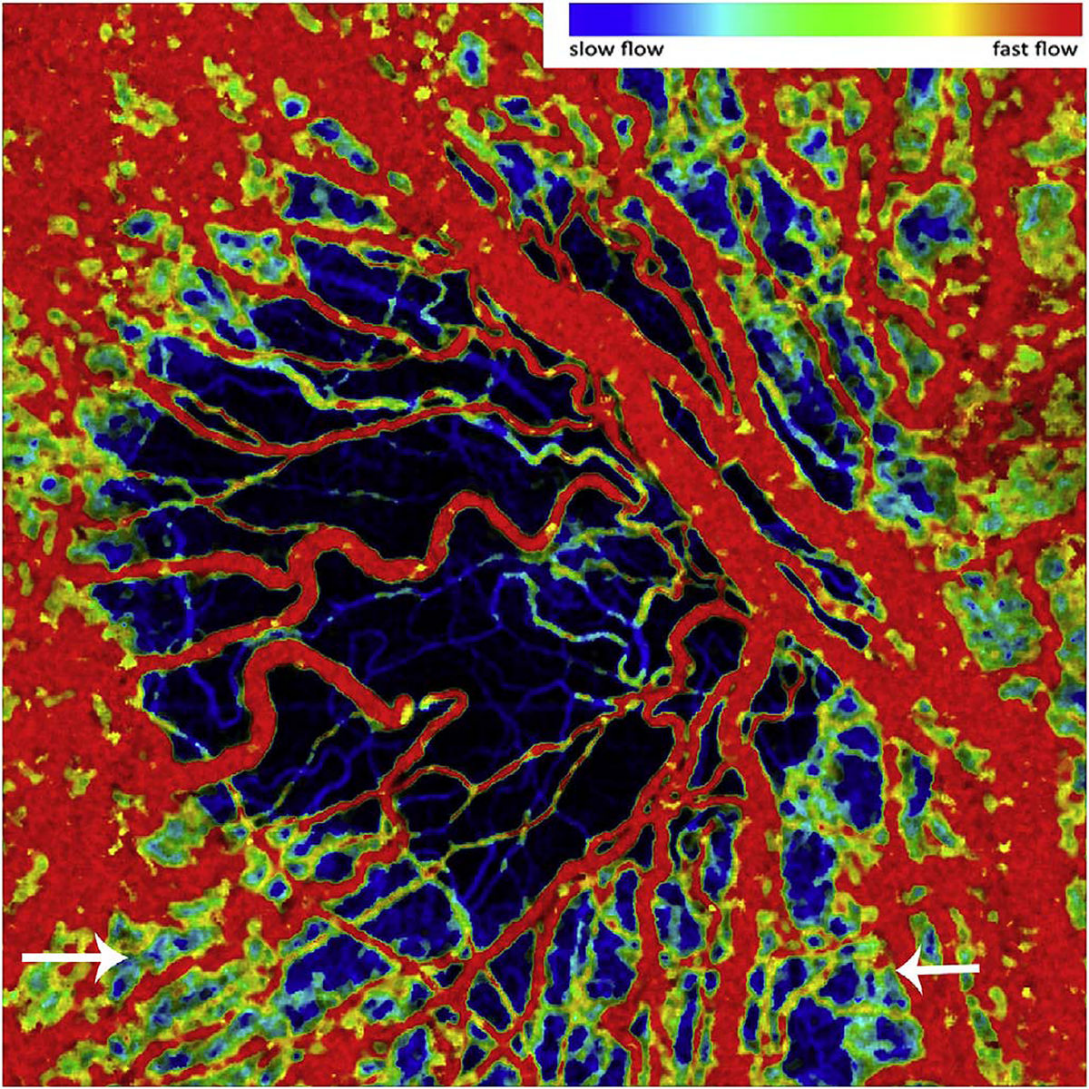
Semi-quantitative OCT angiography using variable interscan time analysis in a patient with geographic atrophy. Red represents fast flow and blue represents slow flow. Note that there is both choriocapillaris loss and slowing of flow underlying the area of geographic atrophy. Surrounding the area of atrophy, some areas have vessels with slower flow (between arrows) while other areas do not have slowing of flow.

At some point along the progression of non-neovascular AMD down the pathway of atrophy, OCT imaging can show thinning of RPE with increased light penetration or hyper-transmission below the level of the RPE into the underlying choroid and sclera (Schaal et al., 2017; Wu et al., 2014, 2015) (Fig. 53). This can start over a large druse, prior to the loss of substance of the druse. The color photographic diagnosis of geographic atrophy (GA), is made when there is a sharply defined area of decreased pigmentation allowing improved visualization of the underlying choroidal vessels (Fig. 54). The OCT findings of RPE thinning and hyper-transmission appear to be a preceding finding that is a point of no return along the pathway of RPE loss, but doesn’t meet the color photographic diagnosis of GA since the underlying choroidal vessels may not be visible. There may be a loss of the outer nuclear layer over the thinning creating a wedge-shaped depression of the outer retina. This has been called nascent GA. On using variable interscan time analysis (VISTA), these flow alterations mostly represent slowing of flow (Fig. 55).

OCTA has also recently demonstrated the presence of type 1 MNV in asymptomatic eyes that would otherwise be classified as intermediate AMD. This may occur in up to 30% of all patients with intermediate AMD per a small published case series (Carnevali et al., 2016). This finding supports a larger study done nearly 20 years previously in which approximately one-quarter of eyes harboring drusen alone of patients with fellow eyes having exudative AMD had ICGA evidence of asymptomatic MNV. This study by Hanutsaha has shown that progression to frank exudative disease is much higher in this group than it is in patients without neovascular plaques (Hanutsaha et al., 1998). The designation intermediate AMD is a holdover from earlier times when eyes were graded with color photography alone. Multimodal imaging provides more accurate ability to evaluate eyes, and the use of nonneovascular AMD to describe eyes with neovascularization is not appropriate.

The evolution of atrophy in eyes with AMD occurs with a final decline in visual function. Loss of the outer retinal structure, including the outer retinal bands and much of the thickness of the outer nuclear layer creates a condition known as outer retinal atrophy. This often occurs following the regression of subretinal drusenoid deposits, also known as pseudodrusen, and prior to the development of any RPE loss (Spaide, 2013). Once loss of the RPE occurs, and there is a complete loss of the RPE and outer retina, which has been called CRORA (Sadda et al., 2017b; Spaide, 2017c). Imaging of CRORA can be done with a variety of modalities, that show somewhat differing results; the color photography of CRORA yields what is termed GA. Histopathologic studies have shown RPE cell loss as well as choriocapillaris atrophy underlying the areas of geographic atrophy (Lutty et al., 1999; McLeod et al., 2002). Lipofuscin accumulates in RPE cells over life and can be used as a measure of the presence of RPE cells. However the retina often shows areas of atrophy that are larger than the corresponding size of the RPE loss (Bird et al., 2014). In addition, OCTA imaging shows choriocapillaris flow abnormalities as evidenced by areas of slower flow and measurable alterations in the distribution of signal void sizes.

Several concurrent changes occur with the development of CRORA: there is loss of the RPE and its contained melanosomes and liposomes, the choriocapillaris is lost, the middle portions of the choroid responsible for providing blood flow also regress causing the deeper, larger choroidal vessels to come to lie in the inner choroid. These vessels can be seen spanning the area underlying the GA (Choi et al., 2015; Ferrara et al., 2014; Sarks et al., 1988; Schuman et al., 2009; Sohrab et al., 2012). These deeper vessels supply and drain blood from other areas of the choroid, which are not necessarily involved with atrophy. In areas of CRORA these larger vessels are readily seen to have flow, and the course of the larger vessels can be followed under the adjacent intact RPE where they appear dark. Thus the ability to see flow in the larger choroidal vessels is influenced by the optical effects of the RPE, choriocapillaris and potentially the middle sections of the choroidal thickness. This implies light scattering, rather than just fringe wash-out is the main reason why flow usually cannot be seen in the deep choroid with OCTA.

The site of where the initial insult in the development of atrophy is seen depends, in part, on the imaging modality used. There is a general loss of choriocapillaris flow associated with drusen and subretinal drusenoid deposits in histologic examination. This is supported by OCTA examination. Hyper-transmission through the RPE seems to occur over larger drusen implying a physical separation of the RPE from the choriocapillaris may also play a role. The rate oxygen delivery from the choriocapillaris has been shown to decrease with increasing distance from the choriocapillaris (Linsenmeier and Padnick–Silver, 2000). The RPE secretes factors, such as VEGF that help maintain the vitality of the choriocapillaris. It is possible that a large deposit of lipoproteinaceous material, a druse, could impede the diffusion of released VEGF. OCT has shown findings of nascent GA can precede RPE atrophy, as does outer retinal atrophy; a recent OCTA study revealed an association between regions of nascent GA and choriocapillaris alteration (Moult et al., 2016). Using a reductivist approach may help simplify the search for the cause of atrophy if only one exists, but a systems approach would suggest there may be more than one pathway that leads to a phenotypically similar outcome.

### 11.6. Exudative age related macular degeneration

Perhaps the most immediate clinical applicability of OCTA in the diagnosis and management of disease is in neovascular age related macular degeneration. Neovascular AMD is characterized by new vessel growth, either underneath the RPE (Type 1 MNV), or between the RPE and the retina (Type 2 MNV) or starting within the retina (Type 3 MNV) (Freund et al., 2010). Note Type 3 neovascularization may not have a choroidal component. Therefore it is not accurate to call it CNV, but rather it is more precise to call it macular neovascularization or MNV. In other conditions such as pathologic myopia pseudoxanthoma elasticum (RXE) or multifocal choroiditis and panuveitis, Type 3 neovascularization does not occur, so the term CNV is also appropriate. Several studies have shown the utility of OCTA in the diagnosis and monitoring of the various kinds of MNV (Carnevali et al., 2016; Inoue et al., 2016; Jia et al., 2014; Kuehlewein et al., 2015b; Moult et al., 2014; Souied et al., 2016; Spaide, 2015a).

#### 11.6.1. Diagnosis of macular neovascular disease

**Fig. 56.**
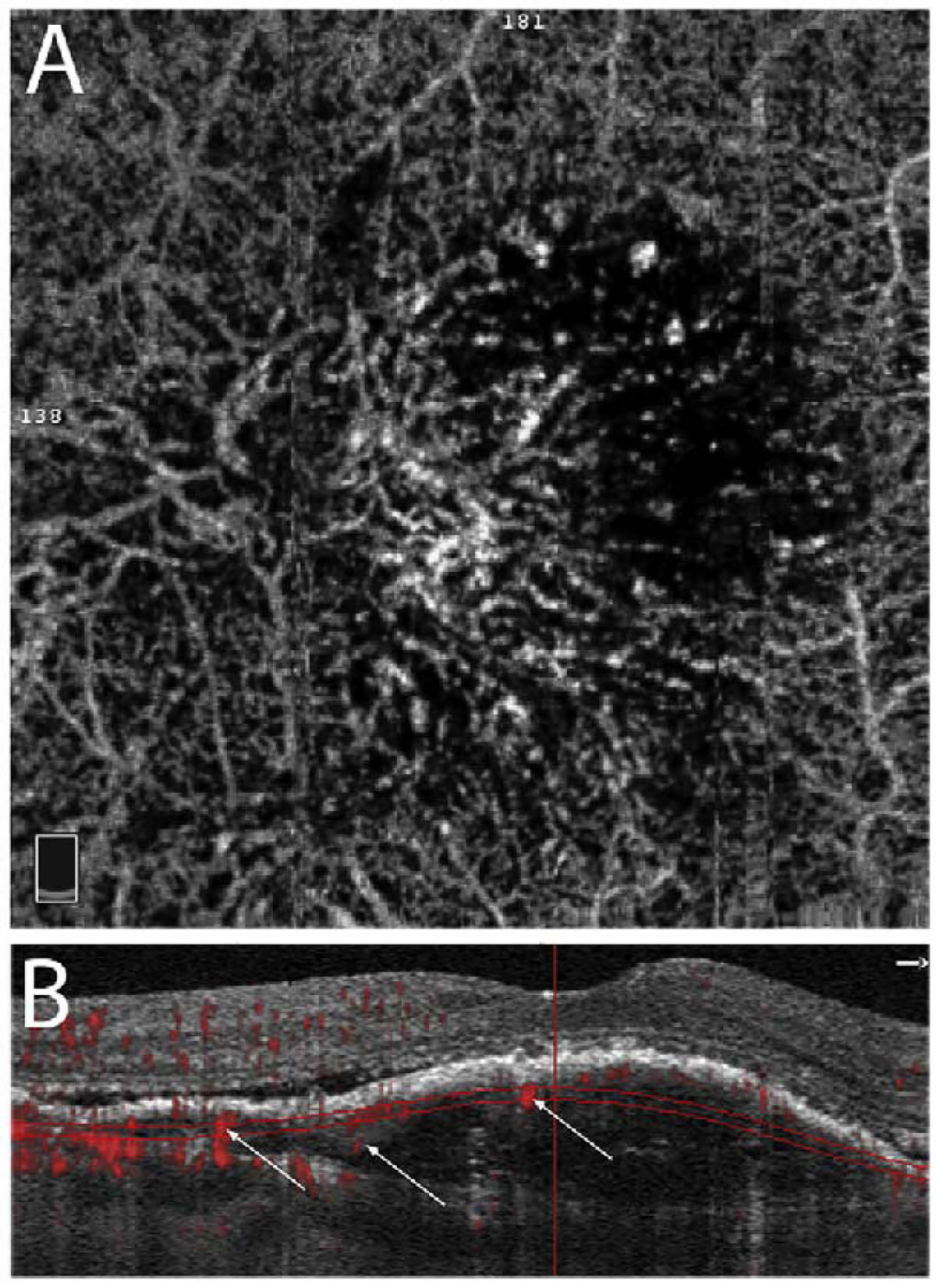
En face OCTA (A) and B scan with flow overlay (B) of a patient with type 1 macular neovascularization (MNV). The B scan shows a pigment epithelial detachment with flow noted within the pigment epithelial detachment (white arrows).

OCTA is useful in the diagnosis of the various forms of MNV, and even more so since the angiographic image on OCTA comes co-registered with a structural OCT. Since structural OCT has been used to describe the OCT based classification system of MNV, this direct correlation between the structural and angiographic image aids in the diagnosis and classification of the MNV in a more direct manner than FA which tried to correlate leakage characteristics to structural characteristics (Coscas et al., 2015). Type 1 MNV can be visualized as a network of vessels lying between the RPE and the Bruch’s membrane, often in the setting of a fibrovascular pigment epithelial detachment (PED) visualized on the OCT (Inoue et al., 2016). Fluid clefts within the PED suggest the presence of active MNV, and there may also be subretinal fluid seen. Type 2 MNV, on the other hand, is seen as a network of vessels in the normally avascular outer retina (Souied et al., 2016) (Fig. 56). This may be associated with subretinal and intraretinal fluid as well as the presence of subretinal hyper-reflective material on the structural OCT scans. While feeder vessels were occasionally seen in type 1 and type 2 MNV on video indocyanine green angiography, type 1 and 2 MNV often show one or more trunk vessels on OCTA imaging, consistent with known histology (Green and Enger, 1993).

The trunk vessels are generally large in diameter and branch to form vessels within the lesions. These vessels show limited branching and prominent anastomotic connections between the larger-diameter vessels including peripheral anastomosis at the edges of the lesions (Gong et al., 2016; Kuehlewein et al., 2015a; Spaide, 2015a). There are fine fibrillary vessels arising from these larger blood vessels both within the substance of the lesion as well as at its margins. Also, as had been previously described histopathologically, there may be drop out of the choriocapillaris surrounding areas of MNV (Bhutto and Lutty, 2012). Loss of the RPE and underlying choriocapillaris can occur in areas of chronic subretinal fluid from exudation. However, decreases in choriocapillaris flow are detectable by OCTA in eyes with MNV and in their fellow eyes uninvolved with MNV. It has been postulated that the hypoxic environment created by the choriocapillaris loss may induce release of VEGF by the intact overlying retinal pigment epithelial cells, which may, in turn, give rise to the MNV (Bhutto and Lutty, 2012). The growth of neovascularization in these eyes may recapitulate the functions of the impaired choriocapillaris. These areas of choriocapillaris loss surrounding MNV, similar to the ones reported on histopathologic studies, have also been reported using OCTA (Moult et al., 2014).

**Fig. 57.**
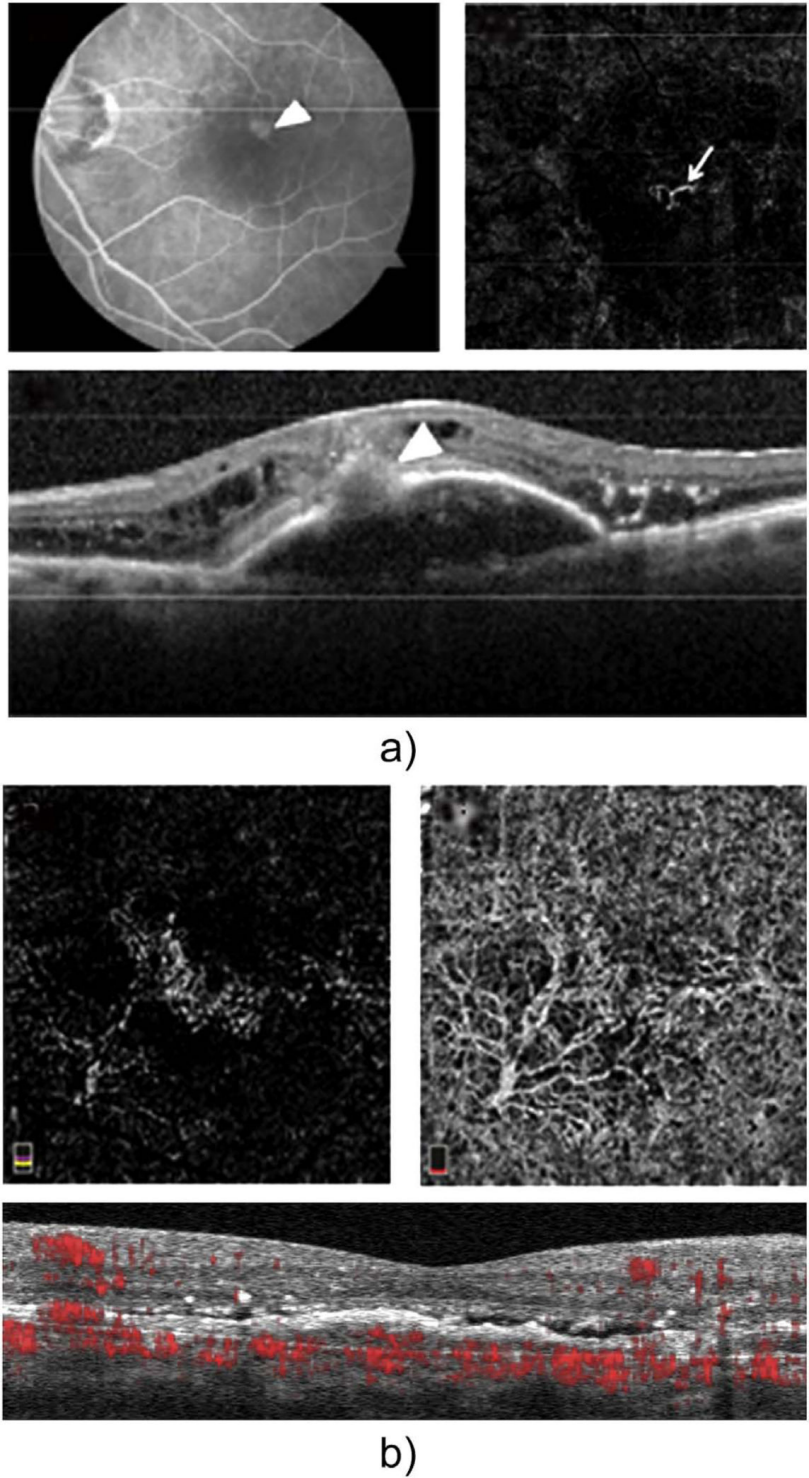
A type 3 (A1-A3) and mixed type 1 and 2 (B1-B3) MNV is seen. A1 is a fluorescein angiogram showing the type 3 lesion. On OCTA, the feeder vessel (A2) that extends from the retina through the break in the RPE (arrowhead A3) and into the pigment epithelial detachment is seen. B1-B3 demonstrate a mixed type 1 and 2 MNV with a type 1 component seen on choriocapillaris segmentation (B2) and a type 1 component seen on outer retinal segmentation. Note the trunk vessel centrally and the arborizing network of vessels around it.

Type 3 MNV, on the other hand, (previously known as retinal angiomatous proliferation and previous to that deep retinal anomalous complexes) generally arises from the retina, and on OCTA can be visualized as a discrete high flow linear structure extending from the middle retinal layers into the deep retina and occasionally also past the RPE (Fig. 57). This vascular structure may be associated with a pigment epithelial detachment, hyper-reflective foci in the outer retina as well as intraretinal hemorrhages and cystic changes. As the type 3 MNV develops, it can be seen as branching vessels that anastomose with the deep retinal capillary plexus, extends into the outer retina and eventually into the sub-RPE space (Kuehlewein et al., 2015b).

One of the big challenges of diagnosing MNV on OCTA is eliminating artifacts that may mimic MNV. These include projection artifacts from the more superficial retinal vasculature that may give the appearance of MNV where there is none (Bhutto and Lutty, 2012). Additionally, and especially in type 3 MNV, bright projection artifacts from overlying retinal vessels on the highly reflective PED may sometimes make it appear as if there is MNV where there is none (Falavarjani et al., 2017a; Zhang et al., 2015a). Moreover, in patients with areas of macular atrophy and corresponding choriocapillaris atrophy, the displacement of the larger choroidal vessels into the space ordinarily occupied by the choriocapillaris may cause the appearance of MNV. A careful mapping of the vessels to the structural layers in which they first start appearing can avoid this misinterpretation. In OCTA of MNV as in real estate, location is key. Careful mapping of the superficial vessels and comparing these vascular patterns to those seen in the presumed MNV can avoid misinterpreting projection artifacts from these overlying vessels as MNV. The projection artifact tools on the commercial machines, although imperfect, can often remove many of these artifacts. Finally, in the case of larger choroidal vessels beneath areas of atrophy, the location of these vessels beneath (and not above) the Bruch’s membrane helps differentiate them from MNV.

Studies have looked at the sensitivity and specificity of OCTA in identifying MNV (de Carlo et al., 2015a; Gong et al., 2016). Looking at all types of MNV, initial studies demonstrated a sensitivity of ~50% and a specificity above 80% (de Carlo et al., 2015a). This, however, included cases that had large amounts of subretinal hemorrhage. Presence of hemorrhage blocks penetration of OCT signal and therefore visualization of MNV, causing a false negative result on OCTA. In a small study, Bonini et al. showed a much higher sensitivity (approaching 100%) in patients with type 1 MNV, and more recently, the larger COFT 1 study demonstrated a similarly high sensitivity for detection of type 1 MNV (66.7%) (Bonini Filho et al., 2015b; Inoue et al., 2016). This was increased to over 87% when structural OCT data was used in conjunction with the OCTA data.

#### 11.6.2. Discrimination between active and inactive MNV

There is much effort being put into whether OCTA can be used to discriminate between active and inactive MNV (Liang et al., 2016). One major difficulty is that there is no accepted definition of what active MNV is. Some authors have concluded that features on OCTA such as the presence of fine vessels at the MNV edge and a peri-lesional dark halo may indicate activity while the presence of many large ‘dead tree’ like vessels and a paucity of fine branching vessels may indicate quiescence (Coscas et al., 2015). However, a paucivascular fibrotic scar would appear to have low vascularity, but these lesions also are associated with poor acuity. Lesions with extensive vascularity, although potentially associated with signs of fluid exudation, often have good acuity. MNV is living tissue produced at the behest of living tissue, and its eradication may obviate the goal of recapitulating the choriocapillaris. Of course, not every aspect of MNV is desirable, but the future of MNV treatment in AMD may be management and selection of desirable aspects as compared with eradication of the whole lesion (Spaide, 2017b).

**Fig. 58.**
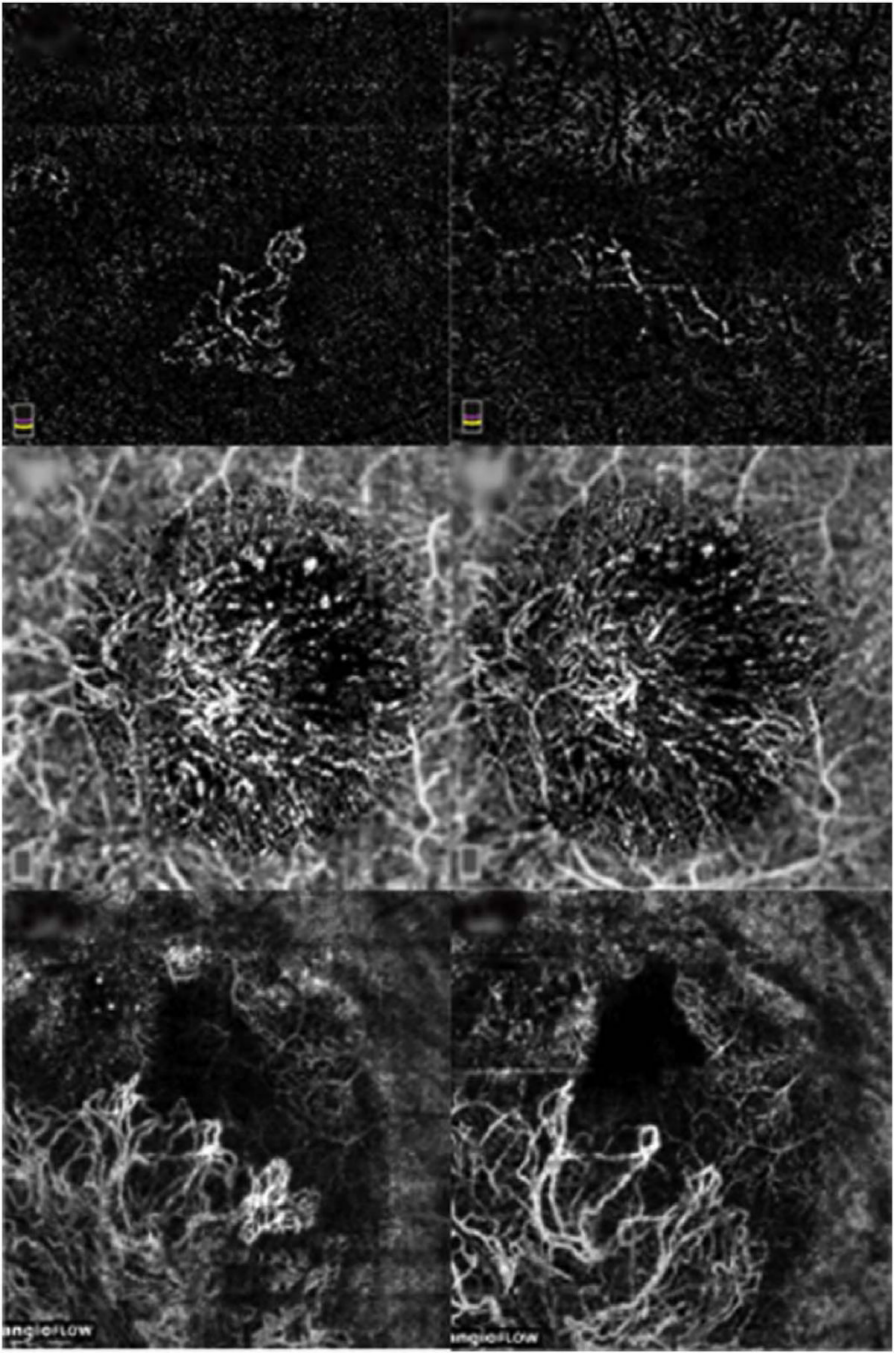
This shows the acute response of MNV 1 month following an injection of antiVEGF agents. A1, B1 and C1 represent pre-injection MNV while A2-C2 represent the corresponding MNVs one month post injection of anti-VEGF agents. The overall size of the MNV shrinks, best noted in A2. The density of the vasculature within the MNV is seen to decrease as are the finer vessels that occupy the margins of the MNV (B2 and C2). Meanwhile, the larger vessels may become larger and occupy more of the lesion. Also note the choriocapillaris loss surrounding the MNV.

Care should be taken in evaluating OCTA images for MNV to avoid mistaking areas of atrophy for neovascularization (Fig. 58). As a rule of thumb the en face images should be evaluated in the context of the Bscan images from which it was derived to assure the intended layer is imaged. Projection artifacts from the retina to retinal elevations such as serous pigment epithelial detachments, drusen, drusenoid pigment epithelial detachments, and pigmented scars, to name a few, can superficially appear like neovascularization.

#### 11.6.3. Follow-up of MNV with treatment

**Fig. 59.**
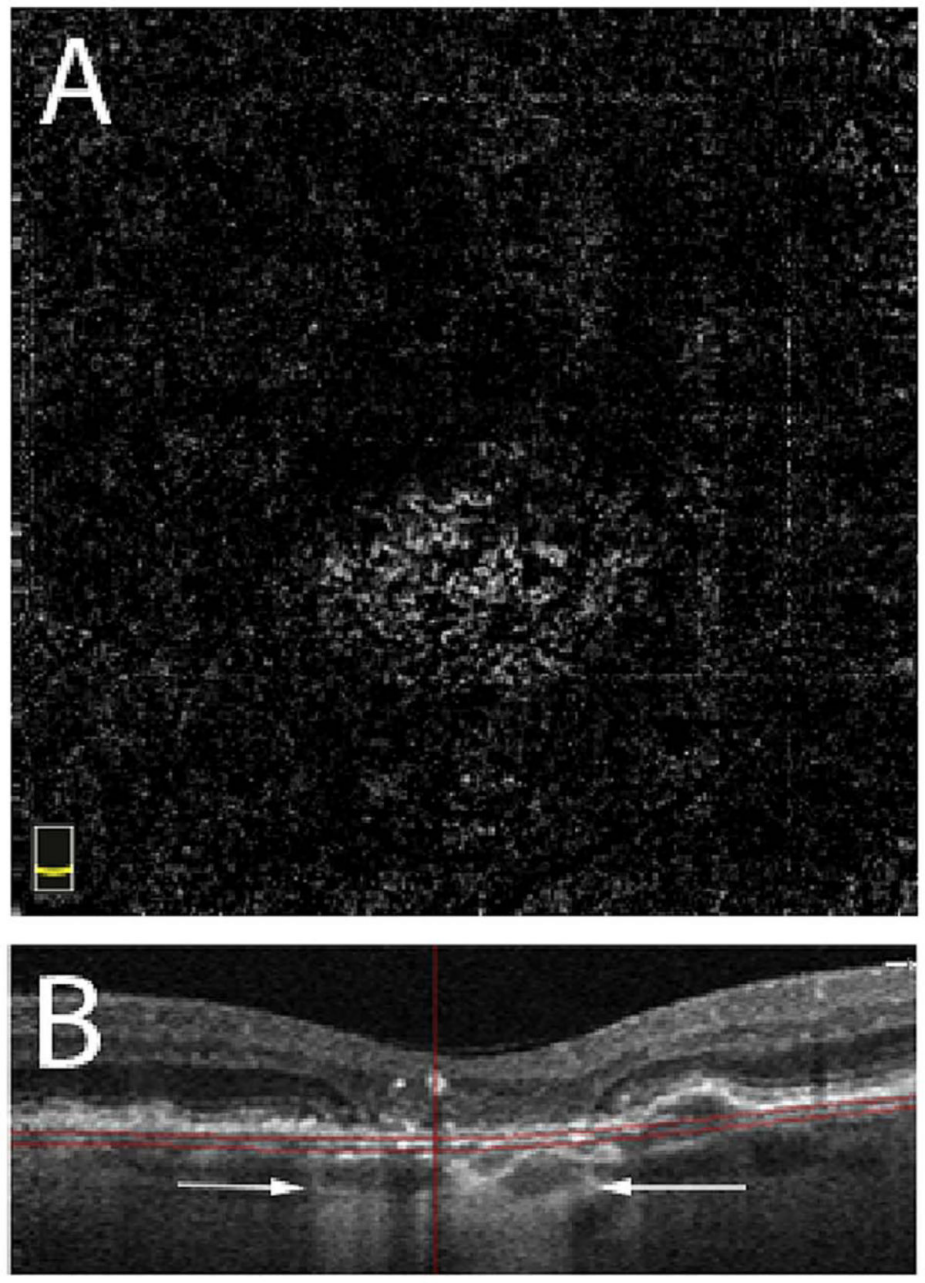
Artefactual appearance of MNV as a result of atrophy. A loss of the choriocapillaris as a result of atrophy (demarcate by the arrows in B) and the resultant superficial migration of the larger choroidal vessels into the area normally occupied by the choriocapillaris gives the appearance of an MNV (A) when segmentation is performed at the choriocapillaris level (red line).

Because OCTA allows for the quantitative and qualitative evaluation of MNV, it is a powerful tool for the monitoring and follow-up of MNV (Fig. 59). After injection with anti-VEGF agents, MNV has been shown to both decrease in size as well as to lose some of the finer, fibrillary vessels at the edges and within the substance of the MNV (Lumbroso et al., 2015; Marques et al., 2016). In a series of patients serially followed up with OCTA, Rispoli, Lumbroso and group have shown that the MNV shows maximal regression at about 1–2 weeks post treatment and starts increasing in size thereafter, while fluid accumulation lags behind lesion growth (Lumbroso et al., 2016). Thus OCTA can be used to monitor treatment response to MNV. It is also intriguing to wonder whether the regression-recurrence patterns seen on OCTA could point to the higher efficacy of a lower dose, more frequent dosage regimen as has been seen in various cancers.

#### 11.6.4. Long term follow-up and future directions

OCTA has enabled us to understand longitudinally the morphologic changes that occur in MNV. In a study about MNV, Spaide et al. proposed, based on OCTA findings, the vascular ‘abnormalization’ occurs as a response to recurrent periodic anti-VEGF therapy (Spaide, 2015a). It is hypothesized that this abnormalization is a result of the periodic pruning of smaller vessels in response to recurrent anti-VEGF therapy as shown in numerous short term follow-up OCTA studies of MNV. Vessels with pericyte coverage remain and are still faced with the forces of arteriogenesis, which is VEGF independent. Over time, this pruning, in the face of unimpeded arteriogenesis, allows for the persistence of large vessels with high flow. The vessels have few branch points and many vascular anastomotic connections among larger vessels (Bellou et al., 2013). The hydrostatic pressure in the shunt vessels is higher than what would be expected in a normal network showing fractal branching patterns and as a consequence any proliferating capillary would be exposed to comparatively high hydrostatic pressure. This may be one of the reasons that periodic recurrent therapy is needed with anti-VEGF agents. It is possible that the larger vessels may eventually be the targets of newer drugs. This is borne by quantitative OCTA flow studies that show high flow in the trunk vessels approaching that of retinal vessels, and that show persistence of high flow in these trunk vessels even in the face of therapy with anti-VEGF agents (Moult et al., 2016).

**Fig. 60.**
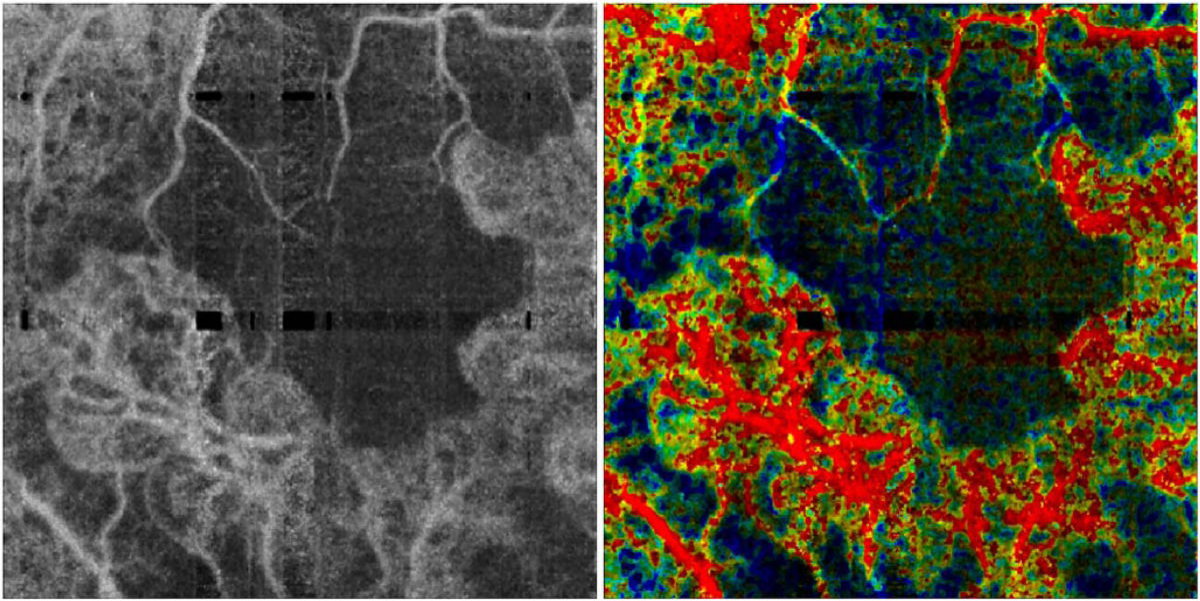
Variable interscan time analysis for blood flow speed shows high speeds of flow approximating that of the larger retinal vessels in the larger trunk vessels of the MNV (seen in red) and slower flow speeds (yellow and blue) in the smaller vessels and in the marginal vessels of the MNV.

The current iterations of OCTA instruments show a limited dynamic range for flow velocity, with the output saturating at low flow rates. Being able to quantify flow rates may be helpful in more accurately identifying MNV and its response to treatment. (Fig. 60).

### 11.7. Optical coherence tomography angiography in myopic MNV

High myopia is a major cause of vision impairment and its prevalence has rapidly increased in the past 50 years (Morgan et al., 2012). In eyes with high axial length the progressive posterior segment elongation and deformation may lead to the development of several lesions, including myopic choroidal neovascularization (MCNV), lacquer cracks, macular retinoschisis, chorioretinal atrophy and posterior staphyloma (Flores-Moreno et al., 2013; Morgan et al., 2012; Ohno-Matsui et al., 2011). These degenerative changes in the sclera, retina and choroid are considered the hallmarks to define high myopia as pathologic myopia (Avila et al., 1984). MCNV has a poor prognosis in the preservation of central vision and can be diagnosed using several imaging techniques.

The current standard to diagnose and evaluate MCNV is to use of FA associated with SD OCT (Leveziel et al., 2013; Neelam et al., 2012). SD OCT revealed MCNV as hyper-reflective material above the RPE, with or without the presence of sub-retinal fluid and intraretinal cysts. On FA, active MCNV appears as well defined hyperfluorescent lesion with typical leakage in the late phase of the examination as classic or type 2 MNV pattern. However, FA is invasive and results in varying degrees of patient discomfort, including nausea, vomiting and anaphylactic reactions. ICGA is another imaging technique which provides valuable details of choroidal vasculature in highly myopic eyes because of its longer wavelength fluorescence with limited diffusion within the choriocapillaries compared to FA (Ohno-Matsui et al., 1998; Quaranta et al., 1996).

**Fig. 61.**
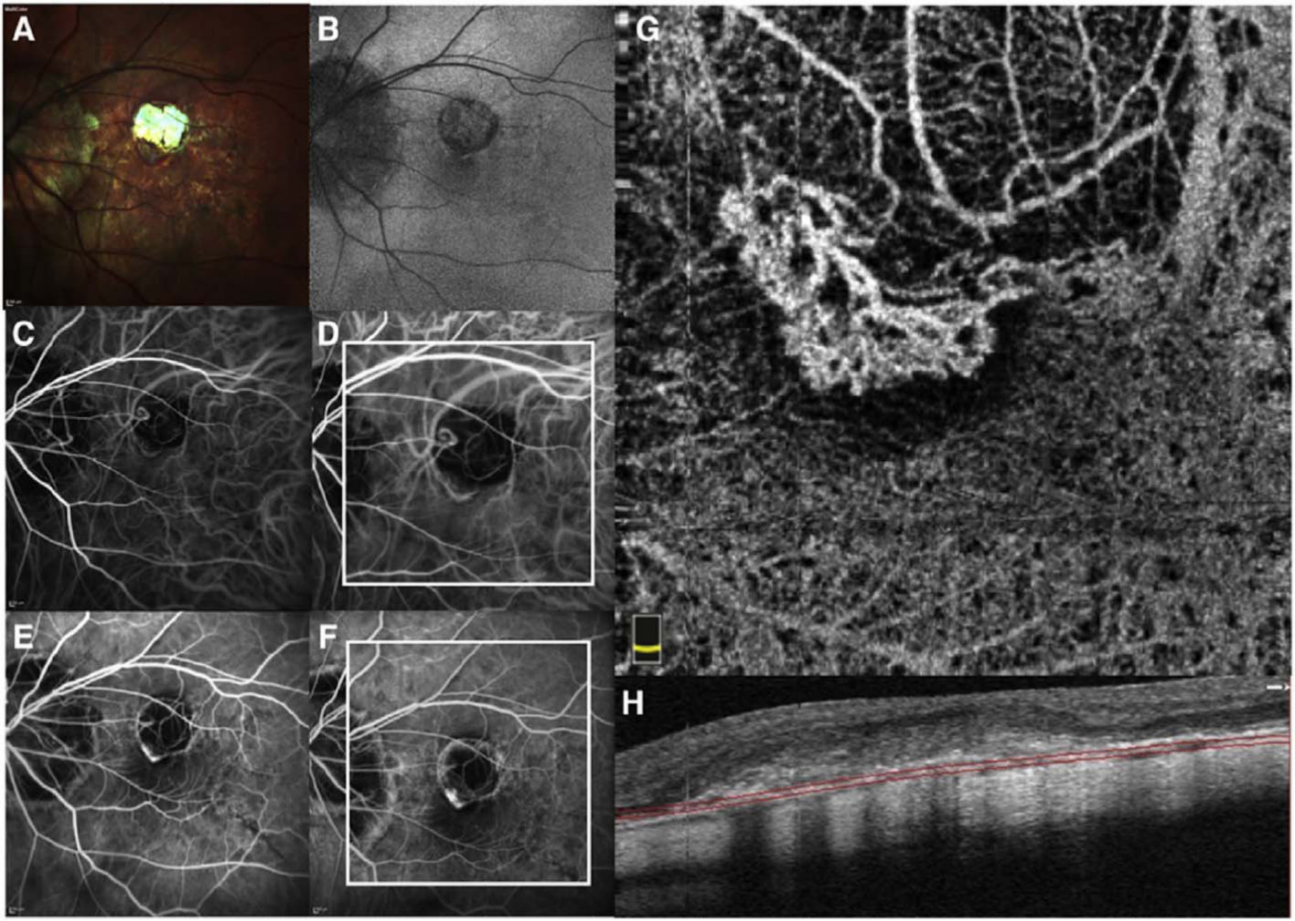
Multimodal imaging of a patients with myopic choroidal neovascularization. MultiColor imaging of a patient with pathologic myopia shows white areas of focal chorioretinal atrophy (A). Blue light fundus AF shows a hypoautofluorescent area (B). Early phase of indocyanine green angiography (ICGA) (C) and FA (FA) (E) reveal an hyperfluorescence area that become more intense with moderate leakage in the late phase (D and F, respectively), as type II classic active choroidal neovascularization. En face OCT angiography section (G) just below retinal pigment epithelium (H) shows type II neovascular network with well circumscribed appearance.

While ICGA image collects all the fluorescence coming from the entire retina, OCTA shows only the layer sampled. Vessels not perfused are not visible in OCTA but are seen in the structural OCT and are indirectly visible in ICGA as masking tissue over the normal choroidal vasculature. OCTA shows MCNV as a loose lacy group of filamentous vessels and few large branches or a tortuous capillary network with many hyperdense vascular ramifications above RPE (Fig. 61). To obtain the best view of the vascular network, the slab segmentation should be manually just above the RPE band. Pathologic myopia is often characterized by posterior macular staphyloma, irregular surface, RPE atrophy, choroidal thinning and consequently highest scleral reflectivity which are the causes of difficult automatic segmentation. To overcome errors in segmentation creating poor en face images, manual B-scan segmentation should be used.

One further typical finding in pathologic myopia is the presence of patchy chorioretinal atrophy at the posterior pole. In areas of patchy atrophy OCTA flow can be seen in the underlying larger choroidal vessels, in contrast to the adjacent areas having RPE and choriocapillaris coverage where both SD and SS-OCT based methods have difficulty in demonstrating flow. Projection artifacts may also limit the accuracy of differentiating between neovascularization and atrophy. With some instruments it is possible to use the scroll wheel on the mouse to move the segmentation up and down to help differentiate atrophy from neovascularization. OCTA in evaluating myopic eyes is not a standalone imaging test. It is part of a larger multimodal imaging approach that can better characterize MCNV and help the treatment decision.

### 11.8. OCT angiography in angioid streaks

Angioid streaks (AS) are more commonly found such in patients with pseudoxanthoma elasticum, Ehler-Danlos syndrome, Paget’s disease of bone, sickle cell disease, and thalassemia; in some cases angioid streaks are thought to be idiopathic (Aessopos et al., 1994; Dabbs and Skjodt, 1990; Gliem et al., 2013a; Green et al., 1966).^279−282^ Of these pseudoxanthoma elasticum is probably the most common and its associated ocular findings classically include AS, optic nerve drusen, peau d’orange, areas of subretinal fluid prior to the development of MNV, a pattern dystrophy appearance with eventual RPE atrophy, and finally CNV (Yang et al., 2003b).

Angioid streaks (AS) are angular branching breaks in Bruch’s membrane rendered brittle because of abnormal thickening and calcification. AS appear as irregular reddish or brownish lines of varying width with jagged borders (Gliem et al., 2013a). The cracks in Bruch’s membrane occur in regions where there appears to be confluent calcification as opposed to spotty calcification of Bruch’s membrane, which creates the appearance of peau d’ orange (Spaide, 2015b). The rupture of Bruch’s membrane causes associated breaks in the overlying RPE monolayer. If the AS passes directly under the fovea, a patient may develop decreased or distorted vision. A major cause of visual impairment in these patients is the development of CNV, typically Type 2, leading to retinal hemorrhage, edema, and a fibrotic scar with permanent corresponding visual loss (Gliem et al., 2013b). Patients with pseudoxanthoma elasticum with AS may also develop areas of RPE loss around the streaks.

Because of the concurrent abnormalities, imaging modalities such as FA, ICGA and structural OCT produce ambiguous images that preclude making a diagnosis of early CNV with certainty. Since the eyes have a pattern dystrophy including both hyperpigmentation and RPE loss in conjunction with AS, the FA may show widespread abnormalities in the posterior pole that can obscure the presence of early coexistent CNV. With ICGA there are also confounding factors such as the intrinsic hyperfluorescence of streaks which is sometimes present and the diffuse irregularities that reduce contrast between the lesion and the surrounding choroid. Structural OCT shows undulation of the posterior pole, large ruptures of rupture of Bruch’s membrane, vitelliform accumulations, shifting areas of subretinal fluid without the presence of MNV, and regions of thickening at the level of the RPE associated with the pattern dystrophy.

**Fig. 62.**
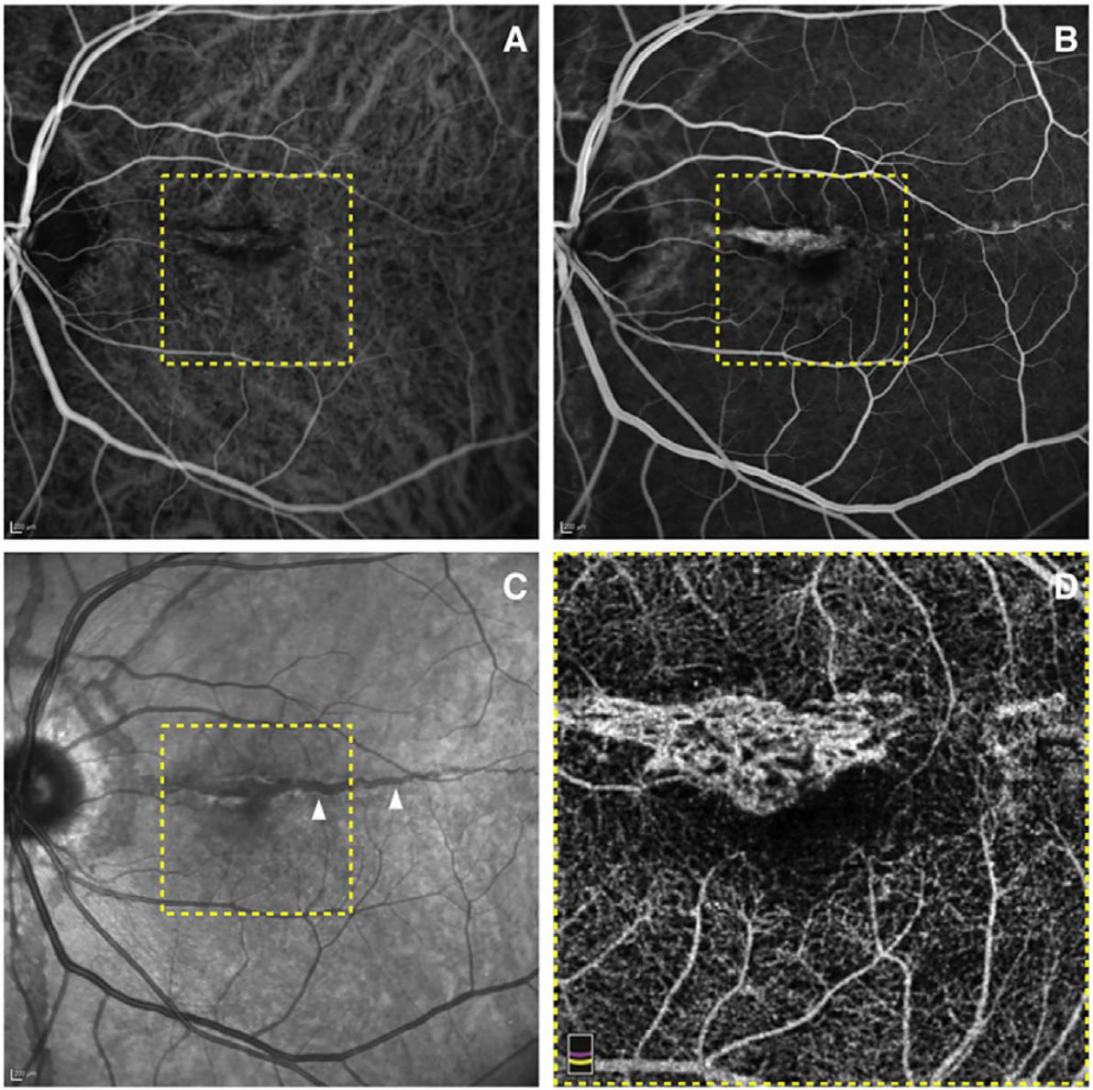
Multimodal imaging of a patient with angioid streak complicated by choroidal neovascularization. ICGA (A) and FA (B) reveal the light hyperfluorescence corresponding to the angioid streak as the results of the rupture of Bruch’s membrane and the hyperfluorescence of the choroidal neovascularization. Infrared reflectance (C) displays the angioid streaks as linear hyporeflective area starting from optic nerve head. Optical coherence tomography angiography (D) shows macular neovascularization with a network that closely followed the trajectory of angioid streak.

OCTA, due to its ability to produce high contrast retinal and choroidal vascular network images, can show the CNV in eyes with AS (GalOr et al., 2015) (Fig. 62). The spatial relationship between Bruch’s membrane and CNV can be precisely studied using OCTA where seems to be a close relationship between the morphology of Bruch’s membrane defects and the topographic pattern of MNV. Deeper than Bruch’s membrane is the choriocapillaris, which shows profound abnormalities even prior to observable atrophy or CNV (Spaide, 2017a).

### 11.9. Optical coherence tomography angiography of ocular inflammatory disease

OCTA is a useful imaging tool in chorioretinal inflammatory diseases. While there are many potential applications, currently two have the greatest clinical utility. First is the ability to examine the retinal circulation for signs of compromise. Examination of the retinal vasculature traditionally has been done with FA by which inflammatory damage of and fluorescein leakage from the higher order retinal arteries and veins are readily visible. However fluorescein leakage obscures imaging of smaller vessels such as the capillaries and the deeper vascular plexus is not visible in FA even in normal eyes (Spaide et al., 2015b). OCT angiography can’t detect leakage, which is a weakness in a sense, but also is a strength in that imaging of the retinal microvasculature is not impeded by leakage. In addition, OCT angiography can image the deep vascular plexus. The second main use of OCT angiography is to evaluate patients for secondary CNV.

#### 11.9.1. Vascular damage associated with uveitis

**Fig. 63.**
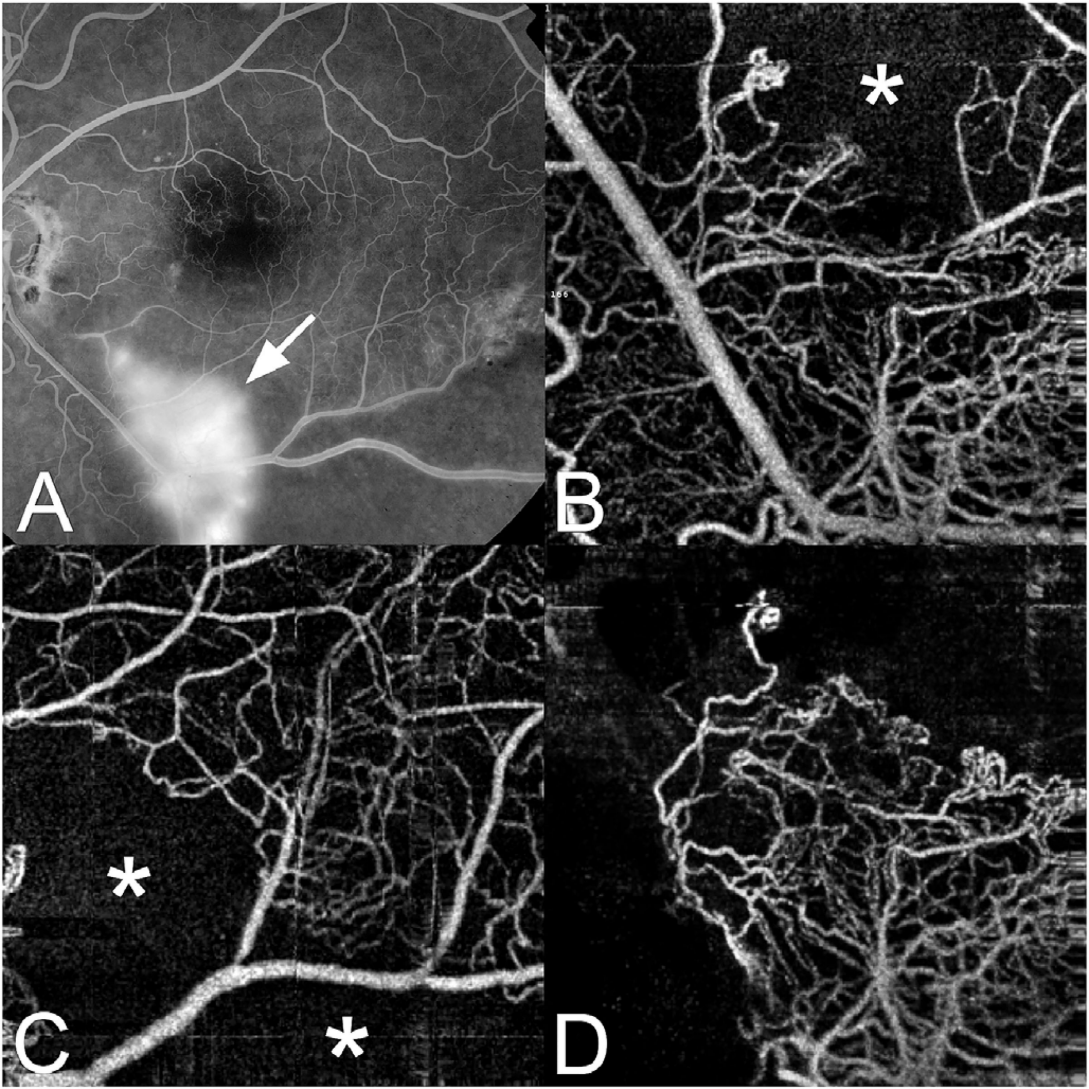
Vascular occlusion in retinal vasculitis with subsequent retinal neovascularization. (A) The most salient feature of the fluorescein angiogram is the area of intense hyperfluorescence from retinal neovascularization (arrow). Temporal and inferior to this are areas seemingly devoid of smaller branch vessels. (B) The OCT angiogram at the level of the retina shows blunted branch vessels and regions of non-perfusion (asterisk). (C) More pronounced pruning is evident with multiple areas of non-perfusion (asterisks). (D) OCT angiography set above the level of the retina shows the retinal neovascularization.

By its nature, intraocular inflammation requires recruitment of various components of the immune system to form an attack against a perceived abnormality. In infection the body’s produces a coordinated and orchestrated attack against a foreign invader. Inflammation appears as an undesirable manifestation in non-infectious uveitis, in that situation the body mounts an attack against some component in the eye. Inflammatory infiltrates around vessels, vasculitis, may occur in association with a systemic vasculitis (Androudi et al., 2013) or occur as an isolated ocular event, which in turn may be related to infectious or non-infectious causes (El-Asrar et al., 2009; Perez et al., 2004; Rosenbaum et al., 2012; Talat et al., 2014; Walton and Ashmore, 2003). Inflammation that primarily involves the retinal arterioles is associated with conditions such as giant cell arteritis, polyarteritis nodosa, granulomatosis with polyangiitis (Wegener’s granulomatosis), and systemic lupus erythematosus. The retinal veins are principally affected by clinically evident inflammation in association with sarcoidosis, multiple sclerosis, tuberculosis, and intermediate uveitis (Arnold et al., 1984; Engell and Andersen, 1982; Green et al., 2010; Hsu et al., 2001; Iida et al., 2002). As in systemic vasculitides, inflammation of retinal vessels can be associated with vascular occlusion (Fig. 63). Inflammation affecting the arterioles, for example, may be associated with arterial occlusion while retinal phlebitis may lead to veno-occlusive disease. OCT angiography can demonstrate larger vascular occlusions as well as retinal neovascularization that can grow in response to consequent ischemia.

**Fig. 64.**
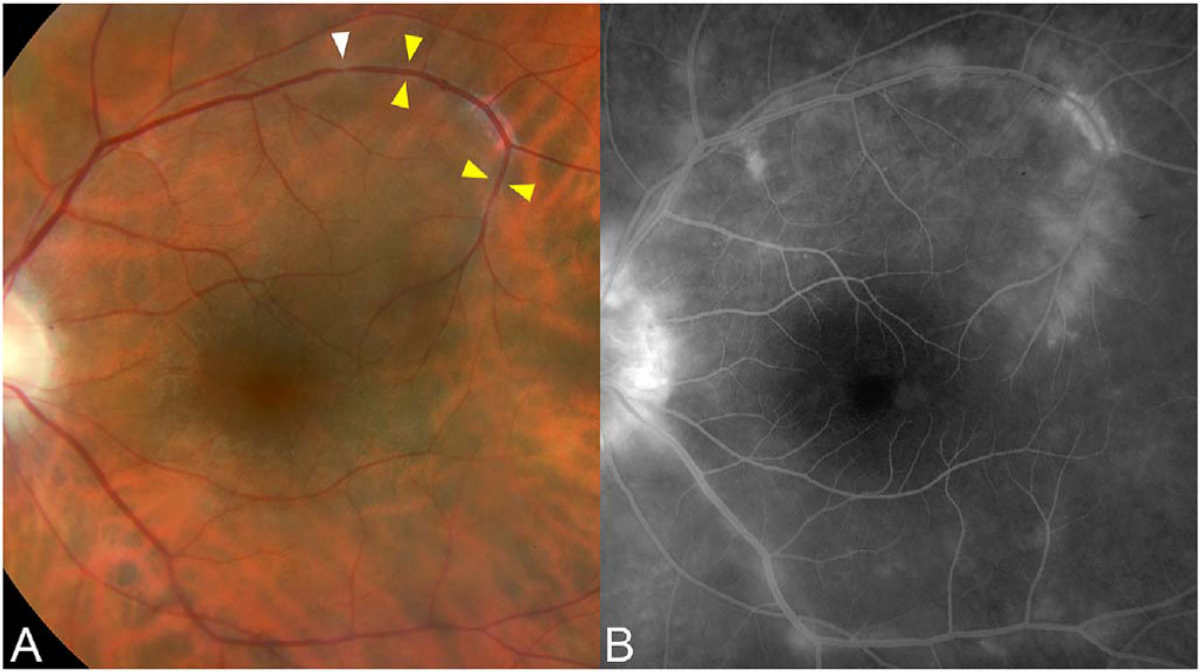
A 49-year-old HLA-B27 + woman with ankylosing spondylitis. (A) There is subtle sheathing of the vein in the superotemporal arcade (yellow arrowheads), and areas of focal caliber changes (highlighted by white arrowhead). (B) FA shows perivascular leakage and staining extending variable distances from this vein as well as other vessels. The fellow eye showed widespread leakage and staining around larger vessels as well.

**Fig. 65.**
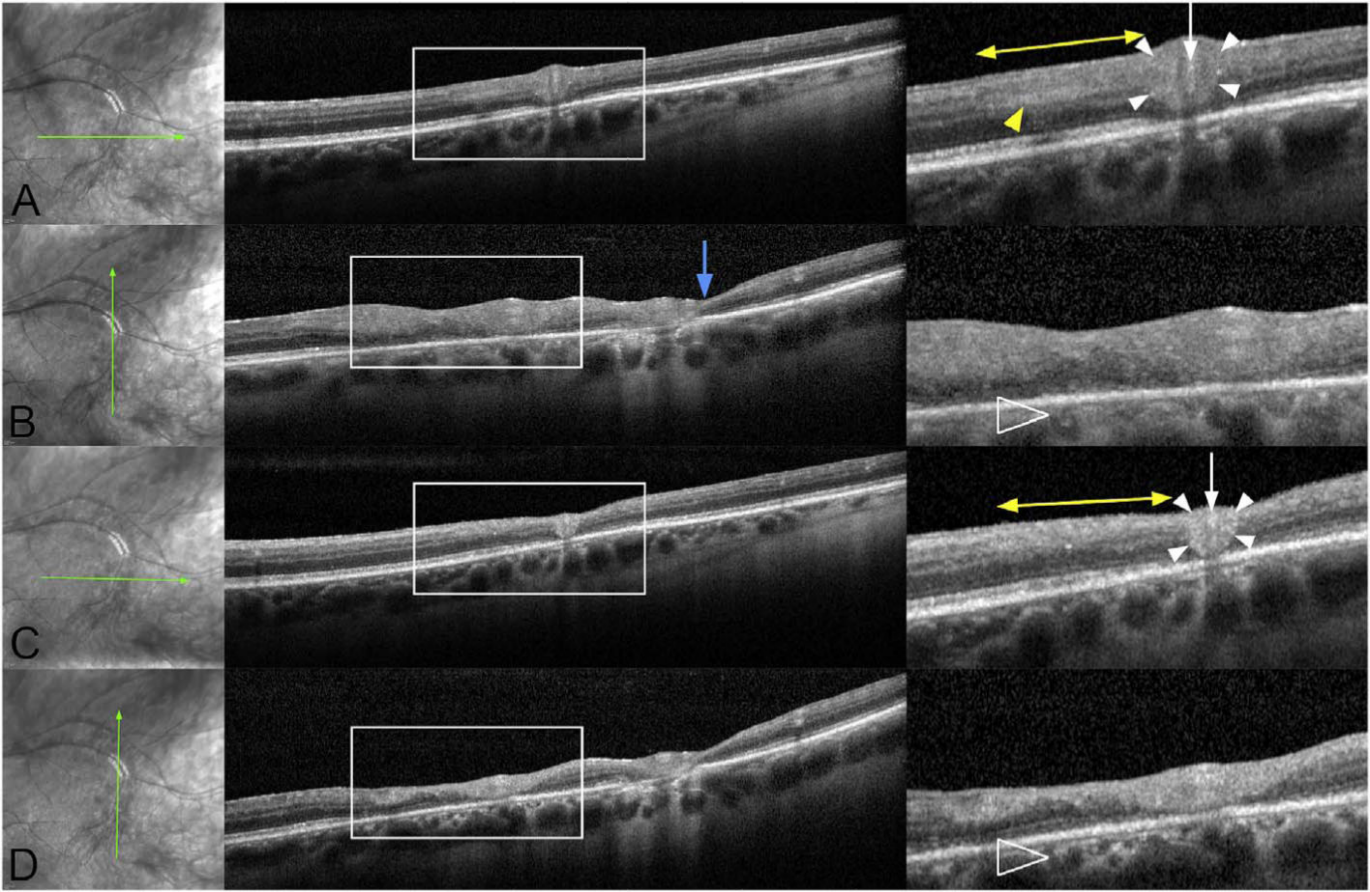
Optical coherence tomography (OCT) findings of perivascular infiltration before and after treatment. Along each row of images, the left panel shows an infrared scanning laser ophthalmoscopic image showing the scan location, the middle panel shows the entire OCT scan, and the right panel shows a highlighted area as demarcated by the white rectangle in the middle panel. (A) Horizontal OCT section through an involved segment of the superotemporal arcade vein. There is an annular zone of increased perivascular reflectivity (arrowheads) around a vein (arrow). Adjacent to this is a region of loss of the laminations of the retina (double arrow). Contained within this region is a zone of increased reflectivity of the inner nuclear layer. It is possible this could be called paracentral acute middle maculopathy, but this term ignores the associated changes in other layers of the retina and the area imaged is not within the anatomic macula. (B) A scan perpendicular to that shown in (A) shows the variable thickening of the retina in the region of loss of laminations. Note the focal area of thinning (blue arrow). The choroid shows increased reflectivity and a loss of visible structure. (C) One week after intravitreal triamcinolone the retina shows thinning in the region of the double arrow. The perivascular reflectivity is thinner (arrowheads) around the retinal vein (arrow). (D) An OCT scan taken perpendicular to (C) shows the thinning

**Fig. 66.**
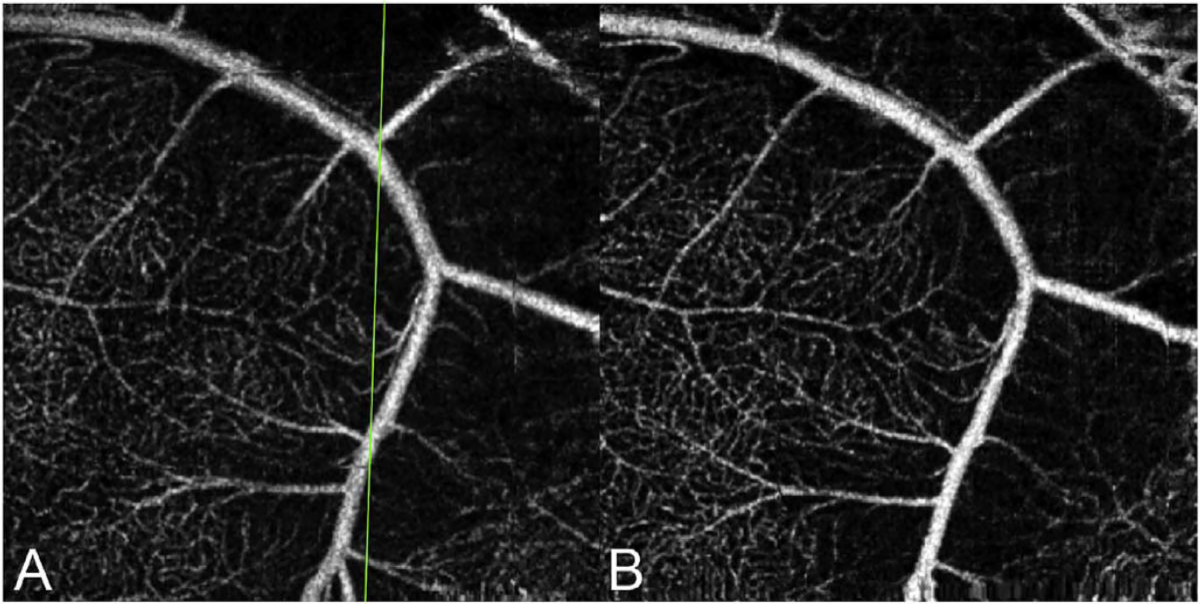
OCT angiograms of affected area in the patient shown in Fig. 64 and 65. (A) The green line corresponds to the OCT sections shown in Fig. 65. Note the poor perfusion, particularly to the right side of the vein. (B) One week after intravitreal triamcinolone the perfusion showed no significant change. Reprinted from Spaide (2017c). as compared with (B). Note the decrease in the choroidal reflectivity with enhanced visualization of choroidal details (open arrowhead). Reprinted from Spaide (2017c).

**Fig. 67.**
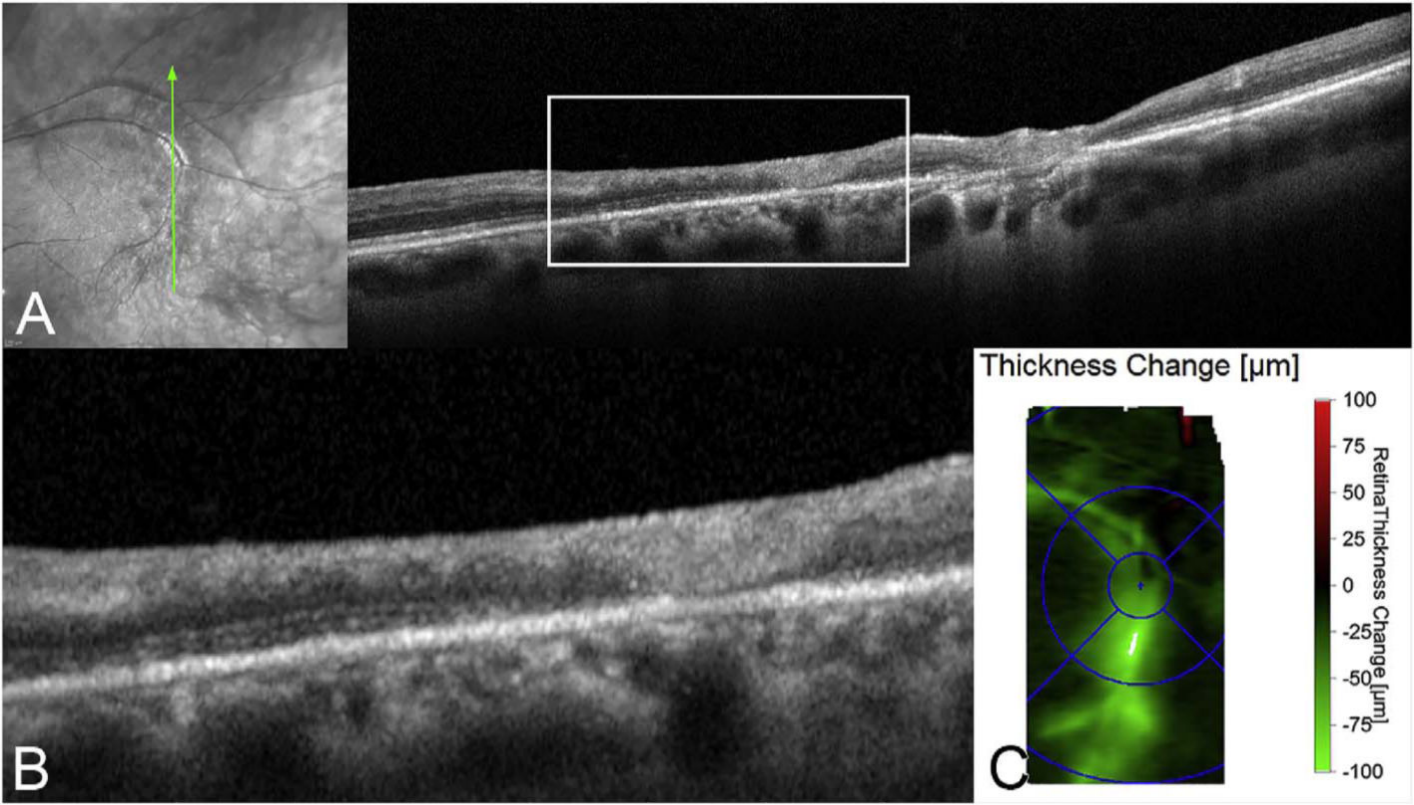
One month after intravitreal triamcinolone. (A) Left panel shows the location of the scan, which is the same as Fig. 65 B and D. The right panel shows continued retinal thinning. (B) Enlarged depiction of the area enclosed by the rectangle in (B). (C) A heat map of the change in retinal thickness from prior to intravitreal triamcinolone. The area of retinal thinning as shown by the heat map is similar to the region of fluorescein leakage and staining seen in Fig. 64 B. Reprinted from Spaide (2017c).

Occlusion of arteries or veins axiomatically would have secondary effects on associated capillaries. A group of patients with clinically evident inflammatory changes involving large vessels, but no frank occlusion of any higher order vessels, were found to develop the OCT appearance in the inner and middle layers of the retina, simulating that seen in retinal ischemia, which then was followed by retinal thinning of the same layers (Spaide, 2017d). These patients had no evidence by fluorescein or OCT angiography of larger vessel occlusion. Indeed the main circulatory anomaly they appeared to have were widespread capillary perfusion abnormalities in both the superficial plexus and deep vascular plexus, but not the usual stigmata of cotton-wool spots or deeper areas of ischemic retinal whitening. Given the diagnosis of retinal vasculitis was made because of perivascular inflammatory infiltration accompanied by fluorescein leakage and staining, capillary problems distant to the larger retinal vessels implies potential for retinal damage in excess of what would be intuitive to expect on the basis of ophthalmoscopy (Figs. 64–67).

Inflammatory attack of vessels can occur through a variety of mechanisms. Infection causes vasculitis by the trafficking of immune cells to sites of infection or direct attack of the infectious organism on blood vessels. Common modes include Type I, II, II, and IV hypersensitivity reactions with or without subsequent intravascular thrombosis. The retina seems susceptible to damage by another mechanism, capillary leukostasis. Release of inflammatory mediators such as tumor necrosis factor alpha (TNFα), interleukin (IL) 1β, and IL-4 among many others lead to expression of various cell markers such as E- and P- selectin, vascular cell adhesion molecule (VCAM) −1, leukocyte adhesion proteins chemokine (C-X3-C motif) ligand 1 (CX3CL1), and intercellular adhesion molecule (ICAM), which bind to leukocyte cell receptors (Fong et al., 1998; Kim et al., 2001; Krieglstein and Granger, 2001; Lipowsky and Chien, 1989; Simon and Goldsmith, 2002; Steeber et al., 1999; Tailor and Granger, 2000; Von Andrian and Arfors, 1992; Zarbock and Ley, 2009). As part of the inflammatory cascade, binding of leukocytes leads to cytoskeletal rearrangement, stiffening, and alteration on the blood flow in the vessel (Akenhead et al., 2015; SchmidSchönbein, 1993). Diapedesis of leukocytes usually occurs in the postcapillary venules (Sainte-Marie et al., 1967; Steeber and Tedder, 2000; Vestweber, 2015), but in a number of pathologic situations leukocytes may occlude capillaries. Leukocyte adhesion and leukostasis involved with ischemia-reperfusion injury in many types of tissue, and also has been implicated in inflammatory damage to the brain, liver, lung, muscle, and kidney (Amantea et al., 2009; Gane and Stockley, 2012; Gute et al., 1998; Hokama et al., 2000; Ishikawa et al., 2016; P Basile and C Yoder, 2014; Tanaka, 2016; Yadav et al., 1999). More relevant to the retina is the finding that leukostasis is a major component in the development and progression of diabetic retinopathy (Joussen et al., 2002, 2004). The retina may be particularly susceptible to ischemic damage because of its inherently high metabolic rate, its segmental, non-redundant blood supply distribution, and the lack of regenerative capabilities of the constituent terminally differentiated cells. As a mechanism of injury, microvascular occlusion in retinal vasculitis may be important and needs to be studies in larger patient series. Early treatment of retinal vasculitis may be in order, if only to prevent retinal loss associated with microvascular flow abnormalities.

#### 11.9.2. Choroidal neovascularization

Multifocal choroiditis and panuveitis is more commonly found in young myopic women. (For the purposes of discussion punctate inner choroidopathy will be considered a subset of multifocal choroiditis and panuveitis) (Spaide et al., 2013). There are two main ways the disease can cause damage, through inflammation or choroidal neovascularization. The inflammation is chiefly manifested as subretinal and subretinal pigment epithelial inflammatory infiltrates. These appear as conical hyperreflective mounds that elevate the retinal pigment epithelium (RPE) and can break through to the outer retina. If severe the inflammatory deposit can expand in the outer retina/subretinal space. In the absence of choroidal neovascularization these lesions can leave a legacy of scarring and destruction of the RPE. The ingrowth of choroidal neovascularization can produce somewhat similar findings, although the subRPE and subretinal material may have a greater heterogeneity in reflection in the structural OCT. Both appear to be associated with subretinal fluid. During FA CNV generally shows greater fluorescence and more pronounced leakage than does the inflammatory infiltrate. Chorioretinal scars can show varying amounts of late staining. In many cases it is difficult to be certain if CNV is present or not.

**Fig. 68.**
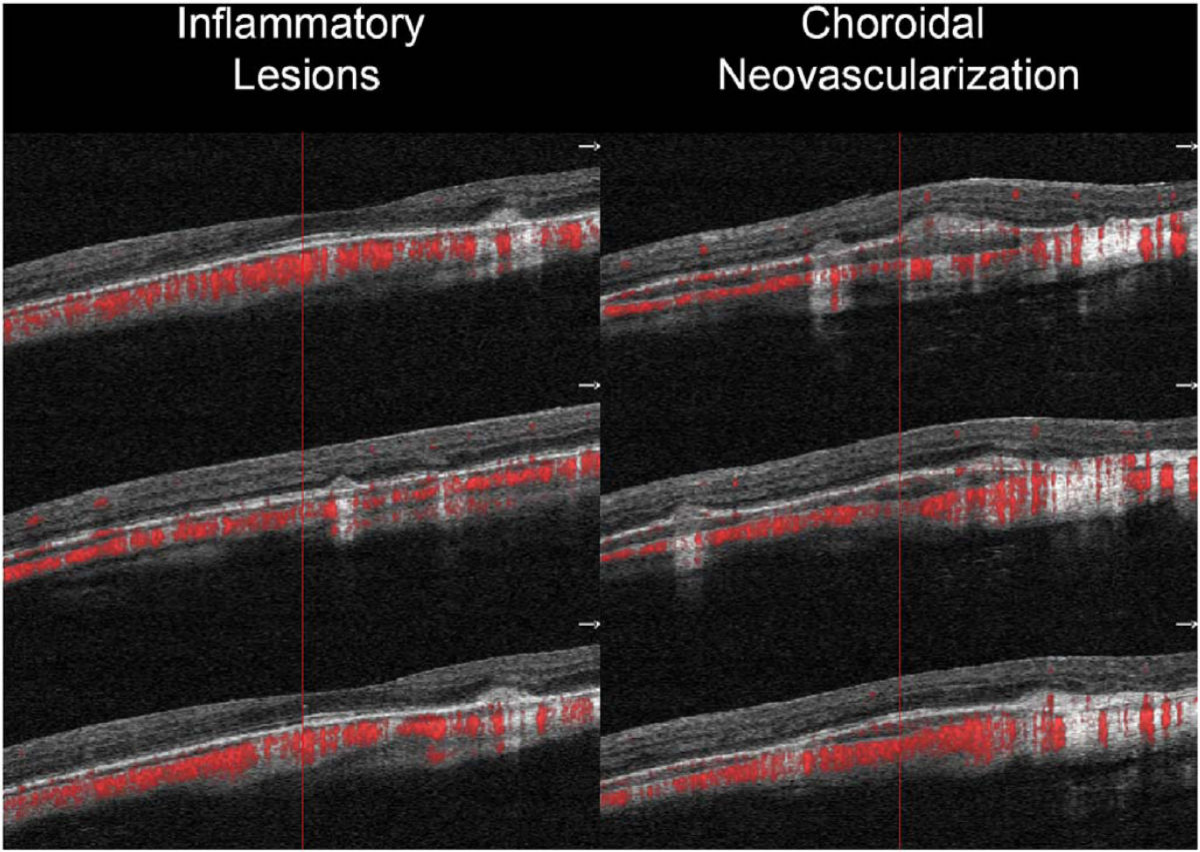
Comparison of presumed inflammatory versus neovascular lesions in multifocal choroiditis and panuveitis. The left column of images shows a conical infiltration of material that is relatively homogenous and has no demonstrable flow. The lesions in the right column show greater heterogeneity, structure, and contain flow signal.

**Fig. 69.**
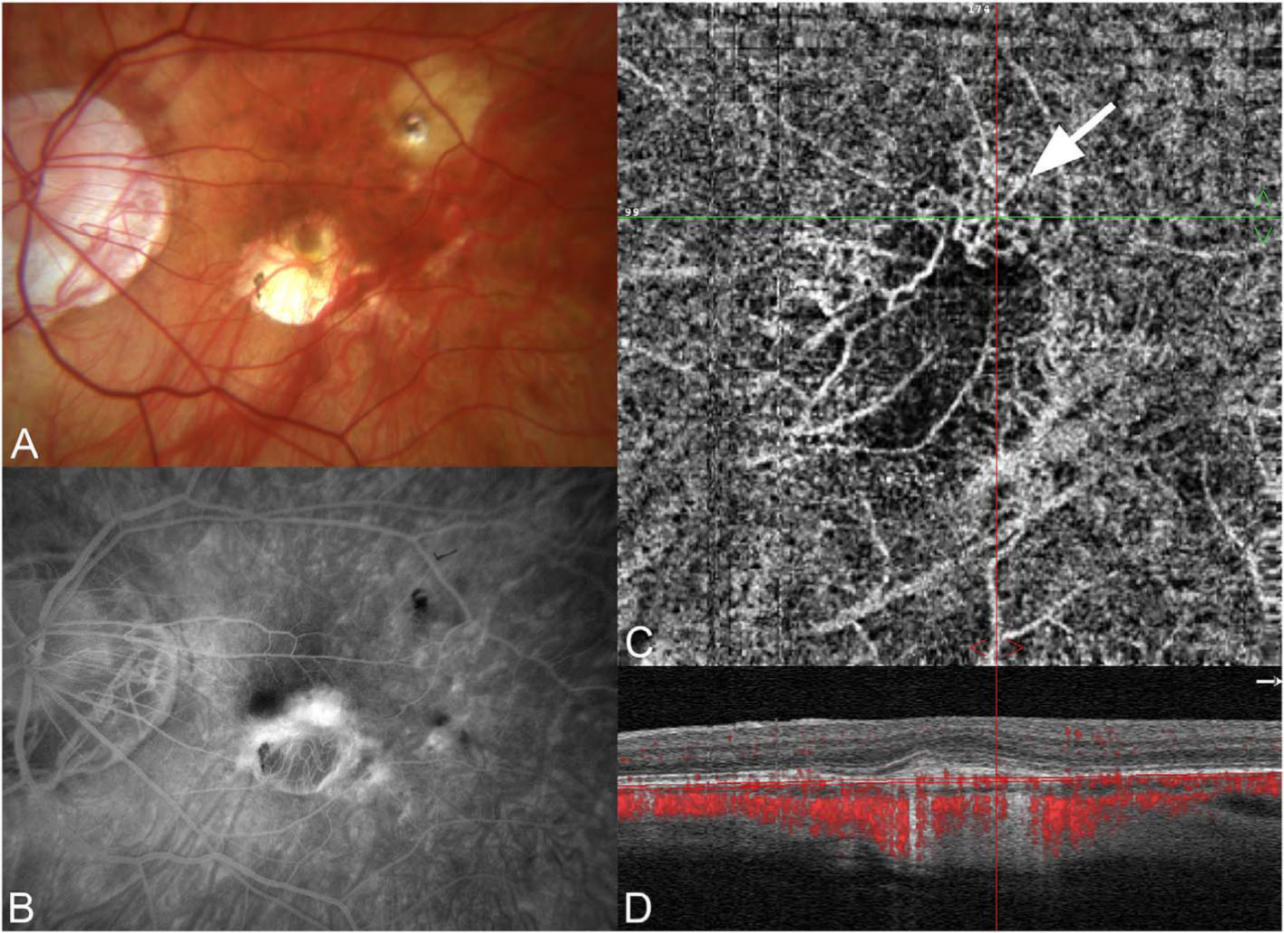
Multifocal choroiditis with chorioretinal scars in a patient previously treated with intravitreal anti-VEGF injections. (A) The patient has 2 visible chorioretinal scars and myopic disc changes. (B) The fluorescein shows late staining around the lesion in the fovea. (C) OCT angiography shows a vessel complex most prominently seen at the superior border of the lesion. (D) B-scan with flow overlay shows a tumefaction containing discrete areas of flow signal, consistent with persistent choroidal neovascularization.

**Fig. 70.**
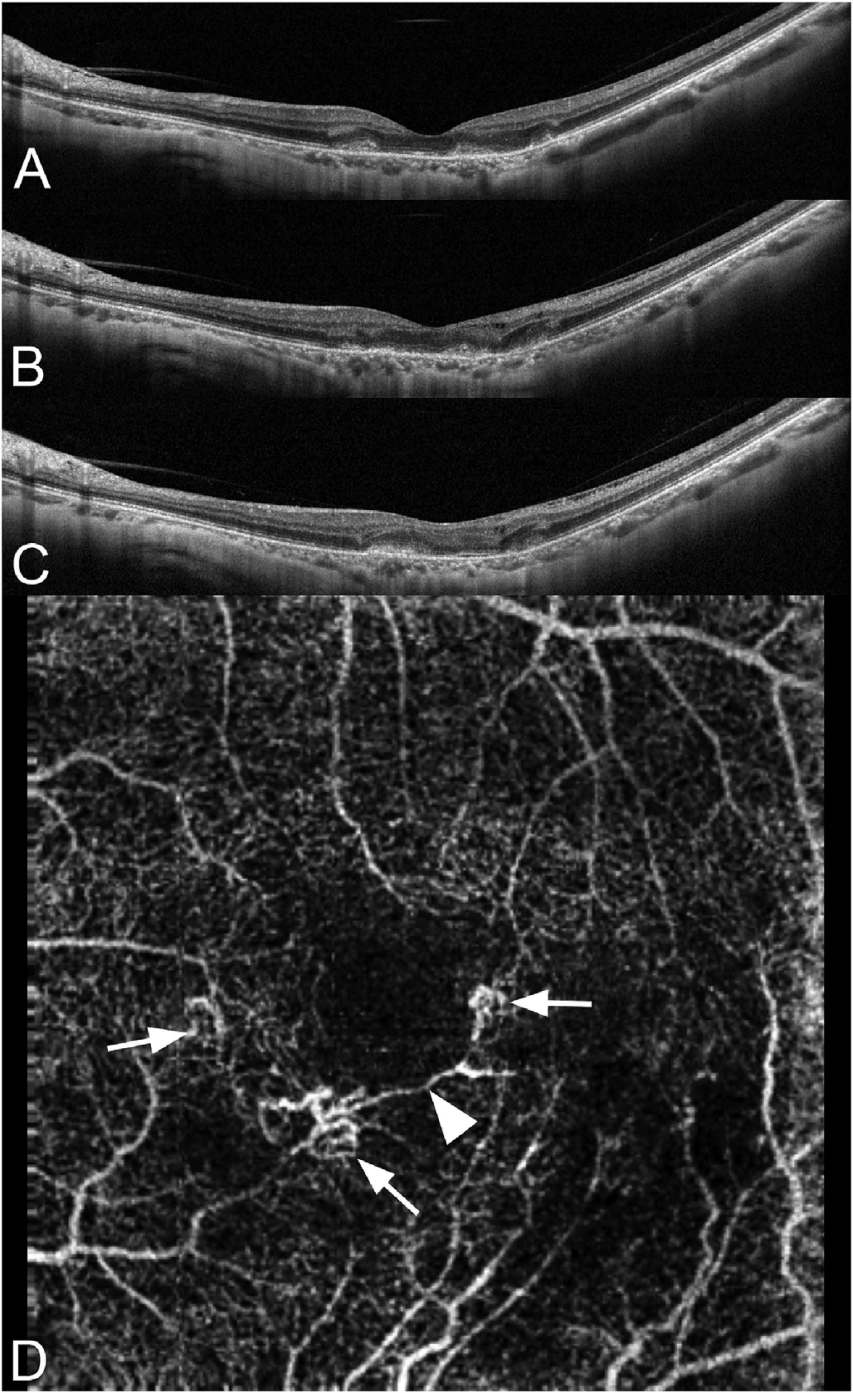
New onset multifocal choroiditis complicated by choroidal neovascularization. A, B, C successive sections showing widespread infiltration of the outer retina with loss of the ellipsoid layer. (D) The OCT angiogram shows discrete areas of CNV, implying the more diffuse deposit is composed of inflammatory collections, exudation from the CNV, or a mixture of both.

**Fig. 71.**
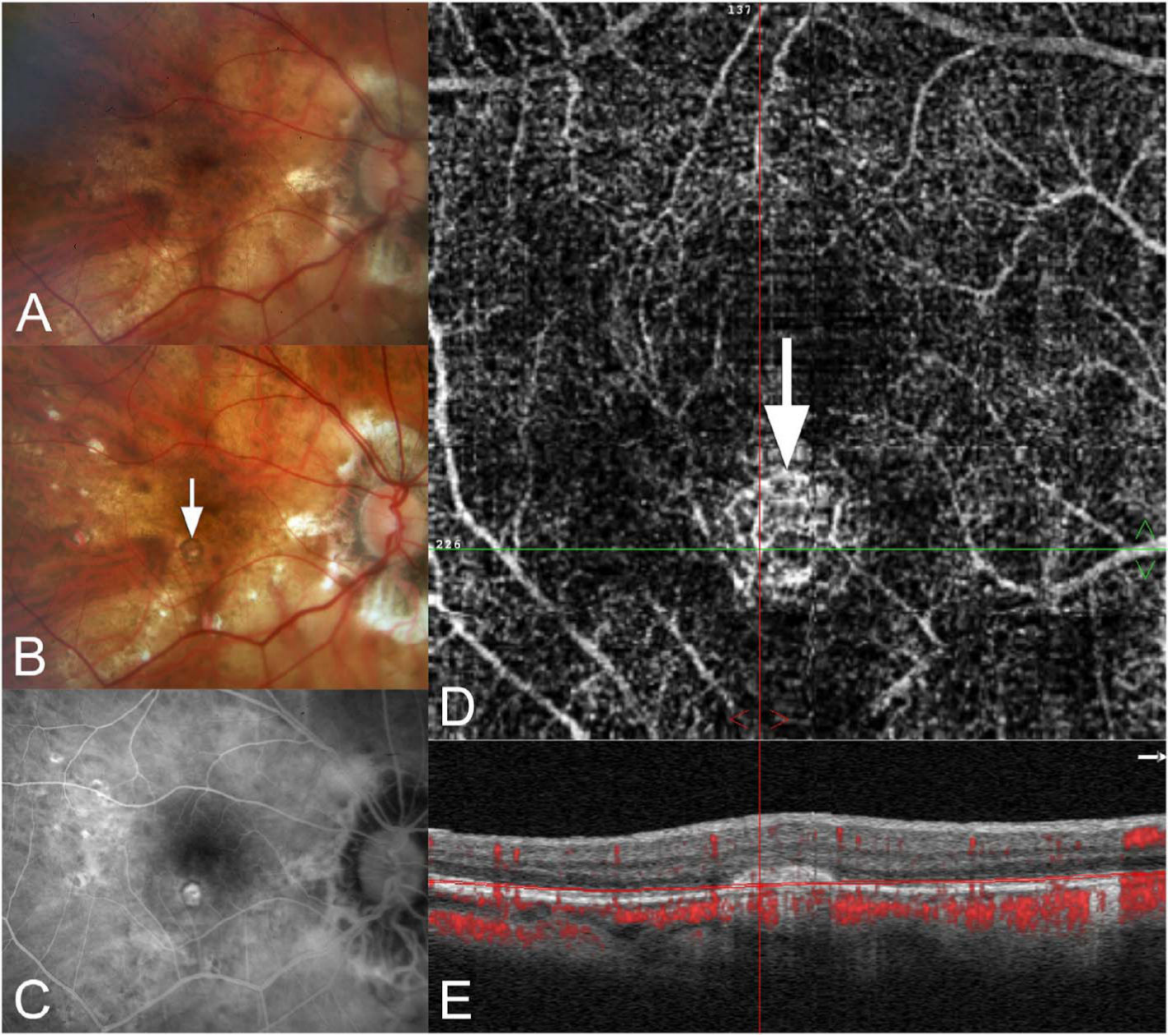
New onset multifocal choroiditis and panuveitis with CNV. (A) This highly myopic patient was seen for her yearly examination. In the past she had surgical repair of a retinal detachment. (B) When seen a year later she had multiple chorioretinal punched out lesions, one of which was ringed by pigment (arrow). (C) FA shows some staining of the pigmented lesion in (B). The depigmented lesions show modest staining. (D) En-face view of OCT angiography shows a lesion containing vessels at the location of the pigmented lesion (arrow). (E) B-scan with flow overlay shows an elevated lesion with internal flow signals that are not projection artifacts from the overlying retina. Because the lesion was pigmented and showed no exudation, the eye was observed with no treatment

OCT angiography appears to be well suited to detect CNV complicating multifocal choroiditis and panuveitis (Levison et al., 2017; Nakao et al., 2016) (Figs. 68–71). Eyes with multifocal choroiditis and panuveitis may have significant amounts of myopia, which generally is a difficulty for present segmentation routines, and therefore produce en-face images that are not reliable. The images from eyes with numerous segmentation defects show flow, but the resultant image is not necessarily of areas relevant for accurate diagnosis or disease quantification. The flow overlay on the b-scan is often the most useful way to evaluate lesions. It is often necessary to scroll through multiple b-scans to achieve a mental picture of the lesions.

Treatment of the CNV is possible with intravitreal injections of antiVEGF agents in some cases. More aggressive disease is not adequately managed by monotherapeutic anti-angiogenic methods because recurrence of the neovascularization is common and frequent. The CNV is driven by the inflammatory component, which is capable of causing damage on its own. Therefore a two pronged approach attacking the neovascularization and the inflammation is typically used. Local corticosteroid injections can help in the acute management of disease. Patients often require years of treatment and to avoid corticosteroid side effects, immunosuppression is used. There have been no published studies concerning the relative efficacy of these medications. A common medication to use is mycophenolate mofetil, in part because of the relatively low proportion of patients exhibiting significant side effects (Thorne et al., 2005). The use of anti-tumor necrosis factor alpha agents is becoming increasingly common (Cordero-Coma and Sobrin, 2015). The goal of treatment is to reduce the inflammatory deposits and prevent exacerbations of the CNV. With successful treatment the lesions regress, sometimes without a trace. With OCT angiography remnants of CNV can still be detected. Immunosuppressive agents have to be withdrawn if pregnancy is contemplated. The inflammatory and often neovascular complications of the disease can be managed with local corticosteroids during this interval. Although FA is not necessarily contraindicated during pregnancy, the ability to image neovascularization without dye is a welcome attribute in this situation.

There are still many aspects of ocular inflammatory disease and the retinal and choroidal vascular changes that remain unknown. OCT angiography offers opportunity to investigate both in ways not possible with dye based angiography. There are numerous areas that could be investigated, particularly once the ability of OCT angiography to image the choroid is improved. Many inflammatory diseases of the posterior pole involve or originate from the choroid. Increased understanding of disease pathogenesis, earlier detection of disease, and improved disease monitoring are achievable goals in the near future.

### 11.10. OCT angiography in dystrophies

#### 11.10.1. Vitelliform macular dystrophy

Vitelliform macular dystrophy (VMD), also known as Best disease, is a slowly progressive disease with onset generally in childhood, is caused by mutations in the BEST1 gene (Best, 1905; Deutman and Hoyng, 2001; Stone et al., 1992). The diagnosis of VMD is based on fundus appearance with yellow yolk-like macular lesion, an abnormal electrooculogram and family history (Godel et al., 1985). The material in the subretinal space starts to accumulate in early childhood. OCT shows varying amounts of subretinal fluid and collection of material on the back surface of the retina and also immediately above the RPE. The outer retina can have a ‘shaggy photoreceptor’ appearance much like that seen in central serous chorioretinopathy. Over the years the material increases in amount, and fills the subretinal space. The lesions generally get larger with age. In the teen years or thereafter, many patients develop scarring and CNV.

**Fig. 72.**
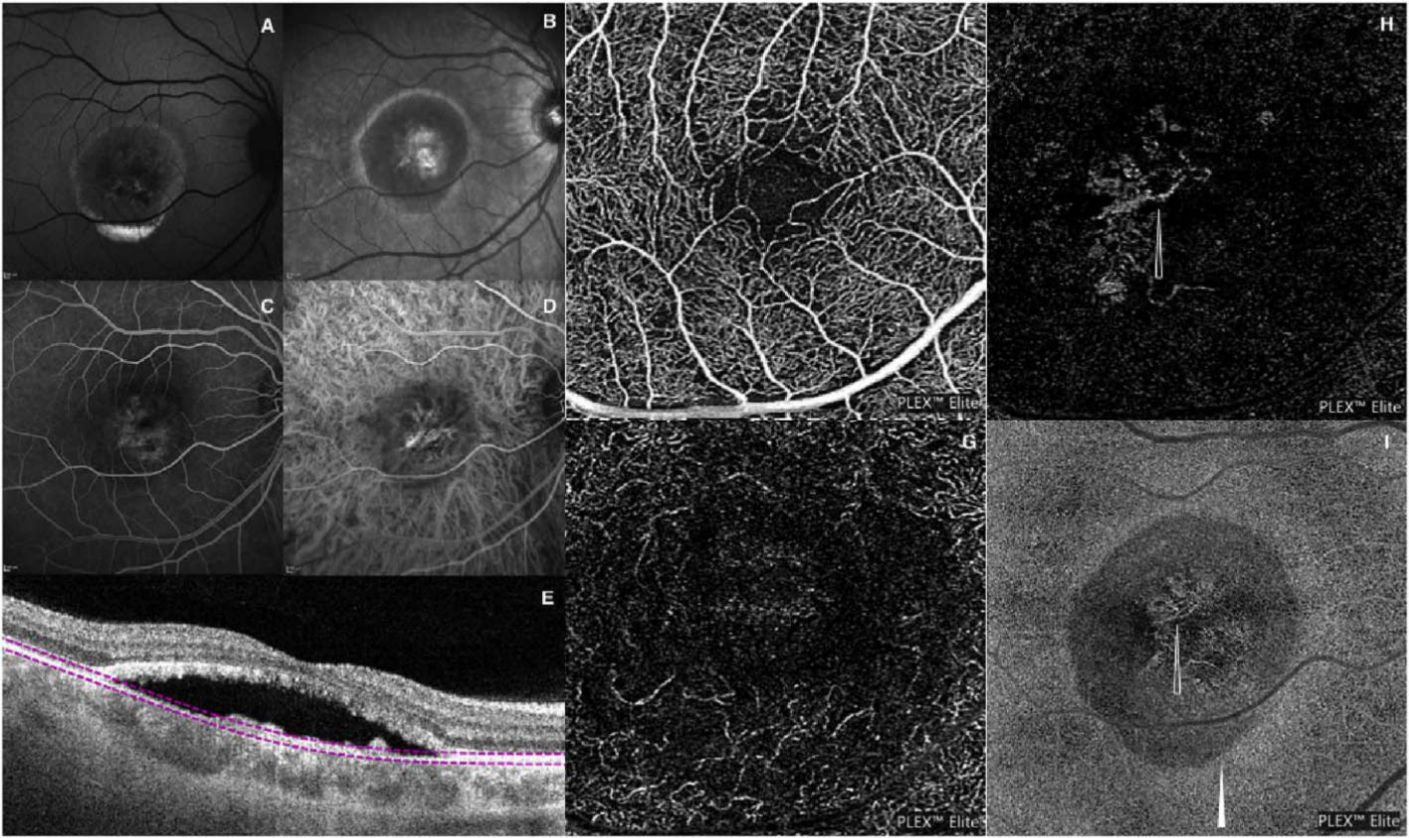
Multimodal imaging of patient with Best vitelliform macular dystrophy. Fundus AF (A) and infrared reflectance (B) show central mixed hyper/hypoautofluorescent lesion with mixed hyper-reflective and hyporeflective material. FA (C) and ICGA (D) reveal a round hypofluorescence area with central hyperfluorescence as window defect and staining, but no leakage. Spectral domain optical coherence tomography (E) shows a flattened lesion by the resorption of the majority of fluid, between retinal pigment epithelium (RPE)/Bruch’s membrane and the ellipsoid zone of the photoreceptors with subtle elevation of the RPE. Optical coherence tomography angiography shows the displacement of blood vessels at both the superficial (F) and deep capillary plexuses of the retina (G). Choriocapillaris (H) reveals a choroidal neovascularization (arrow) vascular network, while choroid segmentation (I) shows a well-defined, large circular area corresponding to the limits of the retinal elevation due to limitation of light penetration.

The material is hyperautofluorescent on fundus AF while on FA and ICGA, lesions can have blocked fluorescence due to the presence of the vitelliform material and pigment in the foveal area, and this can be surrounded by a ring of defective transmission corresponding to an area of atrophied RPE. In the recirculation phase, staining of the vitelliform material by the fluorescein dye is often evident, and the staining pattern can be mistaken for CNV. At the same time, CNV may complicate the natural course of VMD (Miller et al., 1976; Noble et al., 1978). In this case, a combination of clinical assessment and multimodal imaging may be required in order to confirm or exclude the presence of CNV. OCTA is helpful in this regard because it is not impeded by staining as seen in dye based angiography (Fig. 72). It allows visualization of a vascular network which may be masked by the vitelliform material on both FA and ICGA.

#### 11.10.2. Stargardt disease

Recessive Stargardt disease (STGD) is the most common inherited retinal dystrophy and is caused by mutations in the ABCA4 gene. A reduction in ABCA4 activity in the photoreceptor outer segments results in the increased production and accumulation of A2E and related bisretinoids within RPE cells. The result of these pathophysiologic events is a progressive loss of RPE and photoreceptors (Sparrow et al., 2008; Weng et al., 1999).

In STGD, FA is characterized by the absence of normal background choroidal fluorescence mainly due to the presence of RPE lipofuscin that absorbs the blue excitatory light (Fish et al., 1981). ICGA is able to define the lesion as ICG emits light in the near-infrared spectrum, and allows better penetration (Holz et al., 1998; Schwoerer et al., 2000). In particular, lesions are characterized by the presence of late hypofluorescence (dark atrophy) in late ICGA, possibly indicating a greater amount of choriocapillaris loss than what is typically seen in atrophy related to AMD (Giani et al., 2012).

**Fig. 73.**
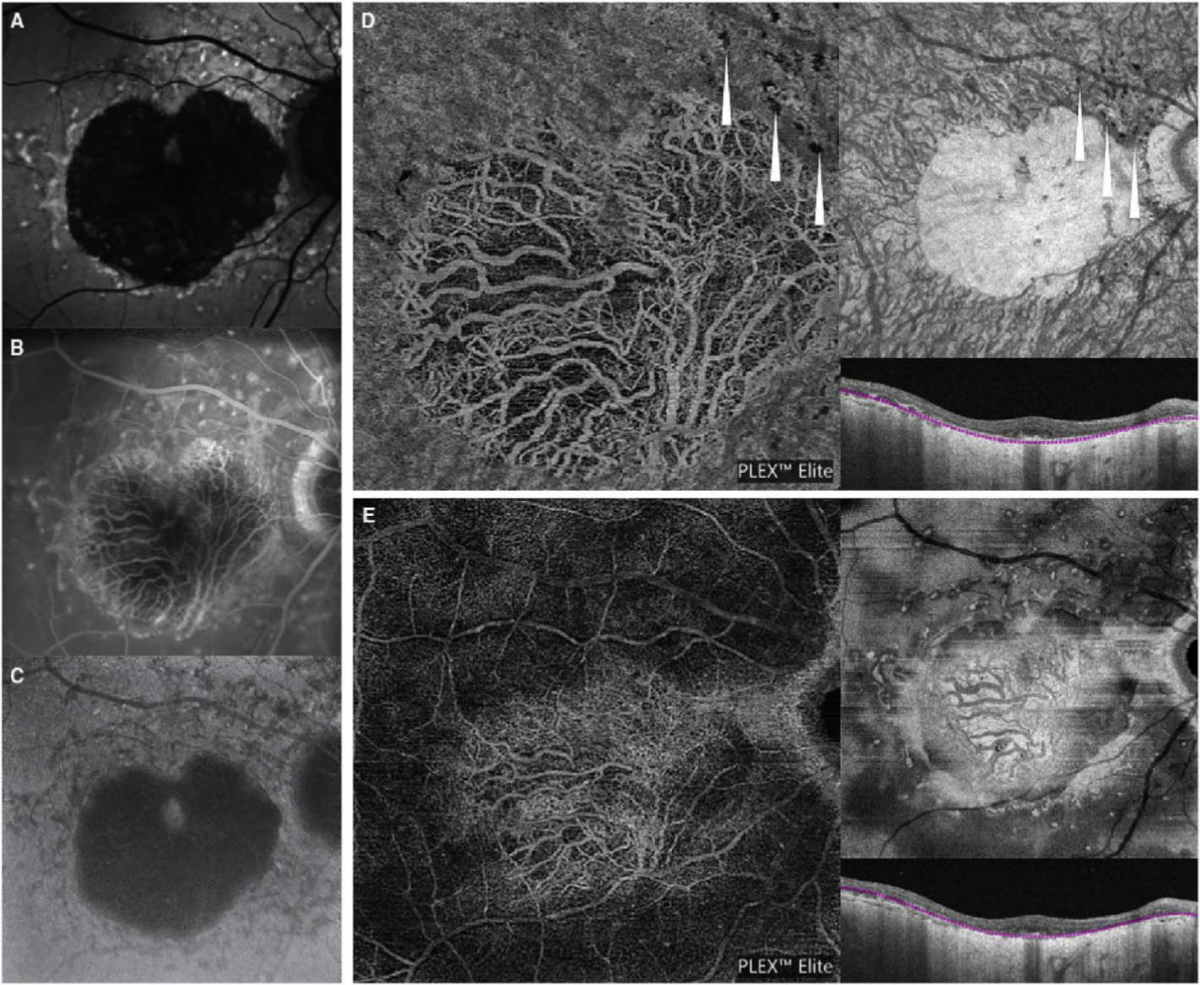
Optical coherence tomography angiography (A), fundus AF (B), FA (C) and ICGA (D) of a patient with Stargardt disease. Optical coherence tomography angiography shows no residual choriocapillaris inside the areas of atrophy where large choroidal vessels are clearly visible. Outside these regions, choriocapillaris lobules appear normal in density (A). Fundus AF (B) reveals a large hypoautofluorescent area due to the loss of the retinal pigment epithelium. FA (C) and ICGA reveal the hypofluorescence area, confirming the absence of choriocapillaris.

Although FA and ICGA are common clinical techniques to study retinal and choroidal vasculature, these imaging modalities are not able to image vessels deep to the RPE very well. OCTA displays severe loss of choriocapillaris within the area of atrophy in eyes affected by STGD (Fig. 73). Residual lobules of choriocapillaris can be found at the inner margins of the atrophic area, where choriocapillaris appear normal in density. At the same time, ICGA shows the presence of residual lobules of choriocapillaris at the edges of the area of RPE atrophy identified in fundus AF revealing a typical isofluorescent ring. In STGD, RPE cells appear to atrophy, and the choriocapillaris and Sattler’s layer consequently disappears (Pellegrini et al., 2016).

#### 11.10.3. Retinitis pigmentosa

Retinitis pigmentosa (RP) is heterogeneous group of hereditary dystrophies characterized by the progressive degeneration of the photoreceptors (rods and cones) leading to progressive visual loss (An and Wang, 2008; Mariampillai et al., 2008). Typical symptoms of RP include night blindness, photopsia, and progressive visual field loss; however, vision can also be affected by cataracts and cystoid macular edema (Berson, 1993; Hartong et al., 2006). The clinical features of typical RP include arteriolar narrowing, “waxy pallor” of the optic nerve head, alteration in pigmentation and atrophy of the retinal pigment epithelium and migration into the retina with intraretinal clumping of melanocytic cells, appearing most often as coarse clumps in a “bone spicule” configuration.

**Fig. 74.**
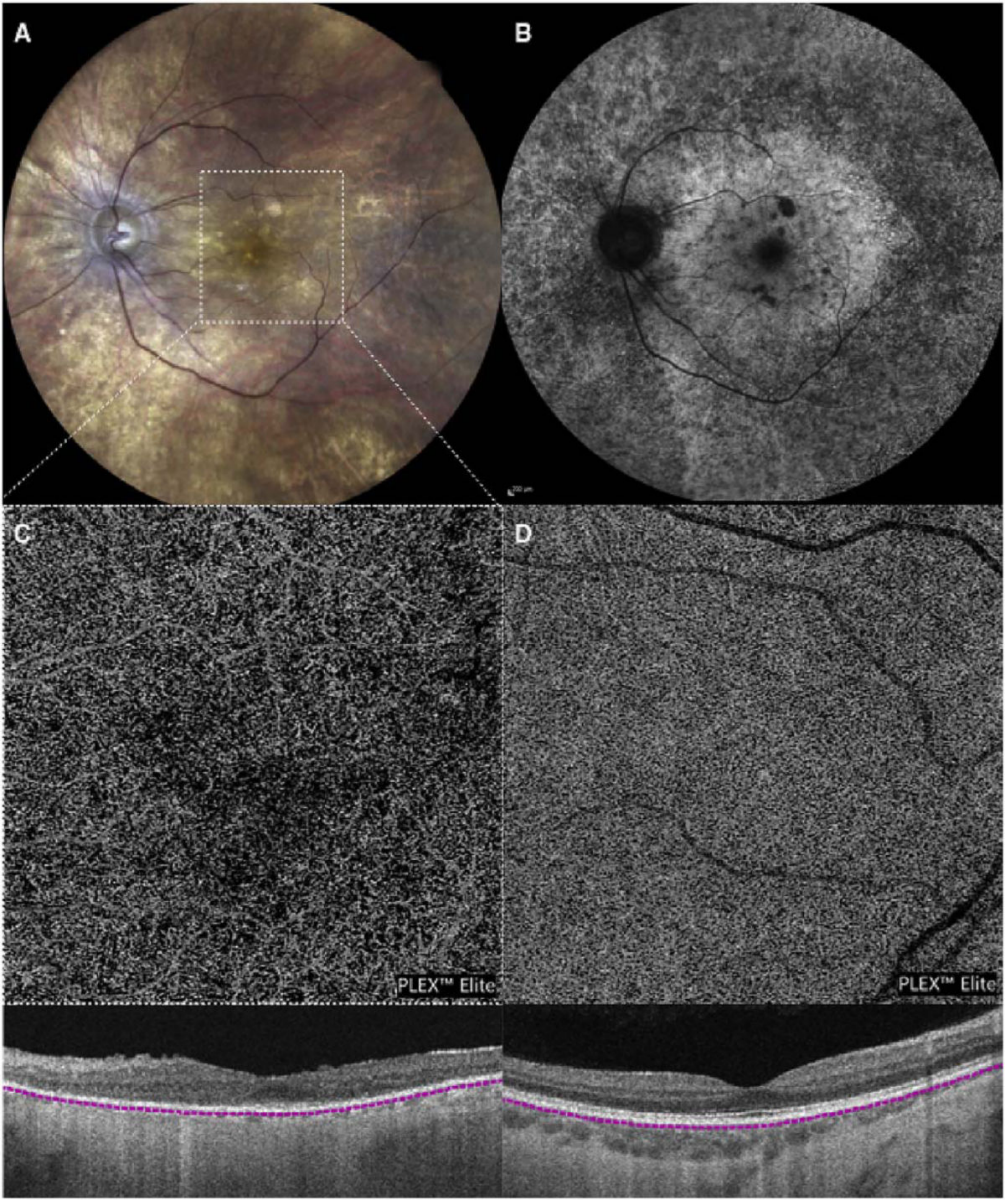
Optical coherence tomography angiography of a patient with retinitis pigmentosa. Image of the fundus (A) and blue light fundus AF (B) of a patient with retinitis pigmentosa. OCTA shows narrowed vessels with a progressive vascular rarefaction, from the papilla towards the periphery at the superficial and deep capillary plexuses (C and D, respectively). No vascular abnormalities were detectable at the choriocapillaris and choroid segmentation (E and F, respectively).

OCTA allows the detection of flow abnormalities and the vascular anatomy in both the retina and choroid; to date there has not been much in the published literature concerning RP (Parodi et al., 2017). OCTA shows narrowed vessels in the superficial capillary plexus of the retina and a progressive vascular rarefaction, from the papilla towards the periphery, of the superficial and deep capillary plexuses (Fig. 74). In macular edema, OCTA reveals focal dislocation of the vascular network at the superficial capillary level in correspondence of the intraretinal cysts. In this case, the fluid accumulates between the inner plexiform layer and the outer plexiform layer causing a preferred distortion of the deep capillary plexus compared to superficial. OCTA is an imaging tool with able to show both retinal and choroidal vascular structures.

### 11.11. OCT angiography of the optic nerve

The nerve fibers in the retina are unmyelinated; from a teleologic standpoint myelination would degrade the image produced by the underlying photoreceptor layer. However unmyelinated retinal nerve fibers require large amounts of energy to maintain ion concentration gradients (Andrews et al., 1999; Bristow et al., 2002; Carelli et al., 2013). The retinal nerve fiber layer is invested by the radial peripapillary capillary network to supply oxygen and metabolites to the nerve fiber layer. As the capillary circulation of the optic nerve head and the peripapillary region are well-seen by OCT angiography, there is an opportunity to use OCTA to evaluate microvascular abnormalities in various optic nerve disorders. It is possible that the vascularity of the nerve fiber layer, the radial peripapillary network, may serve as a surrogate measure for the metabolic activity in that area. The metabolic activity in turn may correlate with the number and vitality of the nerve fibers present.

Jia and colleagues (Jia et al., 2012a) demonstrated a reduction in optic nerve head vessel density and flow index (average of the decorrelation values) in perperimetric glaucoma patients compared to normal controls. Their method was to reduce the thickness of the radial peripapillary capillary network by using maximal intensity projection and then to quantify the proportion of area occupied by the detected microvascular flow. Leveque and colleagues also observed a 20–25% reduction in ONH vessel density in a cohort of glaucoma patients compared with normals (Lévêque et al., 2016). The vessel density reduction also correlated with visual field parameters as well as structural OCT findings in these subjects. Bojikian et al. compared three different ONH perfusion metrics (flux, vessel area density, and normalized flux) among normal, open angle glaucoma (OAG), and normal tension glaucoma (NTG) subjects (Bojikian et al., 2016; Chen et al., 2016a). While ONH perfusion was significantly reduced in both glaucoma groups compared to normal, there was no difference between NTG and OAG groups with similar levels of visual field loss. Rather than evaluate the ONH vessel density, Akagi et al. quantified the peripapillary capillary density in glaucomatous eyes and normals (Akagi et al., 2016). The reduction of peripapillary density in the glaucomatous eyes (without high myopia) spatially co-localized with the region of the visual field defect. These studies as well as others have highlighted the potential value of OCTA in better characterizing patients with glaucoma. OCTA lacks standardized methods to measure vascular density and flow and consequently nomograms for these indices are not yet available. As a consequence, simple metrics such as sensitivity and specificity in diagnosing glaucoma are not known at present.

The best use of OCTA in the management of glaucoma, however, still needs to be defined in careful longitudinal studies with multimodal imaging. One potential application is diagnosis of glaucoma in high myopes, which can be challenging. High myopes can have schisis around the nerve and expansion and distortion of the nerve head which can make nerve fiber layer thickness measurements impossible to obtain. It is possible that vascular density of the radial peripapillary capillary network may prove to be a suitable surrogate measure of nerve fiber thickness. Some studies have used OCTA as a means of differentiating among normals, preperimetric glaucoma or ocular hypertension, and glaucoma (Akil et al., 2017; Chihara et al., 2017). The diagnosis of preperimetric glaucoma (or ocular hypertension) is based on a normal visual field, using a test with imperfect sensitivity and specificity. It is possible that with follow-up studies there are features of OCTA imaging of the radial peripapillary capillary network that will predict or augment the diagnostic capability of the visual field test. Lamina cribrosa defects occur in glaucomatous eyes and these defects are predictive of future field loss. Eyes with lamina cribrosa defects have circumpapillary perfusion defects that spatially correlate to the defect in the lamina (Suh et al., 2016).

**Fig. 75.**
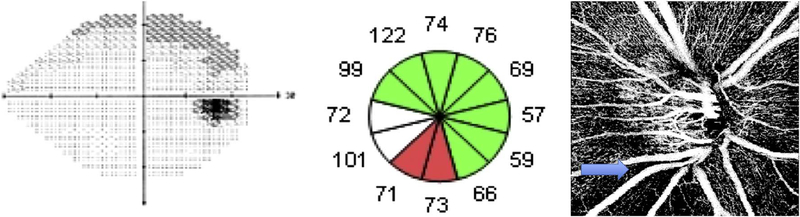
Retinal nerve fiber layer and radial peripapillary capillary changes in glaucoma. Left panel. Anarcuate visual field defect is present. Middle panel, the retinal nerve fiberlayer analysis shows a region of inferotemporal retinal nerve fiber layer thinning.Right panel. The redial peripapillary capillary network shows a sector defect(arrow) corresponding to the nerve fiber layer defect.

**Fig. 76.**
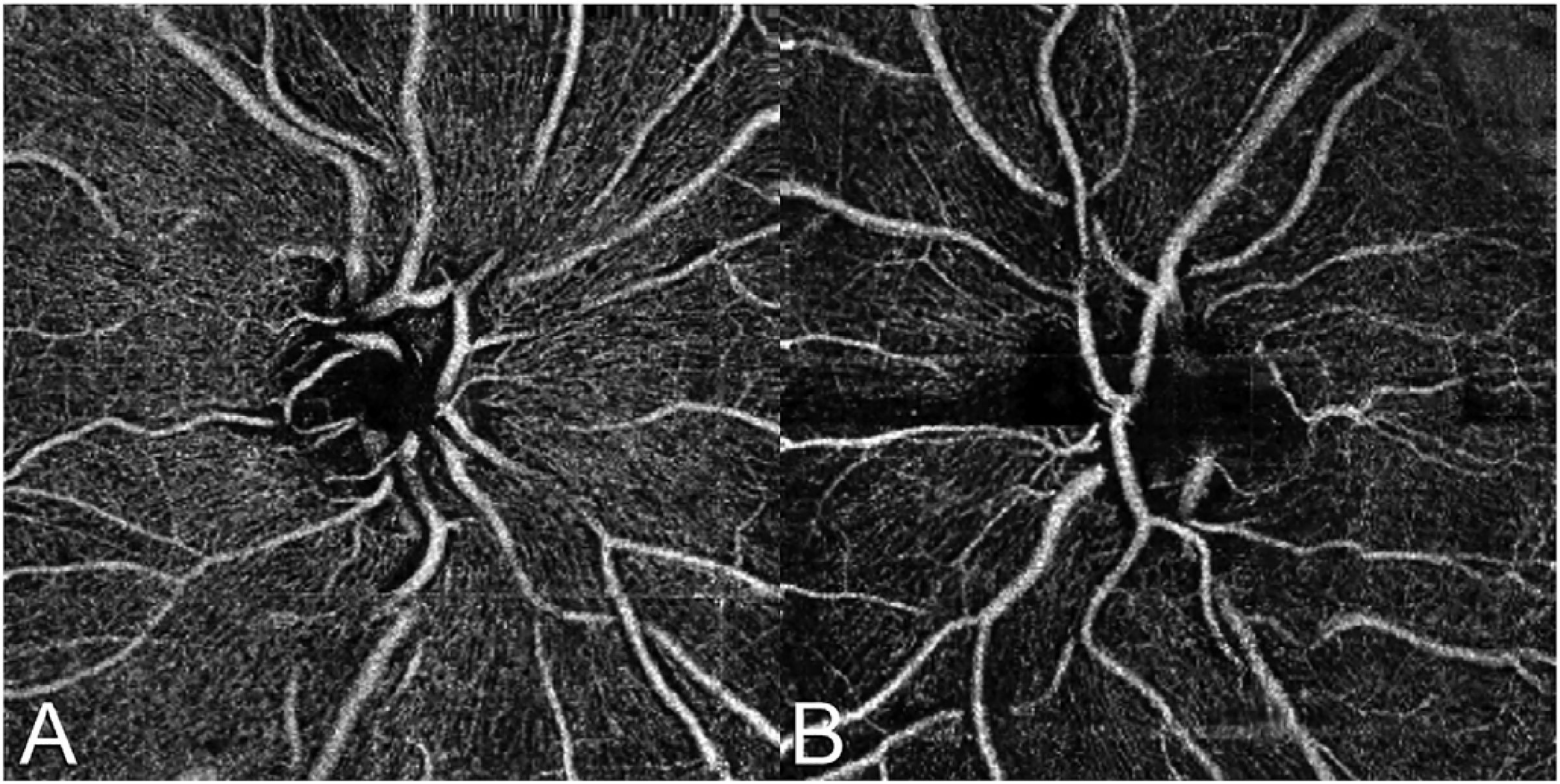
Optic nerve damage in a 38-year-old with a history of optic neuritis. A. The normal uninvolved eye shows a dense pattern of radial peripapillary vessels. B. The involved eye shows a global loss of the radial peripapillary capillaries.

**Fig. 77.**
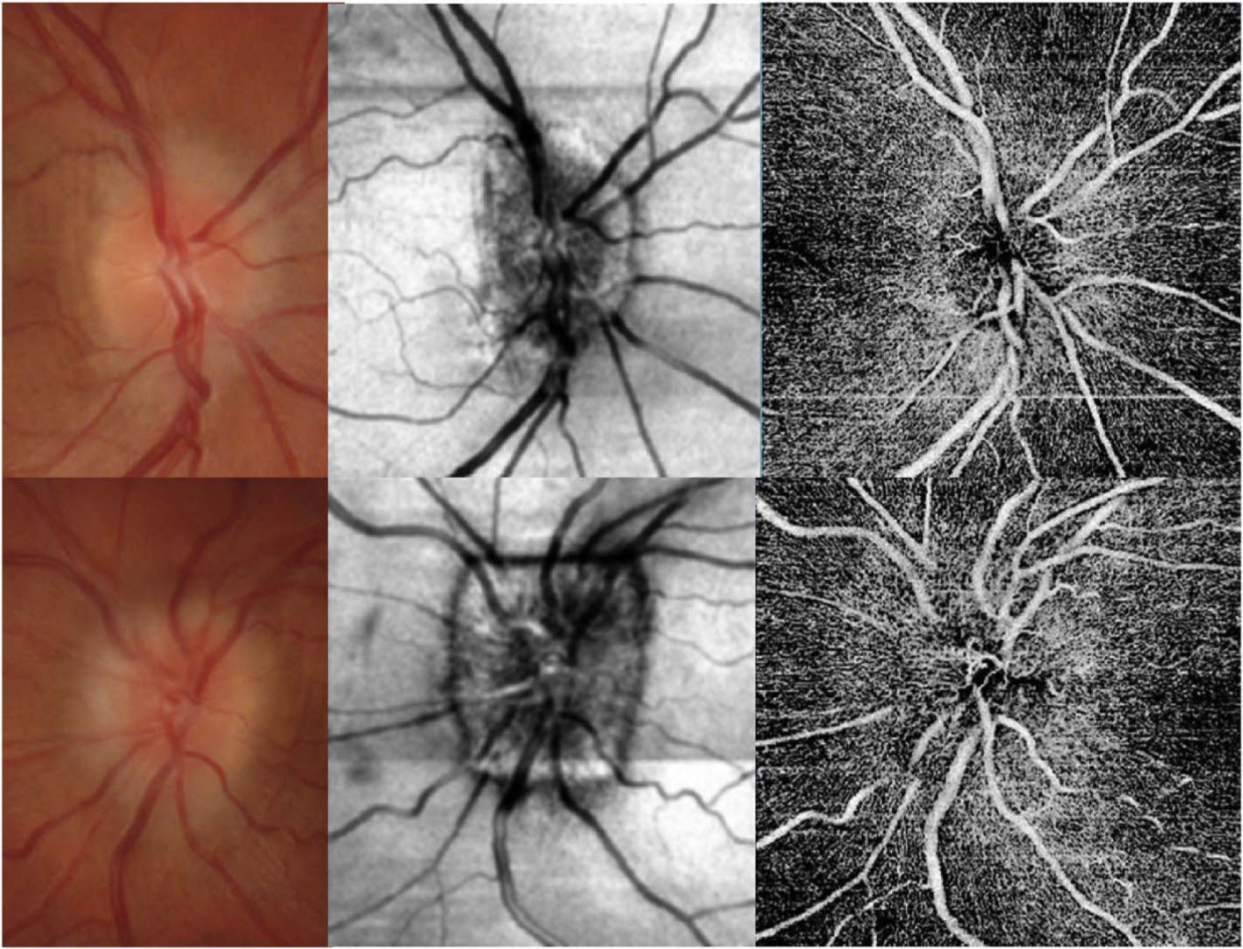
Both eyes of a patient with papilledema due to idiopathic intracranial hypertension (top row – right eye, bottom row – left eye). Color photos (left panels) demonstrate the bilateral disk edema. Disk edema is also evident on the en face OCT images (middle panel). OCT angiogram (slab from internal limiting membrane to lamina cribosa) demonstrates increased prominence of the optic nerve head and peripapillary capillaries.

OCTA has also been used in the evaluation of other optic nerve and central nervous system disorders. Wang and colleagues observed an apparent reduction in the flow index in patients with multiple sclerosis (Wang et al., 2014). Optic neuritis may be associated with significant loss of the radial peripapillary capillaries (Fig. 75). In patients with papilledema for example due to idiopathic intracranial hypertension, OCTA can demonstrate an increased prominence of the ONH and peripapillary retinal capillaries (Fig. 76). In contrast, patients with disk edema from anterior ischemic optic neuropathy may demonstrate some patchy loss of ONH capillaries (Fig. 77). Treatment of ischemic optic neuropathy is difficult, in part, because it is difficult to evaluate the perfusion of the nerve head. OCTA offers the potential to evaluate flow in a precise and longitudinal manner.

**Fig. 78.**
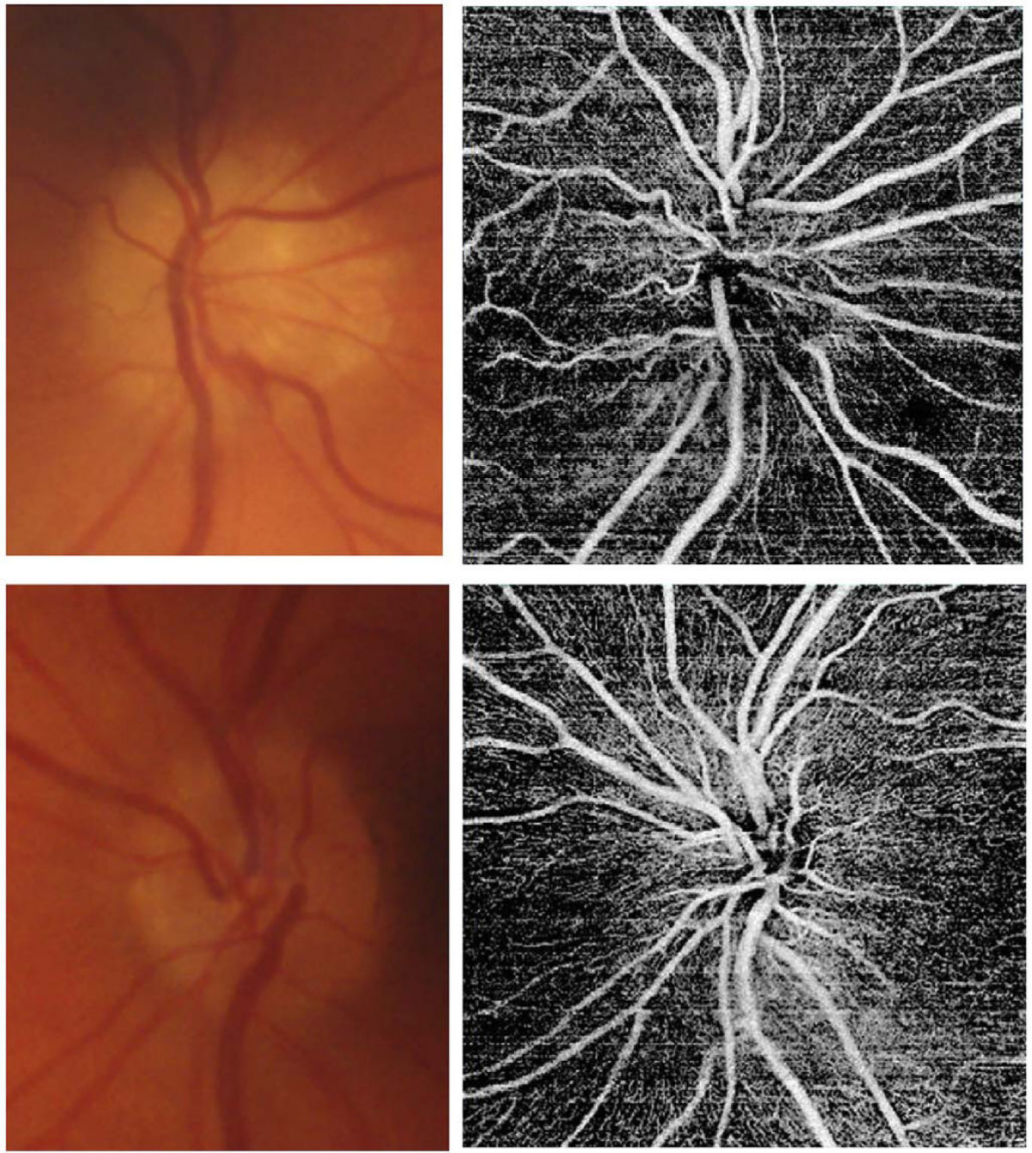
Right (top panels) and left eyes (bottom panels) of a patient who presented with sudden vision loss in the right eye. Patient was noted to have disk edema in the right eye (top right panel) and very small cups (both eyes). A diagnosis of anterior ischemic optic neuropathy was made. OCT angiogram demonstrated patchy loss of ONH capillaries in the right eye (top right panel) compared to the normal left eye (bottom left panel).

**Fig. 79.**
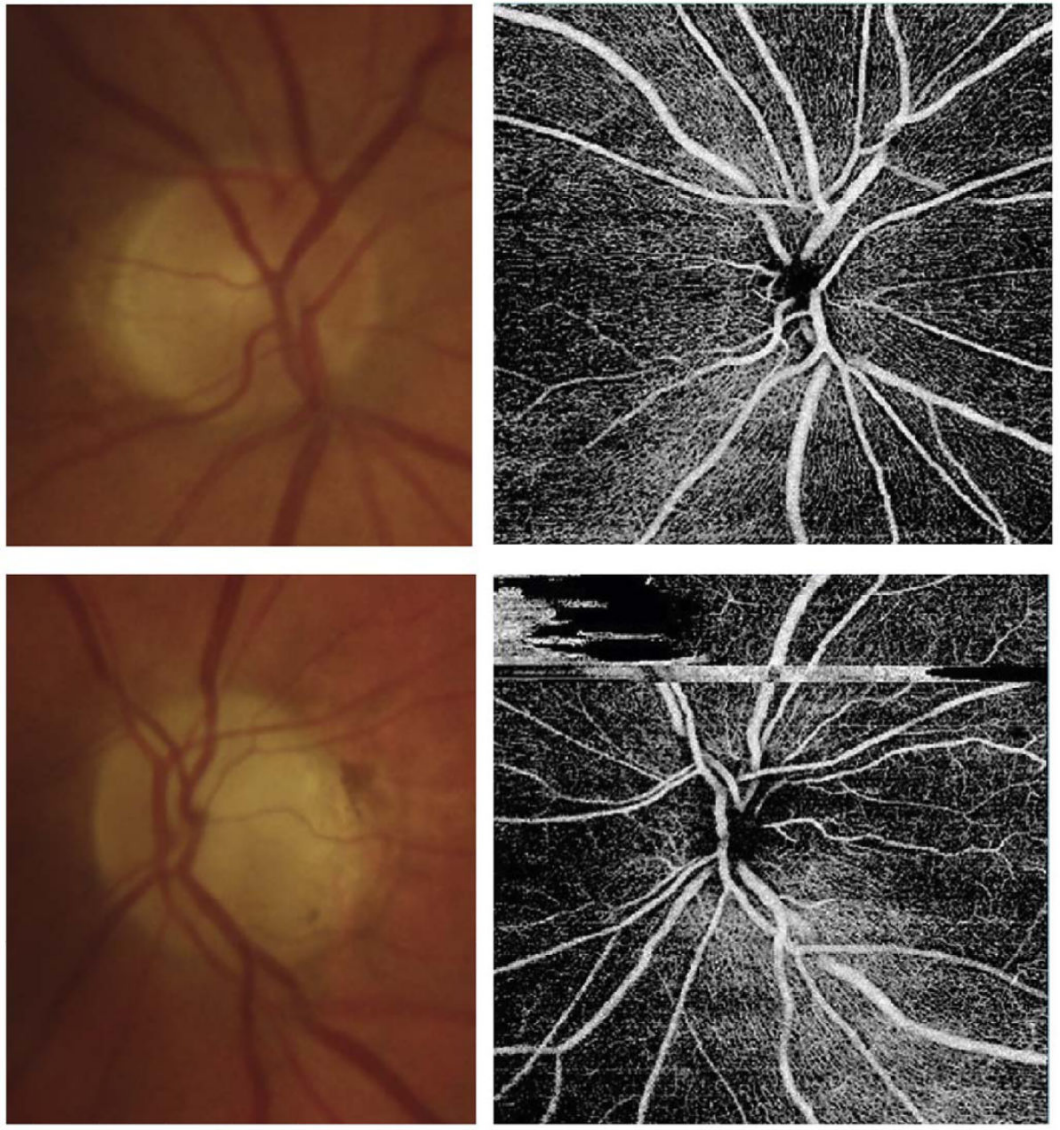
Patient with optic atrophy in the left eye following anterior ischemic neuropathy (Right eye – top panels, Left eye – lower panels). Pallor of the left optic nerve is evident (lower left panel), most prominent superotemporally. The optic nerve head capillary density on OCT angiography is decreased in this superotemporal region (lower left panel) compared to the normal fellow eye (upper right panel).

With optic atrophy from various etiologies, OCTA demonstrates loss of capillaries in the region affected by the injury. This leads to characteristic patterns with certain diseases such as segmental or altitudinal ONH capillary loss in anterior ischemic optic neuropathy (Fig. 78), and capillary loss in the temporal ONH and papillo-macular bundle in cases of dominant optic atrophy. Patients with Leber’s Hereditary Optic Neuropathy may show an evolution of findings on OCTA with increased prominence of the peripapillary capillaries in the acute and subacute phases, and prominent loss of the optic nerve head capillaries in the chronic phase. We can expect the potential OCTA applications for the evaluation of optic nerve disorders to continue to expand with new insights gained into disease pathophysiology and response to therapy. The vascular vs mechanical contributions to the pathogenesis of glaucoma remain unresolved and may be further elucidated with OCTA. OCTA offers the potential to be able to evaluate the blood flow in the prelaminar nerve as well as elsewhere in the pathway from the retinal nerve fiber layer to the postlaminar nerve and to gauge effects of various medications on flow which may open the opportunity for the development of novel therapeutic agents (see Fig. 79).

## 12. OCTA in clinical practice

This paper described the past development of OCTA and current research. Investigations of new imaging modalities often follow a common pattern. The first papers often compare a new imaging technique to an old technique to determine if the new method can image the same things as the old. In case of diabetic retinopathy these studies might examine if microaneurysms are visible and in what layer. These types of papers establish a foothold for the new imaging modality, but have little clinical utility. Imaging enthusiasts are happy because it is interesting to image the eye. A cost-conscious practitioner may well wonder about the need for the modality given the ease of looking in the eye as a simple means of detecting microaneurysms. The next level of papers follows with features of disease that couldn’t have been determined using older methods of imaging. Imaging aficionados and researchers in the particular field are intrigued and elated. The costconscious practitioner is mystified because the new imaging approach doesn’t directly lead to any measurable benefit, medically or financially.

The next stages of development evaluate the imaging modality in terms of enhanced diagnosis of disease or a clue to new approaches in managing disease. These studies often take years. Not all are successful. The people in imaging are often centrally involved, while the costconscious practitioner has long lost interest. Over time, practical and necessary uses of the imaging modality can start to crystalize, making the approach indispensable. At that point everyone wants to adopt the technology, even the practical practitioner. Note the adoption of a new imaging technology is somewhat different than what is described as the Gartner hype cycle (http://www.gartner.com/technology/research/methodologies/hype-cycle.jsp).

OCT angiography is still in the earliest stages of development. There are several indications in which OCTA is better than or equivalent to accepted approaches and many instances where it shows us findings that were unexpected. None of these older imaging modalities can be replaced by OCTA at present. That raises questions about the need for OCTA. One answer to this question is to start thinking about how treatments of today started. Ideas based on imaging along with pathophysiology were at the heart of many modern ophthalmic treatments. Imaging from the 1970’s and 80’s formed the basis of larger studies, which led to theories of disease. In the 1990’s these were used to form plans of attack, pharmacologic, surgical, or both, that were eventually implemented after numerous trials many years later. Our treatments today are better than what came before, but revolutionary advances from what we have now is not likely to come from mining ideas of the 1970’s. The basis for treatments decades in the future are being laid now with our current efforts including OCTA.

The rate of change in OCTA over the last few years has been phenomenal. The safest and easiest way to predict the future is to imagine a continuation of the immediate past. Henry Ford’s apocryphal quote, “If I had asked people what they wanted, they would have said faster horses” illustrates the main problem. It is impossible to predict new disruptive technology or what effect it may have. Sometimes it seems like one can notice disruptive technology when it is just starting. It seems like OCTA is a truly disruptive technology. It is powerful. It is exciting (at least to the authors, all imaging zealots). It is probably going to change the practice of retinal diseases. One really interesting part of OCTA is that it is relatively inexpensive and available to nearly everyone. So, its development can and will be a group effort. Let’s get to work!

## Figures and Tables

**40.**
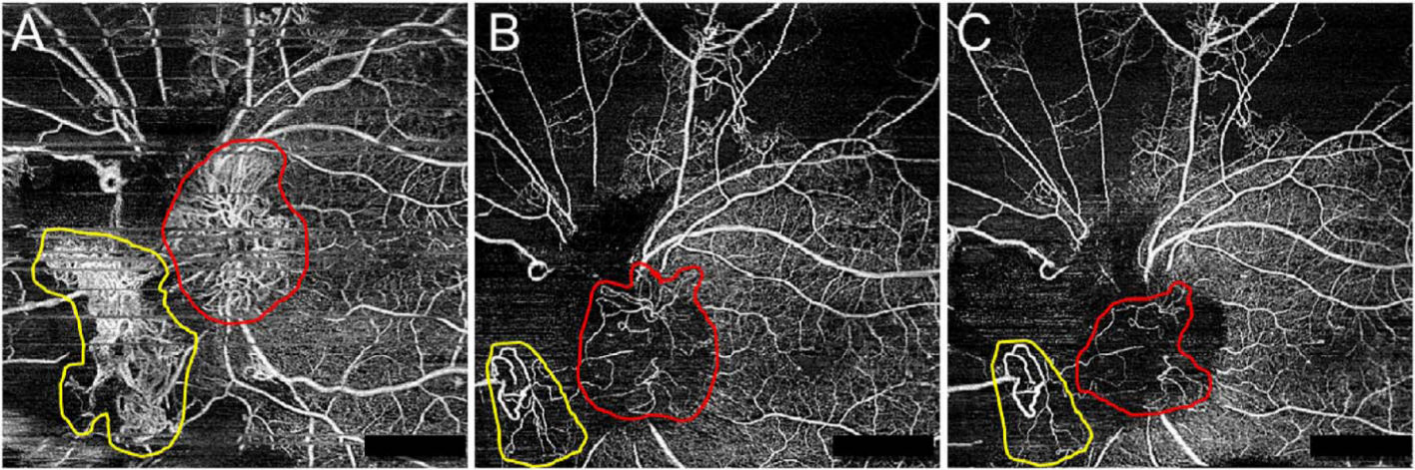
Neovascularization of the disc is seen in this OCTA image projecting into the vitreous space (A). 4 and 8 weeks after treatment with anti-VEGF injections, the pre-retinal neovascularization is seen to regress (B and C panel respectively).

## Abbreviations

AF: Autofluorescence
AMD: Age-related macular degeneration
CCD: Charge-coupled device
CME: Cystoid macular edema
CMOS: Complementary metal oxide semiconductor
CNV: Choroidal neovascularization
CRORA: Complete loss of the RPE and outer retina
FA: Fluorescein angiography
ICG: Indocyanine green
ICGA: Indocyanine green angiography
MNV: Macular neovascularization; includes choroidal neovascularization and Type 3 neovascularization Optical
OCT: coherence tomography
OCTA: Optical coherence tomography angiography
OMAG: Optical microangiography
RPE: Retinal pigment epithelium
SD: Spectral domain
SS: Swept source
VEGF: Vascular endothelial growth factor
VISTA: Variable interscan time analysis
